# Political affiliation impacts climate attention and choice beyond extreme weather exposure

**DOI:** 10.1038/s41558-026-02765-z

**Published:** 2026-10-16

**Authors:** Annika M. Wyss, Emmanuel T. Guizar Rosales, Michael Schulte-Mecklenbeck, Julie Buchs, Ulf J. J. Hahnel, Daria Knoch

**Affiliations:** 1 Department of Social Neuroscience and Social Psychology, University of Bern, Bern, Switzerland; 2 Department of Consumer Behavior, University of Bern, Bern, Switzerland; 3 Max Planck Institute for Human Development, Berlin, Germany; 4 School of Sustainability, Leuphana University of Lüneburg, Lüneburg, Germany; 5 Faculty of Psychology, University of Basel, Basel, Switzerland

**Keywords:** Psychology, Decision making, Climate-change mitigation, Psychology and behaviour, Psychology

## Abstract

Political polarization and increasing exposure to extreme weather can shape public engagement with climate change, yet the underlying psychological mechanisms remain unclear. In this Registered Report, we examine how political affiliation, extreme weather exposure and subjective attribution influence climate-relevant information search and decision-making in a US sample (*N* = 1,451). We combined a process-tracing paradigm within an incentivized decision task involving real carbon emissions with objective data on extreme weather exposure. Democrats attended more to carbon emission information and made more pro-climate choices than Republicans, with no evidence of moderation by objective extreme weather exposure. The subjective attribution of extreme weather to climate change was associated with greater attention to carbon information, more pro-climate choices and moderated the effects of exposure on both outcomes. These findings highlight the importance of political affiliation in climate-relevant decision-making and suggest that extreme weather effects depend less on exposure itself than on how these experiences are interpreted.

## Abstract (web summary)

Mechanisms behind climate decision-making remain unclear. This study shows that political affiliation and subjective attribution shape attention allocation and climate-related choices, with subjective attribution moderating the effects of political affiliation and objective weather exposure.

## Main

As extreme weather events become more intense and frequent with climate change^1^, the role of such events in shaping public awareness and prompting climate action has been a subject of considerable debate^2–4^. Researchers have proposed that these events may act as catalysts for capturing attention, raising awareness and spurring action to combat climate change^5–8^. While evidence suggests that extreme weather events can influence climate change engagement, this influence seems to depend on individual differences such as a person’s political affiliation^6,9,10^. Especially in polarized political contexts such as in the USA, political affiliation may shape whether extreme weather is understood as evidence of climate change or dismissed as unrelated, thereby influencing how people seek and process climate-relevant information.

Among the individual factors that shape responses to extreme weather, political affiliation is one of the strongest predictors of climate change beliefs^11^. Individuals aligned with more liberal (versus conservative) political parties are more likely to believe in climate change and to support climate-relevant behaviours and policies^12–14^, particularly in the USA^14,15^. Despite extensive evidence linking political affiliation to climate beliefs and behaviours, much less is known about how it shapes the psychological processes through which people seek out, attend to and use climate-relevant information, particularly in the context of varying exposure to extreme weather events. These processes may include whether individuals notice and evaluate the carbon footprint of products before purchase, compare environmentally responsible investment opportunities or consider political candidates’ positions on climate action. Understanding how political affiliation influences these information-processing pathways may help explain why similar environmental experiences lead to different patterns of climate engagement and decision-making.

To address this gap, our study investigates how individual characteristics (that is, political affiliation) and physical-level determinants (for example, exposure to extreme weather) jointly shape climate-relevant information search and decision-making. In doing so, we respond to calls for climate-behaviour research that accounts for the variety, complexity and interconnectedness of behavioural determinants^16,17^. We use visual attention to climate-relevant information as an indicator of these underlying processes measured with a MouselabWEB paradigm, which allows us to examine how people allocate attention to may provide insights relevant to future research on communication strategies and policy measures aimed at fostering bipartisan engagement in climate action^18,19^.

Accordingly, this study seeks to address three core research questions: (1) Does political affiliation impact how individuals search for and attend to information during climate-relevant decision-making? (2) Does exposure to extreme weather events interact with political affiliation to shape these information search processes? (3) Does the interaction of exposure to extreme weather events and political affiliation on information search processes depend on individuals’ subjective attribution of these events to climate change?

While the primary emphasis of this study is on information search processes (that is, attention allocation), we also aim to examine how climate-relevant choices (that is, the outcomes of preceding decision-making processes) are influenced by these factors. Specifically, we aim to explore how political affiliation, exposure to extreme weather events and the subjective attribution of such events to climate change impact the actual choices made. In doing so, we provide a comparative analysis of how attention allocation during information search and the resulting choices are shaped by these variables and their interactions.

To answer our research questions, a representative sample of US participants completed a new variant of the Carbon Emission Task (CET)^20^ while we tracked their decision-making processes using web-based process-tracing technology (MouselabWEB)^21^. The CET is a paradigm that has been shown to be externally valid^20^, as it mirrors the critical dynamics and interdependencies between actions and outcomes that characterize many real-world, climate-relevant decision-making situations^22–25^. Many everyday choices reflect this balance between short-term convenience and long-term sustainability—such as driving a car instead of walking or biking to reduce emissions, or taking long, hot showers despite their higher water and energy consumption (see Supplementary Information, section 2.3.1 for more examples).

**Fig. 1:**
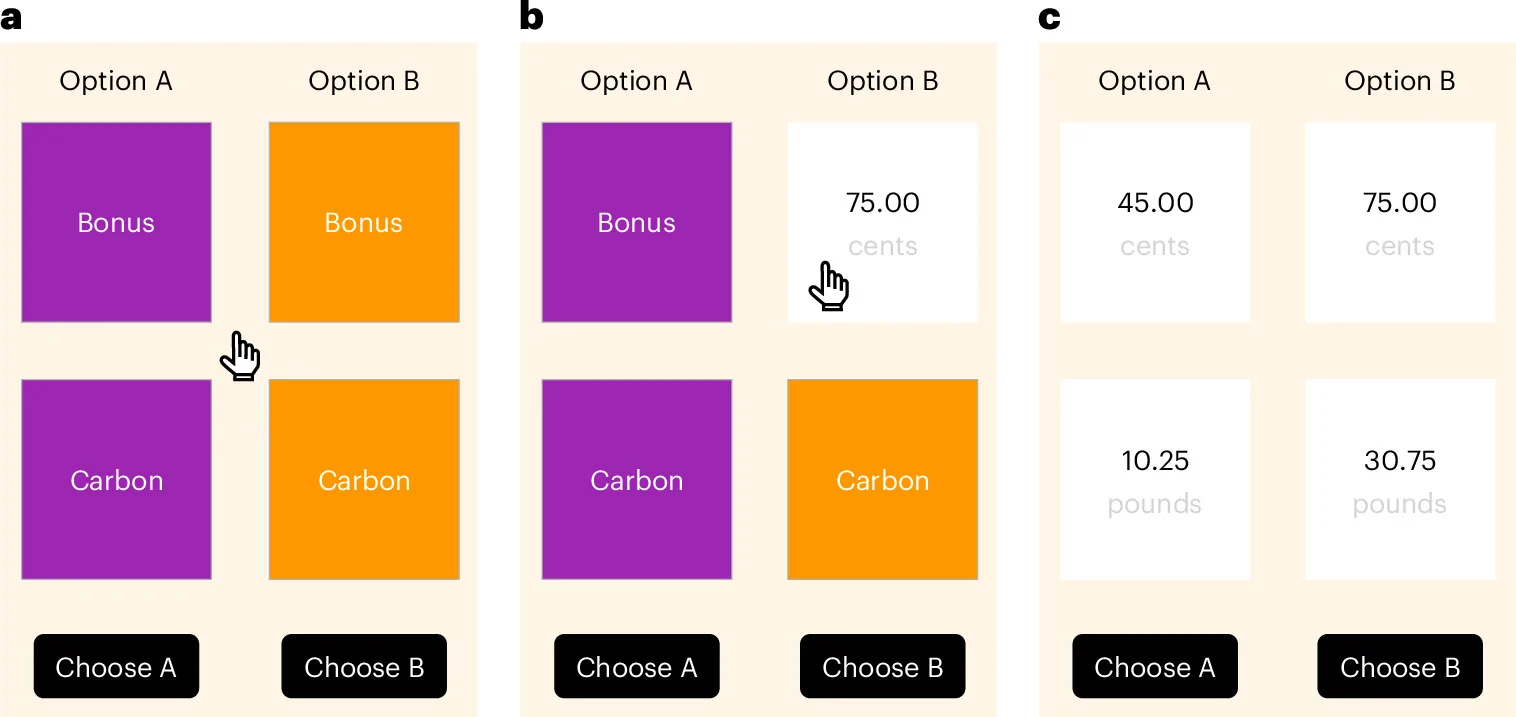
Example trial of the new variant of the CET. Participants choose between two options with different bonus payments and carbon emissions. **a**, Initially, this information is occluded by boxes. **b**, Participants can reveal the specific details of each option by hovering over the boxes with their mouse. Moving the mouse outside of a box occludes the information again. **c**, By visiting all boxes, participants can acquire all available information in a trial (all boxes opened simultaneously for demonstration purposes only). The position of the bonus and carbon information (top versus bottom) is randomized between participants, while the position of the option maximizing the bonus (left versus right) is randomized within participants between trials. Each trial starts by clicking a fixation cross button in the centre of the decision matrix, ensuring equal distance for all box visitations.

Importantly, the carbon emissions in the task are real and are implemented by trading carbon emission certificates in a cap-and-trade system, where polluters must hold emission certificates to offset their carbon output^20,26^ (Methods). Using a MouselabWEB paradigm, information about bonus payments and carbon emissions is concealed and revealed only when participants move the mouse cursor over the corresponding attribute^21^ (Fig. 1). Here we focus on dwell time—an established measure of attention allocation during information search—to examine how participants allocate attention to bonus payments and carbon emissions. This measure allows us to capture the psychological trade-offs individuals make between personal benefits and climate consequences during decision-making.

To examine how attention allocation is shaped by both political affiliation and objective exposure to extreme weather events, we linked participants to county-level records from the National Oceanic and Atmospheric Administration. These data provide high-resolution records of objectively measured extreme weather events and have previously been used to study climate-related perceptions and behaviour^27,28^. For each participant, we assessed exposure during the 30 days preceding study participation (and up to 360 days in sensitivity analyses), while also deriving measures of event severity and abnormality.

We formulated three hypotheses corresponding to our primary (and secondary) research questions (summarized in the Design Table in Supplementary Information, section 2.6). Theoretical accounts of climate change polarization propose that political attitudes shape attention to climate-relevant information, influencing climate-related judgments and behaviour^29–31^. Consistent with this view, prior research shows that liberals and conservatives differ in their attention to climate information. In an attentional blink task, liberals concerned about climate change more accurately identified climate-related than neutral words, whereas conservatives with low climate concern showed no such advantage^32^. Similarly, eye-tracking research found that liberals, compared with conservatives, allocated more visual attention to periods of rising rather than stable global temperatures^30^. Together, these findings suggest that political attitudes shape attention to climate-relevant information. Accordingly, we hypothesized:

Political affiliation hypothesis (H_1_): Democrats (compared to Republicans) prioritize attention to carbon over bonus information in the CET.

Previous research on the relationship between various metrics of exposure to extreme weather and climate beliefs, attitudes and behaviours has produced mixed results. While some studies found that exposure to extreme weather increased climate change concern and engagement^28,33–35^ and attention to climate change in news media, social media content and Google searches^27,36–38^, others reported little to no evidence for such relationships^39–43^. These inconsistencies may arise because the effects of extreme weather depend on other factors^4,10,44^, particularly political affiliation and subjective attribution of events to climate change^9,45–48^. For example, exposure to wildfires increased support for climate-related ballot measures only in Democratic voting areas^49^, and extreme weather generated more climate-related discussion in Democratic than Republican communities, especially when the events were attributable to climate change^6^. Similarly, personal experience with severe flooding predicted mitigation responses only among individuals who attributed the event to climate change^47^. Thus, previous research suggests an intricate interplay between political affiliation, exposure to extreme weather events and their attribution in affecting engagement with climate change. However, most previous studies relied on self-reported measures of both exposure and attribution, raising concerns about common method bias^23,26^. By combining objective measures of extreme weather exposure with subjective attribution, our study addresses this limitation. To this end, we test whether extreme weather exposure amplifies political differences in attention allocation and climate engagement, and whether this relationship depends on subjective attribution. Accordingly, we derived the following hypotheses:

Extreme weather hypothesis (H_2_): The difference in time spent on carbon versus bonus information in the CET increases after exposure (versus without) to extreme weather events between Republicans and Democrats.

Subjective attribution hypothesis (H_3_): The amplifying effect of exposure to extreme weather events on the polarization in information search processes between Republicans and Democrats is more pronounced for individuals who attribute these events more strongly to climate change.

In addition to examining information search processes, we also aimed to investigate how these factors—political affiliation, exposure to extreme weather events and the subjective attribution of these events to climate change—affect the actual choices participants make in the CET. Specifically, we propose that the same interactive dynamics observed in information search will extend to choices in the CET. Thus, we formulate the following secondary hypotheses for climate-relevant choices:

H_S1_: Compared to Republicans, Democrats are more likely to make choices that prioritize climate protection over personal financial gain in the CET. That is, Democrats have a higher probability of making pro-climate choices compared to Republicans.

H_S2_: Exposure to extreme weather events amplifies the polarization in climate-relevant choices, increasing the difference in climate-protecting choices made by Republicans and Democrats.

H_S3_: The amplifying effect of exposure to extreme weather events on the polarization in climate-relevant choices between Republicans and Democrats is more pronounced for individuals who attribute these events more strongly to climate change.

## Results

Data were collected between August and September 2025. The sample (*N* = 2,000) was quota-matched to the US population on political affiliation, age, sex and region, with a deviation tolerance of <5%. After applying all exclusion criteria, the final analytic sample consisted of *n* = 1,451 respondents (see Methods for details). The study closely followed the approved Stage 1 protocol, with the sole deviation involving the operationalization of the recency control variable for extreme weather exposure (‘Implementation’).

In the following, we report detailed results from the baseline models only. A full set of sensitivity analyses—including models with control variables and analyses using the full (pre-exclusion) sample—is reported in Supplementary Information, section 1, unless these analyses yield substantively different conclusions.

To assess robustness, we estimated additional models including task-related, demographic and weather-related control variables (Methods). When control variables in these models were statistically significant, we additionally tested whether they moderated the effect of political affiliation by including interaction terms. All results are reported in Supplementary Information, section 1.

### Political affiliation in attention allocation and choice

**Fig. 2:**
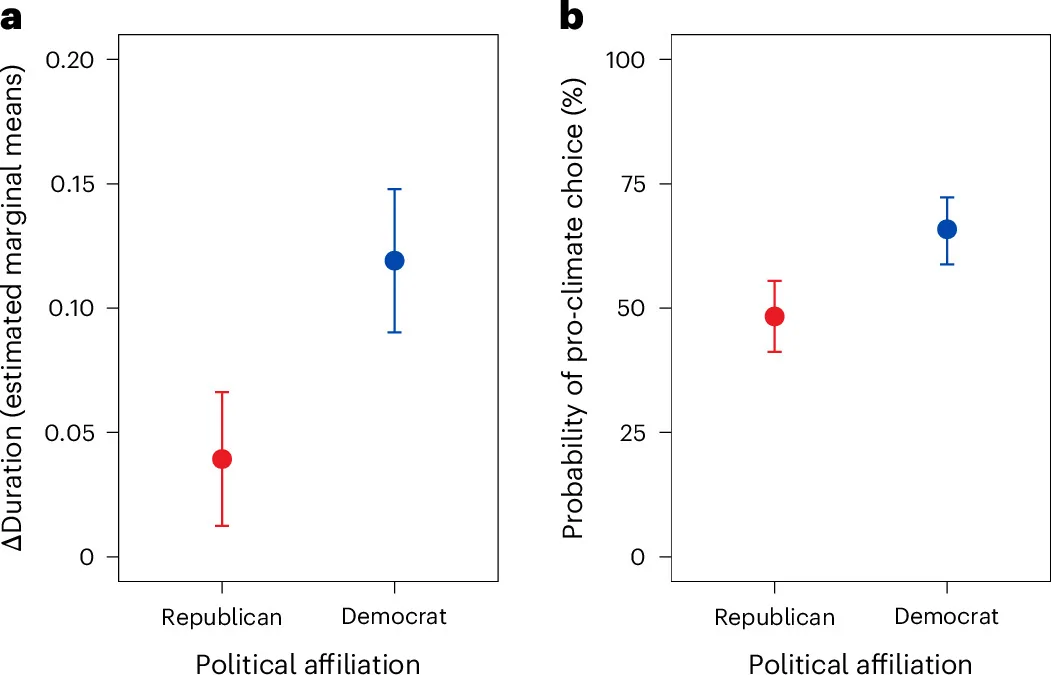
Effects of political affiliation on attention allocation and choice. **a**,**b**, Points represent estimated marginal means for ΔDuration (**a**) and predicted probabilities of pro-climate choice (**b**) by political affiliation (*N* = 1,451 participants). Predictions were derived from a linear mixed-effects model for ΔDuration and a logistic mixed-effects model for choice, including political affiliation as a fixed effect and random intercepts for participants and trials. Error bars represent 95% CIs.

Consistent with our H_1_, political affiliation significantly predicted attention allocation (*b* = 0.08, 95% confidence interval (CI) 0.04–0.12, *P* < 0.001), such that Democrats spent relatively more time attending to carbon information than Republicans (Fig. 2a). This effect remained robust to the inclusion of task-related and demographic controls (Supplementary Table 1), as well as to analyses based on the full sample (Supplementary Table 2). Notably, Democrats and Republicans did not differ in their beliefs about the effectiveness of carbon emission certificates as an emissions reduction mechanism implemented in the task (*M*_Dem_ = 4.13, s.d._Dem_ = 0.85, *M*_Rep_ = 4.10, s.d._Rep_ = 1.02; *P* = 0.496, *t* = −0.68, d.f. = 1446.24, 95% CI −0.13–0.06). The results of the interaction analyses between political affiliation and significant control variables on attention allocation are reported in Supplementary Table 3 and Supplementary Figs. 1 and 2.

In line with our hypothesis H_S1_, political affiliation significantly predicted pro-climate choices (odds ratio (OR) = 2.06, *P* < 0.001; Fig. 2b), and this effect remained significant after adjusting for task characteristics and participant-level controls (Supplementary Table 4).

### Political affiliation and extreme weather events

**Extended Data Fig. 1:**
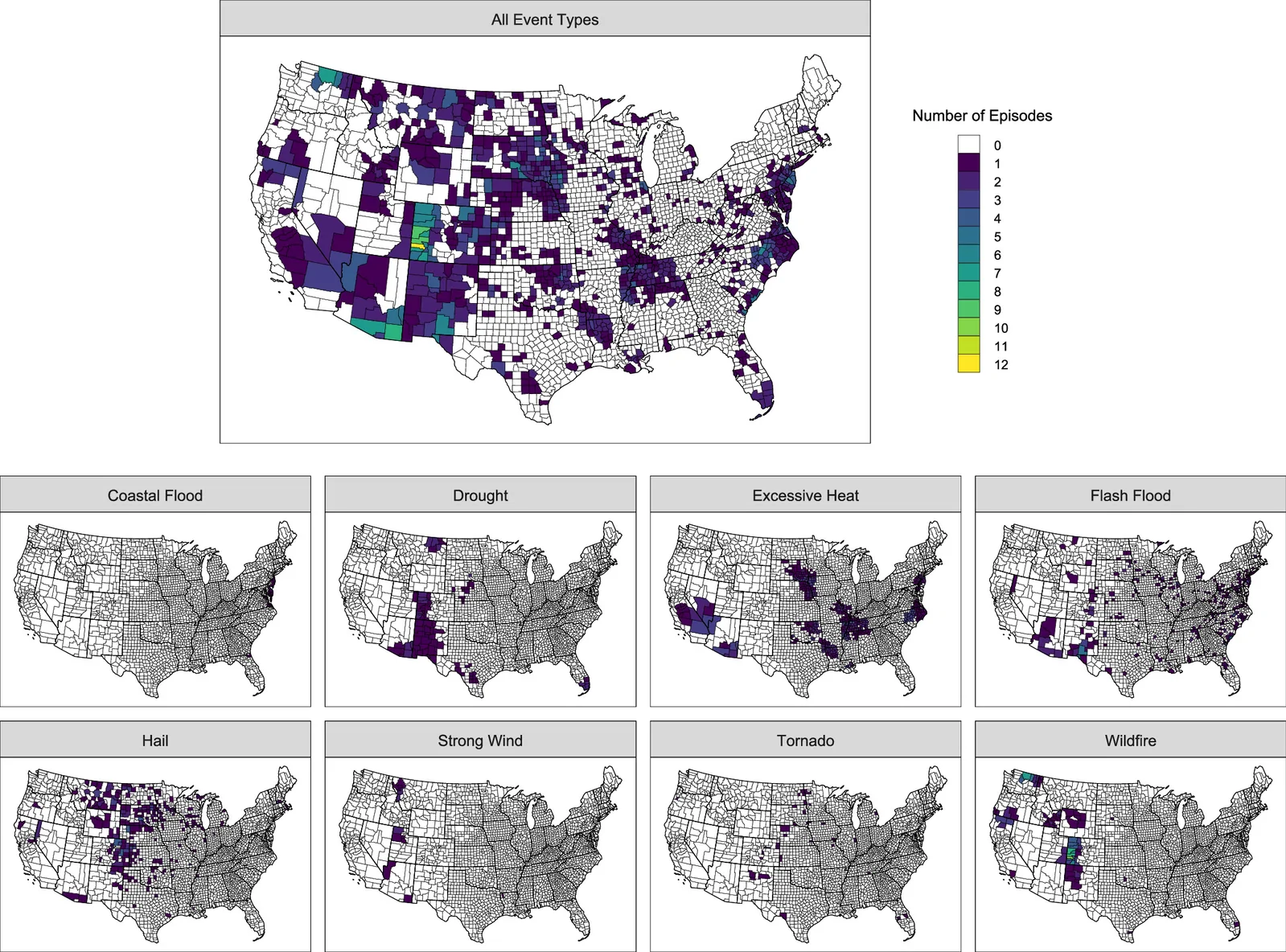
Distribution of extreme weather episodes across U.S. counties (past 30 days). U.S. counties are colored by the number of extreme weather episodes recorded in the past 30 days preceding the midpoint of data collection (≈50% of the sample collected, August 23, 2025). This reference point was chosen because data collection spanned approximately four weeks. Map boundaries are based on the U.S. Census Bureau Cartographic Boundary Files and were visualized using the R package usmap.

Next, we examined the distribution of extreme weather event exposure across the sample. County-level maps (Extended Data Fig. 1) illustrate the occurrence and frequency of different extreme weather event types in the 30 days preceding the midpoint of data collection (see also Supplementary Fig. 3 for a 360-day window). In the final analytic sample, Republicans were slightly more likely than Democrats to have experienced at least one objectively recorded extreme weather event during the study period (48.2% versus 43.4%).

We then tested whether exposure to extreme weather events moderates the relationship between political affiliation and attention allocation (H_2_). For the main effects, political affiliation remained a positive and significant predictor of ΔDuration (*b* = 0.08, 95% CI 0.04–0.12, *P* < 0.001), whereas exposure to at least one extreme weather event was not associated with greater relative attention to carbon information. The interaction between political affiliation and extreme weather exposure was not statistically significant, providing no support for H_2_ that prior exposure amplifies partisan differences in attention allocation to carbon information. This pattern was robust across model specifications, including those adjusting for various controls, using a duration-weighted measure of exposure, and analyses based on the full sample (Supplementary Tables 5 and 6). Details on equivalence tests can be found in Supplementary Fig. 4.

Further sensitivity analyses showed that the interaction between political affiliation and extreme weather exposure on attention allocation was not moderated by county-level characteristics, and remained consistent across event types and exposure windows (1–12 months before study completion). Full results, including equivalence tests, are reported in Supplementary Tables 7–10 and Supplementary Figs. 5–7.

Consistent with the results for attention allocation, the interaction between political affiliation and extreme weather exposure did not significantly predict pro-climate choices (H_S2_; Supplementary Table 11), providing no evidence that actual exposure to extreme weather events moderates partisan differences in pro-environmental decision outcomes.

### Subjective attribution to climate change

We first tested whether the attribution of extreme weather events to climate change varied by political affiliation. Democrats reported higher attribution than Republicans (*M*_Dem_ = 4.52, s.d._Dem_ = 0.65, *M*_Rep_ = 3.94, s.d._Rep_ = 1.16; *P* < 0.001). We then tested main effects and two-way interactions, followed by the three-way interaction between political affiliation, extreme weather exposure and subjective attribution (H_3_).

Subjective attribution was positively associated with attention allocation (*b* = 0.05, 95% CI 0.03–0.07, *P* < 0.001), reflecting greater attention to carbon emissions compared to financial information among participants who more strongly attributed extreme weather events to climate change. This effect held over and above the significant effect of political affiliation (*b* = 0.05, 95% CI 0.01–0.09, *P* = 0.021). In contrast, objective extreme weather exposure was not significantly associated with attention allocation (Supplementary Table 12).

**Fig. 3:**
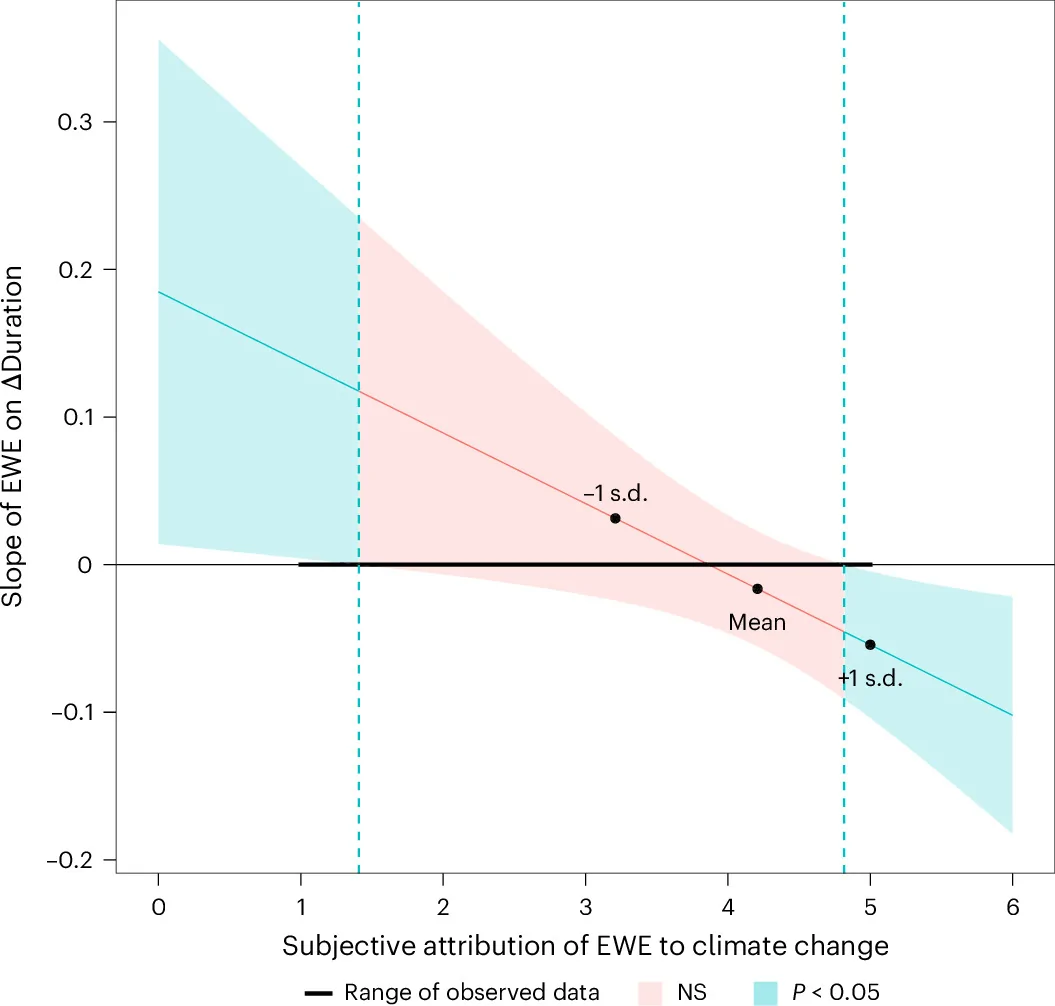
Interaction between extreme weather exposure and subjective attribution of extreme weather to climate change on attention allocation. Johnson–Neyman plot probing the interaction between extreme weather exposure (EWE) and subjective attribution of extreme weather exposure to climate change on ΔDuration (*N* = 1,451 participants). The plotted line represents the conditional effect of political affiliation on ΔDuration across values of subjective attribution. Shaded areas indicate regions where the effect is statistically significant (*P* ≤ 0.05; blue) or non-significant (NS, red), with dashed vertical lines indicating the boundaries between these regions. Points indicate the conditional effects at −1 s.d., the mean and +1 s.d. of subjective attribution. The black horizontal bar represents the observed range of the subjective attribution.

The interaction between political affiliation and subjective attribution of extreme weather events to climate change was positive and statistically significant (*b* = 0.07, 95% CI 0.02–0.12, *P* = 0.006), indicating that the association between political affiliation and attention allocation became stronger as subjective attribution increased. Johnson–Neyman analyses (Supplementary Fig. 8) showed that the effect of political affiliation was statistically significant at low and high levels of subjective attribution, but not across the range of moderate attribution levels. Thus, the strongest partisan differences in attention allocation were observed among participants with higher levels of subjective attribution to climate change (Supplementary Table 12 and Supplementary Fig. 8). The interaction between objective extreme weather exposure and subjective attribution was negative and statistically significant (*b* = −0.05, 95% CI −0.09 to −0.01, *P* = 0.017), indicating that the association between extreme weather exposure and relative attention to carbon information became less positive as subjective attribution increased. Johnson–Neyman analyses (Fig. 3) showed that this association was significantly positive at low levels of subjective attribution, non-significant across moderate levels and significantly negative at high levels of subjective attribution.

The three-way interaction between political affiliation, extreme weather exposure and subjective attribution to climate change (H_3_) was not statistically significant, indicating that the effect of political affiliation on attention allocation did not jointly depend on exposure to extreme weather and subjective attribution. This pattern remained unchanged when including control variables (Supplementary Table 12), as well as when using the full sample (Supplementary Table 13). A marginally significant effect emerged only when using duration-weighted extreme weather events (*b* = 0.01, 95% CI <0.01 to 0.02, *P* = 0.048; Supplementary Table 12 and Supplementary Fig. 10). However, this effect was not observed when using the unweighted exposure measure in sensitivity analyses, including when varying the time window used to measure exposure (1–12 months before study completion; Supplementary Table 15). No county-level variables significantly moderated the three-way interaction (Supplementary Table 14). Further sensitivity analyses based on the unweighted measure indicated some heterogeneity across event types and exposure windows (Supplementary Table 16), with significant three-way interactions limited to specific cases (strong wind events ≥120 days, Supplementary Fig. 13; coastal floods ≥120 days, Supplementary Fig. 14). Equivalence tests for H_3_ are shown in Supplementary Figs. 9, 11 and 12.

Next, we tested whether political affiliation, extreme weather exposure and subjective attribution to climate change interacted in predicting pro-climate choices (H_S3_). Again, we first examined the main effects and two-way interactions before estimating the full three-way interaction (Supplementary Table 17). Political affiliation (OR = 1.68, 95% CI 1.21–2.33, *P* = 0.002) and subjective attribution (OR = 1.41, 95% CI 1.19–1.66, *P* < 0.001) were positively associated with pro-climate choices. In contrast to H_2_, where extreme weather exposure was not a significant predictor of attention allocation, exposure was negatively associated with pro-climate choices when subjective attribution was additionally included in the model (OR = 0.72, 95% CI 0.52–0.98, *P* = 0.036). The interaction between extreme weather exposure and subjective attribution was negative and statistically significant (OR = 0.66, 95% CI 0.47–0.90, *P* = 0.010), indicating that the association between extreme weather exposure and pro-climate choices weakened as subjective attribution increased. By contrast, and in line with the attention hypothesis, the interaction between political affiliation and subjective attribution was positive (OR = 1.64, 95% CI 1.09–2.46, *P* = 0.017), with the difference between Democrats and Republicans increasing for individuals more strongly attributing extreme weather events to climate change.

The three-way interaction between political affiliation, extreme weather exposure and subjective attribution did not significantly predict pro-climate choices, providing no support for H_S3_. This result was robust to additional controls (Supplementary Table 17).

### Subjective experiences of extreme weather events

Next, we explored whether subjective experiences of extreme weather events were associated with attention allocation and decision outcomes. Because subjective exposure was highly right-skewed, one extreme outlier was removed, and the remaining values were log-transformed.

**Fig. 4:**
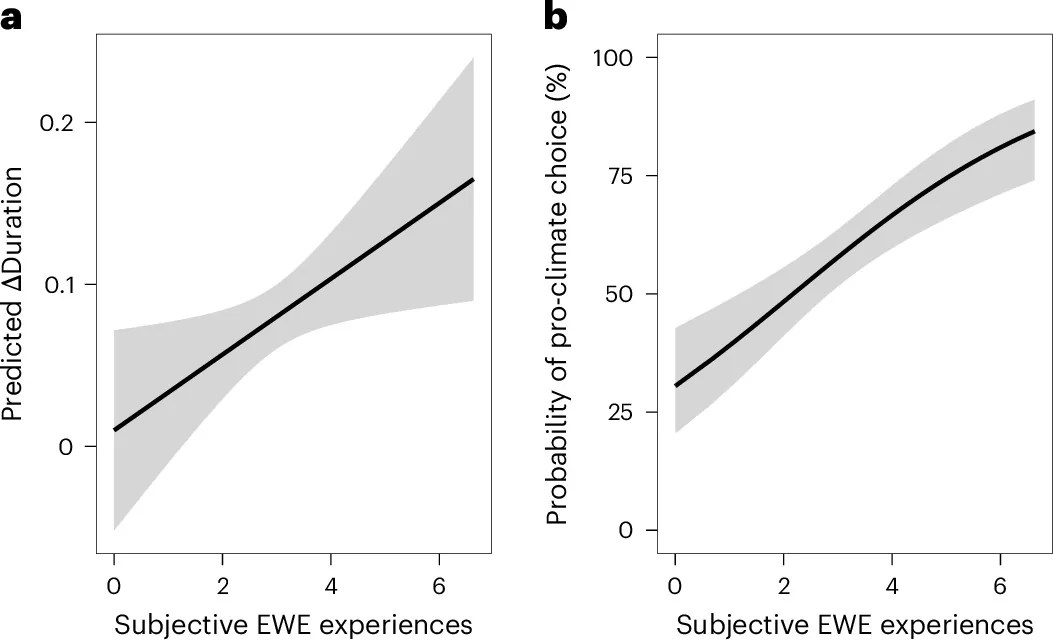
Effects of subjective experiences of extreme weather events on attention allocation and choice. **a**,**b**, Predicted ΔDuration (**a**) and probability of pro-climate choice (**b**) across subjective experiences of extreme weather events (EWE) (*N* = 1,450 participants). Predictions were derived from a linear mixed-effects model for ΔDuration and a logistic mixed-effects model for choice, including subjective experiences and political affiliation as fixed effects and random intercepts for participants and trials. Subjective experiences reflect the summed number of self-reported extreme weather events across event types and were log-transformed for analysis. Predictions were averaged over political affiliation. Shaded areas represent 95% CIs.

Democrats reported higher levels of extreme weather exposure than Republicans (*M*_Dem_ = 3.06, s.d._Dem_ = 0.97, *M*_Rep_ = 2.87, s.d._Rep_ = 1.01, *P* < 0.001), despite lower levels of objective exposure. Subjective experience was positively associated with greater relative attention to carbon information (*b* = 0.02, 95% CI <0.01 to 0.04, *P* = 0.020) and pro-climate choices (OR = 1.46, 95% CI 1.25–1.71, *P* < 0.001) (Fig. 4).

However, subjective experience did not interact with political affiliation or subjective attribution when predicting attention allocation or pro-climate choices. The three-way interaction between political affiliation, subjective extreme weather experience and subjective attribution was not significant (Supplementary Tables 18–21).

## Discussion

We examined how political affiliation, objective exposure to extreme weather events and subjective attribution jointly shape attention to climate-relevant information and pro-climate choices. Our main findings suggest that political differences in climate-related decision-making emerge not only at the point of choice but already during information search, where attention is selectively allocated to environmental and financial information.

Consistent with our H_1_, political affiliation was associated with differences in attention to climate-relevant information. Democrats devoted more attention to carbon emission information (relative to financial attributes) and were more likely to choose the pro-climate option than Republicans. While prior research has primarily documented political differences in climate-related choices^12,14,20,50,51^, our findings suggest that such differences emerge earlier during information acquisition. This pattern is consistent with theories of motivated reasoning and identity-protective cognition, which propose that individuals preferentially attend to information that aligns with their existing beliefs and group identities^29,52–54^. It also accords with previous work showing that political identity shapes attention to information^30,55–57^, and that individuals with stronger pro-environmental commitments prioritize environmental attributes during decision-making^58^.

Contrary to our H_2_, objectively measured exposure to extreme weather events did not moderate the relationship between political affiliation and either attention allocation or pro-climate choices. This finding is consistent with prior work reporting mixed evidence on the effects of extreme weather exposure on climate-related beliefs and behaviours, with effects often depending on moderating factors^4,6,9,59^. This suggests that objective exposure alone is insufficient to shape attention allocation and climate-relevant decision-making, and may depend on how such events are subjectively interpreted.

Contrary to our H_3_, we found little support for a three-way interaction between political affiliation, objective exposure and subjective attribution. However, subjective attribution itself was positively associated with both increased attention to carbon information and pro-climate choices, over and above the effect of political affiliation. This finding underscores the role of beliefs in guiding attention and decision-making, consistent with accounts of motivated reasoning and identity-based information processing^30,52,55^.

We further observed a significant interaction between political affiliation and subjective attribution. Partisan differences in attention allocation and pro-climate choices emerged primarily among individuals who strongly attributed extreme weather events to climate change, suggesting that political differences in information processing depend on whether such events are interpreted in climate-relevant terms^55,56,58^. Notably, we also observed a significant interaction between extreme weather exposure and subjective attribution for both attention allocation and pro-climate choices. Exposure was positively associated with attention to carbon information at low levels of attribution but negatively associated at high levels. This pattern suggests that the effects of exposure depend on how individuals interpret extreme weather events. Among individuals with stronger attribution, the absence of a positive association may reflect a saturation of attention to climate-relevant information, whereas among those with weaker attribution, exposure may act as a salient cue that increases engagement with climate-relevant attributes. Alternatively, individuals who strongly attribute extreme weather events to climate change may reduce attention to threatening information through avoidance or defensive processing^60–62^.

Subjective attribution did not systematically strengthen the joint effect of political affiliation and exposure on attention allocation. Although some indication of such a pattern emerged when exposure was operationalized as cumulative intensity, this effect did not generalize across exposure measures—in line with research showing that effects of extreme weather exposure are heterogeneous and depend on event type and how exposure is operationalized^59^. One possible explanation is that some events are more likely than others to be perceived as climate-relevant, thereby activating processes of attribution and interpretation.

In exploratory analyses, subjective experience of extreme weather predicted both attention allocation and pro-climate choices but did not interact with subjective attribution, political affiliation or in the expected three-way interaction. Notably, Democrats reported more frequent extreme weather experiences despite lower levels of objective exposure. This further supports the idea that exposure is shaped not only by environmental conditions but also by how such events are perceived and interpreted^8,59^. Individuals who are more likely to attribute extreme weather events to climate change may also be more likely to notice, recall or interpret such events as climate-relevant experiences^8,40,63^. Accordingly, political affiliation may function as a perceptual filter that shapes how environmental experiences are encoded, thereby influencing attention allocation and climate-relevant decision-making.

One limitation of the present study is the proportion of participants excluded under the preregistered quality criteria. Although these exclusions ensured high-quality process-tracing data, they removed approximately one quarter of the initial sample, potentially limiting generalizability. However, sensitivity analyses on the full sample yielded substantively similar conclusions, indicating that the findings were robust to the exclusion procedure.

Taken together, our findings highlight the importance of a multi-level perspective on climate-relevant behaviour by showing that cognitive processes, individual characteristics and environmental determinants jointly shape attention allocation and decision-making. Political affiliation was consistently associated with differences in attention allocation and pro-climate choices, whereas the effects of extreme weather exposure depended on subjective attribution. From this perspective, attention allocation reflects the interplay between top-down influences (for example, political identity and beliefs) and bottom-up inputs (for example, exposure to extreme weather). Environmental experiences thus appear to shape climate-relevant decision-making insofar as they are cognitively encoded and interpreted in light of existing belief systems, consistent with accounts of motivated reasoning and identity-protective cognition^29,52,54^. From an applied perspective, increasing exposure to extreme weather events alone is unlikely to foster greater climate-relevant attention or pro-climate choices. Rather, our findings highlight the importance of communication strategies that help people contextualize extreme weather events in relation to climate change.

## Methods

### Design

We conducted a large-scale, cross-sectional online study using a representative US sample coupling a decision-making experiment and survey assessment with data on participants’ objective exposure to extreme weather.

#### Procedure

Participants completed questionnaires in Qualtrics and decision-making tasks in MouselabWEB. After passing preregistered quality checks, participants completed the CET, after which they received feedback regarding the randomly selected incentivized trial. Participants received US$0.95 plus a performance-contingent bonus ranging from US$0.30 to US$0.96. More details on the exact sequencing can be found in Supplementary Information, section 2.1.

#### Device and attention checks, questionnaires and demographics

Participation was restricted to desktop or laptop devices and completed a CAPTCHA verification procedure before entering the study. More details on the survey structure and questions can be found in Supplementary Table 22.

After providing informed consent, participants completed three preregistered attention checks: a consistency check between reported age and year of birth, a comprehension question about the study topic and an instructed-response item requiring selection of “somewhat disagree” on a 5-point scale. Participants who failed any check were redirected to the panel provider, whereas those who passed proceeded to the decision-making experiment.

Following completion of the CET, participants reported the device and navigation method used, any technical difficulties encountered and their beliefs regarding the effectiveness of carbon emission certificates with the following statement (5-point Likert scale ranging from “strongly disagree” to “strongly agree”): “Buying and retiring carbon emission certificates is an effective means to reduce carbon emissions.”

For exploratory analyses, participants reported their subjective experiences with extreme weather events. They were presented with the 45 event types recorded in the Storm Events Database and indicated which they had experienced during the previous 120 days. Definitions of all event types, based on the Storm Events Database documentation, were available via https://decision-making.unibe.ch/EcoTRACE/infoEWE.html. For each endorsed event type, participants reported its frequency and how strongly it had affected them on a 5-point scale ranging from “affected me not at all” to “affected me strongly.”

To capture direct and indirect experiences with extreme weather events, participants completed additional exploratory measures adapted from previous research^47^. Direct experiences included impacts on personal property, disruptions to work or travel, interruptions to essential services and property damage experienced by others in the local area. Indirect experiences included media exposure to extreme weather events occurring locally or elsewhere in the USA and reports of people close to them (for example, family, friends or colleagues) being affected by such events during the preceding 120 days.

The subjective attribution of extreme weather events to climate change was operationalized as the mean agreement across three items assessing the extent to which participants viewed extreme weather events as caused by, indicative of or representative of the impacts of climate change. Climate change concern was additionally assessed using a single-item measure. Full item wording and response scales are reported in Supplementary Table 22.

Next, participants completed a standard 7-point measure of partisan identification, followed by an additional forced-choice item for respondents identifying as politically independent. Following our preregistration, participants were classified as Democrats or Republicans according to their responses, while retaining the original scale for exploratory analyses. In the last block, we assessed the following demographic information from the participants: gender, highest level of education, household income, cultural background (race or ethnicity) and area of residency (urban or big city, suburb or outskirts of a big city, rural town or small city). Finally, we assessed participants’ state and county of residence using Federal Information Processing Standard (FIPS) codes to match participants with data from the Storm Events Database. In case participants did not know their FIPS code, we provided them with a link to a website that returns FIPS codes based on street addresses (https://www.smarty.com/products/single-address) and a short video clip on how to use the website.

#### New variant of CET

To assess information search processes during decision-making with real consequences, we designed a new variant of the validated CET^12^. The CET is a behavioural paradigm for studying decision-making processes in scenarios where individuals must balance economic benefits against environmental consequences, specifically carbon emissions. Across 30 trials, participants chose between two options (A and B) that differed in the amount of bonus payment and carbon emissions they led to. Participants could inspect the exact bonus and carbon consequences of each option by moving their mouse over boxes, revealing this information. Figure 1a,b presents a sample trial, while Fig. 1c displays all available information revealed for demonstration purposes. A working version of the task, with all of the instructions and explanations for participants, can be accessed through the following link: https://decision-making.unibe.ch/EcoTRACE/.

Participants completed three practice trials and three comprehension questions before the task. They were familiarized with carbon emission metrics using examples based on US Environmental Protection Agency estimates^64^. One randomly selected choice was implemented to incentivize consequential decision-making^65,66^. Bonus payments were distributed via the panel provider, and carbon emissions were realized through the retirement of emission certificates in collaboration with a non-governmental organization (https://www.compensators.org/en/compensators/), creating real environmental consequences for participants’ decisions^20,26^.

Across 25 core trials, the average bonus level of Option A and Option B was set to US$0.60, and the average carbon emission level was set to 19.85 lb CO_2_. Importantly, we varied the relative differences between options’ bonus and carbon consequences across trials to assess price- and cost-sensitivity, consistent with prior research^20,67^.To maximize sensitivity to experimental manipulations and individual differences in information search behaviour, relative difference levels were chosen to over-represent small differences (10–15%)^68,69^. Specifically, we fully crossed five difference levels for bonus payments and carbon emissions (10%, 15%, 20%, 50% and 100%). Core trials were designed such that lower bonus payments were paired with lower carbon emissions, reflecting everyday choices, where more sustainable options often frequently come with higher costs—whether monetary, temporal or in terms of effort—forcing individuals to weigh competing priorities. To reduce the possibility that participants could infer unattended attribute values through learning, random deviations were added to bonus and carbon levels in each trial (bonus: *M* = 0, s.d. = US$0.03; carbon: *M* = 0, s.d. = 0.9925 lb CO_2_). Five inversion trials were included to sustain attention and reduce predictability. In these trials, selected from different carbon-difference levels, carbon emissions were swapped between options so that the higher-bonus (‘pro-self’) option was associated with lower emissions. These trials were excluded from analyses. Supplementary Table 23 provides an overview of all core trials and Supplementary Table 24 of all inversion trials.

Finally, three randomization procedures controlled for presentation effects: (1) the position of carbon versus bonus information (top or bottom) was randomized between participants, (2) the location of the higher-bonus option (left or right) was randomized within participants across trials and (3) the trial order was randomized across participants.

#### Process-tracing with MouselabWEB

Information search during decision-making in the new variant of the CET was recorded using MouselabWEB^21^, a process-tracing method that tracks mouse movements to evaluate how participants gather information about options. In MouselabWEB, each piece of information was initially hidden (Fig. 1a). Participants revealed information by moving the mouse over a specific box, which displayed the content (Fig. 1b). When the mouse moved away, the information was hidden again. Thus, mouse movements tracked information search and acquisition. MouselabWEB data are comparable to other process-tracing methods like eye tracking^21,68,70^. For each trial, we computed ΔDuration as follows, with *t* representing the dwell time on a specific attribute (subscript Carbon or Bonus) of options (subscript A or B):

$$\Delta{\mathrm{Duration}}=\frac{({t}_{{\mathrm{Carbon}}_{{\rm{A}}}}+{t}_{{\mathrm{Carbon}}_{{\rm{B}}}})-({t}_{{\mathrm{Bonus}}_{{\rm{A}}}}+{t}_{{\mathrm{Bonus}}_{{\rm{B}}}})}{{t}_{{\mathrm{Carbon}}_{{\rm{A}}}}+{t}_{{\mathrm{Carbon}}_{{\rm{B}}}}+{t}_{{\mathrm{Bonus}}_{{\rm{A}}}}+{t}_{{\mathrm{Bonus}}_{{\rm{B}}}}}$$

ΔDuration indexes the relative dwell time on carbon versus bonus information, normalized by total information acquisition time. Values range from −1 (only bonus information) to +1 (only carbon information), with 0 indicating equal attention to both. Additional exploratory process-tracing measures are reported in Supplementary Information, section 2.4.

Finally, we also assessed the actual decision—that is, the choice participants made in each trial:

$$\mathrm{Choice}=\left\{\begin{array}{cc}0, & \mathrm{pro}-\mathrm{self}\\ 1, & \mathrm{pro}-\mathrm{climate}\end{array}\right.$$

#### Extreme weather exposure

We measured extreme weather exposure using data from the National Oceanic and Atmospheric Administration’s Storm Events Database, maintained by the National Weather Service. Details about this database can be found in Supplementary Information, section 2.5.1. For our analyses, we focused on the types of extreme weather events predicted to increase in frequency and severity due to climate change^1^: excessive heat, droughts, wildfires, flash floods, coastal floods, hail, strong wind and tornados. In previous research, all but flash floods were associated with increases in social media messages about climate change in the local area around the time of each event, indicating that these event types are capable of increasing public (online) attention to climate change^27^. Geographical identifiers from the database, translated to FIPS codes, allowed us to map county-level extreme weather data to participants’ counties of residence, effectively assessing participants’ exposure to extreme weather episodes. County-level exposure provides a meaningful proxy for individual exposure because many extreme weather events affect broad geographic areas and are, therefore, likely to be experienced directly by residents. In addition, indirect exposure through local news and social media may also influence attention to climate-relevant information.

Given the evidence that the effects of extreme weather experiences on climate-related attitudes are strongest for recent events and decline over time^4,5,71^, we focused on exposure to extreme weather events occurring within the 30 days before study completion^28^. Exposure was operationalized as whether participants experienced at least one episode involving excessive heat, drought, wildfire, flash floods, coastal floods, hail, strong wind or tornadoes in their county of residence during this period (binary variable). Additional analyses examined exposure windows ranging from 30 to 360 days in 30-day increments (‘Analysis plan’).

We assessed several variables to account for between-county heterogeneity in both exposure to extreme weather episodes and broader economic and political factors in our sensitivity analyses. Specifically, we considered: (1) the abnormality score for the number of extreme weather episodes, (2) the severity-weighted count of episodes, (3) the amount of financial damage and number of injuries and deaths associated with each episode, (4) the number of days since the most recent extreme weather episode before study completion, (5) the population density of each county, (6) the county-level median household income estimates (2023), sourced from the US Census Bureau’s Small Area Income and Poverty Estimates, and (7) the county’s Partisan Voting Index, calculated based on presidential election results from 2010 to 2020 using data from the Massachusetts Institute of Technology Election Data and Science Lab’s County Presidential Election Returns 2000–2020 dataset. More details about the rationale and formula of the abnormality score as well as alternative indicators of severity can be found in Supplementary Information, section 2.5.2. Risk management of extreme weather events can be found in Supplementary Information, section 2.5.3.

### Analysis plan

The complete preregistered analysis plan, including all model specifications, robustness checks, sensitivity analyses, exploratory analyses and interpretation criteria, is provided in Supplementary Table 26.

#### General analytical approach

All models were estimated as mixed-effects models with random intercepts for participants (‘subject’) and trials (‘trialNum_fixed’) to account for the repeated observations nested within participants and trial-specific variation. This specification accounts for the non-independence of repeated observations while allowing baseline levels of the outcomes to vary across participants and trials. Information search processes were analysed with ΔDuration (‘deltaDuration’) as the main dependent variable, the primary predictors were political affiliation (‘polAff’; 0 = Republican, 1 = Democrat), recent extreme weather exposure (‘ewe’; 0 = no episode, 1 = at least one extreme weather episode in the participant’s county during the 30 days before study completion) and subjective attribution of extreme weather events to climate change (‘subjAttr’; mean-centred continuous variable). Pro-climate decisions (‘choice’; 0 = pro-self, 1 = pro-climate) were analysed using generalized linear mixed-effects models with a binomial link. Unless otherwise noted, robustness analyses additionally adjusted for preregistered task-, demographic-, exposure- and county-level covariates (Supplementary Table 26). Because interaction terms complicate the interpretation of variance inflation factors, multicollinearity was assessed in the corresponding main-effects models only. Variance inflation factors ranged from 1.01 to 1.10, indicating negligible multicollinearity (Supplementary Information, section 1.3.3). All analyses were conducted in R (version 4.5.2) using the packages lme4^72^, tidyverse^73^ and easystats^74^.

#### Political affiliation hypothesis (H_1_ and H_S1_)

To test whether Democrats displayed higher ΔDuration values than Republicans (H_1_), we estimated model M1.1:

1 $$\Delta{\mathrm{Duration}}\sim{\;\mathrm{polAff}}+(1|{\mathrm{subject}})+(1|{\mathrm{trialNum}\_{\mathrm{fixed}}})$$

We subsequently evaluated the robustness of this effect by adding preregistered task-related (M1.2) and demographic (M1.3) covariates. If any control variables significantly predicted ΔDuration, we additionally tested whether they moderated the effect of political affiliation by including interaction terms. Significant interactions were probed by estimating simple effects at the relevant levels of the moderator.

To test whether Democrats were more likely than Republicans to make pro-climate choices (H_S1_), we estimated the corresponding generalized linear mixed-effects models using ‘choice’ as the dependent variable.

#### Extreme weather hypothesis (H_2_ and H_S2_)

To test whether recent extreme weather exposure moderated the relationship between political affiliation and information search processes (H_2_), we first estimated the main effects of political affiliation and extreme weather exposure (M2.1), followed by the preregistered interaction model (M2.2):

2 $$\Delta{\mathrm{Duration}}\sim{\;\mathrm{polAff}}*{\mathrm{ewe}}+(1|{\mathrm{subject}})+(1|{\mathrm{trialNum}\_{\mathrm{fixed}}})$$

Note that in R formula notation, polAff * ewe refers to polAff + ewe + polAff:ewe, that is, specifying an interaction with * automatically includes the corresponding lower-order terms (including the two-way interactions in a model with a three-way interaction). Robustness analyses evaluated the sensitivity of this interaction to the inclusion of significant control variables identified under H_1_ (M2.3), preregistered exposure- and county-level covariates (M2.4), and an alternative duration-weighted measure of extreme weather exposure (M2.5). Complete model specifications are provided in Supplementary Table 26.

As a robustness check, M2.2 (equation (2)) was additionally estimated using the full sample. If the interaction was not statistically significant, we evaluated practical equivalence using the preregistered smallest effect size of interest (*β* = 0.1143). Following ref. ^75^, interaction effects whose (1 − 2α) CIs fell entirely within the equivalence bounds were interpreted as practically equivalent to zero.

To explore contextual heterogeneity, we conducted exploratory moderation analyses examining whether county-level population density, median household income or Partisan Voting Index moderated the interaction between political affiliation and extreme weather exposure.

Additional sensitivity analyses examined whether results depended on the exposure window by estimating interaction effects across time windows ranging from 30 to 360 days before study completion in 30-day increments. Following previous work, these analyses operationalized exposure as the number of extreme weather episodes and repeated the analyses separately for each extreme weather event type^28^.

To test whether extreme weather exposure moderated pro-climate choices (H_S2_), we estimated the corresponding mixed-effects logistic regression models using choice as the dependent variable.

#### Subjective attribution hypothesis (H_3_ and H_S3_)

To test whether the subjective attribution of extreme weather events to climate change moderated the interaction between political affiliation and extreme weather exposure (H_3_), we first estimated models including the main effects (M3.1), followed by separate models testing the two-way interactions between political affiliation and subjective attribution (M3.2), and between extreme weather exposure and subjective attribution (M3.3). We then estimated a model including all two-way interactions (M3.4), before testing the preregistered three-way interaction (M3.5):

3 $$\Delta{\mathrm{Duration}}\sim{\;\mathrm{polAff}}*{\mathrm{ewe}}*{\mathrm{subjAttr}}+(1|{\mathrm{subject}})+(1|{\mathrm{trialNum}\_{\mathrm{fixed}}})$$

Significant interactions were probed by estimating simple effects at low (−1 s.d.), average and high (+1 s.d.) levels of the moderator. Models M3.2, M3.3 and M3.5 were additionally estimated using the full sample. Non-significant interaction effects were evaluated using equivalence testing against the preregistered smallest effect size of interest. For the three-way interaction (M3.5; equation (3)), the smallest effect size of interest was *β* = 0.1345; interaction effects whose (1 − 2α) CIs fell entirely within the equivalence bounds were interpreted as evidence that subjective attribution did not meaningfully moderate the relationship between political affiliation, extreme weather exposure and information search processes^76^.

Robustness analyses further examined the sensitivity of M3.5 (equation (3)) to significant H_1_ control variables (M3.6), preregistered exposure- and county-level covariates (M3.7), and a duration-weighted measure of extreme weather exposure (M3.8). Exploratory analyses additionally examined whether county-level population density, median household income and Partisan Voting Index moderated the three-way interaction. Additional sensitivity analyses estimated the three-way interaction across exposure windows ranging from 1 to 12 standard months and repeated these analyses separately for each extreme weather event type^28^.

To test whether political affiliation, extreme weather exposure and subjective attribution jointly predicted pro-climate choices (H_S3_), we estimated the corresponding mixed-effects logistic regression models using ‘choice’ as the dependent variable.

#### Implementation

Implementation was largely consistent with the preregistered analysis plan, with a minor deviation related to the operationalization of one control variable. In the preregistered analysis plan, we intended to control for the recency of extreme weather exposure using a continuous variable capturing the number of days since the most recent extreme weather episode (for H_2_, model M2.4, and for H_3_, model M3.7). However, this operationalization resulted in missing values for participants who had not experienced any extreme weather events during the observation period. Including this variable in regression models would, therefore, have led to the exclusion of all participants who had not experienced any extreme weather events, making it impossible to estimate the effects of extreme weather exposure.

To address this issue while retaining all observations, we replaced this measure with a recency index defined as 1/days since most recent episode + 1. This transformation captures the temporal proximity of the most recent extreme weather episode while allowing participants without any recorded events to be assigned a value of zero. Higher values, therefore, indicate more recent exposure, whereas a value of zero indicates no exposure within the observation window. This modification allowed us to retain the full sample while preserving the intended interpretation of the recency control.

### Sampling plan

We conducted the study using the market research agency MSI-ACI’s FlexPanel, targeting 2,000 respondents (for details of the power analyses, see Supplementary Information, section 2.7). To ensure it represents the US adult population, quotas were managed across several key dimensions: political affiliation, age, gender and region. Given that climate beliefs and policy support can vary across age groups^11,77^, age and gender were stratified into specific groups based on census data: 18–25, 26–35, 36–45, 46–55, 56–65, 66–74 and 75–85, for both men and women.

#### Exclusion criteria

We employed several exclusion criteria at the participant and trial levels to ensure high data quality. Due to oversampling by the survey provider to ensure quota fulfilment and valid responses, the initial dataset contained *N* = 2,819 participants, of whom *n* = 672 were automatically screened out if they (1) failed the CAPTCHA or (2) did not give informed consent, (3) failed any of the attention checks described in Methods or (4) did not finish the study.

After these initial screen-outs, we excluded participants for whom no valid FIPS code was available (*n* = 58) as well as those residing in Alaska or Hawaii (*n* = 16), resulting in an analytic sample of *n* = 2,073 participants. To obtain the preregistered target sample size (*n* = 2,000), we randomly downsampled the dataset while preserving the planned quota structure across region, age group, gender and political affiliation. Participants were randomly selected within quota cells up to the target counts, and remaining slots were filled by sampling from eligible respondents. The final sample was then adjusted to approximate the targeted 50:50 balance between Democratic and Republican respondents as closely as possible given the available respondents. The final distribution of political affiliation was 50.2% Democrats and 49.8% Republicans. The largest deviation from the target quotas was 4.00% for the West region. Supplementary Table 30 provides an overview of the final sample distribution across all quotas.

Among the remaining *n* = 2,000 participants, we applied exclusion criteria based on process-tracing metrics in the CET. One participant was excluded because no process-tracing data were recorded for the CET. For the remaining *n* = 1,999 participants, we followed standard practice in MouselabWEB studies and excluded box openings within each trial that were shorter than 200 ms, as such brief information acquisitions are unlikely to be consciously processed^21^. Next, we calculated for each trial within each participant the total time that was spent on opening boxes—that is, the time spent on searching for and attending to task-relevant information. In a first step, we used this trial-level total box opening time as a metric to flag trials that could be considered as participant-specific outliers. That is, we identified trials with exceptionally short (indicating low engagement or accidental choice) or long box opening times (indicating task-unrelated distractions or interruptions) with respect to the univariate distribution of box opening times for each participant. We applied the following five methods of outlier detection using the performance package in R^74^. (1) According to the 1.5-times-interquartile range method (corresponding to outliers in typical box plots), a trial is considered an outlier if its total box opening time lies outside 1.5 times the interquartile range. (2) Following the *Z*-score method, a trial is considered an outlier if its total box opening time is more extreme than the mean ± 1.96 s.d. (3) The robust-*Z*-score method is applied by proceeding as in (2), but replacing the mean by the median and s.d. by the median absolute deviation. (4) The 95% CI method defines trials as outliers if they lie outside the 95% CI. (5) Finally, applying the same steps as in (4) but replacing CIs with highest density intervals defines outliers according to the highest density interval method. We leveraged and integrated the unique advantages of each of these methods by defining a trial as a participant-specific outlier if it is flagged as such according to at least three out of the five methods described.

In a second step, we applied the same outlier definition at the sample level rather than the participant level. To this end, we calculated the median box opening time of each participant and identified participants with exceptionally short or long box opening times. We defined participants as outliers relative to the whole sample if they are flagged as such according to at least three out of five of the methods described.

We used these definitions of trial- and participant-level outliers for exclusions of data points for further analyses. First, we excluded individual trials flagged as participant-specific outliers. Next, we excluded participants flagged as outliers relative to the whole sample (*n* = 94). Additionally, we excluded participants who had more than five missing trials (indicating potential technical errors in presenting or saving trials, *n* = 52) or had more than five trials in which they did not open any box (indicating low engagement, *n* = 408). Moreover, we excluded participants who after application of the previous exclusion criteria had less than 20 trials in the CET task (that is, participants whose sum of missing, no-box-opening and excluded trials exceeds five trials, *n* = 1). Mobile phone usage, as an additional exclusion criterion, was not applicable to any of our participants.

In summary, in addition to the participant for whom no MouselabWEB data were recorded, 548 participants were excluded because they met at least one of these criteria (with some participants meeting multiple criteria), resulting in a final analytic sample of *n* = 1,451. Supplementary Table 31 summarizes the sociodemographic characteristics of this final sample.

### Ethics approval and consent to participate

The study was approved by the Ethics Commission of the Faculty of Human Sciences of the University of Bern (approval number 2024-09-02), conducted in accordance with the Declaration of Helsinki. All participants provided informed consent.

### Protocol registration

The Stage 1 protocol, as accepted by the journal on 14 July 2025, can be found at ref. ^78^.

### Reporting summary

Further information on research design is available in the Nature Portfolio Reporting Summary linked to this article.

## Online content

Any methods, additional references, Nature Portfolio reporting summaries, source data, extended data, supplementary information, acknowledgements, peer review information; details of author contributions and competing interests; and statements of data and code availability are available at https://doi.org/10.1038/s41558-026-02765-z.

## Supplementary information

Supplementary Information Supplementary text, Figs. 1–32 and Tables 1–31.

Reporting Summary

## Data Availability

All data and materials are available via *Zenodo* at https://doi.org/10.5281/zenodo.21901131 (ref. ^79^).

## Code availability

All analysis code is available via *Zenodo* at https://doi.org/10.5281/zenodo.21901131 (ref. ^79^). An accompanying website (https://juliebuchs.github.io/EcoTRACE/) presents the Supplementary Information together with the corresponding analysis code in a user-friendly format, linking each reported result directly to the underlying code and facilitating navigation through the analysis workflow.
