# Supplementary material for: Political affiliation impacts climate attention and choice beyond extreme weather exposure
Source: Nat Clim Chang. 2026 Oct 16;16(11):1332–8. doi: 10.1038/s41558-026-02765-z (PMC13731144; doi:10.1038/s41558-026-02765-z)
Supplement: Supplementary file 1 [file 41558_2026_2765_MOESM1_ESM.pdf]

nature climate change

Supplementary information https://doi.org/10.1038/s41558-026-02765-z

Political affiliation impacts climate attention and choice beyond extreme weather exposure

In the format provided by the authors and unedited

Table of contents

1. Results 3
   1.1. Political-Affiliation-Hypothesis (H_1 and H_{S1}) 3
      1.1.1. Effect of Political Affiliation on ΔDuration (H_1) 3
      1.1.2. Effect of Political Affiliation on Choice (H_{S1}) 11
   1.2. Political Affiliation and Extreme Weather Events (H_2) 14
      1.2.1. Extreme Weather Events as a Moderator of the Relationship Between Political Affiliation and ΔDuration (H_2) 15
      1.2.2. Extreme Weather Events as a Moderator of the Relationship Between Political Affiliation and Choice (H_{S2}) 56
   1.3. Subjective-Attribution-Hypothesis (H_3 and H_{S3}) 59
      1.3.1. Subjective Attribution to Climate Change and ΔDuration (H_3) 59
      1.3.2. Subjective Attribution to Climate Change and Choice (H_{S3}) 107
      1.3.3. Multicollinearity tests 114
   1.4. Subjective Experiences of Extreme Weather Events 115
2. Methods 127
   2.1. Procedure 127
   2.2. Survey Structure and Questions 128
   2.3. Carbon Emission Task 131
      2.3.1. Carbon Emission Task: Everyday Examples 131
      2.3.2. Carbon Emission Task: Trials 131
   2.4. Process-tracing with MouselabWEB 133
   2.5. Extreme Weather Data 134
      2.5.1. Details About NOAA's Storm Events Database 134
      2.5.2. Extreme Weather Episode Variables for use in Sensitivity Analyses 135
      2.5.3. Risk Management 136
   2.6. Design Table 142
   2.7. Power Analyses 150
      2.7.1. Purpose & Rationale 150
      2.7.2. Important Variables 150
      2.7.3. SESOI 153
      2.7.4. Main Effect 154
      2.7.5. Two-Way Interaction Effect 159

2.7.6. Three-Way Interaction Effect 167
2.7.7. Conclusion 175
2.8. Sample Characteristics 176
2.9. References 179

1. Results

1.1. Political-Affiliation-Hypothesis (H_1 and H_{S1})

1.1.1. Effect of Political Affiliation on ΔDuration (H_1)

We examined whether Democrats display more attention to carbon information (i.e., higher ΔDuration values) compared to Republicans. To this end, we used mixed-effects models with random intercepts for participants and trials. Model 1 included political affiliation only. Model 2 additionally controlled for task characteristics (attribute differences, trial position, and trial layout). Model 3 further included participant-level controls (gender, age, education, income, ethnicity, and area of residence).

Consistent with our hypothesis, political affiliation significantly predicted ΔDuration values in Model 1 (b = 0.08, p < .001), Model 2 (b = 0.08, p < .001), and Model 3 (b = 0.07, p < .001), indicating that Democrats exhibited higher ΔDuration values than Republicans across specifications.

Among the task-related control variables in Model 2, trial position showed a small but significant effect (b < 0.01, p < .001), indicating that as participants progressed through the task they increasingly allocated visual attention to the carbon emission attribute relative to the financial attribute. Furthermore, the position of the carbon attribute was significantly associated with ΔDuration (b = −0.05, p = .022), indicating that when the carbon attribute appeared in the top row, participants allocated relatively less visual attention to carbon emissions compared to the financial attribute. In Model 3, ΔDuration was significantly associated with gender (male: b = −0.05, p = .010), age (b < 0.01, p < .001) and race/ethnicity, with Asian participants showing higher values relative to the overall mean (b = 0.10, p = .043). The negative coefficient for gender indicates that male participants allocated relatively less visual attention to carbon emissions compared to the financial attribute, whereas older participants devoted relatively more attention to carbon emissions. All regression results are reported in Table S1.

To assess the robustness of the results, we re-estimated Model 1 using the full sample (i.e., all participants with at least one valid ΔDuration observation across trials; N = 1,936; participants without valid observations had either no box openings or only openings shorter than 200 msec). The effect of political affiliation remained statistically significant (b = 0.06, p < .001; see Table S2).

	M1.1			M1.2		
Predictors	*Estimates*	*CI*	*p*	*Estimates*	*CI*	*p*
(Intercept)	0.04	0.01 – 0.07	**0.004**	0.05	0.01 – 0.08	**0.015**
polAff bin str [Dem]	0.08	0.04 – 0.12	**<0.001**	0.08	0.04 – 0.12	**<0.001**
carbon prcnt				-0.00	-0.00 – 0.00	0.113
bonus prcnt				0.00	-0.00 – 0.00	0.234
trialNum				0.00	0.00 – 0.00	**<0.001**
CB carbonUp				-0.05	-0.08 – -0.01	**0.021**
CB selfishLeft				-0.01	-0.02 – 0.00	0.068
**Random Effects**						
σ^2	0.29			0.29		
τ_{00}	0.13 $_{subject}$			0.13 $_{subject}$		
	0.00 $_{trialNum\_fixed}$			0.00 $_{trialNum\_fixed}$		
ICC	0.31			0.31		
N	1451 $_{subject}$			1451 $_{subject}$		
	25 $_{trialNum\_fixed}$			25 $_{trialNum\_fixed}$		
Observations	34294			34294		
Marginal R^2 / Conditional R^2	0.004 / 0.316			0.006 / 0.316		

	M1.3		
Predictors	*Estimates*	*CI*	*p*
(Intercept)	-0.01	-0.12 – 0.09	0.836
polAff bin str [Dem]	0.07	0.03 – 0.11	**0.001**
gender [Male]	-0.05	-0.09 – -0.01	**0.025**
age	0.00	0.00 – 0.00	**0.001**
education [Associates degree]	-0.01	-0.07 – 0.06	0.875
education [Bachelor's degree]	0.03	-0.03 – 0.08	0.304
education [Did not complete high school / GED]	-0.03	-0.22 – 0.17	0.797
education [High school diploma / GED]	0.01	-0.06 – 0.08	0.827
education [Master's degree or higher]	0.02	-0.04 – 0.08	0.492
income [$100,000 - $149,999]	-0.02	-0.06 – 0.02	0.444
income [$30,000 - $49,999]	0.00	-0.05 – 0.06	0.880
income [$50,000 - $74,999]	0.01	-0.04 – 0.05	0.835
income [$75,000 - $99,999]	0.04	-0.01 – 0.08	0.111
income [More than $150,000]	-0.03	-0.08 – 0.02	0.232
racethn [European]	-0.02	-0.06 – 0.02	0.395
racethn [Hispanic]	-0.05	-0.10 – 0.00	0.070
racethn [African]	-0.01	-0.10 – 0.08	0.791
racethn [Asian]	0.10	0.00 – 0.19	**0.043**
racethn [moreThanOne]	-0.01	-0.11 – 0.09	0.813
ruralUrb [Suburb, outskirt of a big city]	-0.01	-0.07 – 0.05	0.744
ruralUrb [Urban, a big city]	-0.03	-0.10 – 0.03	0.323
**Random Effects**			
σ^2	0.29		
$\tau_{00\ \text{trialNum\_fixed}}$	0.13		
$\tau_{00\ \text{trialNum\_fixed}}$	0.00		
ICC	0.31		
N_{subject}	1451		
$N_{\text{trialNum\_fixed}}$	25		
Observations	34294		
Marginal R^2 / Conditional R^2	0.016 / 0.319		

Table S1: ***Mixed-effects models predicting ΔDuration (M1.1-M1.3).*** *ΔDuration (deltaDuration) represents relative dwell time spent on carbon information compared to bonus information. Random intercepts were included for participants (subject) and trials (trialNum_fixed) (N = 1451 participants). Model M1.1 included political affiliation (polAff_bin_str, subject-level binary variable with 0 = Republican and 1 = Democrat). Model M1.2 additionally controlled for the task characteristics: attribute differences (carbon_prcnt and bonus_prcnt), trial position (trialNum), and trial layout (CB_carbonUp and CB_selfishLeft). Model M1.3 further included participant-level covariates: gender, age, education, income, race/ethnicity (racethn), and area of residence (ruralUrb). The covariates education, income, and racethn were effect-coded using sum-to-zero contrasts, such that coefficients represent deviations from the overall mean. For each variable, one category is not displayed and can be derived as the negative sum of the reported coefficients. The omitted*

categories were Some college (education), Under $30,000 (income), and Other (racethn). In contrast, ruralUrb was dummy-coded, with Rural town or small city as the reference category. Estimates represent unstandardized beta coefficients. Confidence intervals are 95%. Statistically significant effects (p < .05) are shown in bold.

	M1.1		
Predictors	*Estimates*	*CI*	*p*
(Intercept)	0.03	0.01 – 0.06	**0.013**
polAff bin str [Dem]	0.06	0.03 – 0.10	**0.001**
**Random Effects**			
σ^2		0.28	
$\tau_{00\ \text{subject}}$		0.17	
$\tau_{00\ \text{trialNum\_fixed}}$		0.00	
ICC		0.38	
N_{subject}		1936	
$N_{\text{trialNum\_fixed}}$		25	
Observations		40823	
Marginal R^2 / Conditional R^2		0.002 / 0.380	

Table S2: ***Mixed-effects model predicting ΔDuration in the full sample (M1.1).*** *ΔDuration (deltaDuration) represents relative dwell time spent on carbon information compared to bonus information. This model was estimated on the full sample (N = 1,936 participants), including all participants with at least one valid ΔDuration observation across trials (participants without valid observations had either no box openings or only openings shorter than 200 ms). The fixed effect was political affiliation (polAff_bin_str, subject-level binary variable with 0 = Republican and 1 = Democrat). Random intercepts were included for participants (subject) and trials (trialNum_fixed). Estimates represent unstandardized beta coefficients. Confidence intervals are 95%. Statistically significant effects (p < .05) are shown in bold.*

1.1.1.1. Interactions Between Political Affiliation and Control Variables on ΔDuration

We additionally tested whether control variables with significant main effects moderate the relationship between political affiliation and ΔDuration (see Table S3).

The interaction between political affiliation and trial position was statistically significant ($b < 0.01$, $p = .008$), indicating that the difference in attention allocated to carbon information (ΔDuration) between Democrats and Republicans increased as participants progressed through the task. As illustrated in Figure S1, the effect of political affiliation on ΔDuration remained significant across all trials but became stronger at later stages of the task. The effect of political affiliation on ΔDuration was also significantly moderated by age ($b < 0.01$, $p = .011$). As shown in Figure S2, the effect of political affiliation on ΔDuration increases with age and becomes statistically significant at approximately 38 years of age (≈0.5 *SD* below the sample mean), indicating that partisan differences in attention allocation are more pronounced among older individuals. The effect of political affiliation on ΔDuration

was also significantly moderated by race/ethnicity for participants identifying as Asian (*b* = 0.25, *p* = .012), suggesting that partisan differences in attention allocation in this subgroup differed from the average effect across all racial/ethnic groups. In contrast, the interaction between political affiliation and carbon attribute placement as well as gender was not statistically significant.

	**M1_trialNum**			**M1_CB_carbonUp**		
Predictors	*Estimates*	*CI*	*p*	*Estimates*	*CI*	*p*
(Intercept)	-0.01	-0.09 – 0.06	0.727	-0.04	-0.12 – 0.04	0.280
polAff bin str [Dem]	0.04	-0.01 – 0.08	0.111	0.10	0.04 – 0.16	**<0.001**
trialNum	0.00	-0.00 – 0.00	0.137	0.00	0.00 – 0.00	**<0.001**
CB carbonUp	-0.04	-0.08 – -0.00	**0.029**	-0.01	-0.06 – 0.04	0.704
gender [Male]	-0.05	-0.09 – -0.01	**0.014**	-0.05	-0.09 – -0.01	**0.015**
age	0.00	0.00 – 0.00	**<0.001**	0.00	0.00 – 0.00	**<0.001**
racethn [European]	-0.01	-0.05 – 0.03	0.543	-0.01	-0.05 – 0.03	0.528
racethn [Hispanic]	-0.05	-0.10 – -0.00	**0.044**	-0.05	-0.10 – -0.00	**0.046**
racethn [African]	-0.02	-0.11 – 0.07	0.634	-0.02	-0.11 – 0.06	0.603
racethn [Asian]	0.11	0.02 – 0.21	**0.015**	0.12	0.03 – 0.21	**0.013**
racethn [moreThanOne]	-0.01	-0.11 – 0.08	0.797	-0.01	-0.11 – 0.08	0.783
polAff bin str [Dem] × trialNum	0.00	0.00 – 0.00	**0.008**			
polAff bin str [Dem] × CB carbonUp				-0.07	-0.15 – 0.01	0.073
**Random Effects**						
σ^2	0.29			0.29		
τ_{00}	0.13 $_{subject}$			0.13 $_{subject}$		
	0.00 $_{trialNum\_fixed}$			0.00 $_{trialNum\_fixed}$		
ICC	0.31			0.31		
N	1451 $_{subject}$			1451 $_{subject}$		
	25 $_{trialNum\_fixed}$			25 $_{trialNum\_fixed}$		
Observations	34294			34294		
Marginal R^2 / Conditional R^2	0.016 / 0.317			0.017 / 0.317		

	M1_gender			M1_age		
Predictors	*Estimates*	*CI*	*p*	*Estimates*	*CI*	*p*
(Intercept)	-0.03	-0.12 – 0.05	0.425	0.05	-0.05 – 0.14	0.306
polAff bin str [Dem]	0.07	0.02 – 0.13	**0.008**	-0.09	-0.20 – 0.03	0.128
gender [Male]	-0.04	-0.09 – 0.01	0.138	-0.05	-0.09 – -0.01	**0.014**
trialNum	0.00	0.00 – 0.00	**<0.001**	0.00	0.00 – 0.00	**<0.001**
CB carbonUp	-0.04	-0.08 – -0.00	**0.029**	-0.04	-0.08 – -0.00	**0.033**
age	0.00	0.00 – 0.00	**<0.001**	0.00	-0.00 – 0.00	0.381
racethn [European]	-0.01	-0.05 – 0.03	0.539	-0.01	-0.05 – 0.03	0.770
racethn [Hispanic]	-0.05	-0.10 – -0.00	**0.048**	-0.06	-0.11 – -0.01	**0.021**
racethn [African]	-0.02	-0.11 – 0.07	0.641	-0.03	-0.12 – 0.06	0.537
racethn [Asian]	0.11	0.02 – 0.21	**0.015**	0.11	0.02 – 0.21	**0.016**
racethn [moreThanOne]	-0.01	-0.11 – 0.08	0.795	-0.01	-0.11 – 0.08	0.809
polAff bin str [Dem] × gender [Male]	-0.02	-0.10 – 0.06	0.620			
polAff bin str [Dem] × age				0.00	0.00 – 0.01	**0.005**
**Random Effects**						
σ^2	0.29			0.29		
τ_{00}	0.13 subject			0.13 subject		
	0.00 trialNum_fixed			0.00 trialNum_fixed		
ICC	0.31			0.31		
N	1451 subject			1451 subject		
	25 trialNum_fixed			25 trialNum_fixed		
Observations	34294			34294		
Marginal R^2 / Conditional R^2	0.016 / 0.317			0.018 / 0.317		

	M1_racethn		
Predictors	*Estimates*	*CI*	*p*
(Intercept)	-0.05	-0.14 – 0.03	0.232
polAff bin str [Dem]	0.09	0.02 – 0.16	**0.013**
racethn [European]	-0.00	-0.07 – 0.06	0.890
racethn [Hispanic]	-0.00	-0.07 – 0.07	0.906
racethn [African]	0.00	-0.16 – 0.16	0.996
racethn [Asian]	-0.04	-0.20 – 0.11	0.606
racethn [moreThanOne]	0.03	-0.11 – 0.17	0.689
trialNum	0.00	0.00 – 0.00	**<0.001**
CB carbonUp	-0.04	-0.08 – -0.01	**0.025**
gender [Male]	-0.05	-0.09 – -0.01	**0.014**
age	0.00	0.00 – 0.00	**<0.001**
polAff bin str [Dem] × racethn [European]	0.00	-0.08 – 0.08	0.988
polAff bin str [Dem] × racethn [Hispanic]	-0.08	-0.18 – 0.01	0.074
polAff bin str [Dem] × racethn [African]	-0.03	-0.22 – 0.17	0.790
polAff bin str [Dem] × racethn [Asian]	0.25	0.06 – 0.44	**0.012**
polAff bin str [Dem] × racethn [moreThanOne]	-0.07	-0.26 – 0.13	0.509
**Random Effects**			
σ^2	0.29		
τ_{00} subject	0.13		
τ_{00} trialNum_fixed	0.00		
ICC	0.31		
$N_{subject}$	1451		
$N_{trialNum\_fixed}$	25		
Observations	34294		
Marginal R^2 / Conditional R^2	0.018 / 0.318		

Table S3: ***Mixed-effects models predicting ΔDuration including interactions with control variables (M1).*** *ΔDuration (deltaDuration) represents relative dwell time spent on carbon information compared to bonus information. The models include interactions between political affiliation (polAff_bin_str) and control variables that showed significant main effects in M1.2–M1.3: trialNum, CB_carbonUP, gender and age. Random intercepts were included for participants (subject) and trials (trialNum_fixed) (N = 1451 participants). Estimates represent unstandardized beta coefficients. Confidence intervals are 95%. Statistically significant effects (p < .05) are shown in bold.*

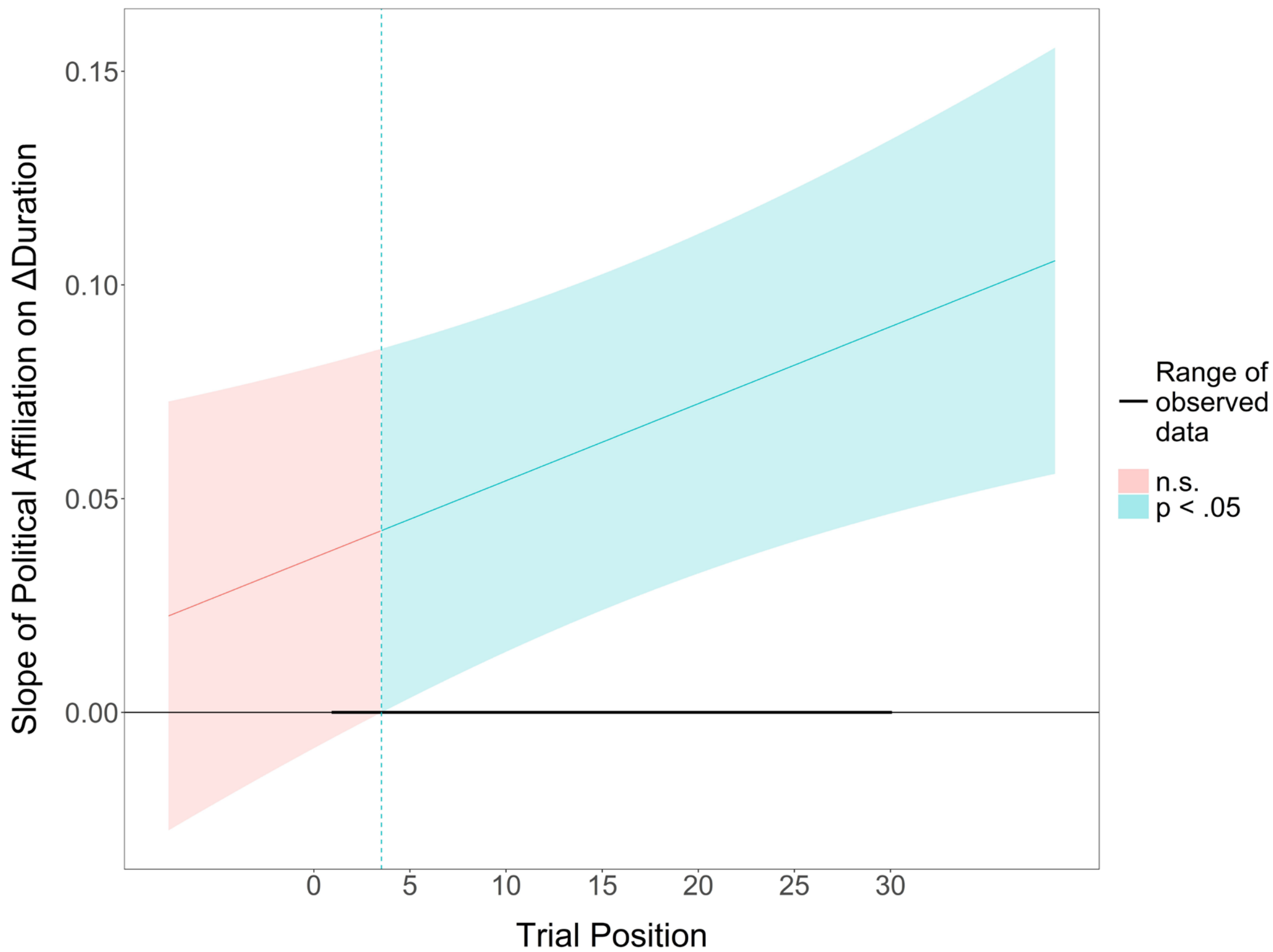

Figure S1: ***Interaction between political affiliation and trial position on ΔDuration.*** *The Johnson–Neyman plot illustrates the interaction between political affiliation and trial position estimated by Model M1_trialNum (N = 1,451 participants). The line represents the conditional effect of political affiliation on ΔDuration across values of trial position. Shaded areas indicate regions where the effect is statistically significant (p < .05; blue) or non-significant (red). The dashed vertical line marks the Johnson–Neyman significance threshold. The black horizontal bar represents the observed range of the moderator.*

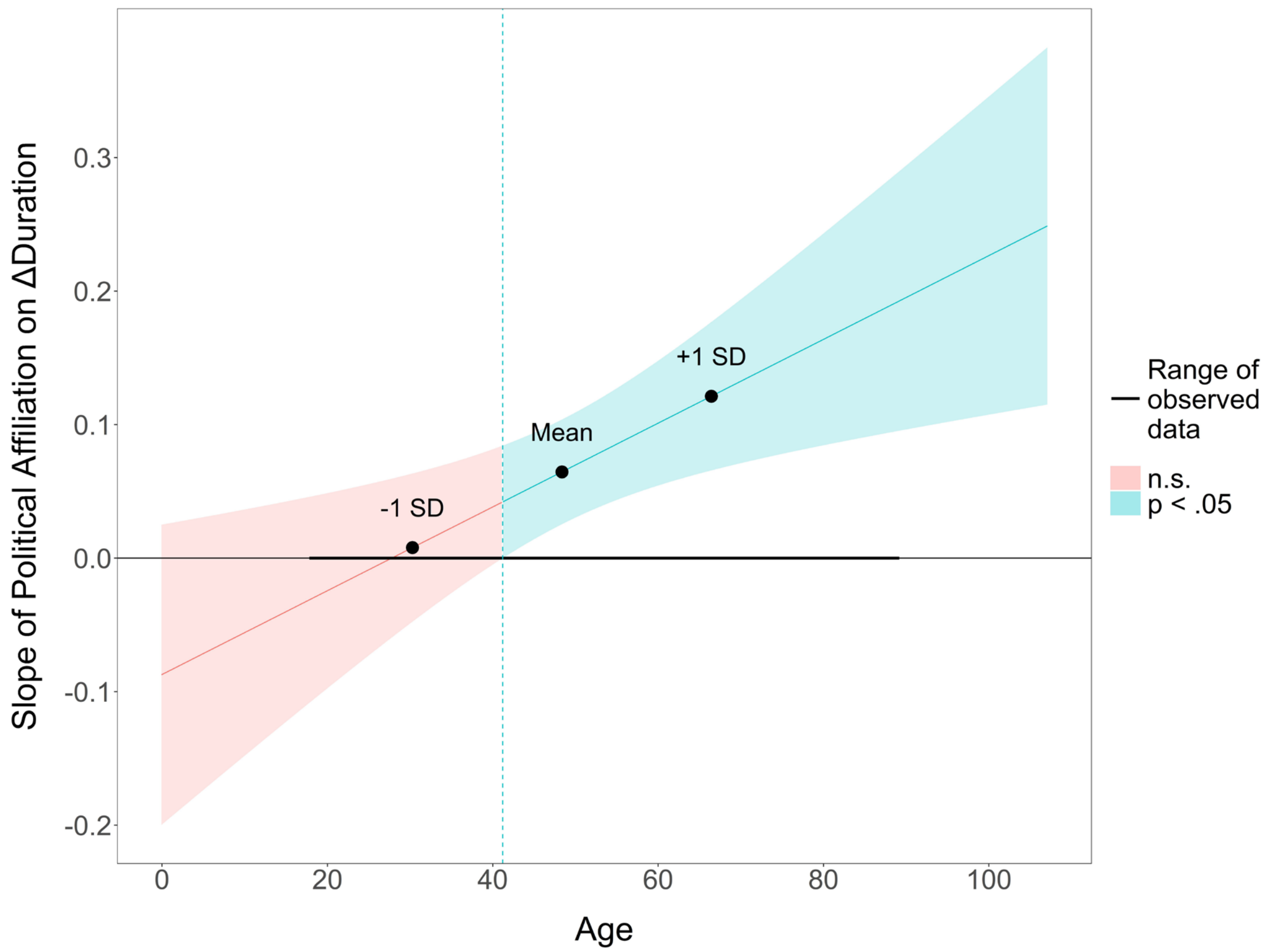

Figure S2: ***Interaction between political affiliation and age on ΔDuration.*** *The Johnson–Neyman plot illustrates the interaction between political affiliation and age estimated by Model M1_age (N = 1,451 participants). The line represents the conditional effect of political affiliation on ΔDuration across values of age. Shaded areas indicate regions where the effect is statistically significant (p < .05; blue) or non-significant (red). The dashed vertical line marks the Johnson–Neyman significance threshold. Points indicate the conditional effects at −1 SD, the mean, and +1 SD of age. The black horizontal bar represents the observed range of the moderator.*

1.1.2. Effect of Political Affiliation on Choice (H_{S1})

We tested the hypothesis that Democrats have a higher probability of making pro-climate choices than Republicans (H_{S1}). To this end, we estimated mixed-effects logistic regression models with the decision outcome as the dependent variable and random intercepts for participants and trials, using the same three model specifications as in the ΔDuration analysis. All regression results are reported in Table S4.

Consistent with our hypothesis, political affiliation significantly predicted pro-climate choices across all model specifications. In Model 1, Democrats were significantly more likely than Republicans to choose the pro-climate option ($OR = 2.06$, $p < .001$). The predicted probability of selecting the pro-climate option was 65.9% for Democrats, compared with 48.3% for Republicans. This effect remained unchanged after controlling task characteristics in Model 2 ($OR = 2.05$, $p < .001$) and after additionally including participant level controls in Model 3 ($OR = 2.03$, $p < .001$). Among the task-related control variables (Model 2), the percentage difference in carbon emissions was positively

associated with pro climate choices (*OR* = 1.01, $p < .001$), indicating that larger emission differences increased the likelihood of choosing the pro-climate option. Furthermore, larger bonus differences were associated with a lower probability of selecting the pro-climate option (*OR* = 0.99, $p < .001$). Consistent with the analysis on attention allocation, older participants were slightly more likely to select the pro-climate option (*OR* = 1.01, $p = .007$). Additionally, participants identifying as African were less likely to select the pro-climate option relative to the overall mean (*OR* = 0.24, $p < .001$).

	**M1.1**			**M1.2**		
Predictors	*Odds Ratios*	*CI*	*p*	*Odds Ratios*	*CI*	*p*
(Intercept)	0.94	0.70 – 1.25	0.648	0.97	0.72 – 1.32	0.862
polAff bin str [Dem]	2.06	1.51 – 2.83	**<0.001**	2.05	1.50 – 2.81	**<0.001**
carbon prcnt				1.01	1.01 – 1.01	**<0.001**
bonus prcnt				0.99	0.99 – 0.99	**<0.001**
trialNum				1.00	1.00 – 1.01	0.193
CB carbonUp				0.89	0.65 – 1.22	0.462
CB selfishLeft				1.01	0.95 – 1.07	0.852
**Random Effects**						
σ^2	3.29			3.29		
τ_{00}	8.50 $_{subject}$			8.49 $_{subject}$		
	0.24 $_{trialNum\_fixed}$			0.02 $_{trialNum\_fixed}$		
ICC	0.73			0.72		
N	1451 $_{subject}$			1451 $_{subject}$		
	25 $_{trialNum\_fixed}$			25 $_{trialNum\_fixed}$		
Observations	34428			34428		
Marginal R^2 / Conditional R^2	0.011 / 0.729			0.029 / 0.729		

	M1.3		
Predictors	*Odds Ratios*	*CI*	*p*
(Intercept)	0.43	0.18 – 1.03	0.059
polAff bin str [Dem]	2.13	1.55 – 2.93	**<0.001**
gender [Male]	0.86	0.62 – 1.19	0.358
age	1.02	1.01 – 1.03	**0.002**
education [Associates degree]	0.78	0.45 – 1.34	0.365
education [Bachelor's degree]	0.97	0.63 – 1.51	0.903
education [Did not complete high school / GED]	0.90	0.18 – 4.50	0.900
education [High school diploma / GED]	1.12	0.63 – 1.99	0.695
education [Master's degree or higher]	1.10	0.68 – 1.78	0.693
income [$100,000 - $149,999]	0.84	0.61 – 1.16	0.285
income [$30,000 - $49,999]	1.17	0.75 – 1.82	0.497
income [$50,000 - $74,999]	0.96	0.65 – 1.41	0.819
income [$75,000 - $99,999]	0.80	0.55 – 1.16	0.235
income [More than $150,000]	0.79	0.53 – 1.17	0.236
racethn [European]	1.10	0.80 – 1.52	0.560
racethn [Hispanic]	1.50	0.99 – 2.26	0.054
racethn [African]	0.24	0.12 – 0.49	**<0.001**
racethn [Asian]	2.02	0.95 – 4.29	0.067
racethn [moreThanOne]	1.05	0.48 – 2.31	0.896
ruralUrb [Suburb, outskirt of a big city]	1.34	0.81 – 2.20	0.250
ruralUrb [Urban, a big city]	0.73	0.43 – 1.24	0.240
**Random Effects**			
σ^2	3.29		
$\tau_{00\ \text{subject}}$	8.07		
$\tau_{00\ \text{trialNum\_fixed}}$	0.24		
ICC	0.72		
N_{subject}	1451		
$N_{\text{trialNum\_fixed}}$	25		
Observations	34428		
Marginal R^2 / Conditional R^2	0.045 / 0.729		

Table S4: ***Mixed-effects logistic regression models predicting choice (M1.1–M1.3).*** *Choice represents the actual decision in the Carbon Emission Task (trial-level variable with 0 = pro-self and 1 = pro-climate). Random intercepts were included for participants (subject) and trials (trialNum_fixed) (N = 1451 participants). Model M1.1 included political affiliation (polAff_bin_str, subject-level binary variable with 0 = Republican and 1 = Democrat). Model M1.2 additionally controlled for the task characteristics: attribute differences (carbon_prcnt and bonus_prcnt), trial position (trialNum), and trial layout (CB_carbonUp and CB_selfishLeft). Model M1.3 further included participant-level covariates: gender, age, education, income, race/ethnicity (racethn), and area of residence (ruralUrb). The covariates education, income, and racethn were effect-coded using sum-to-zero contrasts, such that coefficients represent deviations from the overall mean. For each variable, one category is not displayed and can be derived as the negative sum of the*

reported coefficients. The omitted categories were Some college (education), Under $30,000 (income), and Other (racethn). In contrast, ruralUrb was dummy-coded, with Rural town or small city as the reference category. Confidence intervals are 95%. Statistically significant effects (p < .05) are shown in bold.

1.2. Political Affiliation and Extreme Weather Events (H_2)

Among Republicans, 48.2% of participants lived in counties where at least one episode occurred during the past 30 days (374 of 776), compared with 43.4% among Democrats (293 of 675).

Figure S3 displays separate county-level maps of the United States for each extreme weather event (EWE) type and for all types combined, indicating whether and how many episodes occurred in each county during the 360 days preceding the midpoint of data collection.

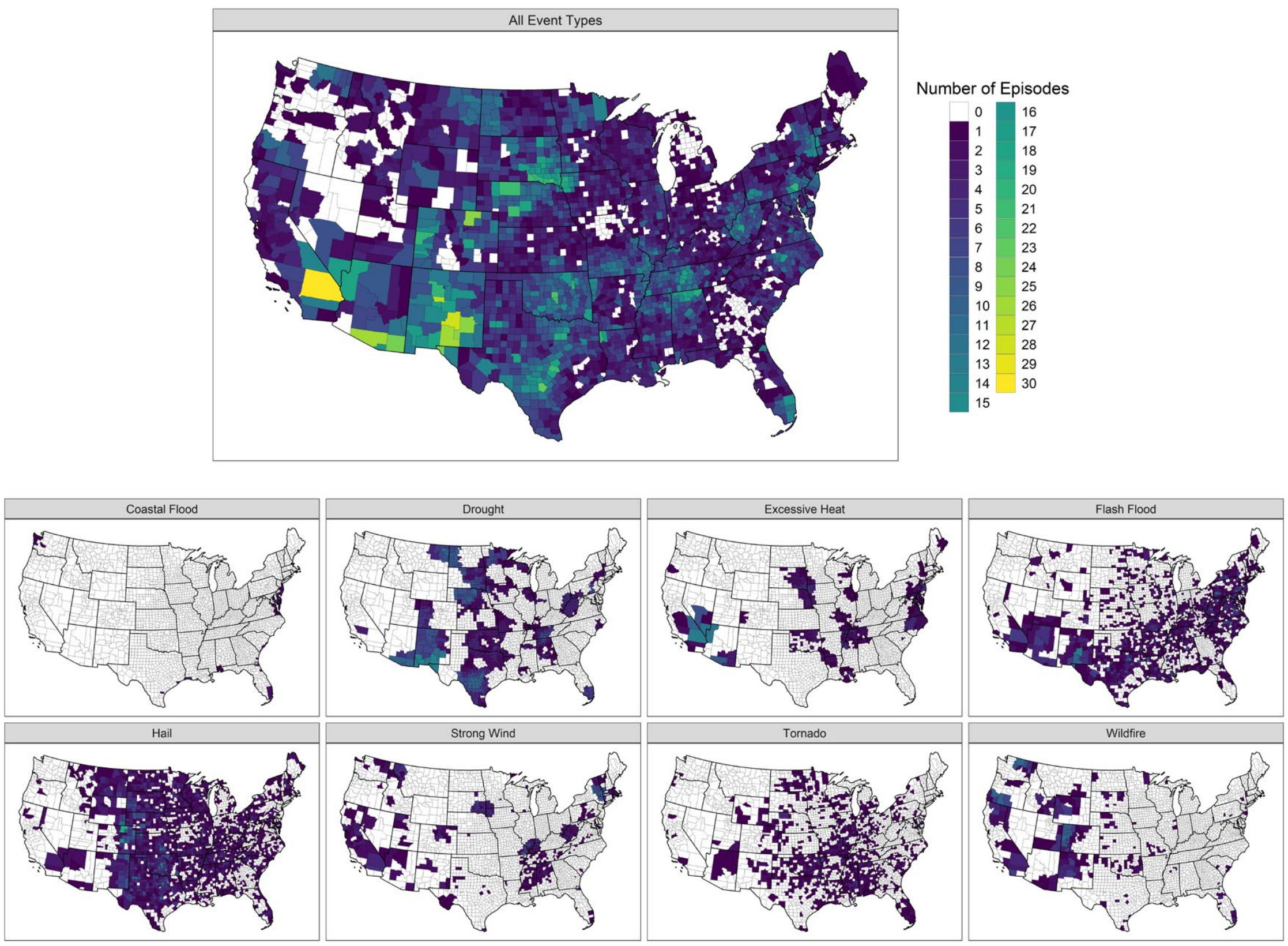

Figure S3: ***Distribution of extreme weather episodes across U.S. counties (past 360 days).*** *Counties are colored by the number of EWE recorded in the past 360 days preceding the midpoint of data collection (≈50% of the sample collected, August 23, 2025). This reference point was chosen because data collection spanned approximately four weeks. Map boundaries are based on the U.S. Census Bureau Cartographic Boundary Files and were visualized using the R package usmap.*

1.2.1. Extreme Weather Events as a Moderator of the Relationship Between Political Affiliation and ΔDuration (H_2)

To examine whether exposure to EWEs moderates the relationship between political affiliation and visual attention, we estimated mixed-effects models with ΔDuration as the dependent variable and random intercepts for participants and trials. Model 1 included political affiliation and a binary indicator of whether participants had experienced at least one EWE episode in the last 30 days. Model 2 additionally included their interaction. Model 3 further controlled for trial number, the position of the carbon attribute, gender, age and race / ethnicity. Model 4 instead controlled for county-level indicators related to EWE severity and impacts (e.g., damage, injuries, deaths) as well as broader county characteristics (mean income, PVI, and population density). Model 5 replaced the binary EWE indicator with a continuous, duration-weighted measure of EWE exposure and its interaction with political affiliation. All regression results are displayed in Table S5.

In Model 1, political affiliation remained a positive and significant predictor of ΔDuration (b = 0.08, p < .001). In contrast, experiencing at least one EWE was not significantly associated with greater relative attention to carbon information. Model 2 included the interaction between political affiliation and EWE exposure; however, this interaction was not statistically significant (b = -0.05, p = .188), providing no support for the hypothesis that prior exposure amplifies partisan differences in visual attention. This null result was robust to the inclusion of control variables (Table S5, Models 3-4, b = -0.07, p = .092, b = -0.05, p = .202), the use of a duration-weighted measure of exposure (Table S5, Model 5, b = -0.00, p = .124), and analyses based on the full sample (Table S6, b = -0.05, p = .189).

Among the control variables, trial number was positively associated with ΔDuration (b = <0.01, p < .001), whereas higher carbon levels were associated with reduced attention to carbon information (b = −0.04, p = .031). Male participants (b = −0.05, p = .013) and participants identifying as Hispanic (b = −0.05, p = .043) showed lower relative attention to carbon information. In contrast, older participants (b = <0.01, p < .001), as well as participants identifying as Asian showed greater attention (b = 0.11, p = .020).

We conducted equivalence tests for non-significant effects to assess whether they were statistically smaller than the smallest effect size of interest (SESOI = 0.1143). As illustrated in Figure S4, the confidence intervals of the interaction terms overlapped the region of practical equivalence (ROPE), suggesting that it is undecided whether to accept or reject practical equivalence of the moderating effect of EWE exposure on the relationship between political affiliation and ΔDuration.

	M2.1			M2.2		
Predictors	*Estimates*	*CI*	*p*	*Estimates*	*CI*	*p*
(Intercept)	0.04	0.01 – 0.08	**0.011**	0.03	-0.01 – 0.07	0.102
polAff bin str [Dem]	0.08	0.04 – 0.12	**<0.001**	0.10	0.05 – 0.16	**<0.001**
nEpisodesIsGreaterThanZeroTRUE	-0.01	-0.05 – 0.03	0.713	0.02	-0.04 – 0.07	0.532
polAff bin str [Dem] × nEpisodesIsGreaterThanZeroTRUE				-0.05	-0.13 – 0.03	0.188
**Random Effects**						
σ^2	0.29			0.29		
τ_{00}	0.13 $_{subject}$			0.13 $_{subject}$		
	0.00 $_{trialNum\_fixed}$			0.00 $_{trialNum\_fixed}$		
ICC	0.31			0.31		
N	1451 $_{subject}$			1451 $_{subject}$		
	25 $_{trialNum\_fixed}$			25 $_{trialNum\_fixed}$		
Observations	34294			34294		
Marginal R^2 / Conditional R^2	0.004 / 0.316			0.004 / 0.316		

	M2.3		
Predictors	*Estimates*	*CI*	*p*
(Intercept)	-0.05	-0.13 – 0.04	0.282
polAff bin str [Dem]	0.10	0.04 – 0.15	**<0.001**
nEpisodesIsGreaterThanZeroTRUE	0.03	-0.02 – 0.09	0.228
trialNum	0.00	0.00 – 0.00	**<0.001**
CB carbonUp	-0.04	-0.08 – -0.00	**0.031**
gender [Male]	-0.05	-0.09 – -0.01	**0.013**
age	0.00	0.00 – 0.00	**<0.001**
racethn [European]	-0.01	-0.05 – 0.03	0.540
racethn [Hispanic]	-0.05	-0.10 – -0.00	**0.043**
racethn [African]	-0.02	-0.11 – 0.07	0.660
racethn [Asian]	0.11	0.02 – 0.20	**0.020**
racethn [moreThanOne]	-0.01	-0.11 – 0.09	0.836
polAff bin str [Dem] × nEpisodesIsGreaterThanZeroTRUE	-0.07	-0.14 – 0.01	0.092
**Random Effects**			
σ^2	0.29		
$\tau_{00\ subject}$	0.13		
$\tau_{00\ trialNum\_fixed}$	0.00		
ICC	0.31		
$N_{subject}$	1451		
$N_{trialNum\_fixed}$	25		
Observations	34294		
Marginal R^2 / Conditional R^2	0.017 / 0.318		

	M2.4		
Predictors	*Estimates*	*CI*	*p*
(Intercept)	0.03	-0.01 – 0.07	0.147
polAff bin str [Dem]	0.11	0.06 – 0.16	**<0.001**
nEpisodesIsGreaterThanZeroTRUE	0.04	-0.02 – 0.10	0.234
abnormality final	0.00	-0.02 – 0.02	0.983
sum damage	0.00	-0.02 – 0.02	0.704
sum injuries	0.01	-0.01 – 0.03	0.568
sum deaths	0.01	-0.01 – 0.03	0.604
recency index	-0.19	-0.45 – 0.08	0.161
population density peopPerSqKilometer	0.02	-0.00 – 0.04	0.093
countyPvi	-0.02	-0.04 – 0.01	0.216
countyIncome	-0.02	-0.04 – 0.01	0.164
polAff bin str [Dem] × nEpisodesIsGreaterThanZeroTRUE	-0.05	-0.13 – 0.03	0.202
**Random Effects**			
σ^2	0.29		
$\tau_{00\ subject}$	0.13		
$\tau_{00\ trialNum\_fixed}$	0.00		
ICC	0.31		
$N_{subject}$	1451		
$N_{trialNum\_fixed}$	25		
Observations	34294		
Marginal R^2 / Conditional R^2	0.006 / 0.317		

	M2.5		
Predictors	*Estimates*	*CI*	*p*
(Intercept)	0.03	0.00 – 0.06	**0.021**
polAff bin str [Dem]	0.09	0.05 – 0.13	**<0.001**
nEpisodes durationWeighted	0.00	-0.00 – 0.00	0.216
polAff bin str [Dem] × nEpisodes durationWeighted	-0.00	-0.01 – 0.00	0.124
**Random Effects**			
σ^2	0.29		
$\tau_{00\ subject}$	0.13		
$\tau_{00\ trialNum\_fixed}$	0.00		
ICC	0.31		
$N_{subject}$	1451		
$N_{trialNum\_fixed}$	25		
Observations	34294		
Marginal R^2 / Conditional R^2	0.004 / 0.316		

*Table S5: **Mixed-effects models predicting ΔDuration (M2.1–M2.5).** ΔDuration (deltaDuration) represents relative dwell time spent on carbon information compared to bonus information. Random intercepts were included for participants (subject) and trials (trialNum_fixed) (N = 1451 participants). Model M2.1 included political affiliation (polAff_bin_str, subject-level binary variable with 0 = Republican and 1 = Democrat) and a binary indicator of whether participants had experienced at least one EWE episode in the last 30 days (nEpisodesIsGreaterThanZeroTRUE). Model M2.2 additionally included their interaction. Model M2.3 further controlled for trial position (trialNum), the position of the carbon attribute (CB_carbonUp), gender, age and race/ethnicity (racethn). racethn was effect-coded using sum-to-zero contrasts, such that coefficients represent deviations from the overall mean. The category Other is not displayed and can be derived as the negative sum of the reported coefficients. Model M2.4 instead controlled for county-level indicators related to EWE severity and impacts, specifically the abnormality score for the number of EWE (abnormality_final), the amount of financial damage (sum_damage), and the number of injuries (sum_injuries) and deaths (sum_deaths) associated with each episode. The model further controlled for the temporal proximity of the most recent extreme weather episode (recency_index), as well as broader county characteristics: population density (population_density_peopPerSqKilometer), median household income (countyIncome), and the county-level Partisan Voting Index (countyPvi). All county-level variables included as controls in Model M2.4 were z-standardized, except recency_index. Model M2.5 replaced the binary EWE indicator with a continuous, duration-weighted measure of EWE exposure (nEpisodes_durationWeighted) and its interaction with political affiliation. Estimates represent unstandardized beta coefficients. Confidence intervals are 95%. Statistically significant effects (p < .05) are shown in bold.*

	M2.2		
Predictors	*Estimates*	*CI*	*p*
(Intercept)	0.02	-0.02 – 0.05	0.391
polAff bin str [Dem]	0.09	0.04 – 0.14	**0.001**
nEpisodesIsGreaterThanZeroTRUE	0.04	-0.02 – 0.09	0.174
polAff bin str [Dem] × nEpisodesIsGreaterThanZeroTRUE	-0.05	-0.13 – 0.03	0.189
**Random Effects**			
σ^2		0.28	
$\tau_{00\ subject}$		0.17	
$\tau_{00\ trialNum\_fixed}$		0.00	
ICC		0.38	
$N_{subject}$		1936	
$N_{trialNum\_fixed}$		25	
Observations		40823	
Marginal R^2 / Conditional R^2		0.003 / 0.380	

Table S6: ***Mixed-effects model predicting ΔDuration in the full sample (M2.2).*** *ΔDuration (deltaDuration) represents relative dwell time spent on carbon information compared to bonus information. This model was estimated on the full sample (N = 1,936 participants), including all participants with at least one valid ΔDuration observation across trials (participants without valid observations had either no box openings or only openings shorter than 200 ms). The fixed effects were political affiliation (polAff_bin_str, subject-level binary variable with 0 = Republican and 1 = Democrat), a binary indicator of whether participants had experienced at least one EWE episode in the last 30 days (nEpisodesIsGreaterThanZeroTRUE), and their interaction. Random intercepts were included for participants (subject) and trials (trialNum_fixed). Estimates represent unstandardized beta coefficients. Confidence intervals are 95%. Statistically significant effects (p < .05) are shown in bold.*

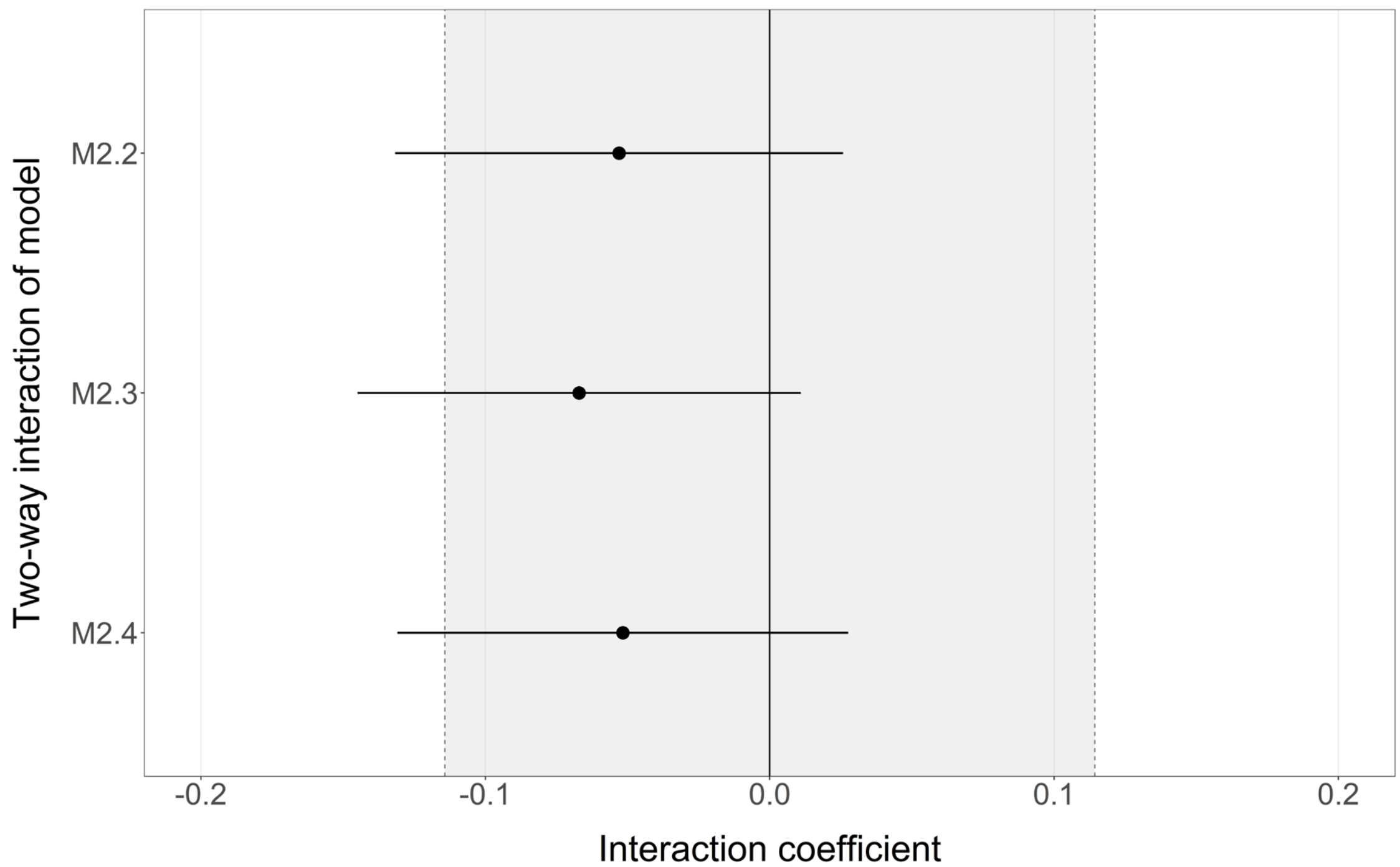

Figure S4: ***Equivalence testing of interaction coefficients across models (M2.2–M2.4).*** *Points represent estimated interaction coefficients for the interaction between political affiliation and EWE predicting ΔDuration across Models M2.2–2.4 (N = 1,451 participants), and horizontal lines indicate 95% confidence intervals. The shaded area denotes the region of practical equivalence (ROPE; ±0.1143), based on the smallest effect size of interest (SESOI).*

1.2.1.1. Interactions with County-Level Characteristics

We additionally explored whether county-level characteristics moderate the interaction between political affiliation and EWE exposure. Three-way interactions with population density, median household income, and county-level PVI were not statistically significant. Full results are reported in Table S7 and Figure S5.

	M2_popDensity		
Predictors	*Estimates*	*CI*	*p*
(Intercept)	0.03	-0.01 – 0.07	0.099
polAff bin str [Dem]	0.10	0.05 – 0.15	**<0.001**
nEpisodesIsGreaterThanZeroTRUE	0.02	-0.04 – 0.07	0.576
population density peopPerSqKilometer	-0.01	-0.04 – 0.02	0.707
polAff bin str [Dem] × nEpisodesIsGreaterThanZeroTRUE	-0.04	-0.13 – 0.04	0.279
polAff bin str [Dem] × population density peopPerSqKilometer	0.03	-0.01 – 0.08	0.101
nEpisodesIsGreaterThanZeroTRUE × population density peopPerSqKilometer	-0.01	-0.09 – 0.07	0.764
(polAff bin str [Dem] × nEpisodesIsGreaterThanZeroTRUE) × population density peopPerSqKilometer	0.00	-0.15 – 0.15	0.985
**Random Effects**			
σ^2	0.29		
$\tau_{00\ subject}$	0.13		
$\tau_{00\ trialNum\_fixed}$	0.00		
ICC	0.31		
N $_{subject}$	1451		
N $_{trialNum\_fixed}$	25		
Observations	34294		
Marginal R^2 / Conditional R^2	0.005 / 0.317		

	M2_countyIncome		
Predictors	*Estimates*	*CI*	*p*
(Intercept)	0.03	-0.00 – 0.07	0.084
polAff bin str [Dem]	0.10	0.05 – 0.16	**<0.001**
nEpisodesIsGreaterThanZeroTRUE	0.01	-0.04 – 0.07	0.609
countyIncome	-0.03	-0.07 – -0.00	**0.041**
polAff bin str [Dem] × nEpisodesIsGreaterThanZeroTRUE	-0.05	-0.13 – 0.03	0.193
polAff bin str [Dem] × countyIncome	0.03	-0.02 – 0.07	0.259
nEpisodesIsGreaterThanZeroTRUE × countyIncome	0.03	-0.04 – 0.09	0.405
(polAff bin str [Dem] × nEpisodesIsGreaterThanZeroTRUE) × countyIncome	-0.03	-0.13 – 0.06	0.450
**Random Effects**			
σ^2	0.29		
$\tau_{00\ subject}$	0.13		
$\tau_{00\ trialNum\_fixed}$	0.00		
ICC	0.31		
$N_{subject}$	1451		
$N_{trialNum\_fixed}$	25		
Observations	34294		
Marginal R^2 / Conditional R^2	0.005 / 0.317		

	M2_countyPvi		
Predictors	*Estimates*	*CI*	*p*
(Intercept)	0.02	-0.02 – 0.06	0.280
polAff bin str [Dem]	0.11	0.06 – 0.17	**<0.001**
nEpisodesIsGreaterThanZeroTRUE	0.03	-0.03 – 0.08	0.315
countyPvi	-0.04	-0.07 – -0.00	**0.025**
polAff bin str [Dem] × nEpisodesIsGreaterThanZeroTRUE	-0.07	-0.15 – 0.01	0.092
polAff bin str [Dem] × countyPvi	0.03	-0.02 – 0.08	0.209
nEpisodesIsGreaterThanZeroTRUE × countyPvi	0.03	-0.02 – 0.09	0.231
(polAff bin str [Dem] × nEpisodesIsGreaterThanZeroTRUE) × countyPvi	0.01	-0.07 – 0.09	0.779
**Random Effects**			
σ^2	0.29		
$\tau_{00\ subject}$	0.13		
$\tau_{00\ trialNum\_fixed}$	0.00		
ICC	0.31		
$N_{subject}$	1451		
$N_{trialNum\_fixed}$	25		
Observations	34294		
Marginal R^2 / Conditional R^2	0.006 / 0.317		

Table S7: ***Mixed-effects models predicting ΔDuration including interactions with county-level characteristics (M2).*** *ΔDuration (deltaDuration) represents relative dwell time spent on carbon information compared to bonus information). Random intercepts were included for participants (subject) and trials (trialNum_fixed) (N = 1451 participants). Models included a three-way interaction between political affiliation (polAff_bin_str), EWE exposure (nEpisodesIsGreaterThanZeroTRUE), and one county-level characteristic: population density (population_density_peopPerSqKilometer), median household income (countyIncome), or Partisan Voting Index (countyPvi), along with all corresponding lower-order terms. County-level variables were z-standardized prior to analysis. Estimates represent unstandardized beta coefficients. Confidence intervals are 95%. Statistically significant effects (p < .05) are shown in bold.*

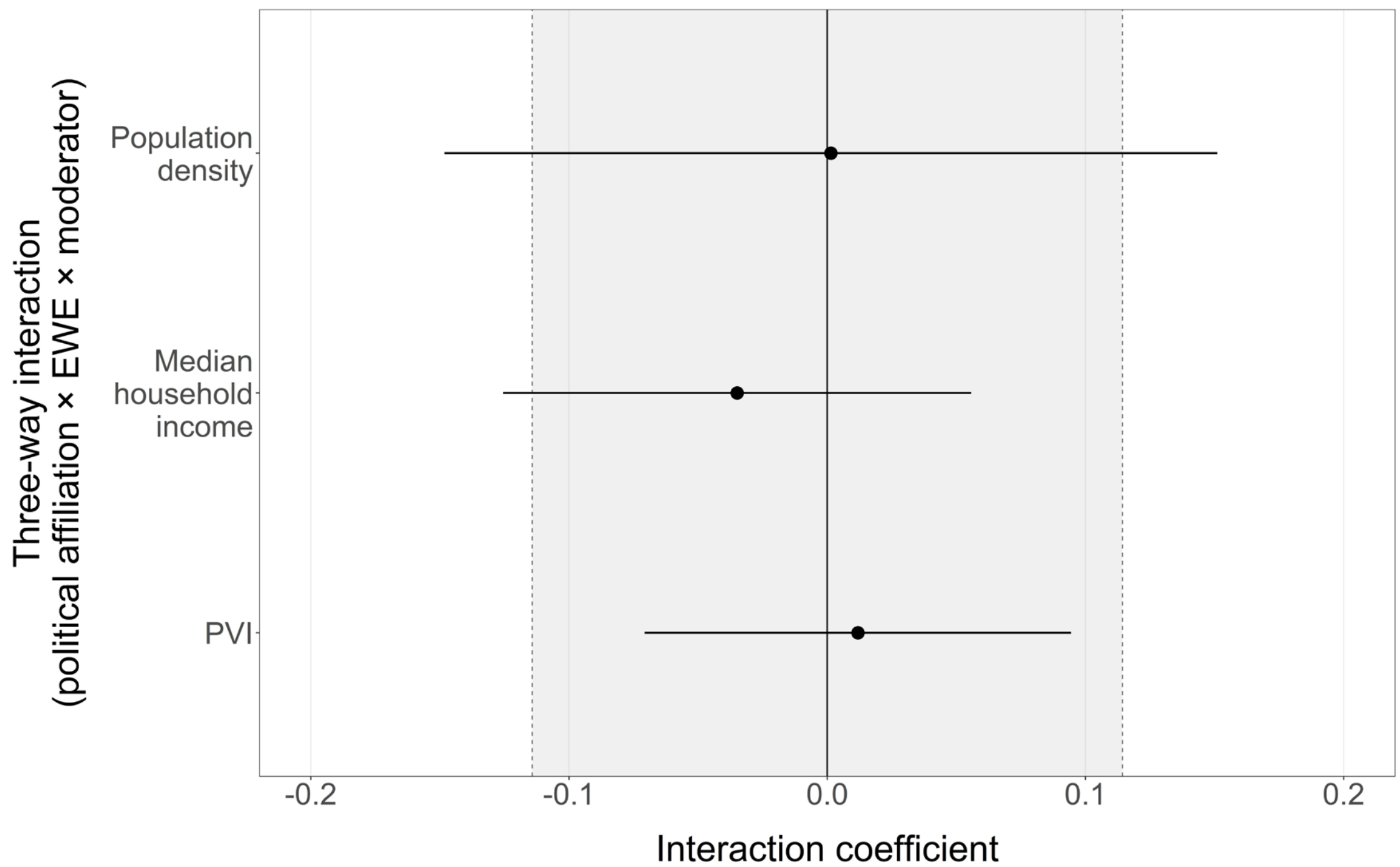

Figure S5: ***Equivalence testing of interaction coefficients across models with county-level moderators (M2).*** *Points represent estimated three-way interaction coefficients for the interaction between political affiliation, EWE exposure, and county-level characteristics predicting ΔDuration (N = 1,451 participants), and horizontal lines indicate 95% confidence intervals. County-level characteristics include population density, median household income, and Partisan Voting Index. The shaded area denotes the region of practical equivalence (ROPE; ±0.1143), based on the smallest effect size of interest (SESOI).*

1.2.1.2. Sensitivity Analysis for Extreme Weather Events

Furthermore, we conducted preregistered sensitivity analyses testing whether the interaction between political affiliation and EWE exposure depends on the time window used to measure exposure (1–12 months prior to study completion). An overview of the average number of episodes per time window is provided in Table S8. Across all time windows, the interaction between political affiliation and the number of EWE remained statistically non-significant (see Table S9). As shown in Figure S6, the confidence intervals of all interaction terms lie entirely within the ROPE (±0.1143), indicating practical equivalence.

Time window (days since event)	Mean nEpisodes	*SD* nEpisodes
30	0.87	1.26
60	1.86	2.35
90	2.66	3.18
120	3.45	3.73
150	4.04	4.04
180	4.52	4.25
210	4.81	4.32
240	4.94	4.39
270	5.09	4.50
300	5.39	4.75
330	5.68	5.05
360	6.11	5.44

*Table S8: **Descriptive statistics of EWE exposure across time windows.** Mean and SD of the number of EWE (nEpisodes) per participant (N = 1,451 participants) within different time windows (30–360 days) prior to measurement.*

	M2.2_30d			M2.2_60d			M2.2_90d			M2.2_120d		
Predictors	*Estimates*	*CI*	*p*	*Estimates*	*CI*	*p*	*Estimates*	*CI*	*p*	*Estimates*	*CI*	*p*
(Intercept)	0.03	-0.00 – 0.07	0.050	0.03	-0.00 – 0.07	0.073	0.03	-0.01 – 0.06	0.149	0.02	-0.02 – 0.06	0.314
polAff bin str [Dem]	0.10	0.05 – 0.14	**<0.001**	0.10	0.05 – 0.15	**<0.001**	0.11	0.06 – 0.16	**<0.001**	0.11	0.06 – 0.17	**<0.001**
nEpisodes	0.01	-0.01 – 0.03	0.509	0.00	-0.01 – 0.02	0.476	0.00	-0.00 – 0.01	0.238	0.01	-0.00 – 0.01	0.108
polAff bin str [Dem] × nEpisodes	-0.02	-0.05 – 0.01	0.210	-0.01	-0.03 – 0.01	0.176	-0.01	-0.02 – 0.00	0.059	-0.01	-0.02 – 0.00	0.071
**Random Effects**												
σ^2	0.29			0.29			0.29			0.29		
τ_{00}	0.13 $_{subject}$			0.13 $_{subject}$			0.13 $_{subject}$			0.13 $_{subject}$		
	0.00 $_{trialNum\_fixed}$			0.00 $_{trialNum\_fixed}$			0.00 $_{trialNum\_fixed}$			0.00 $_{trialNum\_fixed}$		
ICC	0.31			0.31			0.31			0.31		
N	1451 $_{subject}$			1451 $_{subject}$			1451 $_{subject}$			1451 $_{subject}$		
	25 $_{trialNum\_fixed}$			25 $_{trialNum\_fixed}$			25 $_{trialNum\_fixed}$			25 $_{trialNum\_fixed}$		
Observations	34294			34294			34294			34294		
Marginal R^2 / Conditional R^2	0.004 / 0.316			0.004 / 0.316			0.005 / 0.316			0.005 / 0.316		

	M2.2_150d			M2.2_180d			M2.2_210d			M2.2_240d		
Predictors	*Estimates*	*CI*	*p*	*Estimates*	*CI*	*p*	*Estimates*	*CI*	*p*	*Estimates*	*CI*	*p*
(Intercept)	0.01	-0.03 – 0.05	0.501	0.02	-0.02 – 0.06	0.385	0.02	-0.02 – 0.06	0.381	0.02	-0.02 – 0.06	0.350
polAff bin str [Dem]	0.12	0.06 – 0.17	**<0.001**	0.12	0.06 – 0.17	**<0.001**	0.12	0.06 – 0.17	**<0.001**	0.11	0.06 – 0.17	**<0.001**
nEpisodes	0.01	-0.00 – 0.01	0.061	0.00	-0.00 – 0.01	0.146	0.00	-0.00 – 0.01	0.180	0.00	-0.00 – 0.01	0.213
polAff bin str [Dem] × nEpisodes	-0.01	-0.02 – 0.00	0.077	-0.01	-0.02 – 0.00	0.098	-0.01	-0.02 – 0.00	0.107	-0.01	-0.02 – 0.00	0.129
**Random Effects**												
σ^2	0.29			0.29			0.29			0.29		
τ_{00}	0.13 subject			0.13 subject			0.13 subject			0.13 subject		
	0.00 trialNum_fixed			0.00 trialNum_fixed			0.00 trialNum_fixed			0.00 trialNum_fixed		
ICC	0.31			0.31			0.31			0.31		
N	1451 subject			1451 subject			1451 subject			1451 subject		
	25 trialNum_fixed			25 trialNum_fixed			25 trialNum_fixed			25 trialNum_fixed		
Observations	34294			34294			34294			34294		
Marginal R^2 / Conditional R^2	0.005 / 0.316			0.004 / 0.316			0.004 / 0.316			0.004 / 0.316		

	M2.2_270d			M2.2_300d			M2.2_330d			M2.2_360d		
Predictors	*Estimates*	*CI*	*p*	*Estimates*	*CI*	*p*	*Estimates*	*CI*	*p*	*Estimates*	*CI*	*p*
(Intercept)	0.02	-0.02 – 0.06	0.274	0.02	-0.02 – 0.07	0.241	0.02	-0.02 – 0.06	0.271	0.02	-0.02 – 0.06	0.274
polAff bin str [Dem]	0.11	0.05 – 0.17	**<0.001**	0.10	0.04 – 0.16	**0.001**	0.11	0.05 – 0.17	**<0.001**	0.11	0.05 – 0.16	**<0.001**
nEpisodes	0.00	-0.00 – 0.01	0.309	0.00	-0.00 – 0.01	0.363	0.00	-0.00 – 0.01	0.309	0.00	-0.00 – 0.01	0.307
polAff bin str [Dem] × nEpisodes	-0.01	-0.01 – 0.00	0.213	-0.00	-0.01 – 0.00	0.306	-0.00	-0.01 – 0.00	0.239	-0.00	-0.01 – 0.00	0.251
**Random Effects**												
σ^2	0.29			0.29			0.29			0.29		
τ_{00}	0.13 $_{subject}$			0.13 $_{subject}$			0.13 $_{subject}$			0.13 $_{subject}$		
	0.00 $_{trialNum\_fixed}$			0.00 $_{trialNum\_fixed}$			0.00 $_{trialNum\_fixed}$			0.00 $_{trialNum\_fixed}$		
ICC	0.31			0.31			0.31			0.31		
N	1451 $_{subject}$			1451 $_{subject}$			1451 $_{subject}$			1451 $_{subject}$		
	25 $_{trialNum\_fixed}$			25 $_{trialNum\_fixed}$			25 $_{trialNum\_fixed}$			25 $_{trialNum\_fixed}$		
Observations	34294			34294			34294			34294		
Marginal R^2 / Conditional R^2	0.004 / 0.316			0.004 / 0.316			0.004 / 0.316			0.004 / 0.316		

*Table S9: **Sensitivity analyses: Mixed-effects models predicting ΔDuration across different EWE time windows.** ΔDuration (deltaDuration) represents relative dwell time spent on carbon information compared to bonus information. Random intercepts were included for participants (subject) and trials (trialNum_fixed) (N = 1,451 participants). The models included political affiliation (polAff_bin_str, subject-level binary variable with 0 = Republican and 1 = Democrat), the number of EWEs a participant experienced (nEpisodes) and their interaction. Models are calculated for different time windows (60-360 days). Estimates represent unstandardized beta coefficients. Confidence intervals are 95%. Statistically significant effects (p < .05) are shown in bold.*

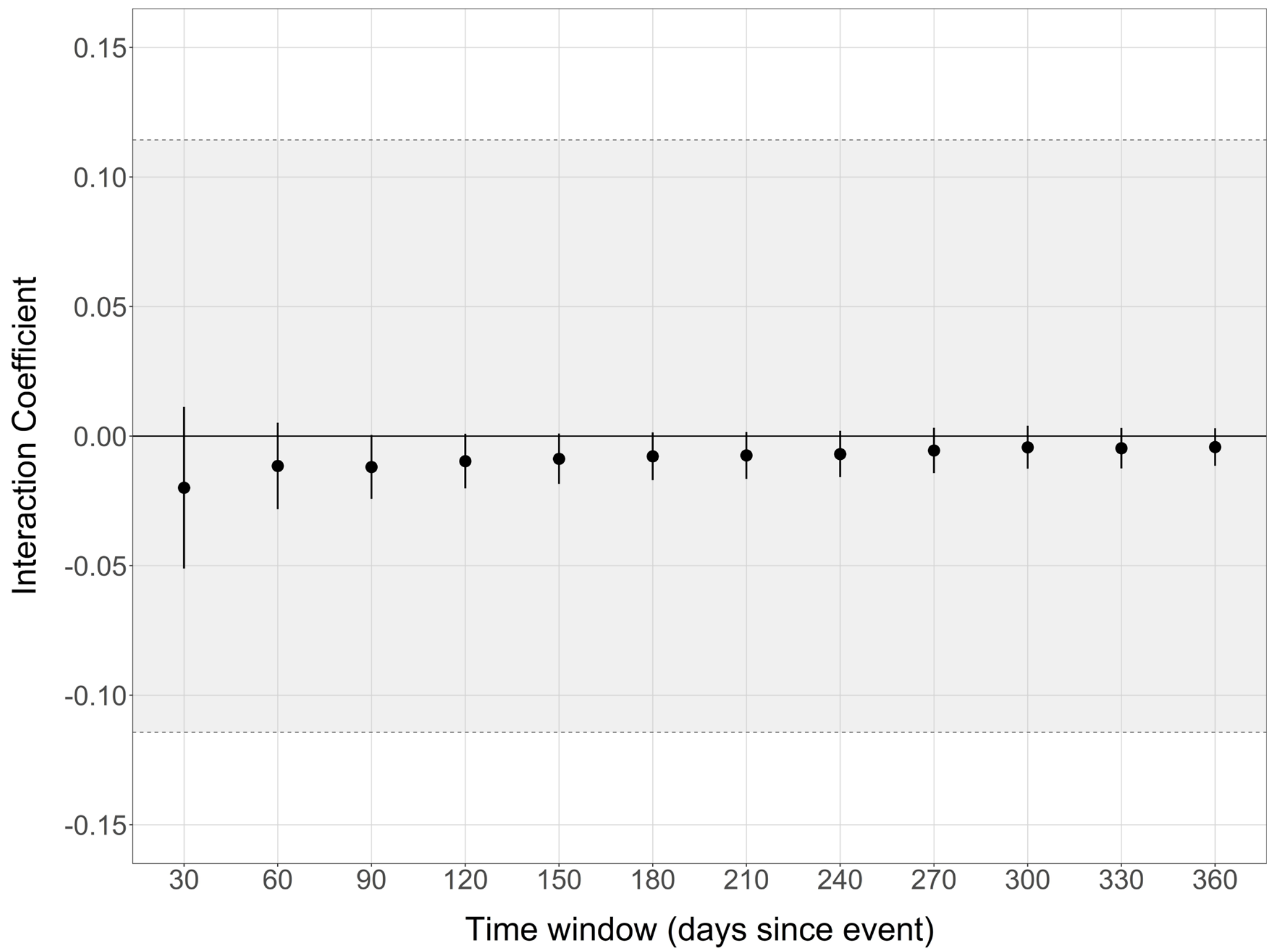

Figure S6: ***Equivalence testing of interaction coefficients across different EWE time windows****: Points represent estimated interaction coefficients for the interaction between political affiliation and the number of EWEs a participant experienced predicting ΔDuration across models using different EWE time windows (30–360 days preceding measurement; N = 1,451 participants). Vertical lines indicate 95% confidence intervals. The shaded area denotes the region of practical equivalence (ROPE; ±0.1143), based on the smallest effect size of interest (SESOI).*

We additionally repeated the sensitivity analyses separately for each EWE type. Across all event types and time windows, the estimated interaction effects were small and statistically non significant (Table S10), with confidence intervals largely falling within the ROPE (±0.1143), as shown in Figure S7.

	M2.2_Coastal Flood_30d			M2.2_Drought_30d			M2.2_Excessive Heat_30d			M2.2_Flash Flood_30d		
Predictors	*Estimates*	*CI*	*p*	*Estimates*	*CI*	*p*	*Estimates*	*CI*	*p*	*Estimates*	*CI*	*p*
(Intercept)	0.04	0.01 – 0.07	**0.004**	0.04	0.01 – 0.07	**0.004**	0.04	0.01 – 0.06	**0.010**	0.04	0.01 – 0.07	**0.004**
polAff bin str [Dem]	0.08	0.04 – 0.12	**<0.001**	0.08	0.04 – 0.12	**<0.001**	0.09	0.05 – 0.13	**<0.001**	0.08	0.04 – 0.12	**<0.001**
nEvents	-0.42	-1.16 – 0.33	0.275	-0.01	-0.23 – 0.20	0.893	0.01	-0.03 – 0.06	0.548	-0.01	-0.04 – 0.02	0.566
polAff bin str [Dem] × nEvents	0.64	-0.22 – 1.50	0.146	-0.02	-0.34 – 0.30	0.919	-0.04	-0.10 – 0.03	0.288	-0.00	-0.05 – 0.05	0.949
**Random Effects**												
σ^2	0.29			0.29			0.29			0.29		
τ_{00}	0.13 $_{subject}$			0.13 $_{subject}$			0.13 $_{subject}$			0.13 $_{subject}$		
	0.00 $_{trialNum\_fixed}$			0.00 $_{trialNum\_fixed}$			0.00 $_{trialNum\_fixed}$			0.00 $_{trialNum\_fixed}$		
ICC	0.31			0.31			0.31			0.31		
N	1451 $_{subject}$			1451 $_{subject}$			1451 $_{subject}$			1451 $_{subject}$		
	25 $_{trialNum\_fixed}$			25 $_{trialNum\_fixed}$			25 $_{trialNum\_fixed}$			25 $_{trialNum\_fixed}$		
Observations	34294			34294			34294			34294		
Marginal R^2 / Conditional R^2	0.004 / 0.316			0.004 / 0.316			0.004 / 0.316			0.004 / 0.316		

	M2.2_Hail_30d			M2.2_Strong Wind_30d			M2.2_Tornado_30d			M2.2_Wildfire_30d		
Predictors	*Estimates*	*CI*	*p*	*Estimates*	*CI*	*p*	*Estimates*	*CI*	*p*	*Estimates*	*CI*	*p*
(Intercept)	0.04	0.01 – 0.07	**0.008**	0.04	0.01 – 0.07	**0.004**	0.04	0.01 – 0.07	**0.004**	0.04	0.01 – 0.07	**0.007**
polAff bin str [Dem]	0.08	0.04 – 0.12	**<0.001**	0.08	0.04 – 0.12	**<0.001**	0.08	0.04 – 0.12	**<0.001**	0.08	0.04 – 0.12	**<0.001**
nEvents	0.01	-0.07 – 0.10	0.764	0.02	-0.51 – 0.55	0.941	0.07	-0.46 – 0.60	0.793	0.01	-0.05 – 0.08	0.725
polAff bin str [Dem] × nEvents	-0.01	-0.13 – 0.11	0.847	-0.10	-0.85 – 0.65	0.794	-0.03	-0.94 – 0.88	0.952	-0.05	-0.16 – 0.06	0.342
**Random Effects**												
σ^2	0.29			0.29			0.29			0.29		
τ_{00}	0.13 $_{subject}$			0.13 $_{subject}$			0.13 $_{subject}$			0.13 $_{subject}$		
	0.00 $_{trialNum\_fixed}$			0.00 $_{trialNum\_fixed}$			0.00 $_{trialNum\_fixed}$			0.00 $_{trialNum\_fixed}$		
ICC	0.31			0.31			0.31			0.31		
N	1451 $_{subject}$			1451 $_{subject}$			1451 $_{subject}$			1451 $_{subject}$		
	25 $_{trialNum\_fixed}$			25 $_{trialNum\_fixed}$			25 $_{trialNum\_fixed}$			25 $_{trialNum\_fixed}$		
Observations	34294			34294			34294			34294		
Marginal R^2 / Conditional R^2	0.004 / 0.316			0.004 / 0.316			0.004 / 0.316			0.004 / 0.316		

	M2.2_Coastal Flood_60d			M2.2_Drought_60d			M2.2_Excessive Heat_60d			M2.2_Flash Flood_60d		
Predictors	*Estimates*	*CI*	*p*	*Estimates*	*CI*	*p*	*Estimates*	*CI*	*p*	*Estimates*	*CI*	*p*
(Intercept)	0.04	0.01 – 0.07	**0.004**	0.04	0.01 – 0.06	**0.010**	0.04	0.01 – 0.07	**0.011**	0.04	0.01 – 0.07	**0.010**
polAff bin str [Dem]	0.08	0.04 – 0.12	**<0.001**	0.08	0.04 – 0.12	**<0.001**	0.09	0.05 – 0.13	**<0.001**	0.08	0.04 – 0.13	**<0.001**
nEvents	-0.42	-1.16 – 0.33	0.275	0.05	-0.02 – 0.12	0.162	0.01	-0.02 – 0.03	0.568	-0.00	-0.02 – 0.02	0.842
polAff bin str [Dem] × nEvents	0.64	-0.22 – 1.50	0.146	-0.10	-0.24 – 0.04	0.169	-0.02	-0.06 – 0.01	0.234	-0.00	-0.03 – 0.02	0.826
**Random Effects**												
σ^2	0.29			0.29			0.29			0.29		
τ_{00}	0.13 $_{subject}$			0.13 $_{subject}$			0.13 $_{subject}$			0.13 $_{subject}$		
	0.00 $_{trialNum\_fixed}$			0.00 $_{trialNum\_fixed}$			0.00 $_{trialNum\_fixed}$			0.00 $_{trialNum\_fixed}$		
ICC	0.31			0.31			0.31			0.31		
N	1451 $_{subject}$			1451 $_{subject}$			1451 $_{subject}$			1451 $_{subject}$		
	25 $_{trialNum\_fixed}$			25 $_{trialNum\_fixed}$			25 $_{trialNum\_fixed}$			25 $_{trialNum\_fixed}$		
Observations	34294			34294			34294			34294		
Marginal R^2 / Conditional R^2	0.004 / 0.316			0.004 / 0.316			0.004 / 0.316			0.004 / 0.316		

	M2.2_Hail_60d			M2.2_Strong Wind_60d			M2.2_Tornado_60d			M2.2_Wildfire_60d		
Predictors	*Estimates*	*CI*	*p*	*Estimates*	*CI*	*p*	*Estimates*	*CI*	*p*	*Estimates*	*CI*	*p*
(Intercept)	0.04	0.01 – 0.07	**0.010**	0.04	0.01 – 0.07	**0.004**	0.04	0.01 – 0.07	**0.006**	0.04	0.01 – 0.07	**0.005**
polAff bin str [Dem]	0.09	0.04 – 0.13	**<0.001**	0.08	0.04 – 0.12	**<0.001**	0.08	0.04 – 0.12	**<0.001**	0.08	0.04 – 0.12	**<0.001**
nEvents	0.01	-0.02 – 0.04	0.648	0.01	-0.42 – 0.45	0.949	0.06	-0.15 – 0.27	0.578	-0.00	-0.06 – 0.05	0.906
polAff bin str [Dem] × nEvents	-0.02	-0.07 – 0.03	0.398	-0.09	-0.78 – 0.59	0.788	0.01	-0.25 – 0.28	0.921	-0.02	-0.10 – 0.06	0.551
**Random Effects**												
σ^2	0.29			0.29			0.29			0.29		
τ_{00}	0.13 $_{subject}$			0.13 $_{subject}$			0.13 $_{subject}$			0.13 $_{subject}$		
	0.00 $_{trialNum\_fixed}$			0.00 $_{trialNum\_fixed}$			0.00 $_{trialNum\_fixed}$			0.00 $_{trialNum\_fixed}$		
ICC	0.31			0.31			0.31			0.31		
N	1451 $_{subject}$			1451 $_{subject}$			1451 $_{subject}$			1451 $_{subject}$		
	25 $_{trialNum\_fixed}$			25 $_{trialNum\_fixed}$			25 $_{trialNum\_fixed}$			25 $_{trialNum\_fixed}$		
Observations	34294			34294			34294			34294		
Marginal R^2 / Conditional R^2	0.004 / 0.316			0.004 / 0.316			0.004 / 0.316			0.004 / 0.316		

	M2.2_Coastal Flood_90d			M2.2_Drought_90d			M2.2_Excessive Heat_90d			M2.2_Flash Flood_90d		
Predictors	*Estimates*	*CI*	*p*	*Estimates*	*CI*	*p*	*Estimates*	*CI*	*p*	*Estimates*	*CI*	*p*
(Intercept)	0.04	0.01 – 0.07	**0.004**	0.03	0.01 – 0.06	**0.018**	0.04	0.01 – 0.06	**0.012**	0.04	0.01 – 0.07	**0.013**
polAff bin str [Dem]	0.08	0.04 – 0.12	**<0.001**	0.09	0.05 – 0.13	**<0.001**	0.09	0.05 – 0.13	**<0.001**	0.09	0.04 – 0.13	**<0.001**
nEvents	-0.42	-1.16 – 0.33	0.275	0.02	-0.01 – 0.05	0.155	0.01	-0.01 – 0.02	0.535	-0.00	-0.02 – 0.01	0.884
polAff bin str [Dem] × nEvents	0.44	-0.37 – 1.25	0.286	-0.03	-0.08 – 0.01	0.164	-0.02	-0.05 – 0.01	0.126	-0.01	-0.03 – 0.02	0.575
**Random Effects**												
σ^2	0.29			0.29			0.29			0.29		
τ_{00}	0.13 $_{subject}$			0.13 $_{subject}$			0.13 $_{subject}$			0.13 $_{subject}$		
	0.00 $_{trialNum\_fixed}$			0.00 $_{trialNum\_fixed}$			0.00 $_{trialNum\_fixed}$			0.00 $_{trialNum\_fixed}$		
ICC	0.31			0.31			0.31			0.31		
N	1451 $_{subject}$			1451 $_{subject}$			1451 $_{subject}$			1451 $_{subject}$		
	25 $_{trialNum\_fixed}$			25 $_{trialNum\_fixed}$			25 $_{trialNum\_fixed}$			25 $_{trialNum\_fixed}$		
Observations	34294			34294			34294			34294		
Marginal R^2 / Conditional R^2	0.004 / 0.316			0.004 / 0.316			0.004 / 0.316			0.004 / 0.316		

	M2.2_Hail_90d			M2.2_Strong Wind_90d			M2.2_Tornado_90d			M2.2_Wildfire_90d		
Predictors	*Estimates*	*CI*	*p*	*Estimates*	*CI*	*p*	*Estimates*	*CI*	*p*	*Estimates*	*CI*	*p*
(Intercept)	0.03	0.00 – 0.06	**0.024**	0.04	0.01 – 0.06	**0.006**	0.03	0.00 – 0.06	**0.032**	0.04	0.01 – 0.07	**0.010**
polAff bin str [Dem]	0.09	0.05 – 0.13	**<0.001**	0.08	0.04 – 0.12	**<0.001**	0.08	0.04 – 0.12	**<0.001**	0.08	0.04 – 0.13	**<0.001**
nEvents	0.01	-0.01 – 0.03	0.217	0.10	-0.08 – 0.28	0.282	0.18	0.06 – 0.29	**0.002**	0.01	-0.04 – 0.06	0.602
polAff bin str [Dem] × nEvents	-0.02	-0.05 – 0.01	0.171	-0.21	-0.52 – 0.10	0.188	-0.07	-0.22 – 0.09	0.377	-0.03	-0.11 – 0.04	0.369
**Random Effects**												
σ^2	0.29			0.29			0.29			0.29		
τ_{00}	0.13 $_{subject}$			0.13 $_{subject}$			0.13 $_{subject}$			0.13 $_{subject}$		
	0.00 $_{trialNum\_fixed}$			0.00 $_{trialNum\_fixed}$			0.00 $_{trialNum\_fixed}$			0.00 $_{trialNum\_fixed}$		
ICC	0.31			0.31			0.31			0.31		
N	1451 $_{subject}$			1451 $_{subject}$			1451 $_{subject}$			1451 $_{subject}$		
	25 $_{trialNum\_fixed}$			25 $_{trialNum\_fixed}$			25 $_{trialNum\_fixed}$			25 $_{trialNum\_fixed}$		
Observations	34294			34294			34294			34294		
Marginal R^2 / Conditional R^2	0.004 / 0.316			0.004 / 0.316			0.007 / 0.316			0.004 / 0.316		

	M2.2_Coastal Flood_120d			M2.2_Drought_120d			M2.2_Excessive Heat_120d			M2.2_Flash Flood_120d		
Predictors	*Estimates*	*CI*	*p*	*Estimates*	*CI*	*p*	*Estimates*	*CI*	*p*	*Estimates*	*CI*	*p*
(Intercept)	0.04	0.01 – 0.07	**0.005**	0.04	0.01 – 0.07	**0.010**	0.04	0.01 – 0.06	**0.012**	0.04	0.01 – 0.07	**0.019**
polAff bin str [Dem]	0.08	0.04 – 0.12	**<0.001**	0.08	0.04 – 0.12	**<0.001**	0.09	0.05 – 0.13	**<0.001**	0.09	0.04 – 0.14	**<0.001**
nEvents	0.07	-0.36 – 0.50	0.749	0.01	-0.01 – 0.02	0.601	0.01	-0.01 – 0.02	0.548	0.00	-0.01 – 0.01	0.978
polAff bin str [Dem] × nEvents	0.05	-0.42 – 0.51	0.850	-0.01	-0.05 – 0.02	0.480	-0.02	-0.05 – 0.01	0.128	-0.01	-0.03 – 0.01	0.514
**Random Effects**												
σ^2	0.29			0.29			0.29			0.29		
τ_{00}	0.13 $_{subject}$			0.13 $_{subject}$			0.13 $_{subject}$			0.13 $_{subject}$		
	0.00 $_{trialNum\_fixed}$			0.00 $_{trialNum\_fixed}$			0.00 $_{trialNum\_fixed}$			0.00 $_{trialNum\_fixed}$		
ICC	0.31			0.31			0.31			0.31		
N	1451 $_{subject}$			1451 $_{subject}$			1451 $_{subject}$			1451 $_{subject}$		
	25 $_{trialNum\_fixed}$			25 $_{trialNum\_fixed}$			25 $_{trialNum\_fixed}$			25 $_{trialNum\_fixed}$		
Observations	34294			34294			34294			34294		
Marginal R^2 / Conditional R^2	0.004 / 0.316			0.004 / 0.316			0.004 / 0.316			0.004 / 0.316		

	M2.2_Hail_120d			M2.2_Strong Wind_120d			M2.2_Tornado_120d			M2.2_Wildfire_120d		
Predictors	*Estimates*	*CI*	*p*	*Estimates*	*CI*	*p*	*Estimates*	*CI*	*p*	*Estimates*	*CI*	*p*
(Intercept)	0.02	-0.01 – 0.05	0.128	0.04	0.01 – 0.07	**0.005**	0.03	0.00 – 0.06	**0.046**	0.04	0.01 – 0.07	**0.009**
polAff bin str [Dem]	0.10	0.05 – 0.14	**<0.001**	0.08	0.04 – 0.12	**<0.001**	0.08	0.04 – 0.12	**<0.001**	0.08	0.04 – 0.12	**<0.001**
nEvents	0.02	0.00 – 0.03	**0.046**	-0.00	-0.12 – 0.11	0.969	0.09	0.02 – 0.17	**0.012**	0.01	-0.04 – 0.06	0.760
polAff bin str [Dem] × nEvents	-0.02	-0.04 – 0.01	0.143	-0.08	-0.24 – 0.09	0.360	-0.03	-0.13 – 0.08	0.598	-0.03	-0.10 – 0.05	0.480
**Random Effects**												
σ^2	0.29			0.29			0.29			0.29		
τ_{00}	0.13 $_{subject}$			0.13 $_{subject}$			0.13 $_{subject}$			0.13 $_{subject}$		
	0.00 $_{trialNum\_fixed}$			0.00 $_{trialNum\_fixed}$			0.00 $_{trialNum\_fixed}$			0.00 $_{trialNum\_fixed}$		
ICC	0.31			0.31			0.31			0.31		
N	1451 $_{subject}$			1451 $_{subject}$			1451 $_{subject}$			1451 $_{subject}$		
	25 $_{trialNum\_fixed}$			25 $_{trialNum\_fixed}$			25 $_{trialNum\_fixed}$			25 $_{trialNum\_fixed}$		
Observations	34294			34294			34294			34294		
Marginal R^2 / Conditional R^2	0.005 / 0.316			0.004 / 0.316			0.006 / 0.316			0.004 / 0.316		

	M2.2_Coastal Flood_150d			M2.2_Drought_150d			M2.2_Excessive Heat_150d			M2.2_Flash Flood_150d		
Predictors	*Estimates*	*CI*	*p*	*Estimates*	*CI*	*p*	*Estimates*	*CI*	*p*	*Estimates*	*CI*	*p*
(Intercept)	0.04	0.01 – 0.07	**0.004**	0.04	0.01 – 0.07	**0.010**	0.04	0.01 – 0.06	**0.012**	0.04	0.00 – 0.07	**0.030**
polAff bin str [Dem]	0.08	0.04 – 0.12	**<0.001**	0.08	0.04 – 0.12	**<0.001**	0.09	0.05 – 0.13	**<0.001**	0.09	0.04 – 0.14	**<0.001**
nEvents	0.02	-0.35 – 0.39	0.917	0.00	-0.01 – 0.02	0.581	0.01	-0.01 – 0.02	0.548	0.00	-0.01 – 0.02	0.869
polAff bin str [Dem] × nEvents	0.09	-0.33 – 0.50	0.686	-0.01	-0.04 – 0.02	0.434	-0.02	-0.05 – 0.01	0.128	-0.01	-0.03 – 0.01	0.558
**Random Effects**												
σ^2	0.29			0.29			0.29			0.29		
τ_{00}	0.13 $_{subject}$			0.13 $_{subject}$			0.13 $_{subject}$			0.13 $_{subject}$		
	0.00 $_{trialNum\_fixed}$			0.00 $_{trialNum\_fixed}$			0.00 $_{trialNum\_fixed}$			0.00 $_{trialNum\_fixed}$		
ICC	0.31			0.31			0.31			0.31		
N	1451 $_{subject}$			1451 $_{subject}$			1451 $_{subject}$			1451 $_{subject}$		
	25 $_{trialNum\_fixed}$			25 $_{trialNum\_fixed}$			25 $_{trialNum\_fixed}$			25 $_{trialNum\_fixed}$		
Observations	34294			34294			34294			34294		
Marginal R^2 / Conditional R^2	0.004 / 0.316			0.004 / 0.316			0.004 / 0.316			0.004 / 0.316		

	M2.2_Hail_150d			M2.2_Strong Wind_150d			M2.2_Tornado_150d			M2.2_Wildfire_150d		
Predictors	*Estimates*	*CI*	*p*	*Estimates*	*CI*	*p*	*Estimates*	*CI*	*p*	*Estimates*	*CI*	*p*
(Intercept)	0.02	-0.01 – 0.05	0.236	0.04	0.01 – 0.07	**0.006**	0.03	0.00 – 0.06	**0.043**	0.04	0.01 – 0.07	**0.006**
polAff bin str [Dem]	0.10	0.05 – 0.14	**<0.001**	0.08	0.04 – 0.12	**<0.001**	0.08	0.04 – 0.12	**<0.001**	0.08	0.04 – 0.12	**<0.001**
nEvents	0.02	0.00 – 0.03	**0.019**	-0.00	-0.09 – 0.08	0.932	0.05	-0.00 – 0.11	0.063	-0.00	-0.04 – 0.03	0.917
polAff bin str [Dem] × nEvents	-0.01	-0.03 – 0.01	0.175	-0.04	-0.15 – 0.08	0.548	-0.01	-0.09 – 0.07	0.821	-0.01	-0.07 – 0.04	0.600
**Random Effects**												
σ^2	0.29			0.29			0.29			0.29		
τ_{00}	0.13 $_{subject}$			0.13 $_{subject}$			0.13 $_{subject}$			0.13 $_{subject}$		
	0.00 $_{trialNum\_fixed}$			0.00 $_{trialNum\_fixed}$			0.00 $_{trialNum\_fixed}$			0.00 $_{trialNum\_fixed}$		
ICC	0.31			0.31			0.31			0.31		
N	1451 $_{subject}$			1451 $_{subject}$			1451 $_{subject}$			1451 $_{subject}$		
	25 $_{trialNum\_fixed}$			25 $_{trialNum\_fixed}$			25 $_{trialNum\_fixed}$			25 $_{trialNum\_fixed}$		
Observations	34294			34294			34294			34294		
Marginal R^2 / Conditional R^2	0.005 / 0.316			0.004 / 0.316			0.005 / 0.316			0.004 / 0.316		

	M2.2_Coastal Flood_180d			M2.2_Drought_180d			M2.2_Excessive Heat_180d			M2.2_Flash Flood_180d		
Predictors	*Estimates*	*CI*	*p*	*Estimates*	*CI*	*p*	*Estimates*	*CI*	*p*	*Estimates*	*CI*	*p*
(Intercept)	0.04	0.01 – 0.07	**0.004**	0.04	0.01 – 0.07	**0.009**	0.04	0.01 – 0.06	**0.012**	0.04	0.00 – 0.07	**0.027**
polAff bin str [Dem]	0.08	0.04 – 0.12	**<0.001**	0.08	0.04 – 0.12	**<0.001**	0.09	0.05 – 0.13	**<0.001**	0.09	0.04 – 0.14	**0.001**
nEvents	0.02	-0.35 – 0.39	0.917	0.00	-0.01 – 0.02	0.710	0.01	-0.01 – 0.02	0.548	-0.00	-0.01 – 0.01	0.969
polAff bin str [Dem] × nEvents	0.09	-0.33 – 0.50	0.686	-0.01	-0.04 – 0.02	0.461	-0.02	-0.05 – 0.01	0.128	-0.01	-0.03 – 0.01	0.543
**Random Effects**												
σ^2	0.29			0.29			0.29			0.29		
τ_{00}	0.13 $_{subject}$			0.13 $_{subject}$			0.13 $_{subject}$			0.13 $_{subject}$		
	0.00 $_{trialNum\_fixed}$			0.00 $_{trialNum\_fixed}$			0.00 $_{trialNum\_fixed}$			0.00 $_{trialNum\_fixed}$		
ICC	0.31			0.31			0.31			0.31		
N	1451 $_{subject}$			1451 $_{subject}$			1451 $_{subject}$			1451 $_{subject}$		
	25 $_{trialNum\_fixed}$			25 $_{trialNum\_fixed}$			25 $_{trialNum\_fixed}$			25 $_{trialNum\_fixed}$		
Observations	34294			34294			34294			34294		
Marginal R^2 / Conditional R^2	0.004 / 0.316			0.004 / 0.316			0.004 / 0.316			0.004 / 0.316		

	M2.2_Hail_180d			M2.2_Strong Wind_180d			M2.2_Tornado_180d			M2.2_Wildfire_180d		
Predictors	*Estimates*	*CI*	*p*	*Estimates*	*CI*	*p*	*Estimates*	*CI*	*p*	*Estimates*	*CI*	*p*
(Intercept)	0.02	-0.01 – 0.05	0.219	0.04	0.01 – 0.07	**0.004**	0.02	-0.01 – 0.06	0.135	0.04	0.01 – 0.07	**0.006**
polAff bin str [Dem]	0.10	0.05 – 0.14	**<0.001**	0.08	0.04 – 0.13	**<0.001**	0.09	0.04 – 0.14	**<0.001**	0.08	0.04 – 0.12	**<0.001**
nEvents	0.01	0.00 – 0.03	**0.036**	-0.02	-0.07 – 0.03	0.509	0.04	-0.00 – 0.09	0.066	-0.00	-0.04 – 0.03	0.845
polAff bin str [Dem] × nEvents	-0.01	-0.03 – 0.01	0.209	-0.02	-0.09 – 0.05	0.567	-0.03	-0.09 – 0.04	0.430	-0.01	-0.06 – 0.04	0.709
**Random Effects**												
σ^2	0.29			0.29			0.29			0.29		
τ_{00}	0.13 $_{subject}$			0.13 $_{subject}$			0.13 $_{subject}$			0.13 $_{subject}$		
	0.00 $_{trialNum\_fixed}$			0.00 $_{trialNum\_fixed}$			0.00 $_{trialNum\_fixed}$			0.00 $_{trialNum\_fixed}$		
ICC	0.31			0.31			0.31			0.31		
N	1451 $_{subject}$			1451 $_{subject}$			1451 $_{subject}$			1451 $_{subject}$		
	25 $_{trialNum\_fixed}$			25 $_{trialNum\_fixed}$			25 $_{trialNum\_fixed}$			25 $_{trialNum\_fixed}$		
Observations	34294			34294			34294			34294		
Marginal R^2 / Conditional R^2	0.005 / 0.316			0.004 / 0.316			0.005 / 0.316			0.004 / 0.316		

	M2.2_Coastal Flood_210d			M2.2_Drought_210d			M2.2_Excessive Heat_210d			M2.2_Flash Flood_210d		
Predictors	*Estimates*	*CI*	*p*	*Estimates*	*CI*	*p*	*Estimates*	*CI*	*p*	*Estimates*	*CI*	*p*
(Intercept)	0.04	0.01 – 0.07	**0.004**	0.04	0.01 – 0.07	**0.009**	0.04	0.01 – 0.06	**0.012**	0.04	0.00 – 0.07	**0.031**
polAff bin str [Dem]	0.08	0.04 – 0.12	**<0.001**	0.08	0.04 – 0.12	**<0.001**	0.09	0.05 – 0.13	**<0.001**	0.09	0.04 – 0.14	**0.001**
nEvents	0.02	-0.35 – 0.39	0.917	0.00	-0.01 – 0.02	0.718	0.01	-0.01 – 0.02	0.548	0.00	-0.01 – 0.01	0.996
polAff bin str [Dem] × nEvents	0.09	-0.33 – 0.50	0.686	-0.01	-0.04 – 0.01	0.430	-0.02	-0.05 – 0.01	0.128	-0.01	-0.03 – 0.01	0.543
**Random Effects**												
σ^2	0.29			0.29			0.29			0.29		
τ_{00}	0.13 $_{subject}$			0.13 $_{subject}$			0.13 $_{subject}$			0.13 $_{subject}$		
	0.00 $_{trialNum\_fixed}$			0.00 $_{trialNum\_fixed}$			0.00 $_{trialNum\_fixed}$			0.00 $_{trialNum\_fixed}$		
ICC	0.31			0.31			0.31			0.31		
N	1451 $_{subject}$			1451 $_{subject}$			1451 $_{subject}$			1451 $_{subject}$		
	25 $_{trialNum\_fixed}$			25 $_{trialNum\_fixed}$			25 $_{trialNum\_fixed}$			25 $_{trialNum\_fixed}$		
Observations	34294			34294			34294			34294		
Marginal R^2 / Conditional R^2	0.004 / 0.316			0.004 / 0.316			0.004 / 0.316			0.004 / 0.316		

	M2.2_Hail_210d			M2.2_Strong Wind_210d			M2.2_Tornado_210d			M2.2_Wildfire_210d		
Predictors	*Estimates*	*CI*	*p*	*Estimates*	*CI*	*p*	*Estimates*	*CI*	*p*	*Estimates*	*CI*	*p*
(Intercept)	0.02	-0.01 – 0.05	0.215	0.05	0.02 – 0.08	**0.002**	0.02	-0.01 – 0.05	0.160	0.04	0.01 – 0.07	**0.008**
polAff bin str [Dem]	0.10	0.05 – 0.14	**<0.001**	0.08	0.04 – 0.12	**<0.001**	0.09	0.05 – 0.14	**<0.001**	0.08	0.04 – 0.13	**<0.001**
nEvents	0.01	0.00 – 0.03	**0.039**	-0.03	-0.07 – 0.02	0.211	0.05	0.00 – 0.09	**0.044**	0.00	-0.03 – 0.03	0.940
polAff bin str [Dem] × nEvents	-0.01	-0.03 – 0.01	0.207	0.01	-0.05 – 0.06	0.868	-0.03	-0.09 – 0.04	0.376	-0.02	-0.07 – 0.03	0.478
**Random Effects**												
σ^2	0.29			0.29			0.29			0.29		
τ_{00}	0.13 $_{subject}$			0.13 $_{subject}$			0.13 $_{subject}$			0.13 $_{subject}$		
	0.00 $_{trialNum\_fixed}$			0.00 $_{trialNum\_fixed}$			0.00 $_{trialNum\_fixed}$			0.00 $_{trialNum\_fixed}$		
ICC	0.31			0.31			0.31			0.31		
N	1451 $_{subject}$			1451 $_{subject}$			1451 $_{subject}$			1451 $_{subject}$		
	25 $_{trialNum\_fixed}$			25 $_{trialNum\_fixed}$			25 $_{trialNum\_fixed}$			25 $_{trialNum\_fixed}$		
Observations	34294			34294			34294			34294		
Marginal R^2 / Conditional R^2	0.005 / 0.316			0.004 / 0.316			0.005 / 0.316			0.004 / 0.316		

	M2.2_Coastal Flood_240d			M2.2_Drought_240d			M2.2_Excessive Heat_240d			M2.2_Flash Flood_240d		
Predictors	*Estimates*	*CI*	*p*	*Estimates*	*CI*	*p*	*Estimates*	*CI*	*p*	*Estimates*	*CI*	*p*
(Intercept)	0.04	0.01 – 0.07	**0.004**	0.04	0.01 – 0.07	**0.006**	0.04	0.01 – 0.06	**0.012**	0.04	0.00 – 0.07	**0.036**
polAff bin str [Dem]	0.08	0.04 – 0.12	**<0.001**	0.08	0.04 – 0.12	**<0.001**	0.09	0.05 – 0.13	**<0.001**	0.09	0.04 – 0.14	**<0.001**
nEvents	0.02	-0.35 – 0.39	0.917	-0.00	-0.01 – 0.01	0.973	0.01	-0.01 – 0.02	0.542	0.00	-0.01 – 0.01	0.917
polAff bin str [Dem] × nEvents	0.09	-0.33 – 0.50	0.686	-0.01	-0.03 – 0.02	0.581	-0.02	-0.05 – 0.01	0.127	-0.01	-0.03 – 0.01	0.509
**Random Effects**												
σ^2	0.29			0.29			0.29			0.29		
τ_{00}	0.13 $_{subject}$			0.13 $_{subject}$			0.13 $_{subject}$			0.13 $_{subject}$		
	0.00 $_{trialNum\_fixed}$			0.00 $_{trialNum\_fixed}$			0.00 $_{trialNum\_fixed}$			0.00 $_{trialNum\_fixed}$		
ICC	0.31			0.31			0.31			0.31		
N	1451 $_{subject}$			1451 $_{subject}$			1451 $_{subject}$			1451 $_{subject}$		
	25 $_{trialNum\_fixed}$			25 $_{trialNum\_fixed}$			25 $_{trialNum\_fixed}$			25 $_{trialNum\_fixed}$		
Observations	34294			34294			34294			34294		
Marginal R^2 / Conditional R^2	0.004 / 0.316			0.004 / 0.316			0.004 / 0.316			0.004 / 0.316		

	M2.2_Hail_240d			M2.2_Strong Wind_240d			M2.2_Tornado_240d			M2.2_Wildfire_240d		
Predictors	*Estimates*	*CI*	*p*	*Estimates*	*CI*	*p*	*Estimates*	*CI*	*p*	*Estimates*	*CI*	*p*
(Intercept)	0.02	-0.01 – 0.05	0.212	0.05	0.02 – 0.07	**0.002**	0.02	-0.01 – 0.05	0.207	0.04	0.01 – 0.07	**0.007**
polAff bin str [Dem]	0.10	0.05 – 0.14	**<0.001**	0.08	0.04 – 0.12	**<0.001**	0.09	0.05 – 0.14	**<0.001**	0.08	0.04 – 0.12	**<0.001**
nEvents	0.01	0.00 – 0.02	**0.042**	-0.02	-0.06 – 0.01	0.226	0.05	0.00 – 0.09	**0.032**	-0.00	-0.03 – 0.03	0.900
polAff bin str [Dem] × nEvents	-0.01	-0.03 – 0.01	0.229	0.01	-0.05 – 0.06	0.797	-0.03	-0.09 – 0.03	0.348	-0.01	-0.06 – 0.04	0.623
**Random Effects**												
σ^2	0.29			0.29			0.29			0.29		
τ_{00}	0.13 $_{subject}$			0.13 $_{subject}$			0.13 $_{subject}$			0.13 $_{subject}$		
	0.00 $_{trialNum\_fixed}$			0.00 $_{trialNum\_fixed}$			0.00 $_{trialNum\_fixed}$			0.00 $_{trialNum\_fixed}$		
ICC	0.31			0.31			0.31			0.31		
N	1451 $_{subject}$			1451 $_{subject}$			1451 $_{subject}$			1451 $_{subject}$		
	25 $_{trialNum\_fixed}$			25 $_{trialNum\_fixed}$			25 $_{trialNum\_fixed}$			25 $_{trialNum\_fixed}$		
Observations	34294			34294			34294			34294		
Marginal R^2 / Conditional R^2	0.005 / 0.316			0.004 / 0.316			0.005 / 0.316			0.004 / 0.316		

	M2.2_Coastal Flood_270d			M2.2_Drought_270d			M2.2_Excessive Heat_270d			M2.2_Flash Flood_270d		
Predictors	*Estimates*	*CI*	*p*	*Estimates*	*CI*	*p*	*Estimates*	*CI*	*p*	*Estimates*	*CI*	*p*
(Intercept)	0.04	0.01 – 0.07	**0.004**	0.04	0.01 – 0.07	**0.006**	0.04	0.01 – 0.06	**0.012**	0.04	0.00 – 0.07	**0.032**
polAff bin str [Dem]	0.08	0.04 – 0.12	**<0.001**	0.08	0.04 – 0.12	**<0.001**	0.09	0.05 – 0.13	**<0.001**	0.09	0.04 – 0.14	**<0.001**
nEvents	0.02	-0.35 – 0.39	0.917	-0.00	-0.01 – 0.01	0.893	0.01	-0.01 – 0.02	0.542	0.00	-0.01 – 0.01	0.946
polAff bin str [Dem] × nEvents	0.09	-0.33 – 0.50	0.686	-0.00	-0.03 – 0.02	0.722	-0.02	-0.05 – 0.01	0.127	-0.01	-0.02 – 0.01	0.532
**Random Effects**												
σ^2	0.29			0.29			0.29			0.29		
τ_{00}	0.13 $_{subject}$			0.13 $_{subject}$			0.13 $_{subject}$			0.13 $_{subject}$		
	0.00 $_{trialNum\_fixed}$			0.00 $_{trialNum\_fixed}$			0.00 $_{trialNum\_fixed}$			0.00 $_{trialNum\_fixed}$		
ICC	0.31			0.31			0.31			0.31		
N	1451 $_{subject}$			1451 $_{subject}$			1451 $_{subject}$			1451 $_{subject}$		
	25 $_{trialNum\_fixed}$			25 $_{trialNum\_fixed}$			25 $_{trialNum\_fixed}$			25 $_{trialNum\_fixed}$		
Observations	34294			34294			34294			34294		
Marginal R^2 / Conditional R^2	0.004 / 0.316			0.004 / 0.316			0.004 / 0.316			0.004 / 0.316		

	M2.2_Hail_270d			M2.2_Strong Wind_270d			M2.2_Tornado_270d			M2.2_Wildfire_270d		
Predictors	*Estimates*	*CI*	*p*	*Estimates*	*CI*	*p*	*Estimates*	*CI*	*p*	*Estimates*	*CI*	*p*
(Intercept)	0.02	-0.01 – 0.05	0.223	0.05	0.02 – 0.08	**0.001**	0.02	-0.01 – 0.06	0.160	0.04	0.01 – 0.07	**0.007**
polAff bin str [Dem]	0.10	0.05 – 0.14	**<0.001**	0.07	0.03 – 0.12	**0.001**	0.09	0.04 – 0.14	**<0.001**	0.08	0.04 – 0.12	**<0.001**
nEvents	0.01	0.00 – 0.02	**0.037**	-0.03	-0.07 – 0.00	0.065	0.04	-0.00 – 0.08	0.076	-0.00	-0.03 – 0.03	0.900
polAff bin str [Dem] × nEvents	-0.01	-0.03 – 0.01	0.243	0.02	-0.02 – 0.07	0.300	-0.02	-0.08 – 0.04	0.523	-0.01	-0.06 – 0.04	0.623
**Random Effects**												
σ^2	0.29			0.29			0.29			0.29		
τ_{00}	0.13 $_{subject}$			0.13 $_{subject}$			0.13 $_{subject}$			0.13 $_{subject}$		
	0.00 $_{trialNum\_fixed}$			0.00 $_{trialNum\_fixed}$			0.00 $_{trialNum\_fixed}$			0.00 $_{trialNum\_fixed}$		
ICC	0.31			0.31			0.31			0.31		
N	1451 $_{subject}$			1451 $_{subject}$			1451 $_{subject}$			1451 $_{subject}$		
	25 $_{trialNum\_fixed}$			25 $_{trialNum\_fixed}$			25 $_{trialNum\_fixed}$			25 $_{trialNum\_fixed}$		
Observations	34294			34294			34294			34294		
Marginal R^2 / Conditional R^2	0.005 / 0.316			0.005 / 0.316			0.005 / 0.316			0.004 / 0.316		

	M2.2_Coastal Flood_300d			M2.2_Drought_300d			M2.2_Excessive Heat_300d			M2.2_Flash Flood_300d		
Predictors	*Estimates*	*CI*	*p*	*Estimates*	*CI*	*p*	*Estimates*	*CI*	*p*	*Estimates*	*CI*	*p*
(Intercept)	0.04	0.01 – 0.06	**0.006**	0.04	0.01 – 0.07	**0.005**	0.04	0.01 – 0.06	**0.012**	0.04	0.00 – 0.07	**0.033**
polAff bin str [Dem]	0.08	0.04 – 0.12	**<0.001**	0.08	0.04 – 0.12	**<0.001**	0.09	0.05 – 0.13	**<0.001**	0.09	0.04 – 0.14	**0.001**
nEvents	0.09	-0.08 – 0.27	0.281	-0.00	-0.01 – 0.01	0.719	0.01	-0.01 – 0.02	0.542	0.00	-0.01 – 0.01	0.925
polAff bin str [Dem] × nEvents	-0.03	-0.24 – 0.18	0.812	-0.00	-0.02 – 0.02	0.986	-0.02	-0.05 – 0.01	0.127	-0.01	-0.02 – 0.01	0.566
**Random Effects**												
σ^2	0.29			0.29			0.29			0.29		
τ_{00}	0.13 subject			0.13 subject			0.13 subject			0.13 subject		
	0.00 trialNum_fixed			0.00 trialNum_fixed			0.00 trialNum_fixed			0.00 trialNum_fixed		
ICC	0.31			0.31			0.31			0.31		
N	1451 subject			1451 subject			1451 subject			1451 subject		
	25 trialNum_fixed			25 trialNum_fixed			25 trialNum_fixed			25 trialNum_fixed		
Observations	34294			34294			34294			34294		
Marginal R^2 / Conditional R^2	0.004 / 0.316			0.004 / 0.316			0.004 / 0.316			0.004 / 0.316		

	M2.2_Hail_300d			M2.2_Strong Wind_300d			M2.2_Tornado_300d			M2.2_Wildfire_300d		
Predictors	*Estimates*	*CI*	*p*	*Estimates*	*CI*	*p*	*Estimates*	*CI*	*p*	*Estimates*	*CI*	*p*
(Intercept)	0.02	-0.01 – 0.05	0.223	0.05	0.02 – 0.08	**0.001**	0.02	-0.01 – 0.05	0.211	0.04	0.01 – 0.07	**0.005**
polAff bin str [Dem]	0.10	0.05 – 0.14	**<0.001**	0.07	0.03 – 0.12	**0.001**	0.09	0.04 – 0.14	**<0.001**	0.08	0.04 – 0.12	**<0.001**
nEvents	0.01	0.00 – 0.02	**0.037**	-0.02	-0.06 – 0.01	0.124	0.04	0.00 – 0.08	**0.039**	-0.01	-0.03 – 0.02	0.686
polAff bin str [Dem] × nEvents	-0.01	-0.03 – 0.01	0.249	0.02	-0.02 – 0.06	0.388	-0.02	-0.08 – 0.04	0.467	-0.01	-0.05 – 0.04	0.790
**Random Effects**												
σ^2	0.29			0.29			0.29			0.29		
τ_{00}	0.13 $_{subject}$			0.13 $_{subject}$			0.13 $_{subject}$			0.13 $_{subject}$		
	0.00 $_{trialNum\_fixed}$			0.00 $_{trialNum\_fixed}$			0.00 $_{trialNum\_fixed}$			0.00 $_{trialNum\_fixed}$		
ICC	0.31			0.31			0.31			0.31		
N	1451 $_{subject}$			1451 $_{subject}$			1451 $_{subject}$			1451 $_{subject}$		
	25 $_{trialNum\_fixed}$			25 $_{trialNum\_fixed}$			25 $_{trialNum\_fixed}$			25 $_{trialNum\_fixed}$		
Observations	34294			34294			34294			34294		
Marginal R^2 / Conditional R^2	0.005 / 0.316			0.004 / 0.316			0.005 / 0.316			0.004 / 0.316		

	M2.2_Coastal Flood_330d			M2.2_Drought_330d			M2.2_Excessive Heat_330d			M2.2_Flash Flood_330d		
Predictors	*Estimates*	*CI*	*p*	*Estimates*	*CI*	*p*	*Estimates*	*CI*	*p*	*Estimates*	*CI*	*p*
(Intercept)	0.03	0.00 – 0.06	**0.023**	0.04	0.01 – 0.07	**0.005**	0.04	0.01 – 0.06	**0.011**	0.04	0.00 – 0.07	**0.040**
polAff bin str [Dem]	0.09	0.05 – 0.13	**<0.001**	0.08	0.04 – 0.12	**<0.001**	0.09	0.05 – 0.13	**<0.001**	0.09	0.04 – 0.14	**0.001**
nEvents	0.09	0.01 – 0.17	**0.025**	-0.00	-0.01 – 0.01	0.699	0.00	-0.01 – 0.02	0.558	0.00	-0.01 – 0.01	0.852
polAff bin str [Dem] × nEvents	-0.08	-0.19 – 0.03	0.171	0.00	-0.02 – 0.02	0.993	-0.02	-0.04 – 0.00	0.123	-0.01	-0.02 – 0.01	0.552
**Random Effects**												
σ^2	0.29			0.29			0.29			0.29		
τ_{00}	0.13 $_{subject}$			0.13 $_{subject}$			0.13 $_{subject}$			0.13 $_{subject}$		
	0.00 $_{trialNum\_fixed}$			0.00 $_{trialNum\_fixed}$			0.00 $_{trialNum\_fixed}$			0.00 $_{trialNum\_fixed}$		
ICC	0.31			0.31			0.31			0.31		
N	1451 $_{subject}$			1451 $_{subject}$			1451 $_{subject}$			1451 $_{subject}$		
	25 $_{trialNum\_fixed}$			25 $_{trialNum\_fixed}$			25 $_{trialNum\_fixed}$			25 $_{trialNum\_fixed}$		
Observations	34294			34294			34294			34294		
Marginal R^2 / Conditional R^2	0.005 / 0.316			0.004 / 0.316			0.004 / 0.316			0.004 / 0.316		

	M2.2_Hail_330d			M2.2_Strong Wind_330d			M2.2_Tornado_330d			M2.2_Wildfire_330d		
Predictors	*Estimates*	*CI*	*p*	*Estimates*	*CI*	*p*	*Estimates*	*CI*	*p*	*Estimates*	*CI*	*p*
(Intercept)	0.02	-0.01 – 0.05	0.209	0.05	0.02 – 0.08	**0.001**	0.01	-0.02 – 0.05	0.460	0.04	0.01 – 0.07	**0.004**
polAff bin str [Dem]	0.10	0.05 – 0.14	**<0.001**	0.07	0.03 – 0.12	**0.001**	0.10	0.05 – 0.14	**<0.001**	0.08	0.04 – 0.12	**<0.001**
nEvents	0.01	0.00 – 0.02	**0.044**	-0.02	-0.05 – 0.01	0.137	0.05	0.01 – 0.09	**0.010**	-0.01	-0.04 – 0.02	0.558
polAff bin str [Dem] × nEvents	-0.01	-0.03 – 0.01	0.269	0.02	-0.02 – 0.06	0.423	-0.03	-0.09 – 0.03	0.303	-0.00	-0.04 – 0.04	0.881
**Random Effects**												
σ^2	0.29			0.29			0.29			0.29		
τ_{00}	0.13 $_{subject}$			0.13 $_{subject}$			0.13 $_{subject}$			0.13 $_{subject}$		
	0.00 $_{trialNum\_fixed}$			0.00 $_{trialNum\_fixed}$			0.00 $_{trialNum\_fixed}$			0.00 $_{trialNum\_fixed}$		
ICC	0.31			0.31			0.31			0.31		
N	1451 $_{subject}$			1451 $_{subject}$			1451 $_{subject}$			1451 $_{subject}$		
	25 $_{trialNum\_fixed}$			25 $_{trialNum\_fixed}$			25 $_{trialNum\_fixed}$			25 $_{trialNum\_fixed}$		
Observations	34294			34294			34294			34294		
Marginal R^2 / Conditional R^2	0.005 / 0.316			0.004 / 0.316			0.006 / 0.316			0.004 / 0.316		

	M2.2_Coastal Flood_360d			M2.2_Drought_360d			M2.2_Excessive Heat_360d			M2.2_Flash Flood_360d		
Predictors	*Estimates*	*CI*	*p*	*Estimates*	*CI*	*p*	*Estimates*	*CI*	*p*	*Estimates*	*CI*	*p*
(Intercept)	0.03	0.01 – 0.06	**0.018**	0.04	0.01 – 0.07	**0.006**	0.04	0.01 – 0.06	**0.012**	0.04	0.00 – 0.07	**0.040**
polAff bin str [Dem]	0.08	0.04 – 0.13	**<0.001**	0.08	0.04 – 0.12	**<0.001**	0.09	0.05 – 0.13	**<0.001**	0.09	0.04 – 0.14	**0.001**
nEvents	0.05	-0.01 – 0.10	0.091	-0.00	-0.01 – 0.01	0.745	0.00	-0.01 – 0.02	0.523	0.00	-0.01 – 0.01	0.854
polAff bin str [Dem] × nEvents	-0.04	-0.12 – 0.04	0.291	0.00	-0.02 – 0.02	0.978	-0.02	-0.04 – 0.00	0.107	-0.00	-0.02 – 0.01	0.632
**Random Effects**												
σ^2	0.29			0.29			0.29			0.29		
τ_{00}	0.13 $_{subject}$			0.13 $_{subject}$			0.13 $_{subject}$			0.13 $_{subject}$		
	0.00 $_{trialNum\_fixed}$			0.00 $_{trialNum\_fixed}$			0.00 $_{trialNum\_fixed}$			0.00 $_{trialNum\_fixed}$		
ICC	0.31			0.31			0.31			0.31		
N	1451 $_{subject}$			1451 $_{subject}$			1451 $_{subject}$			1451 $_{subject}$		
	25 $_{trialNum\_fixed}$			25 $_{trialNum\_fixed}$			25 $_{trialNum\_fixed}$			25 $_{trialNum\_fixed}$		
Observations	34294			34294			34294			34294		
Marginal R^2 / Conditional R^2	0.004 / 0.316			0.004 / 0.316			0.004 / 0.316			0.004 / 0.316		

	M2.2_Hail_360d			M2.2_Strong Wind_360d			M2.2_Tornado_360d			M2.2_Wildfire_360d		
Predictors	*Estimates*	*CI*	*p*	*Estimates*	*CI*	*p*	*Estimates*	*CI*	*p*	*Estimates*	*CI*	*p*
(Intercept)	0.02	-0.01 – 0.05	0.183	0.05	0.02 – 0.08	**0.001**	0.01	-0.02 – 0.04	0.576	0.04	0.01 – 0.07	**0.003**
polAff bin str [Dem]	0.09	0.05 – 0.14	**<0.001**	0.07	0.03 – 0.12	**0.001**	0.10	0.05 – 0.15	**<0.001**	0.08	0.04 – 0.12	**<0.001**
nEvents	0.01	-0.00 – 0.02	0.063	-0.02	-0.05 – 0.01	0.168	0.06	0.02 – 0.10	**0.005**	-0.01	-0.03 – 0.02	0.457
polAff bin str [Dem] × nEvents	-0.01	-0.03 – 0.01	0.310	0.01	-0.03 – 0.05	0.521	-0.03	-0.09 – 0.03	0.306	0.00	-0.03 – 0.04	0.980
**Random Effects**												
σ^2	0.29			0.29			0.29			0.29		
τ_{00}	0.13 $_{subject}$			0.13 $_{subject}$			0.13 $_{subject}$			0.13 $_{subject}$		
	0.00 $_{trialNum\_fixed}$			0.00 $_{trialNum\_fixed}$			0.00 $_{trialNum\_fixed}$			0.00 $_{trialNum\_fixed}$		
ICC	0.31			0.31			0.31			0.31		
N	1451 $_{subject}$			1451 $_{subject}$			1451 $_{subject}$			1451 $_{subject}$		
	25 $_{trialNum\_fixed}$			25 $_{trialNum\_fixed}$			25 $_{trialNum\_fixed}$			25 $_{trialNum\_fixed}$		
Observations	34294			34294			34294			34294		
Marginal R^2 / Conditional R^2	0.005 / 0.316			0.004 / 0.316			0.006 / 0.316			0.004 / 0.316		

Table S10: ***Sensitivity analyses: Mixed-effects models predicting ΔDuration across different EWE time windows and event types.*** *ΔDuration (deltaDuration) represents relative dwell time spent on carbon information compared to bonus information. Random intercepts were included for participants (subject) and trials (trialNum_fixed) (N = 1,451 participants). The models included political affiliation (polAff_bin_str, subject-level binary variable with 0 = Republican and 1 = Democrat), the number of EWEs a participant experienced (nEvents) and their interaction. Models were estimated for different time windows (60-360 days) and event types (coastal flood, drought, excessive heat, flash flood, hail, strong wind, tornado, wildfire). Estimates represent unstandardized beta coefficients. Confidence intervals are 95%. Statistically significant effects (p < .05) are shown in bold.*

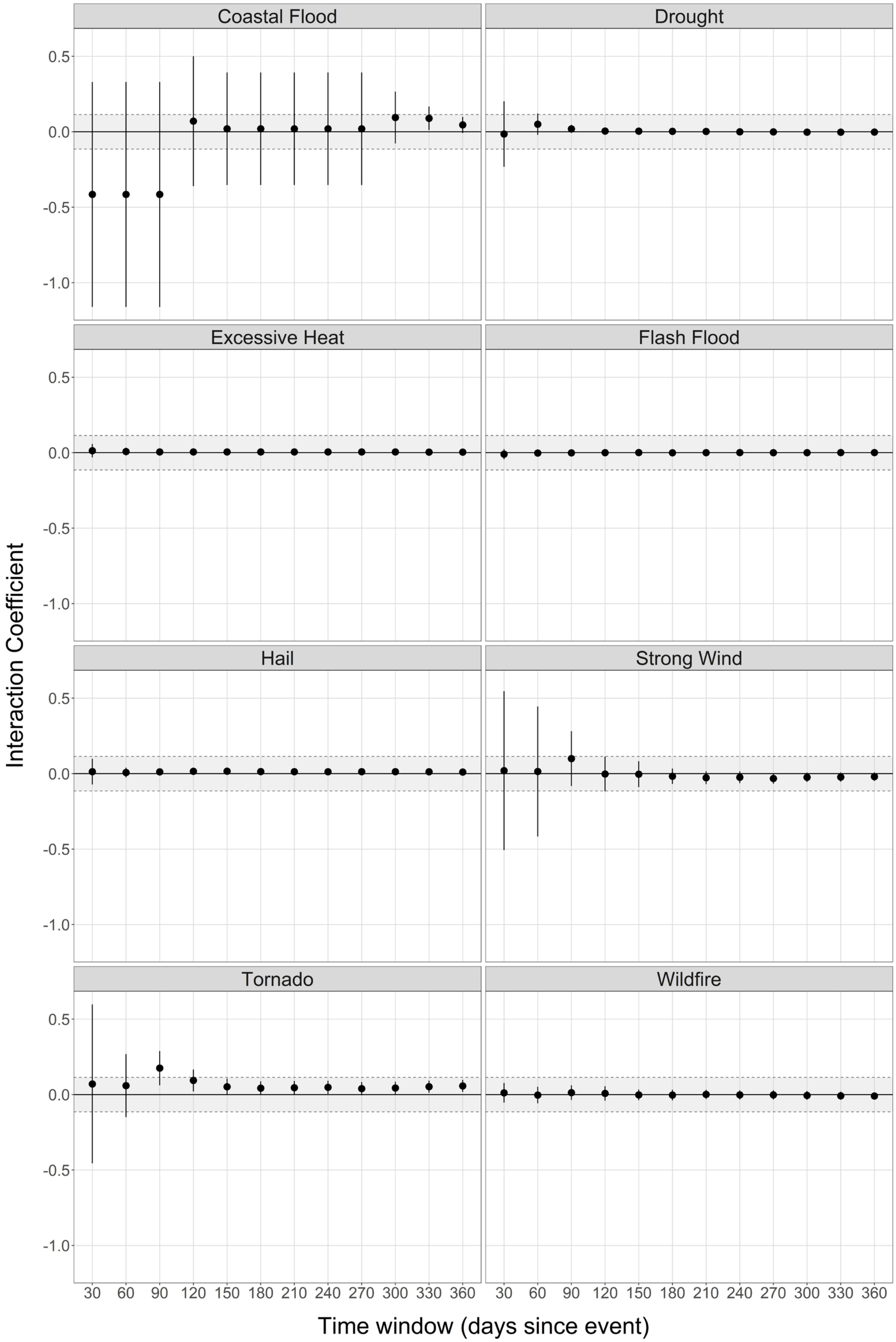
Coastal Flood
Drought
Excessive Heat
Flash Flood
Hail
Strong Wind
Tornado
Wildfire
Interaction Coefficient
0.5
0.0
-0.5
-1.0
30 60 90 120 150 180 210 240 270 300 330 360
Time window (days since event)

Figure S7: ***Equivalence testing of interaction coefficients across different EWE time windows and event types****: Points represent estimated interaction coefficients for the interaction between political affiliation and the number of EWEs a participant experienced predicting ΔDuration across models using different EWE time windows (30–360 days preceding measurement) and event types (coastal flood, drought, excessive heat, flash flood, hail, strong wind, tornado, wildfire) (N = 1,451 participants). Vertical lines indicate 95% confidence intervals. The shaded area denotes the region of practical equivalence (ROPE; ±0.1143), based on the smallest effect size of interest (SESOI).*

1.2.2. Extreme Weather Events as a Moderator of the Relationship Between Political Affiliation and Choice (H_{S2})

Consistent with the results for attention allocation, the interaction between political affiliation and EWE exposure did not significantly predict pro-climate choices (H_{S2}; Table S11), providing no evidence that exposure moderates partisan differences in pro-environmental decision outcomes.

	M2.1			M2.2		
Predictors	*Odds Ratios*	*CI*	*p*	*Odds Ratios*	*CI*	*p*
(Intercept)	1.08	0.78 – 1.49	0.648	1.07	0.75 – 1.52	0.726
polAff bin str [Dem]	2.03	1.48 – 2.79	**<0.001**	2.09	1.36 – 3.20	**0.001**
nEpisodesIsGreaterThanZeroTRUE	0.75	0.54 – 1.02	0.067	0.76	0.50 – 1.17	0.218
polAff bin str [Dem] × nEpisodesIsGreaterThanZeroTRUE				0.95	0.50 – 1.78	0.863
**Random Effects**						
σ^2	3.29			3.29		
τ_{00}	8.47 subject			8.47 subject		
	0.24 trialNum_fixed			0.24 trialNum_fixed		
ICC	0.73			0.73		
N	1451 subject			1451 subject		
	25 trialNum_fixed			25 trialNum_fixed		
Observations	34428			34428		
Marginal R^2 / Conditional R^2	0.012 / 0.729			0.013 / 0.729		

	M2.3		
Predictors	*Odds Ratios*	*CI*	*p*
(Intercept)	0.28	0.15 – 0.53	**<0.001**
polAff bin str [Dem]	2.24	1.46 – 3.42	**<0.001**
nEpisodesIsGreaterThanZeroTRUE	0.86	0.56 – 1.31	0.471
carbon prcnt	1.01	1.01 – 1.01	**<0.001**
bonus prcnt	0.99	0.99 – 0.99	**<0.001**
age	1.03	1.02 – 1.04	**<0.001**
racethn [European]	1.12	0.81 – 1.55	0.488
racethn [Hispanic]	1.19	0.81 – 1.75	0.383
racethn [African]	0.27	0.13 – 0.55	**<0.001**
racethn [Asian]	1.90	0.90 – 4.00	0.091
racethn [moreThanOne]	1.16	0.53 – 2.53	0.715
polAff bin str [Dem] × nEpisodesIsGreaterThanZeroTRUE	0.85	0.46 – 1.60	0.621
**Random Effects**			
σ^2		3.29	
$\tau_{00\ subject}$		8.20	
$\tau_{00\ trialNum\_fixed}$		0.02	
ICC		0.71	
$N_{subject}$		1451	
$N_{trialNum\_fixed}$		25	
Observations		34428	
Marginal R^2 / Conditional R^2		0.052 / 0.729	

	M2.4		
Predictors	*Odds Ratios*	*CI*	*p*
(Intercept)	1.00	0.70 – 1.44	0.983
polAff bin str [Dem]	2.19	1.43 – 3.37	<0.001
nEpisodesIsGreaterThanZeroTRUE	0.83	0.51 – 1.35	0.453
abnormality final	0.95	0.81 – 1.11	0.519
sum damage	1.02	0.86 – 1.19	0.856
sum injuries	1.08	0.93 – 1.27	0.313
sum deaths	0.99	0.85 – 1.17	0.947
recency index	1.32	0.17 – 10.61	0.791
population density peopPerSqKilometer	1.01	0.85 – 1.21	0.882
countyPvi	0.79	0.65 – 0.96	0.019
countyIncome	1.13	0.95 – 1.35	0.158
polAff bin str [Dem] × nEpisodesIsGreaterThanZeroTRUE	0.89	0.47 – 1.67	0.710
**Random Effects**			
σ^2		3.29	
$\tau_{00\ subject}$		8.42	
$\tau_{00\ trialNum\_fixed}$		0.24	
ICC		0.72	
$N_{subject}$		1451	
N trialNum_fixed		25	
Observations		34428	
Marginal R^2 / Conditional R^2		0.017 / 0.729	

	M2.5		
Predictors	*Odds Ratios*	*CI*	*p*
(Intercept)	1.06	0.79 – 1.42	0.706
polAff bin str [Dem]	1.89	1.36 – 2.62	<0.001
nEpisodes durationWeighted	0.97	0.96 – 0.99	0.001
polAff bin str [Dem] × nEpisodes durationWeighted	1.02	0.99 – 1.04	0.279
**Random Effects**			
σ^2		3.29	
$\tau_{00\ subject}$		8.43	
$\tau_{00\ trialNum\_fixed}$		0.24	
ICC		0.72	
$N_{subject}$		1451	
$N_{trialNum\_fixed}$		25	
Observations		34428	
Marginal R^2 / Conditional R^2		0.017 / 0.730	

Table S11: ***Mixed-effects logistic regression models predicting choice (M2.1–M2.5).*** *Choice represents the actual decision in the Carbon Emission Task (trial-level variable with 0 = pro-self and 1 = pro-climate). Random intercepts were included for participants (subject) and trials (trialNum_fixed) (N = 1,451 participants). Model M2.1 included political affiliation (polAff_bin_str, subject-level binary variable with 0 = Republican and 1 = Democrat) and a binary indicator of whether participants had experienced at least one EWE episode in the last 30 days (nEpisodesIsGreaterThanZeroTRUE). Model M2.2 additionally included their interaction. Model M2.3 further controlled for attribute differences (carbon_prcnt and bonus_prcnt), age and race/ethnicity (racethn). racethn was effect-coded using sum-to-zero contrasts, such that coefficients represent deviations from the overall mean. The category Other is not displayed and can be derived as the negative sum of the reported coefficients. Model M2.4 instead controlled for county-level indicators related to EWE severity and impacts, specifically the abnormality score for the number of EWE (abnormality_final), the amount of financial damage (sum_damage), and the number of injuries (sum_injuries) and deaths (sum_deaths) associated with each episode. The model further controlled for the temporal proximity of the most recent extreme weather episode (recency_index), as well as broader county characteristics: population density (population_density_peopPerSqKilometer), median household income (countyIncome), and the county-level Partisan Voting Index (countyPvi). All county-level variables included as controls in Model M2.4 were z-standardized, except recency_index. Model M2.5 replaced the binary EWE indicator with a continuous, duration-weighted measure of EWE exposure (nEpisodes_durationWeighted) and its interaction with political affiliation. Confidence intervals are 95%. Statistically significant effects (p < .05) are shown in bold.*

1.3. Subjective-Attribution-Hypothesis (H_3 and H_{S3})

1.3.1. Subjective Attribution to Climate Change and ΔDuration (H_3)

As expected, subjective attribution of EWE to climate change differed by political affiliation. Democrats reported higher levels of attribution to climate change (*M* = 4.52, *SD* = 0.65) than Republican participants (*M* = 3.94, *SD* = 1.16). To test the hypothesis that subjective attribution of EWE to climate change moderates the interaction effect of political affiliation and EWE exposure on visual attention, we estimated mixed-effects models with ΔDuration

as the dependent variable and random intercepts for participants and trials. All regression results are displayed in Table S12.

In Model 1, political affiliation, EWE exposure, and subjective attribution of EWE to climate change were included as predictors of ΔDuration. Political affiliation remained a significant predictor (b = 0.05, p = .021), while EWE exposure was not. Subjective attribution of EWE to climate change was positively associated with ΔDuration (b = 0.05, p < .001), suggesting that participants who more strongly attributed EWE to climate change devoted relatively more visual attention to the carbon emission attribute.

Model 2 included political affiliation, subjective attribution of EWE to climate change and their interaction. This interaction was positive and statistically significant (b = 0.07, p = .006), indicating that the effect of political affiliation on ΔDuration became stronger as subjective attribution of EWE to climate change increased. Johnson–Neyman analyses indicated that the effect of political affiliation on ΔDuration was negative at very low levels of subjective attribution, not significant at moderate levels, and positive at high levels of attribution (Figure S8). Re-estimating Model 2 using the full sample yielded a similar positive and statistically significant interaction (Table S13, b = 0.06, p = .017).

Model 3 included EWE exposure and subjective attribution of EWE to climate change and their interaction. Contrary to our expectations, this interaction was negative and statistically significant (b = −0.05, p = .017), indicating that the positive association between EWE exposure and ΔDuration diminishes as subjective attribution increases. The interaction remained negative and statistically significant when Model 3 was re-estimated using the full sample (Table S13, b = -0.04, p = .034).

Model 4 included political affiliation, EWE exposure, and subjective attribution of EWE to climate change, as well as all three two-way interactions. As in Hypothesis 2, the interaction between political affiliation and EWE exposure was not statistically significant (b = -0.03, p = .539). Model 5 additionally included the full three-way interaction between political affiliation, EWE exposure, and subjective attribution to climate change. This interaction was not statistically significant, indicating that the relationship between political affiliation and ΔDuration did not depend jointly on EWE exposure and subjective attribution (b = -0.02, p = .714). Re-estimating Model 5 using the full sample yielded the same conclusion: the three-way interaction remained non significant (Table S13, b = -0.05, p = .376). This pattern remained unchanged when additionally controlling for task- and participant-level characteristics (Model 6, b = -0.03, p = .621) and county-level characteristics (Model 7, b = -0.02, p = .660). The confidence intervals of the interaction terms lie entirely within the region of practical equivalence, indicating practical equivalence of this three-way interaction (see Figure S9).

However, when replacing the binary exposure indicator with a duration-weighted measure of EWE exposure, the three-way interaction between political affiliation, EWE exposure, and subjective attribution was positively and statistically significant (b = 0.01, p = .048). Figure S10 displays the corresponding plot, indicating that the association between EWE exposure and ΔDuration differs by political affiliation at low levels of subjective attribution, but this difference diminishes as attribution increases.

	M3.1			M3.2		
Predictors	*Estimates*	*CI*	*p*	*Estimates*	*CI*	*p*
(Intercept)	-0.16	-0.25 – -0.08	**<0.001**	-0.11	-0.20 – -0.02	**0.021**
polAff bin str [Dem]	0.05	0.01 – 0.09	**0.021**	-0.25	-0.48 – -0.03	**0.024**
nEpisodesIsGreaterThanZeroTRUE	-0.01	-0.05 – 0.03	0.483			
EWE subjAttr mean	0.05	0.03 – 0.07	**<0.001**	0.04	0.02 – 0.06	**0.001**
polAff bin str [Dem] × EWE subjAttr mean				0.07	0.02 – 0.12	**0.006**
**Random Effects**						
σ^2	0.29			0.29		
τ_{00}	0.13 $_{subject}$			0.13 $_{subject}$		
	0.00 $_{trialNum\_fixed}$			0.00 $_{trialNum\_fixed}$		
ICC	0.31			0.31		
N	1451 $_{subject}$			1451 $_{subject}$		
	25 $_{trialNum\_fixed}$			25 $_{trialNum\_fixed}$		
Observations	34294			34294		
Marginal R^2 / Conditional R^2	0.010 / 0.316			0.011 / 0.316		

	M3.3		
Predictors	*Estimates*	*CI*	*p*
(Intercept)	-0.25	-0.36 – -0.14	**<0.001**
nEpisodesIsGreaterThanZeroTRUE	0.18	0.01 – 0.36	**0.034**
EWE subjAttr mean	0.08	0.05 – 0.11	**<0.001**
nEpisodesIsGreaterThanZeroTRUE × EWE subjAttr mean	-0.05	-0.09 – -0.01	**0.017**
**Random Effects**			
σ^2	0.29		
$\tau_{00\ subject}$	0.13		
$\tau_{00\ trialNum\_fixed}$	0.00		
ICC	0.31		
$N_{subject}$	1451		
$N_{trialNum\_fixed}$	25		
Observations	34294		
Marginal R^2 / Conditional R^2	0.010 / 0.316		

		**M3.4**	
Predictors	*Estimates*	*CI*	*p*
(Intercept)	-0.18	-0.30 – -0.07	**0.002**
polAff bin str [Dem]	-0.24	-0.46 – -0.02	**0.034**
nEpisodesIsGreaterThanZeroTRUE	0.17	0.00 – 0.35	**0.045**
EWE subjAttr mean	0.06	0.03 – 0.08	**<0.001**
polAff bin str [Dem] × nEpisodesIsGreaterThanZeroTRUE	-0.03	-0.11 – 0.06	0.539
polAff bin str [Dem] × EWE subjAttr mean	0.07	0.02 – 0.12	**0.007**
nEpisodesIsGreaterThanZeroTRUE × EWE subjAttr mean	-0.04	-0.08 – -0.00	**0.047**
**Random Effects**			
σ^2		0.29	
$\tau_{00\ subject}$		0.13	
$\tau_{00\ trialNum\_fixed}$		0.00	
ICC		0.31	
$N_{subject}$		1451	
$N_{trialNum\_fixed}$		25	
Observations		34294	
Marginal R^2 / Conditional R^2		0.013 / 0.317	

		**M3.5**	
Predictors	*Estimates*	*CI*	*p*
(Intercept)	-0.18	-0.30 – -0.05	**0.005**
polAff bin str [Dem]	-0.27	-0.55 – 0.01	0.058
nEpisodesIsGreaterThanZeroTRUE	0.16	-0.03 – 0.35	0.101
EWE subjAttr mean	0.05	0.02 – 0.08	**<0.001**
polAff bin str [Dem] × nEpisodesIsGreaterThanZeroTRUE	0.06	-0.40 – 0.51	0.803
polAff bin str [Dem] × EWE subjAttr mean	0.08	0.01 – 0.14	**0.020**
nEpisodesIsGreaterThanZeroTRUE × EWE subjAttr mean	-0.04	-0.08 – 0.01	0.109
(polAff bin str [Dem] × nEpisodesIsGreaterThanZeroTRUE) × EWE subjAttr mean	-0.02	-0.12 – 0.08	0.714
**Random Effects**			
σ^2		0.29	
$\tau_{00\ subject}$		0.13	
τ00 trialNum_fixed		0.00	
ICC		0.31	
$N_{subject}$		1451	
$N_{trialNum\_fixed}$		25	
Observations		34294	
Marginal R^2 / Conditional R^2		0.013 / 0.317	

		M3.6	
Predictors	*Estimates*	*CI*	*p*
(Intercept)	-0.38	-0.54 – -0.23	**<0.001**
polAff bin str [Dem]	-0.13	-0.41 – 0.15	0.352
nEpisodesIsGreaterThanZeroTRUE	0.16	-0.02 – 0.35	0.089
EWE subjAttr mean	0.08	0.05 – 0.11	**<0.001**
trialNum	0.00	0.00 – 0.00	**<0.001**
CB carbonUp	-0.04	-0.08 – -0.00	**0.040**
gender [Male]	-0.04	-0.08 – -0.00	**0.039**
age	0.00	0.00 – 0.00	**<0.001**
racethn [European]	-0.00	-0.04 – 0.04	0.881
racethn [Hispanic]	-0.06	-0.11 – -0.01	**0.012**
racethn [African]	-0.02	-0.11 – 0.07	0.634
racethn [Asian]	0.11	0.02 – 0.20	**0.020**
racethn [moreThanOne]	-0.02	-0.12 – 0.07	0.622
polAff bin str [Dem] × nEpisodesIsGreaterThanZeroTRUE	0.07	-0.38 – 0.52	0.751
polAff bin str [Dem] × EWE subjAttr mean	0.04	-0.02 – 0.10	0.221
nEpisodesIsGreaterThanZeroTRUE × EWE subjAttr mean	-0.03	-0.08 – 0.01	0.134
(polAff bin str [Dem] × nEpisodesIsGreaterThanZeroTRUE) × EWE subjAttr mean	-0.03	-0.13 – 0.08	0.621
**Random Effects**			
σ^2		0.29	
$\tau_{00\ subject}$		0.12	
$\tau_{00\ trialNum\_fixed}$		0.00	
ICC		0.30	
$N_{subject}$		1451	
$N_{trialNum\_fixed}$		25	
Observations		34294	
Marginal R^2 / Conditional R^2		0.028 / 0.318	

	M3.7		
Predictors	*Estimates*	*CI*	*p*
(Intercept)	-0.20	-0.33 – -0.08	**0.001**
polAff bin str [Dem]	-0.26	-0.54 – 0.02	0.073
nEpisodesIsGreaterThanZeroTRUE	0.19	-0.01 – 0.38	0.059
EWE subjAttr mean	0.06	0.03 – 0.09	**<0.001**
abnormality final	-0.00	-0.02 – 0.02	0.946
sum damage	0.00	-0.02 – 0.02	0.764
sum injuries	0.01	-0.01 – 0.03	0.497
sum deaths	0.01	-0.02 – 0.03	0.620
recency index	-0.16	-0.42 – 0.10	0.230
population density peopPerSqKilometer	0.02	-0.00 – 0.04	0.060
countyPvi	-0.02	-0.05 – 0.00	0.057
countyIncome	-0.02	-0.04 – 0.00	0.107
polAff bin str [Dem] × nEpisodesIsGreaterThanZeroTRUE	0.08	-0.38 – 0.53	0.743
polAff bin str [Dem] × EWE subjAttr mean	0.07	0.01 – 0.14	**0.024**
nEpisodesIsGreaterThanZeroTRUE × EWE subjAttr mean	-0.04	-0.09 – 0.01	0.092
(polAff bin str [Dem] × nEpisodesIsGreaterThanZeroTRUE) × EWE subjAttr mean	-0.02	-0.12 – 0.08	0.660
**Random Effects**			
σ^2		0.29	
$\tau_{00\ subject}$		0.13	
$\tau_{00\ trialNum\_fixed}$		0.00	
ICC		0.31	
$N_{subject}$		1451	
$N_{trialNum\_fixed}$		25	
Observations		34294	
Marginal R^2 / Conditional R^2		0.016 / 0.318	

	M3.8		
Predictors	*Estimates*	*CI*	*p*
(Intercept)	-0.12	-0.22 – -0.02	**0.015**
polAff bin str [Dem]	-0.20	-0.43 – 0.03	0.087
nEpisodes durationWeighted	0.00	-0.00 – 0.01	0.467
EWE subjAttr mean	0.04	0.02 – 0.06	**0.001**
polAff bin str [Dem] × nEpisodes durationWeighted	-0.05	-0.09 – -0.00	**0.035**
polAff bin str [Dem] × EWE subjAttr mean	0.06	0.01 – 0.11	**0.023**
nEpisodes durationWeighted × EWE subjAttr mean	-0.00	-0.00 – 0.00	0.692
(polAff bin str [Dem] × nEpisodes durationWeighted) × EWE subjAttr mean	0.01	0.00 – 0.02	**0.048**
**Random Effects**			
σ^2		0.29	
$\tau_{00\ subject}$		0.13	
$\tau_{00\ trialNum\_fixed}$		0.00	
ICC		0.31	
$N_{subject}$		1451	
$N_{trialNum\_fixed}$		25	
Observations		34294	
Marginal R^2 / Conditional R^2		0.013 / 0.317	

Table S12: ***Mixed-effects models predicting ΔDuration (M3.1–M3.8).*** *ΔDuration (deltaDuration) represents relative dwell time spent on carbon information compared to bonus information. Random intercepts were included for participants (subject) and trials (trialNum_fixed) (N = 1,451 participants). In Model M3.1, political affiliation (polAff_bin_str), EWE exposure (nEpisodesIsGreaterThanZero) and subjective attribution of EWE to climate change (EWE_subAttr_mean) were included as predictors of ΔDuration. Model M3.2 included political affiliation, subjective attribution and their interaction. Model M3.3 included EWE exposure and subjective attribution and their interaction. Model M3.4 included political affiliation, EWE exposure, and subjective attribution, as well as all three two-way interactions. Model M3.5 additionally included the three-way interaction between political affiliation, EWE exposure and subjective attribution. Model M3.6 further controlled for trial position (trialNum), the position of the carbon attribute (CB_carbonUp), gender, age and race/ethnicity (racethn). racethn was effect-coded using sum-to-zero contrasts, such that coefficients represent deviations from the overall mean. The category Other is not displayed and can be derived as the negative sum of the reported coefficients. Model M3.7 instead controlled for county-level indicators related to EWE severity and impacts, specifically the abnormality score for the number of EWE (abnormality_final), the amount of financial damage (sum_damage), and the number of injuries (sum_injuries) and deaths (sum_deaths) associated with each episode. The model further controlled for the temporal proximity of the most recent extreme weather episode (recency_index), as well as broader county characteristics: population density (population_density_peopPerSqKilometer), median household income (countyIncome), and the county-level Partisan Voting Index (countyPvi). All county-level variables included as controls in Model M3.7 were z-standardized, except recency_index. Model M3.8 included political affiliation, subjective attribution and a continuous, duration-weighted measure of EWE exposure (nEpisodes_durationWeighted), as well as all three two-way interactions and the three-way interaction. Estimates represent unstandardized beta coefficients. Confidence intervals are 95%. Statistically significant effects ($p < .05$) are shown in bold.*

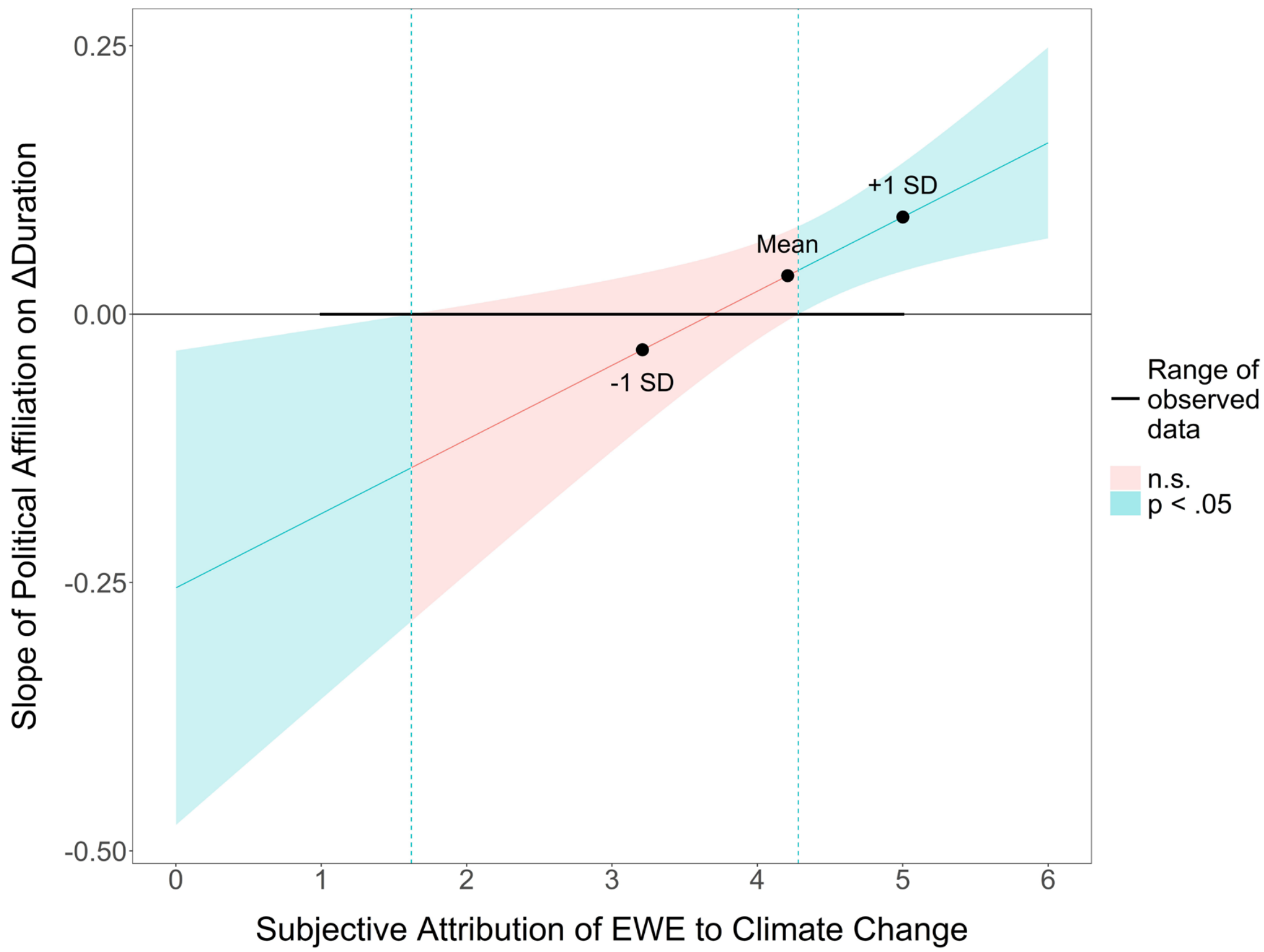

Figure S8: ***Interaction between political affiliation and subjective attribution of EWEs to climate change on ΔDuration.*** *The Johnson–Neyman plot illustrates the interaction between political affiliation and subjective attribution of EWEs to climate change estimated in Model M3.2 (N = 1,451 participants). The line represents the conditional effect of political affiliation on ΔDuration across values of subjective attribution. Shaded areas indicate regions where the effect is statistically significant ($p < .05$; blue) or non-significant (red). Points indicate the conditional effects at −1 SD, the mean, and +1 SD of subjective attribution. The black horizontal bar represents the observed range of the subjective attribution.*

	M3.2			M3.3		
Predictors	*Estimates*	*CI*	*p*	*Estimates*	*CI*	*p*
(Intercept)	-0.10	-0.20 – -0.00	**0.040**	-0.24	-0.35 – -0.13	**<0.001**
polAff bin str [Dem]	-0.23	-0.46 – -0.01	**0.043**			
EWE subjAttr mean	0.03	0.01 – 0.06	**0.004**	0.07	0.05 – 0.10	**<0.001**
polAff bin str [Dem] × EWE subjAttr mean	0.06	0.01 – 0.11	**0.017**			
nEpisodesIsGreaterThanZeroTRUE				0.19	0.02 – 0.37	**0.033**
nEpisodesIsGreaterThanZeroTRUE × EWE subjAttr mean				-0.04	-0.08 – -0.00	**0.034**
**Random Effects**						
σ^2	0.28			0.28		
τ_{00}	0.17 subject			0.17 subject		
	0.00 trialNum_fixed			0.00 trialNum_fixed		
ICC	0.37			0.38		
N	1936 subject			1936 subject		
	25 trialNum_fixed			25 trialNum_fixed		
Observations	40823			40823		
Marginal R^2 / Conditional R^2	0.008 / 0.380			0.007 / 0.380		

		M3.5	
Predictors	*Estimates*	*CI*	*p*
(Intercept)	-0.16	-0.29 – -0.03	**0.013**
polAff bin str [Dem]	-0.32	-0.62 – -0.02	**0.039**
nEpisodesIsGreaterThanZeroTRUE	0.15	-0.05 – 0.34	0.141
EWE subjAttr mean	0.05	0.01 – 0.08	**0.004**
polAff bin str [Dem] × nEpisodesIsGreaterThanZeroTRUE	0.18	-0.28 – 0.64	0.439
polAff bin str [Dem] × EWE subjAttr mean	0.08	0.02 – 0.15	**0.016**
nEpisodesIsGreaterThanZeroTRUE × EWE subjAttr mean	-0.03	-0.08 – 0.02	0.225
(polAff bin str [Dem] × nEpisodesIsGreaterThanZeroTRUE) × EWE subjAttr mean	-0.05	-0.15 – 0.06	0.376
**Random Effects**			
σ^2		0.28	
$\tau_{00\ subject}$		0.17	
$\tau_{00\ trialNum\_fixed}$		0.00	
ICC		0.37	
$N_{subject}$		1936	
$N_{trialNum\_fixed}$		25	
Observations		40823	
Marginal R^2 / Conditional R^2		0.010 / 0.380	

Table S13: ***Mixed-effects models predicting ΔDuration in the full sample (M3.2, M3.3, and M3.5).*** *ΔDuration (deltaDuration) represents relative dwell time spent on carbon information compared to bonus information. The models were estimated on the full sample (N = 1,936 participants), including all participants with at least one valid ΔDuration observation across trials (participants without valid observations had either no box openings or only box openings shorter than 200 ms). Model M3.2 included political affiliation (polAff_bin_str), subjective attribution of EWE to climate change (EWE_subAttr_mean) and their interaction. Model M3.3 included EWE exposure (nEpisodesIsGreaterThanZero) and subjective attribution and their interaction. Model M3.5 included political affiliation, EWE exposure and subjective attribution, as well as all three two-way interactions and the three-way interaction. Estimates represent unstandardized beta coefficients. Confidence intervals are 95%. Statistically significant effects (p < .05) are shown in bold.*

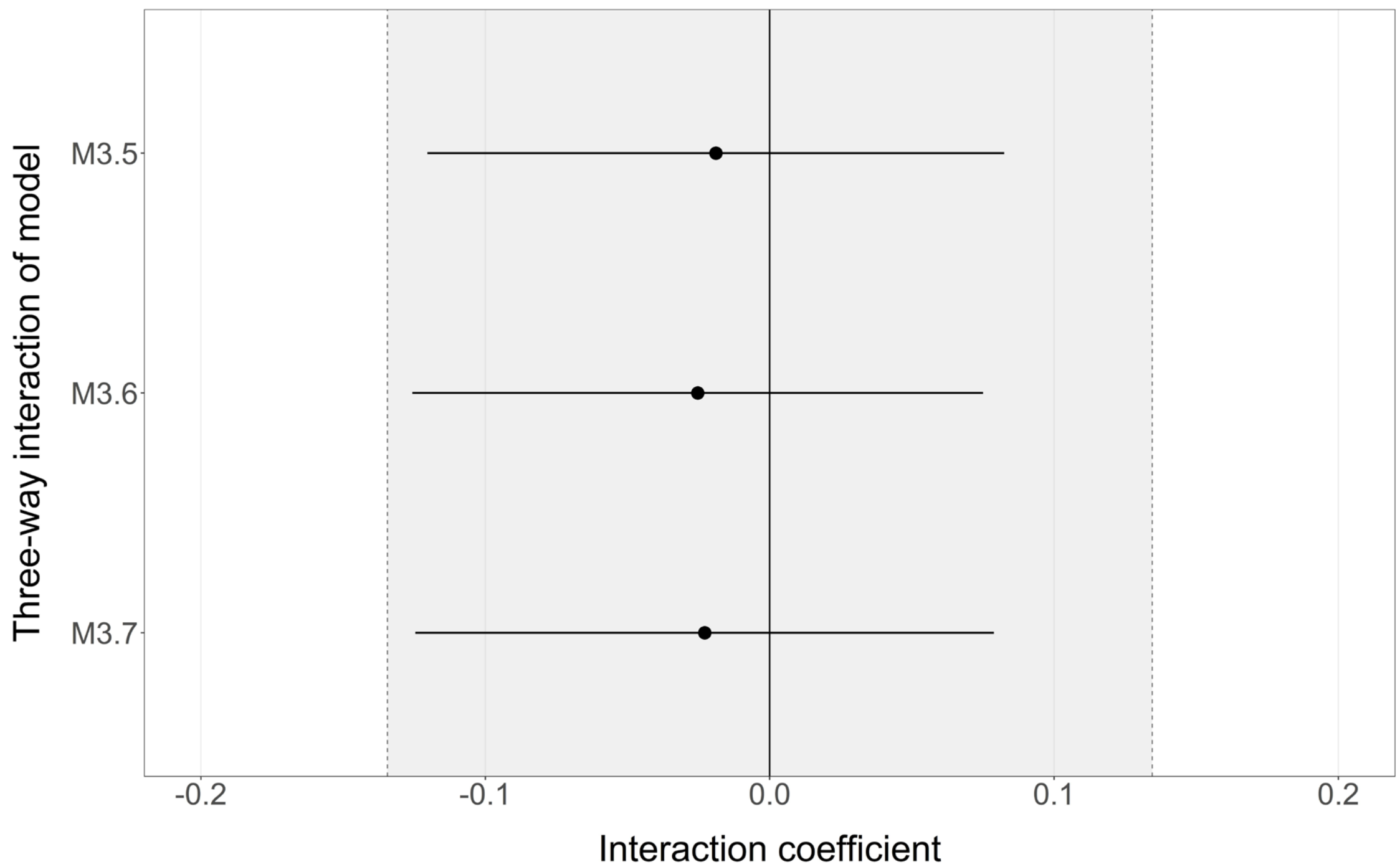

Figure S9: ***Equivalence testing of three-way interaction coefficients across models (M3.5–M3.7).*** *Points represent estimated three-way interaction coefficients for the interaction between political affiliation, EWE exposure, and subjective attribution of EWE to climate change predicting ΔDuration across Models M3.5–M3.7 (N = 1,451 participants), and horizontal lines indicate 95% confidence intervals. The shaded area denotes the region of practical equivalence (ROPE; ±0.1345), based on the smallest effect size of interest (SESOI).*

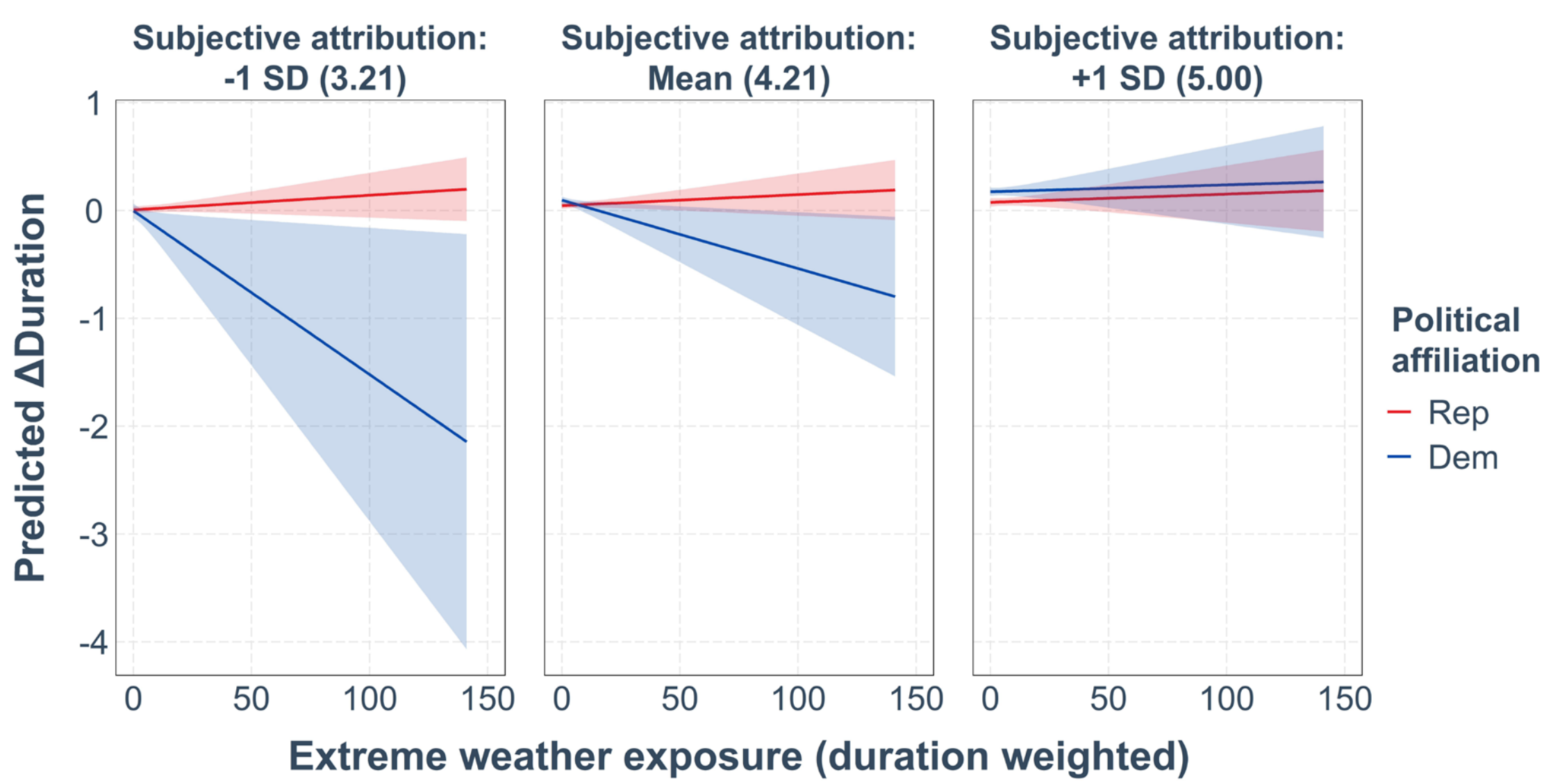

Figure S10: ***Three-way interaction between political affiliation, duration-weighted EWE exposure, and subjective attribution on ΔDuration.*** *Predicted values are based on Model M3.8 (N = 1,451 participants). Panels display predicted ΔDuration as a function of duration-weighted EWE exposure at low (−1 SD), mean, and high (+1 SD) levels of subjective attribution of EWE to climate change (bounded by the observed range), separately for Republicans and Democrats. Red and blue lines represent predicted values for Republicans and Democrats, respectively. Shaded areas represent 95% confidence intervals.*

1.3.1.1. Four-way Interactions with County-Level Characteristics on ΔDuration

We additionally explored whether county-level characteristics moderate the interaction between political affiliation, EWE exposure and subjective attribution of EWE to climate change. Four-way interactions with population density, median household income, and county-level PVI were not statistically significant. Full results are reported in Table S14 and Figure S11.

	M3_popDensity		
Predictors	*Estimates*	*CI*	*p*
(Intercept)	-0.14	-0.29 – 0.02	0.077
polAff bin str [Dem]	-0.32	-0.62 – -0.02	**0.037**
nEpisodesIsGreaterThanZeroTRUE	0.14	-0.11 – 0.40	0.267
EWE subjAttr mean	0.05	0.01 – 0.08	**0.013**
population density peopPerSqKilometer	0.32	-0.07 – 0.72	0.105
abnormality final	0.00	-0.02 – 0.02	0.887
sum damage	0.00	-0.02 – 0.02	0.743
sum injuries	0.01	-0.01 – 0.03	0.506
sum deaths	0.01	-0.01 – 0.03	0.592
recency index	-0.17	-0.43 – 0.10	0.216
countyIncome	-0.02	-0.04 – 0.00	0.106
countyPvi	-0.02	-0.05 – 0.00	0.052
polAff bin str [Dem] × nEpisodesIsGreaterThanZeroTRUE	0.08	-0.51 – 0.67	0.792
polAff bin str [Dem] × EWE subjAttr mean	0.08	0.02 – 0.15	**0.013**
nEpisodesIsGreaterThanZeroTRUE × EWE subjAttr mean	-0.03	-0.09 – 0.03	0.293
polAff bin str [Dem] × population density peopPerSqKilometer	-0.37	-0.79 – 0.05	0.084
nEpisodesIsGreaterThanZeroTRUE × population density peopPerSqKilometer	-0.18	-0.89 – 0.53	0.612
EWE subjAttr mean × population density peopPerSqKilometer	-0.07	-0.16 – 0.01	0.102
(polAff bin str [Dem] × nEpisodesIsGreaterThanZeroTRUE) × EWE subjAttr mean	-0.02	-0.15 – 0.11	0.749
(polAff bin str [Dem] × nEpisodesIsGreaterThanZeroTRUE) × population density peopPerSqKilometer	0.09	-1.66 – 1.83	0.922
(polAff bin str [Dem] × EWE subjAttr mean) × population density peopPerSqKilometer	0.09	-0.00 – 0.19	0.051
(nEpisodesIsGreaterThanZeroTRUE × EWE subjAttr mean) × population density peopPerSqKilometer	0.04	-0.12 – 0.20	0.619
(polAff bin str [Dem] × nEpisodesIsGreaterThanZeroTRUE × EWE subjAttr mean) × population density peopPerSqKilometer	-0.02	-0.40 – 0.36	0.917
**Random Effects**			
σ^2	0.29		
$\tau_{00\ subject}$	0.13		
$\tau_{00\ trialNum\_fixed}$	0.00		
ICC	0.31		
$N_{subject}$	1451		
$N_{trialNum\_fixed}$	25		
Observations	34294		
Marginal R^2 / Conditional R^2	0.018 / 0.319		

	M3_countyIncome		
Predictors	*Estimates*	*CI*	*p*
(Intercept)	-0.21	-0.34 – -0.08	**0.001**
polAff bin str [Dem]	-0.27	-0.55 – 0.02	0.066
nEpisodesIsGreaterThanZeroTRUE	0.22	0.01 – 0.42	**0.037**
EWE subjAttr mean	0.06	0.03 – 0.09	**<0.001**
countyIncome	0.00	-0.14 – 0.14	0.987
abnormality final	0.00	-0.02 – 0.02	0.993
sum damage	0.00	-0.02 – 0.02	0.746
sum injuries	0.01	-0.01 – 0.03	0.510
sum deaths	0.01	-0.01 – 0.03	0.574
recency index	-0.16	-0.43 – 0.10	0.226
population density peopPerSqKilometer	0.02	0.00 – 0.05	**0.039**
countyPvi	-0.03	-0.05 – -0.00	**0.043**
polAff bin str [Dem] × nEpisodesIsGreaterThanZeroTRUE	0.08	-0.38 – 0.54	0.738
polAff bin str [Dem] × EWE subjAttr mean	0.07	0.01 – 0.14	**0.025**
nEpisodesIsGreaterThanZeroTRUE × EWE subjAttr mean	-0.05	-0.10 – 0.00	0.055
polAff bin str [Dem] × countyIncome	-0.24	-0.50 – 0.02	0.065
nEpisodesIsGreaterThanZeroTRUE × countyIncome	0.08	-0.14 – 0.29	0.485
EWE subjAttr mean × countyIncome	-0.01	-0.04 – 0.02	0.534
(polAff bin str [Dem] × nEpisodesIsGreaterThanZeroTRUE) × EWE subjAttr mean	-0.02	-0.12 – 0.08	0.687
(polAff bin str [Dem] × nEpisodesIsGreaterThanZeroTRUE) × countyIncome	0.31	-0.19 – 0.82	0.226
(polAff bin str [Dem] × EWE subjAttr mean) × countyIncome	0.06	0.01 – 0.12	**0.032**
(nEpisodesIsGreaterThanZeroTRUE × EWE subjAttr mean) × countyIncome	-0.01	-0.07 – 0.04	0.692
(polAff bin str [Dem] × nEpisodesIsGreaterThanZeroTRUE × EWE subjAttr mean) × countyIncome	-0.08	-0.19 – 0.04	0.185
**Random Effects**			
σ^2		0.29	
$\tau_{00\ subject}$		0.13	
$\tau_{00\ trialNum\_fixed}$		0.00	
ICC		0.31	
$N_{subject}$		1451	
$N_{trialNum\_fixed}$		25	
Observations		34294	
Marginal R^2 / Conditional R^2		0.018 / 0.319	

	M3_countyPvi		
Predictors	*Estimates*	*CI*	*p*
(Intercept)	-0.27	-0.42 – -0.12	**<0.001**
polAff bin str [Dem]	-0.21	-0.50 – 0.09	0.173
nEpisodesIsGreaterThanZeroTRUE	0.29	0.06 – 0.51	**0.013**
EWE subjAttr mean	0.07	0.04 – 0.11	**<0.001**
countyPvi	-0.09	-0.22 – 0.04	0.174
abnormality final	-0.00	-0.02 – 0.02	0.849
sum damage	0.00	-0.02 – 0.02	0.902
sum injuries	0.01	-0.01 – 0.03	0.595
sum deaths	0.00	-0.02 – 0.02	0.731
recency index	-0.13	-0.39 – 0.13	0.338
population density peopPerSqKilometer	0.03	0.00 – 0.05	**0.023**
countyIncome	-0.02	-0.04 – 0.01	0.166
polAff bin str [Dem] × nEpisodesIsGreaterThanZeroTRUE	-0.06	-0.54 – 0.41	0.791
polAff bin str [Dem] × EWE subjAttr mean	0.06	-0.00 – 0.13	0.065
nEpisodesIsGreaterThanZeroTRUE × EWE subjAttr mean	-0.06	-0.11 – -0.01	**0.026**
polAff bin str [Dem] × countyPvi	-0.01	-0.27 – 0.26	0.947
nEpisodesIsGreaterThanZeroTRUE × countyPvi	0.15	-0.05 – 0.36	0.142
EWE subjAttr mean × countyPvi	0.01	-0.03 – 0.04	0.734
(polAff bin str [Dem] × nEpisodesIsGreaterThanZeroTRUE) × EWE subjAttr mean	0.00	-0.10 – 0.11	0.928
(polAff bin str [Dem] × nEpisodesIsGreaterThanZeroTRUE) × countyPvi	-0.24	-0.68 – 0.20	0.284
(polAff bin str [Dem] × EWE subjAttr mean) × countyPvi	0.01	-0.05 – 0.07	0.672
(nEpisodesIsGreaterThanZeroTRUE × EWE subjAttr mean) × countyPvi	-0.03	-0.08 – 0.02	0.301
(polAff bin str [Dem] × nEpisodesIsGreaterThanZeroTRUE × EWE subjAttr mean) × countyPvi	0.06	-0.04 – 0.16	0.268
**Random Effects**			
σ^2	0.29		
$\tau_{00\ subject}$	0.13		
$\tau_{00\ trialNum\_fixed}$	0.00		
ICC	0.31		
$N_{subject}$	1451		
$N_{trialNum\_fixed}$	25		
Observations	34294		
Marginal R^2 / Conditional R^2	0.019 / 0.319		

Table S14: ***Mixed-effects models predicting ΔDuration including four-way interactions with county-level characteristics (M3).*** *ΔDuration (deltaDuration) represents relative dwell time spent on carbon information compared to bonus information. Random intercepts were included for participants (subject) and trials (trialNum_fixed) (N = 1,451 participants). Models included a four-way interaction between political affiliation (polAff_bin_str), EWE exposure (nEpisodesIsGreaterThanZeroTRUE), subjective attribution of EWE to climate change (EWE_subjAttr_mean), and one county-level characteristic: population density (population_density_peopPerSqKilometer), median household income (countyIncome), or Partisan Voting Index (countyPvi), along with all corresponding lower-order terms. Each model controlled for county-level indicators related to EWE severity and impacts, specifically the abnormality score for the number of EWE (abnormality_final), the amount of financial damage (sum_damage), and the number of injuries (sum_injuries) and deaths (sum_deaths) associated with each episode. The models further controlled for the temporal proximity of the most recent extreme weather episode (recency_index) and the two remaining county-level characteristics not included in the interaction term. All variables (except polAff_bin_str, nEpisodesIsGreaterThanZeroTRUE, EWE_subjAttr_mean and recency_index) were z-standardized prior to analysis. Estimates represent unstandardized beta coefficients. Confidence intervals are 95%. Statistically significant effects (p < .05) are shown in bold.*

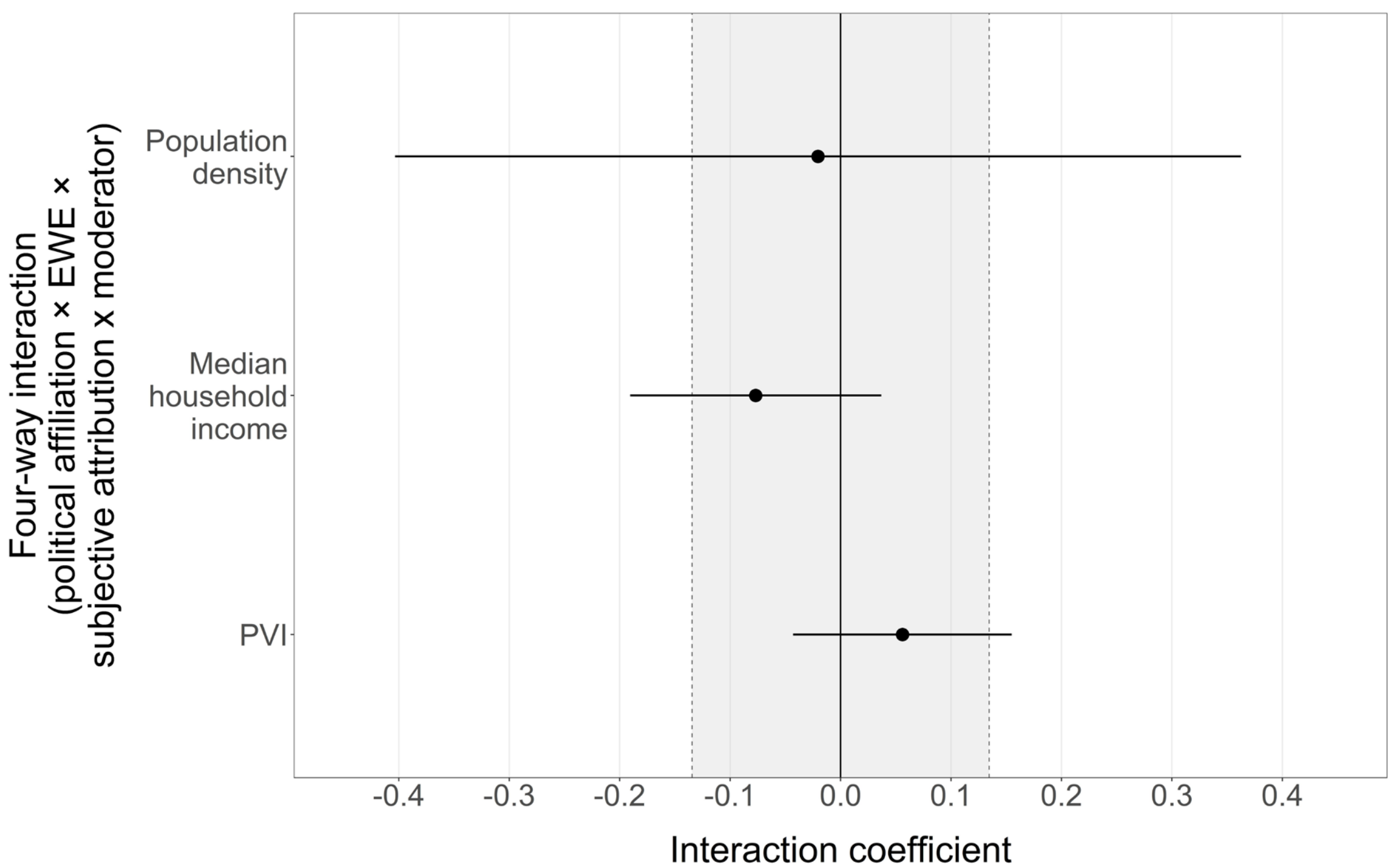

Figure S11: ***Equivalence testing of four-way interaction coefficients across models with county-level characteristics (M3).*** *Points represent estimated four-way interaction coefficients for the interaction between political affiliation, EWE exposure, subjective attribution of EWE to climate change, and county-level characteristics predicting ΔDuration (N = 1,451 participants). County-level characteristics include population density, median household income, and Partisan Voting Index. Horizontal lines indicate 95% confidence intervals. The shaded area denotes the region of practical equivalence (ROPE; ±0.1345), based on the smallest effect size of interest (SESOI).*

1.3.1.2. Sensitivity Analysis for Extreme Weather Events

We additionally conducted preregistered sensitivity analyses testing whether the three-way interaction between political affiliation, EWE exposure, and subjective attribution depends on the time window used to measure exposure (1–12 months prior to study completion). Across all time windows, the three-way interaction remained statistically non-significant (Table S15). As shown in Figure S12, the confidence intervals of all interaction terms lie entirely within the ROPE (±0.1143), indicating practical equivalence.

	M3.3_30d			M3.3_60d			M3.3_90d			M3.3_120d		
Predictors	*Estimates*	*CI*	*p*	*Estimates*	*CI*	*p*	*Estimates*	*CI*	*p*	*Estimates*	*CI*	*p*
(Intercept)	-0.17	-0.28 – -0.06	**0.002**	-0.19	-0.30 – -0.07	**0.002**	-0.19	-0.31 – -0.07	**0.002**	-0.21	-0.33 – -0.08	**0.001**
polAff bin str [Dem]	-0.19	-0.45 – 0.08	0.165	-0.14	-0.42 – 0.14	0.327	-0.12	-0.42 – 0.17	0.409	-0.06	-0.37 – 0.25	0.702
nEpisodes	0.06	0.00 – 0.12	**0.041**	0.04	0.00 – 0.07	**0.036**	0.03	0.00 – 0.05	**0.034**	0.02	0.00 – 0.05	**0.025**
EWE subjAttr mean	0.05	0.03 – 0.08	**<0.001**	0.06	0.03 – 0.08	**<0.001**	0.06	0.03 – 0.08	**<0.001**	0.06	0.03 – 0.09	**<0.001**
polAff bin str [Dem] × nEpisodes	-0.08	-0.27 – 0.12	0.444	-0.06	-0.15 – 0.04	0.249	-0.05	-0.12 – 0.03	0.234	-0.06	-0.12 – 0.01	0.096
polAff bin str [Dem] × EWE subjAttr mean	0.05	-0.00 – 0.11	0.067	0.05	-0.02 – 0.11	0.153	0.04	-0.02 – 0.11	0.182	0.03	-0.04 – 0.10	0.370
nEpisodes × EWE subjAttr mean	-0.01	-0.03 – 0.00	0.052	-0.01	-0.02 – 0.00	0.050	-0.01	-0.01 – 0.00	0.071	-0.00	-0.01 – 0.00	0.081
(polAff bin str [Dem] × nEpisodes) × EWE subjAttr mean	0.02	-0.03 – 0.06	0.492	0.01	-0.01 – 0.03	0.295	0.01	-0.01 – 0.02	0.319	0.01	-0.00 – 0.02	0.141
**Random Effects**												
σ^2		0.29			0.29			0.29			0.29	
τ_{00}		0.13_{subject}			0.13_{subject}			0.13_{subject}			0.13_{subject}	
		$0.00_{\text{trialNum\_fixed}}$			$0.00_{\text{trialNum\_fixed}}$			$0.00_{\text{trialNum\_fixed}}$			$0.00_{\text{trialNum\_fixed}}$	
ICC		0.31			0.31			0.31			0.31	
N		1451_{subject}			1451_{subject}			1451_{subject}			1451_{subject}	
		$25_{\text{trialNum\_fixed}}$			$25_{\text{trialNum\_fixed}}$			$25_{\text{trialNum\_fixed}}$			$25_{\text{trialNum\_fixed}}$	
Observations		34294			34294			34294			34294	
Marginal R^2 / Conditional R^2		0.013 / 0.317			0.013 / 0.317			0.013 / 0.317			0.013 / 0.317	

	M3.3_150d			M3.3_180d			M3.3_210d			M3.3_240d		
Predictors	*Estimates*	*CI*	*p*	*Estimates*	*CI*	*p*	*Estimates*	*CI*	*p*	*Estimates*	*CI*	*p*
(Intercept)	-0.22	-0.35 – -0.09	**0.001**	-0.22	-0.35 – -0.08	**0.002**	-0.22	-0.35 – -0.09	**0.001**	-0.21	-0.35 – -0.08	**0.002**
polAff bin str [Dem]	-0.03	-0.35 – 0.28	0.831	-0.03	-0.35 – 0.29	0.836	-0.01	-0.34 – 0.31	0.935	-0.04	-0.36 – 0.29	0.826
nEpisodes	0.03	0.00 – 0.05	**0.016**	0.02	0.00 – 0.04	**0.031**	0.02	0.00 – 0.04	**0.027**	0.02	0.00 – 0.04	**0.039**
EWE subjAttr mean	0.06	0.03 – 0.09	**<0.001**	0.06	0.03 – 0.09	**<0.001**	0.06	0.03 – 0.09	**<0.001**	0.06	0.03 – 0.09	**<0.001**
polAff bin str [Dem] × nEpisodes	-0.05	-0.11 – 0.01	0.079	-0.05	-0.11 – 0.01	0.083	-0.05	-0.11 – 0.00	0.061	-0.05	-0.10 – 0.01	0.093
polAff bin str [Dem] × EWE subjAttr mean	0.03	-0.04 – 0.10	0.473	0.03	-0.05 – 0.10	0.482	0.02	-0.05 – 0.09	0.576	0.03	-0.05 – 0.10	0.493
nEpisodes × EWE subjAttr mean	-0.00	-0.01 – 0.00	0.065	-0.00	-0.01 – 0.00	0.081	-0.00	-0.01 – 0.00	0.061	-0.00	-0.01 – 0.00	0.081
(polAff bin str [Dem] × nEpisodes) × EWE subjAttr mean	0.01	-0.00 – 0.02	0.116	0.01	-0.00 – 0.02	0.119	0.01	-0.00 – 0.02	0.086	0.01	-0.00 – 0.02	0.126
**Random Effects**												
σ^2	0.29			0.29			0.29			0.29		
τ_{00}	0.13 subject			0.13 subject			0.13 subject			0.13 subject		
	0.00 trialNum_fixed			0.00 trialNum_fixed			0.00 trialNum_fixed			0.00 trialNum_fixed		
ICC	0.31			0.31			0.31			0.31		
N	1451 subject			1451 subject			1451 subject			1451 subject		
	25 trialNum_fixed			25 trialNum_fixed			25 trialNum_fixed			25 trialNum_fixed		
Observations	34294			34294			34294			34294		
Marginal R^2 / Conditional R^2	0.013 / 0.317			0.013 / 0.317			0.013 / 0.317			0.013 / 0.317		

	M3.3_270d			M3.3_300d			M3.3_330d			M3.3_360d		
Predictors	*Estimates*	*CI*	*p*	*Estimates*	*CI*	*p*	*Estimates*	*CI*	*p*	*Estimates*	*CI*	*p*
(Intercept)	-0.20	-0.34 – -0.06	**0.004**	-0.19	-0.33 – -0.05	**0.008**	-0.18	-0.32 – -0.04	**0.010**	-0.18	-0.32 – -0.04	**0.014**
polAff bin str [Dem]	-0.05	-0.38 – 0.27	0.744	-0.07	-0.40 – 0.25	0.652	-0.06	-0.39 – 0.26	0.698	-0.08	-0.40 – 0.25	0.644
nEpisodes	0.02	-0.00 – 0.04	0.072	0.01	-0.00 – 0.03	0.147	0.01	-0.00 – 0.03	0.165	0.01	-0.01 – 0.03	0.218
EWE subjAttr mean	0.06	0.02 – 0.09	**0.001**	0.05	0.02 – 0.09	**0.002**	0.05	0.02 – 0.09	**0.003**	0.05	0.02 – 0.09	**0.004**
polAff bin str [Dem] × nEpisodes	-0.04	-0.10 – 0.01	0.119	-0.04	-0.09 – 0.01	0.164	-0.04	-0.09 – 0.01	0.136	-0.03	-0.08 – 0.01	0.163
polAff bin str [Dem] × EWE subjAttr mean	0.03	-0.04 – 0.10	0.443	0.03	-0.04 – 0.11	0.377	0.03	-0.04 – 0.10	0.393	0.03	-0.04 – 0.11	0.351
nEpisodes × EWE subjAttr mean	-0.00	-0.01 – 0.00	0.125	-0.00	-0.01 – 0.00	0.230	-0.00	-0.01 – 0.00	0.273	-0.00	-0.01 – 0.00	0.342
(polAff bin str [Dem] × nEpisodes) × EWE subjAttr mean	0.01	-0.00 – 0.02	0.151	0.01	-0.00 – 0.02	0.200	0.01	-0.00 – 0.02	0.179	0.01	-0.00 – 0.02	0.212
**Random Effects**												
σ^2	0.29			0.29			0.29			0.29		
τ_{00}	0.13 subject			0.13 subject			0.13 subject			0.13 subject		
	0.00 trialNum_fixed			0.00 trialNum_fixed			0.00 trialNum_fixed			0.00 trialNum_fixed		
ICC	0.31			0.31			0.31			0.31		
N	1451 subject			1451 subject			1451 subject			1451 subject		
	25 trialNum_fixed			25 trialNum_fixed			25 trialNum_fixed			25 trialNum_fixed		
Observations	34294			34294			34294			34294		
Marginal R^2 / Conditional R^2	0.013 / 0.317			0.012 / 0.317			0.012 / 0.317			0.012 / 0.317		

Table S15: ***Sensitivity analyses: Mixed-effects models predicting ΔDuration across different EWE time windows (M3).*** *ΔDuration (deltaDuration) represents relative dwell time spent on carbon information compared to bonus information. Random intercepts were included for participants (subject) and trials (trialNum_fixed) (N = 1,451 participants). The models included political affiliation (polAff_bin_str, subject-level binary variable with 0 = Republican and 1 = Democrat), the number of EWE a participant experienced (nEpisodes), subjective attribution and their interaction. Models are calculated for different time windows (30-360 days). Estimates represent unstandardized beta coefficients. Confidence intervals are 95%. Statistically significant effects (p < .05) are shown in bold.*

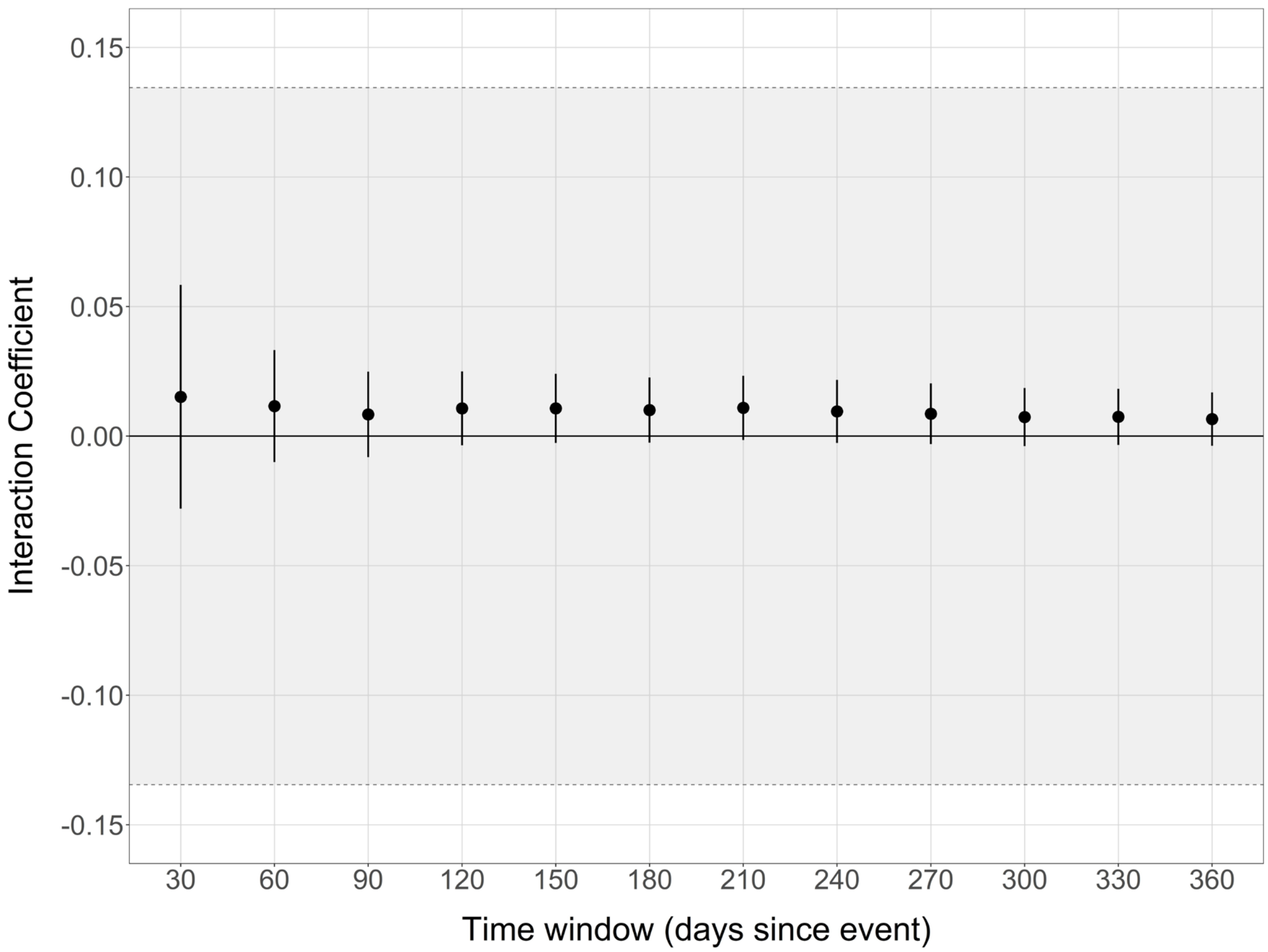

Figure S12: ***Equivalence testing of interaction coefficients across different EWE time windows (M3).*** *Points represent estimated three-way interaction coefficients for the interaction between political affiliation, the number of EWEs a participant experienced, and subjective attribution of EWE to climate change predicting ΔDuration across models using different EWE time windows (30–360 days preceding measurement; N = 1,451 participants). Vertical lines indicate 95% confidence intervals. The shaded area denotes the region of practical equivalence (ROPE; ±0.1345), based on the smallest effect size of interest (SESOI).*

In a second set of sensitivity analyses (A3.2), we repeated the models separately for each EWE type. Results revealed some heterogeneity across event types and exposure windows. The three-way interaction between political affiliation, exposure, and subjective attribution was statistically significant for strong wind events from exposure windows of 120 days onward (Figure S13). For coastal floods, the interaction was significant for intermediate exposure windows (120–330 days) but not for longer windows (Figure S14). For the remaining event types, the three-way interaction was not statistically significant. Full results are reported in Table S16.

	M3.3_Coastal Flood_30d			M3.3_Drought_30d			M3.3_Excessive Heat_30d			M3.3_Flash Flood_30d		
Predictors	*Estimates*	*CI*	*p*	*Estimates*	*CI*	*p*	*Estimates*	*CI*	*p*	*Estimates*	*CI*	*p*
(Intercept)	-0.11	-0.20 – -0.02	**0.020**	-0.12	-0.22 – -0.03	**0.012**	-0.12	-0.22 – -0.02	**0.022**	-0.15	-0.26 – -0.05	**0.003**
polAff bin str [Dem]	-0.26	-0.48 – -0.04	**0.023**	-0.25	-0.47 – -0.02	**0.030**	-0.23	-0.46 – 0.00	0.051	-0.23	-0.47 – 0.02	0.073
nEvents	-2.60	-6.87 – 1.68	0.233	0.36	-0.13 – 0.86	0.147	0.02	-0.11 – 0.15	0.763	0.12	0.00 – 0.23	**0.046**
EWE subjAttr mean	0.04	0.02 – 0.06	**0.001**	0.04	0.02 – 0.06	**0.001**	0.04	0.01 – 0.06	**0.002**	0.05	0.03 – 0.08	**<0.001**
polAff bin str [Dem] × nEvents	1.03	-0.01 – 2.07	0.053	0.71	-6.07 – 7.48	0.838	-0.13	-0.55 – 0.29	0.546	-0.07	-0.40 – 0.26	0.682
polAff bin str [Dem] × EWE subjAttr mean	0.07	0.02 – 0.12	**0.006**	0.07	0.02 – 0.12	**0.008**	0.07	0.01 – 0.12	**0.014**	0.06	0.01 – 0.12	**0.031**
nEvents × EWE subjAttr mean	0.46	-0.44 – 1.36	0.316	-0.11	-0.26 – 0.03	0.130	-0.00	-0.03 – 0.03	0.995	-0.03	-0.06 – -0.00	**0.022**
(polAff bin str [Dem] × nEvents) × EWE subjAttr mean				-0.12	-1.53 – 1.28	0.866	0.02	-0.07 – 0.11	0.677	0.02	-0.05 – 0.09	0.603
**Random Effects**												
σ^2	0.29			0.29			0.29			0.29		
τ_{00}	0.13 $_{subject}$			0.13 $_{subject}$			0.13 $_{subject}$			0.13 $_{subject}$		
	0.00 $_{trialNum\_fixed}$			0.00 $_{trialNum\_fixed}$			0.00 $_{trialNum\_fixed}$			0.00 $_{trialNum\_fixed}$		
ICC	0.31			0.31			0.31			0.31		
N	1451 $_{subject}$			1451 $_{subject}$			1451 $_{subject}$			1451 $_{subject}$		
	25 $_{trialNum\_fixed}$			25 $_{trialNum\_fixed}$			25 $_{trialNum\_fixed}$			25 $_{trialNum\_fixed}$		
Observations	34294			34294			34294			34294		
Marginal R^2 / Conditional R^2	0.012 / 0.317			0.012 / 0.317			0.012 / 0.317			0.013 / 0.317		

	M3.3_Hail_30d			M3.3_Strong Wind_30d			M3.3_Tornado_30d			M3.3_Wildfire_30d		
Predictors	*Estimates*	*CI*	*p*	*Estimates*	*CI*	*p*	*Estimates*	*CI*	*p*	*Estimates*	*CI*	*p*
(Intercept)	-0.10	-0.20 – -0.00	**0.045**	-0.11	-0.21 – -0.02	**0.020**	-0.11	-0.20 – -0.02	**0.021**	-0.12	-0.21 – -0.02	**0.016**
polAff bin str [Dem]	-0.30	-0.53 – -0.07	**0.012**	-0.25	-0.47 – -0.03	**0.025**	-0.26	-0.48 – -0.03	**0.024**	-0.23	-0.46 – -0.01	**0.039**
nEvents	-0.13	-0.42 – 0.16	0.391	0.75	-1.91 – 3.41	0.579	-0.48	-7.26 – 6.31	0.890	0.07	-0.08 – 0.22	0.358
EWE subjAttr mean	0.03	0.01 – 0.06	**0.004**	0.04	0.02 – 0.06	**0.001**	0.04	0.02 – 0.06	**0.001**	0.04	0.02 – 0.06	**0.001**
polAff bin str [Dem] × nEvents	0.44	-0.26 – 1.14	0.217	2.01	-11.33 – 15.36	0.767	-0.15	-1.53 – 1.23	0.836	-0.48	-1.25 – 0.30	0.229
polAff bin str [Dem] × EWE subjAttr mean	0.08	0.03 – 0.13	**0.003**	0.07	0.02 – 0.12	**0.006**	0.07	0.02 – 0.12	**0.006**	0.06	0.01 – 0.11	**0.011**
nEvents × EWE subjAttr mean	0.03	-0.04 – 0.10	0.356	-0.27	-1.31 – 0.77	0.610	0.12	-1.44 – 1.68	0.877	-0.02	-0.07 – 0.02	0.356
(polAff bin str [Dem] × nEvents) × EWE subjAttr mean	-0.10	-0.26 – 0.05	0.200	-0.40	-3.71 – 2.90	0.811				0.10	-0.07 – 0.28	0.251
**Random Effects**												
σ^2	0.29			0.29			0.29			0.29		
τ_{00}	0.13 $_{subject}$			0.13 $_{subject}$			0.13 $_{subject}$			0.13 $_{subject}$		
	0.00 $_{trialNum\_fixed}$			0.00 $_{trialNum\_fixed}$			0.00 $_{trialNum\_fixed}$			0.00 $_{trialNum\_fixed}$		
ICC	0.31			0.31			0.31			0.31		
N	1451 $_{subject}$			1451 $_{subject}$			1451 $_{subject}$			1451 $_{subject}$		
	25 $_{trialNum\_fixed}$			25 $_{trialNum\_fixed}$			25 $_{trialNum\_fixed}$			25 $_{trialNum\_fixed}$		
Observations	34294			34294			34294			34294		
Marginal R^2 / Conditional R^2	0.012 / 0.317			0.012 / 0.317			0.011 / 0.317			0.012 / 0.317		

	M3.3_Coastal Flood_60d			M3.3_Drought_60d			M3.3_Excessive Heat_60d			M3.3_Flash Flood_60d		
Predictors	*Estimates*	*CI*	*p*	*Estimates*	*CI*	*p*	*Estimates*	*CI*	*p*	*Estimates*	*CI*	*p*
(Intercept)	-0.11	-0.20 – -0.02	**0.020**	-0.12	-0.22 – -0.02	**0.016**	-0.13	-0.23 – -0.03	**0.014**	-0.18	-0.29 – -0.07	**0.001**
polAff bin str [Dem]	-0.26	-0.48 – -0.04	**0.023**	-0.24	-0.47 – -0.02	**0.032**	-0.23	-0.46 – 0.01	0.059	-0.22	-0.48 – 0.05	0.105
nEvents	-2.60	-6.87 – 1.68	0.233	0.05	-0.15 – 0.25	0.646	0.03	-0.04 – 0.11	0.387	0.08	0.01 – 0.14	**0.020**
EWE subjAttr mean	0.04	0.02 – 0.06	**0.001**	0.04	0.02 – 0.06	**0.001**	0.04	0.02 – 0.07	**0.001**	0.06	0.03 – 0.08	**<0.001**
polAff bin str [Dem] × nEvents	1.03	-0.01 – 2.07	0.053	-0.83	-2.56 – 0.89	0.342	-0.05	-0.24 – 0.13	0.568	-0.04	-0.20 – 0.12	0.638
polAff bin str [Dem] × EWE subjAttr mean	0.07	0.02 – 0.12	**0.006**	0.07	0.02 – 0.12	**0.008**	0.06	0.01 – 0.12	**0.017**	0.06	0.00 – 0.12	**0.048**
nEvents × EWE subjAttr mean	0.46	-0.44 – 1.36	0.316	0.00	-0.05 – 0.06	0.898	-0.01	-0.03 – 0.01	0.506	-0.02	-0.04 – -0.00	**0.014**
(polAff bin str [Dem] × nEvents) × EWE subjAttr mean				0.15	-0.21 – 0.51	0.421	0.01	-0.03 – 0.05	0.694	0.01	-0.03 – 0.05	0.563
**Random Effects**												
σ^2	0.29			0.29			0.29			0.29		
τ_{00}	0.13 $_{subject}$			0.13 $_{subject}$			0.13 $_{subject}$			0.13 $_{subject}$		
	0.00 $_{trialNum\_fixed}$			0.00 $_{trialNum\_fixed}$			0.00 $_{trialNum\_fixed}$			0.00 $_{trialNum\_fixed}$		
ICC	0.31			0.31			0.31			0.31		
N	1451 $_{subject}$			1451 $_{subject}$			1451 $_{subject}$			1451 $_{subject}$		
	25 $_{trialNum\_fixed}$			25 $_{trialNum\_fixed}$			25 $_{trialNum\_fixed}$			25 $_{trialNum\_fixed}$		
Observations	34294			34294			34294			34294		
Marginal R^2 / Conditional R^2	0.012 / 0.317			0.013 / 0.317			0.012 / 0.317			0.013 / 0.317		

	M3.3_Hail_60d			M3.3_Strong Wind_60d			M3.3_Tornado_60d			M3.3_Wildfire_60d		
Predictors	*Estimates*	*CI*	*p*	*Estimates*	*CI*	*p*	*Estimates*	*CI*	*p*	*Estimates*	*CI*	*p*
(Intercept)	-0.11	-0.21 – -0.01	**0.030**	-0.11	-0.21 – -0.02	**0.020**	-0.11	-0.20 – -0.01	**0.023**	-0.11	-0.21 – -0.02	**0.018**
polAff bin str [Dem]	-0.26	-0.50 – -0.02	**0.034**	-0.25	-0.47 – -0.03	**0.025**	-0.25	-0.48 – -0.03	**0.027**	-0.23	-0.46 – -0.01	**0.041**
nEvents	-0.01	-0.12 – 0.11	0.930	0.75	-1.90 – 3.40	0.580	-0.06	-0.70 – 0.58	0.860	0.03	-0.10 – 0.17	0.619
EWE subjAttr mean	0.04	0.01 – 0.06	**0.002**	0.04	0.02 – 0.06	**0.001**	0.04	0.01 – 0.06	**0.001**	0.04	0.02 – 0.06	**0.001**
polAff bin str [Dem] × nEvents	0.02	-0.39 – 0.44	0.912	2.02	-11.32 – 15.36	0.767	-0.12	-1.72 – 1.48	0.885	-0.28	-0.71 – 0.15	0.196
polAff bin str [Dem] × EWE subjAttr mean	0.07	0.02 – 0.13	**0.009**	0.07	0.02 – 0.12	**0.007**	0.07	0.02 – 0.12	**0.007**	0.06	0.01 – 0.11	**0.013**
nEvents × EWE subjAttr mean	0.00	-0.02 – 0.03	0.845	-0.27	-1.29 – 0.76	0.610	0.03	-0.13 – 0.20	0.682	-0.01	-0.05 – 0.03	0.491
(polAff bin str [Dem] × nEvents) × EWE subjAttr mean	-0.01	-0.10 – 0.08	0.827	-0.41	-3.71 – 2.89	0.809	0.02	-0.34 – 0.38	0.917	0.06	-0.04 – 0.16	0.206
**Random Effects**												
σ^2	0.29			0.29			0.29			0.29		
τ_{00}	0.13 $_{subject}$			0.13 $_{subject}$			0.13 $_{subject}$			0.13 $_{subject}$		
	0.00 $_{trialNum\_fixed}$			0.00 $_{trialNum\_fixed}$			0.00 $_{trialNum\_fixed}$			0.00 $_{trialNum\_fixed}$		
ICC	0.31			0.31			0.31			0.31		
N	1451 $_{subject}$			1451 $_{subject}$			1451 $_{subject}$			1451 $_{subject}$		
	25 $_{trialNum\_fixed}$			25 $_{trialNum\_fixed}$			25 $_{trialNum\_fixed}$			25 $_{trialNum\_fixed}$		
Observations	34294			34294			34294			34294		
Marginal R^2 / Conditional R^2	0.012 / 0.317			0.012 / 0.317			0.012 / 0.317			0.012 / 0.317		

	M3.3_Coastal Flood_90d			M3.3_Drought_90d			M3.3_Excessive Heat_90d			M3.3_Flash Flood_90d		
Predictors	*Estimates*	*CI*	*p*	*Estimates*	*CI*	*p*	*Estimates*	*CI*	*p*	*Estimates*	*CI*	*p*
(Intercept)	-0.11	-0.20 – -0.02	**0.020**	-0.12	-0.22 – -0.02	**0.018**	-0.13	-0.23 – -0.03	**0.011**	-0.17	-0.28 – -0.05	**0.004**
polAff bin str [Dem]	-0.26	-0.48 – -0.03	**0.024**	-0.23	-0.46 – -0.01	**0.041**	-0.21	-0.45 – 0.03	0.080	-0.25	-0.52 – 0.02	0.065
nEvents	-1.14	-5.02 – 2.74	0.565	0.02	-0.07 – 0.11	0.664	0.03	-0.02 – 0.08	0.259	0.05	-0.01 – 0.10	0.090
EWE subjAttr mean	0.04	0.02 – 0.06	**0.001**	0.04	0.01 – 0.06	**0.002**	0.04	0.02 – 0.07	**0.001**	0.05	0.02 – 0.08	**<0.001**
polAff bin str [Dem] × nEvents	0.59	-0.32 – 1.51	0.204	-0.42	-0.96 – 0.12	0.126	-0.06	-0.21 – 0.08	0.391	-0.00	-0.12 – 0.12	0.983
polAff bin str [Dem] × EWE subjAttr mean	0.07	0.02 – 0.12	**0.006**	0.07	0.02 – 0.12	**0.010**	0.06	0.01 – 0.11	**0.024**	0.07	0.01 – 0.13	**0.026**
nEvents × EWE subjAttr mean	0.15	-0.67 – 0.97	0.720	-0.00	-0.02 – 0.02	0.993	-0.01	-0.02 – 0.01	0.339	-0.01	-0.03 – 0.00	0.074
(polAff bin str [Dem] × nEvents) × EWE subjAttr mean				0.08	-0.03 – 0.20	0.165	0.01	-0.02 – 0.04	0.505	0.00	-0.03 – 0.03	0.949
**Random Effects**												
σ^2	0.29			0.29			0.29			0.29		
τ_{00}	0.13 $_{\text{subject}}$			0.13 $_{\text{subject}}$			0.13 $_{\text{subject}}$			0.13 $_{\text{subject}}$		
	0.00 $_{\text{trialNum\_fixed}}$			0.00 $_{\text{trialNum\_fixed}}$			0.00 $_{\text{trialNum\_fixed}}$			0.00 $_{\text{trialNum\_fixed}}$		
ICC	0.31			0.31			0.31			0.31		
N	1451 $_{\text{subject}}$			1451 $_{\text{subject}}$			1451 $_{\text{subject}}$			1451 $_{\text{subject}}$		
	25 $_{\text{trialNum\_fixed}}$			25 $_{\text{trialNum\_fixed}}$			25 $_{\text{trialNum\_fixed}}$			25 $_{\text{trialNum\_fixed}}$		
Observations	34294			34294			34294			34294		
Marginal R^2 / Conditional R^2	0.012 / 0.317			0.013 / 0.317			0.012 / 0.317			0.012 / 0.317		

	M3.3_Hail_90d			M3.3_Strong Wind_90d			M3.3_Tornado_90d			M3.3_Wildfire_90d		
Predictors	*Estimates*	*CI*	*p*	*Estimates*	*CI*	*p*	*Estimates*	*CI*	*p*	*Estimates*	*CI*	*p*
(Intercept)	-0.12	-0.23 – -0.02	**0.018**	-0.13	-0.22 – -0.03	**0.010**	-0.12	-0.21 – -0.02	**0.015**	-0.11	-0.21 – -0.02	**0.019**
polAff bin str [Dem]	-0.26	-0.51 – -0.01	**0.045**	-0.23	-0.45 – -0.00	**0.045**	-0.25	-0.47 – -0.02	**0.032**	-0.23	-0.45 – -0.01	**0.044**
nEvents	0.03	-0.05 – 0.10	0.519	0.39	-0.09 – 0.86	0.116	0.14	-0.30 – 0.57	0.540	0.03	-0.09 – 0.15	0.621
EWE subjAttr mean	0.04	0.01 – 0.06	**0.002**	0.04	0.02 – 0.06	**<0.001**	0.04	0.01 – 0.06	**0.001**	0.04	0.02 – 0.06	**0.001**
polAff bin str [Dem] × nEvents	0.01	-0.27 – 0.30	0.920	-1.93	-4.38 – 0.52	0.123	-0.02	-1.19 – 1.14	0.968	-0.29	-0.70 – 0.12	0.166
polAff bin str [Dem] × EWE subjAttr mean	0.07	0.02 – 0.13	**0.012**	0.06	0.01 – 0.11	**0.013**	0.07	0.02 – 0.12	**0.008**	0.06	0.01 – 0.11	**0.012**
nEvents × EWE subjAttr mean	-0.00	-0.02 – 0.01	0.707	-0.09	-0.24 – 0.06	0.257	0.01	-0.10 – 0.12	0.847	-0.01	-0.04 – 0.03	0.708
(polAff bin str [Dem] × nEvents) × EWE subjAttr mean	-0.01	-0.07 – 0.05	0.813	0.43	-0.15 – 1.01	0.147	-0.01	-0.27 – 0.24	0.912	0.06	-0.03 – 0.16	0.210
**Random Effects**												
σ^2	0.29			0.29			0.29			0.29		
τ_{00}	0.13 subject			0.13 subject			0.13 subject			0.13 subject		
	0.00 trialNum_fixed			0.00 trialNum_fixed			0.00 trialNum_fixed			0.00 trialNum_fixed		
ICC	0.31			0.31			0.31			0.31		
N	1451 subject			1451 subject			1451 subject			1451 subject		
	25 trialNum_fixed			25 trialNum_fixed			25 trialNum_fixed			25 trialNum_fixed		
Observations	34294			34294			34294			34294		
Marginal R^2 / Conditional R^2	0.012 / 0.317			0.013 / 0.317			0.014 / 0.317			0.012 / 0.317		

	M3.3_Coastal Flood_120d			M3.3_Drought_120d			M3.3_Excessive Heat_120d			M3.3_Flash Flood_120d		
Predictors	*Estimates*	*CI*	*p*	*Estimates*	*CI*	*p*	*Estimates*	*CI*	*p*	*Estimates*	*CI*	*p*
(Intercept)	-0.11	-0.21 – -0.02	**0.016**	-0.12	-0.22 – -0.02	**0.017**	-0.13	-0.23 – -0.03	**0.011**	-0.16	-0.28 – -0.05	**0.006**
polAff bin str [Dem]	-0.25	-0.47 – -0.03	**0.028**	-0.23	-0.45 – -0.01	**0.044**	-0.21	-0.45 – 0.02	0.080	-0.23	-0.51 – 0.04	0.094
nEvents	2.49	0.23 – 4.76	**0.031**	0.02	-0.04 – 0.08	0.543	0.03	-0.02 – 0.08	0.267	0.04	-0.01 – 0.08	0.127
EWE subjAttr mean	0.04	0.02 – 0.06	**0.001**	0.04	0.02 – 0.06	**0.001**	0.04	0.02 – 0.07	**0.001**	0.05	0.02 – 0.08	**<0.001**
polAff bin str [Dem] × nEvents	-3.54	-6.69 – -0.40	**0.027**	-0.33	-0.73 – 0.07	0.106	-0.06	-0.21 – 0.08	0.397	-0.01	-0.12 – 0.09	0.805
polAff bin str [Dem] × EWE subjAttr mean	0.07	0.02 – 0.12	**0.008**	0.06	0.01 – 0.11	**0.012**	0.06	0.01 – 0.11	**0.023**	0.06	0.00 – 0.12	**0.039**
nEvents × EWE subjAttr mean	-0.59	-1.13 – -0.05	**0.032**	-0.00	-0.02 – 0.01	0.647	-0.01	-0.02 – 0.01	0.345	-0.01	-0.02 – 0.00	0.123
(polAff bin str [Dem] × nEvents) × EWE subjAttr mean	0.86	0.13 – 1.60	**0.022**	0.07	-0.02 – 0.15	0.122	0.01	-0.02 – 0.04	0.511	0.00	-0.02 – 0.03	0.799
**Random Effects**												
σ^2	0.29			0.29			0.29			0.29		
τ_{00}	0.13 subject			0.13 subject			0.13 subject			0.13 subject		
	0.00 trialNum_fixed			0.00 trialNum_fixed			0.00 trialNum_fixed			0.00 trialNum_fixed		
ICC	0.31			0.31			0.31			0.31		
N	1451 subject			1451 subject			1451 subject			1451 subject		
	25 trialNum_fixed			25 trialNum_fixed			25 trialNum_fixed			25 trialNum_fixed		
Observations	34294			34294			34294			34294		
Marginal R^2 / Conditional R^2	0.013 / 0.317			0.012 / 0.317			0.012 / 0.317			0.012 / 0.317		

	M3.3_Hail_120d			M3.3_Strong Wind_120d			M3.3_Tornado_120d			M3.3_Wildfire_120d		
Predictors	*Estimates*	*CI*	*p*	*Estimates*	*CI*	*p*	*Estimates*	*CI*	*p*	*Estimates*	*CI*	*p*
(Intercept)	-0.16	-0.27 – -0.05	**0.004**	-0.12	-0.22 – -0.03	**0.012**	-0.12	-0.22 – -0.02	**0.021**	-0.12	-0.21 – -0.02	**0.017**
polAff bin str [Dem]	-0.14	-0.41 – 0.12	0.297	-0.21	-0.43 – 0.02	0.072	-0.18	-0.42 – 0.05	0.124	-0.23	-0.45 – -0.00	**0.045**
nEvents	0.05	-0.01 – 0.10	0.092	0.20	-0.14 – 0.55	0.246	0.02	-0.21 – 0.25	0.858	0.04	-0.08 – 0.16	0.511
EWE subjAttr mean	0.05	0.02 – 0.07	**0.001**	0.04	0.02 – 0.06	**0.001**	0.04	0.01 – 0.06	**0.003**	0.04	0.02 – 0.06	**0.001**
polAff bin str [Dem] × nEvents	-0.13	-0.31 – 0.06	0.183	-1.52	-2.77 – -0.28	**0.016**	-0.46	-1.07 – 0.15	0.141	-0.28	-0.68 – 0.12	0.174
polAff bin str [Dem] × EWE subjAttr mean	0.05	-0.01 – 0.11	0.113	0.06	0.01 – 0.11	**0.022**	0.05	0.00 – 0.11	**0.043**	0.06	0.01 – 0.11	**0.014**
nEvents × EWE subjAttr mean	-0.01	-0.02 – 0.01	0.240	-0.05	-0.15 – 0.04	0.243	0.02	-0.04 – 0.08	0.484	-0.01	-0.05 – 0.02	0.511
(polAff bin str [Dem] × nEvents) × EWE subjAttr mean	0.02	-0.02 – 0.06	0.239	0.33	0.05 – 0.61	**0.021**	0.09	-0.05 – 0.22	0.206	0.06	-0.03 – 0.15	0.194
**Random Effects**												
σ^2	0.29			0.29			0.29			0.29		
τ_{00}	0.13 subject			0.13 subject			0.13 subject			0.13 subject		
	0.00 trialNum_fixed			0.00 trialNum_fixed			0.00 trialNum_fixed			0.00 trialNum_fixed		
ICC	0.31			0.31			0.31			0.31		
N	1451 subject			1451 subject			1451 subject			1451 subject		
	25 trialNum_fixed			25 trialNum_fixed			25 trialNum_fixed			25 trialNum_fixed		
Observations	34294			34294			34294			34294		
Marginal R^2 / Conditional R^2	0.013 / 0.317			0.013 / 0.317			0.015 / 0.317			0.012 / 0.317		

	M3.3_Coastal Flood_150d			M3.3_Drought_150d			M3.3_Excessive Heat_150d			M3.3_Flash Flood_150d		
Predictors	*Estimates*	*CI*	*p*	*Estimates*	*CI*	*p*	*Estimates*	*CI*	*p*	*Estimates*	*CI*	*p*
(Intercept)	-0.11	-0.21 – -0.02	**0.016**	-0.12	-0.22 – -0.02	**0.016**	-0.13	-0.23 – -0.03	**0.011**	-0.17	-0.29 – -0.05	**0.005**
polAff bin str [Dem]	-0.25	-0.47 – -0.03	**0.028**	-0.23	-0.46 – -0.01	**0.044**	-0.21	-0.45 – 0.02	0.080	-0.23	-0.51 – 0.05	0.105
nEvents	2.43	0.23 – 4.63	**0.030**	0.02	-0.04 – 0.07	0.525	0.03	-0.02 – 0.08	0.267	0.04	-0.01 – 0.08	0.100
EWE subjAttr mean	0.04	0.02 – 0.06	**0.001**	0.04	0.02 – 0.06	**0.001**	0.04	0.02 – 0.07	**0.001**	0.05	0.02 – 0.08	**<0.001**
polAff bin str [Dem] × nEvents	-3.08	-6.07 – -0.08	**0.044**	-0.25	-0.58 – 0.07	0.126	-0.06	-0.21 – 0.08	0.397	-0.01	-0.12 – 0.09	0.788
polAff bin str [Dem] × EWE subjAttr mean	0.07	0.02 – 0.12	**0.008**	0.06	0.01 – 0.12	**0.012**	0.06	0.01 – 0.11	**0.023**	0.06	0.00 – 0.12	**0.048**
nEvents × EWE subjAttr mean	-0.57	-1.08 – -0.06	**0.028**	-0.00	-0.02 – 0.01	0.630	-0.01	-0.02 – 0.01	0.345	-0.01	-0.02 – 0.00	0.100
(polAff bin str [Dem] × nEvents) × EWE subjAttr mean	0.74	0.06 – 1.43	**0.034**	0.05	-0.02 – 0.12	0.146	0.01	-0.02 – 0.04	0.511	0.00	-0.02 – 0.03	0.761
**Random Effects**												
σ^2	0.29			0.29			0.29			0.29		
τ_{00}	0.13 $_{subject}$			0.13 $_{subject}$			0.13 $_{subject}$			0.13 $_{subject}$		
	0.00 $_{trialNum\_fixed}$			0.00 $_{trialNum\_fixed}$			0.00 $_{trialNum\_fixed}$			0.00 $_{trialNum\_fixed}$		
ICC	0.31			0.31			0.31			0.31		
N	1451 $_{subject}$			1451 $_{subject}$			1451 $_{subject}$			1451 $_{subject}$		
	25 $_{trialNum\_fixed}$			25 $_{trialNum\_fixed}$			25 $_{trialNum\_fixed}$			25 $_{trialNum\_fixed}$		
Observations	34294			34294			34294			34294		
Marginal R^2 / Conditional R^2	0.013 / 0.317			0.012 / 0.317			0.012 / 0.317			0.012 / 0.317		

	M3.3_Hail_150d			M3.3_Strong Wind_150d			M3.3_Tornado_150d			M3.3_Wildfire_150d		
Predictors	*Estimates*	*CI*	*p*	*Estimates*	*CI*	*p*	*Estimates*	*CI*	*p*	*Estimates*	*CI*	*p*
(Intercept)	-0.18	-0.29 – -0.06	**0.003**	-0.13	-0.23 – -0.03	**0.008**	-0.13	-0.24 – -0.03	**0.010**	-0.11	-0.21 – -0.02	**0.019**
polAff bin str [Dem]	-0.16	-0.44 – 0.11	0.246	-0.17	-0.40 – 0.05	0.137	-0.16	-0.40 – 0.08	0.187	-0.22	-0.45 – 0.00	0.054
nEvents	0.05	-0.00 – 0.10	0.073	0.21	-0.07 – 0.48	0.146	0.08	-0.09 – 0.25	0.358	0.02	-0.09 – 0.13	0.721
EWE subjAttr mean	0.05	0.02 – 0.08	**0.001**	0.04	0.02 – 0.07	**<0.001**	0.04	0.02 – 0.07	**0.001**	0.04	0.02 – 0.06	**0.001**
polAff bin str [Dem] × nEvents	-0.07	-0.22 – 0.08	0.344	-1.17	-1.93 – -0.42	**0.002**	-0.49	-1.01 – 0.03	0.065	-0.33	-0.70 – 0.05	0.087
polAff bin str [Dem] × EWE subjAttr mean	0.05	-0.01 – 0.11	0.094	0.05	0.00 – 0.10	**0.049**	0.05	-0.01 – 0.10	0.076	0.06	0.01 – 0.11	**0.017**
nEvents × EWE subjAttr mean	-0.01	-0.02 – 0.00	0.235	-0.05	-0.13 – 0.02	0.135	-0.01	-0.05 – 0.04	0.799	-0.01	-0.03 – 0.02	0.623
(polAff bin str [Dem] × nEvents) × EWE subjAttr mean	0.01	-0.02 – 0.04	0.429	0.26	0.09 – 0.43	**0.003**	0.10	-0.01 – 0.22	0.079	0.07	-0.01 – 0.16	0.096
**Random Effects**												
σ^2	0.29			0.29			0.29			0.29		
τ_{00}	0.13 $_{subject}$			0.13 $_{subject}$			0.13 $_{subject}$			0.13 $_{subject}$		
	0.00 $_{trialNum\_fixed}$			0.00 $_{trialNum\_fixed}$			0.00 $_{trialNum\_fixed}$			0.00 $_{trialNum\_fixed}$		
ICC	0.31			0.31			0.31			0.31		
N	1451 $_{subject}$			1451 $_{subject}$			1451 $_{subject}$			1451 $_{subject}$		
	25 $_{trialNum\_fixed}$			25 $_{trialNum\_fixed}$			25 $_{trialNum\_fixed}$			25 $_{trialNum\_fixed}$		
Observations	34294			34294			34294			34294		
Marginal R^2 / Conditional R^2	0.013 / 0.317			0.014 / 0.317			0.014 / 0.317			0.012 / 0.317		

	M3.3_Coastal Flood_180d			M3.3_Drought_180d			M3.3_Excessive Heat_180d			M3.3_Flash Flood_180d		
Predictors	*Estimates*	*CI*	*p*	*Estimates*	*CI*	*p*	*Estimates*	*CI*	*p*	*Estimates*	*CI*	*p*
(Intercept)	-0.11	-0.21 – -0.02	**0.016**	-0.12	-0.22 – -0.02	**0.017**	-0.13	-0.23 – -0.03	**0.011**	-0.17	-0.29 – -0.05	**0.005**
polAff bin str [Dem]	-0.25	-0.47 – -0.03	**0.028**	-0.23	-0.46 – -0.01	**0.042**	-0.21	-0.45 – 0.02	0.080	-0.23	-0.51 – 0.06	0.115
nEvents	2.43	0.23 – 4.63	**0.030**	0.01	-0.03 – 0.06	0.564	0.03	-0.02 – 0.08	0.267	0.04	-0.01 – 0.08	0.117
EWE subjAttr mean	0.04	0.02 – 0.06	**0.001**	0.04	0.02 – 0.06	**0.001**	0.04	0.02 – 0.07	**0.001**	0.05	0.02 – 0.08	**<0.001**
polAff bin str [Dem] × nEvents	-3.08	-6.07 – -0.08	**0.044**	-0.21	-0.49 – 0.08	0.152	-0.06	-0.21 – 0.08	0.397	-0.02	-0.12 – 0.09	0.768
polAff bin str [Dem] × EWE subjAttr mean	0.07	0.02 – 0.12	**0.008**	0.07	0.01 – 0.12	**0.011**	0.06	0.01 – 0.11	**0.023**	0.06	-0.00 – 0.12	0.055
nEvents × EWE subjAttr mean	-0.57	-1.08 – -0.06	**0.028**	-0.00	-0.01 – 0.01	0.646	-0.01	-0.02 – 0.01	0.345	-0.01	-0.02 – 0.00	0.097
(polAff bin str [Dem] × nEvents) × EWE subjAttr mean	0.74	0.06 – 1.43	**0.034**	0.04	-0.02 – 0.10	0.177	0.01	-0.02 – 0.04	0.511	0.00	-0.02 – 0.03	0.738
**Random Effects**												
σ^2	0.29			0.29			0.29			0.29		
τ_{00}	0.13 $_{subject}$			0.13 $_{subject}$			0.13 $_{subject}$			0.13 $_{subject}$		
	0.00 $_{trialNum\_fixed}$			0.00 $_{trialNum\_fixed}$			0.00 $_{trialNum\_fixed}$			0.00 $_{trialNum\_fixed}$		
ICC	0.31			0.31			0.31			0.31		
N	1451 $_{subject}$			1451 $_{subject}$			1451 $_{subject}$			1451 $_{subject}$		
	25 $_{trialNum\_fixed}$			25 $_{trialNum\_fixed}$			25 $_{trialNum\_fixed}$			25 $_{trialNum\_fixed}$		
Observations	34294			34294			34294			34294		
Marginal R^2 / Conditional R^2	0.013 / 0.317			0.012 / 0.317			0.012 / 0.317			0.012 / 0.317		

	M3.3_Hail_180d			M3.3_Strong Wind_180d			M3.3_Tornado_180d			M3.3_Wildfire_180d		
Predictors	*Estimates*	*CI*	*p*	*Estimates*	*CI*	*p*	*Estimates*	*CI*	*p*	*Estimates*	*CI*	*p*
(Intercept)	-0.17	-0.29 – -0.05	**0.005**	-0.13	-0.22 – -0.03	**0.014**	-0.13	-0.23 – -0.02	**0.019**	-0.11	-0.21 – -0.02	**0.019**
polAff bin str [Dem]	-0.19	-0.46 – 0.09	0.186	-0.18	-0.41 – 0.05	0.127	-0.15	-0.40 – 0.09	0.224	-0.22	-0.45 – 0.00	0.054
nEvents	0.04	-0.01 – 0.09	0.129	0.07	-0.07 – 0.20	0.351	0.06	-0.10 – 0.21	0.474	0.02	-0.09 – 0.13	0.707
EWE subjAttr mean	0.05	0.02 – 0.08	**0.001**	0.04	0.02 – 0.07	**0.001**	0.04	0.01 – 0.06	**0.004**	0.04	0.02 – 0.06	**0.001**
polAff bin str [Dem] × nEvents	-0.05	-0.18 – 0.09	0.504	-0.50	-0.92 – -0.08	**0.019**	-0.44	-0.90 – 0.02	0.059	-0.33	-0.70 – 0.05	0.088
polAff bin str [Dem] × EWE subjAttr mean	0.06	-0.00 – 0.12	0.068	0.05	0.00 – 0.10	**0.043**	0.05	-0.01 – 0.10	0.083	0.06	0.01 – 0.11	**0.017**
nEvents × EWE subjAttr mean	-0.01	-0.02 – 0.01	0.326	-0.02	-0.06 – 0.01	0.229	-0.00	-0.04 – 0.03	0.807	-0.01	-0.04 – 0.02	0.591
(polAff bin str [Dem] × nEvents) × EWE subjAttr mean	0.01	-0.02 – 0.04	0.603	0.11	0.02 – 0.20	**0.022**	0.09	-0.01 – 0.19	0.078	0.07	-0.01 – 0.16	0.093
**Random Effects**												
σ^2	0.29			0.29			0.29			0.29		
τ_{00}	0.13 subject			0.13 subject			0.13 subject			0.13 subject		
	0.00 trialNum_fixed			0.00 trialNum_fixed			0.00 trialNum_fixed			0.00 trialNum_fixed		
ICC	0.31			0.31			0.31			0.31		
N	1451 subject			1451 subject			1451 subject			1451 subject		
	25 trialNum_fixed			25 trialNum_fixed			25 trialNum_fixed			25 trialNum_fixed		
Observations	34294			34294			34294			34294		
Marginal R^2 / Conditional R^2	0.013 / 0.317			0.013 / 0.317			0.013 / 0.317			0.012 / 0.317		

	M3.3_Coastal Flood_210d			M3.3_Drought_210d			M3.3_Excessive Heat_210d			M3.3_Flash Flood_210d		
Predictors	*Estimates*	*CI*	*p*	*Estimates*	*CI*	*p*	*Estimates*	*CI*	*p*	*Estimates*	*CI*	*p*
(Intercept)	-0.11	-0.21 – -0.02	**0.016**	-0.12	-0.22 – -0.02	**0.019**	-0.13	-0.23 – -0.03	**0.011**	-0.17	-0.29 – -0.05	**0.005**
polAff bin str [Dem]	-0.25	-0.47 – -0.03	**0.028**	-0.24	-0.46 – -0.01	**0.040**	-0.21	-0.45 – 0.02	0.080	-0.23	-0.51 – 0.05	0.106
nEvents	2.43	0.23 – 4.63	**0.030**	0.01	-0.03 – 0.06	0.617	0.03	-0.02 – 0.08	0.267	0.04	-0.01 – 0.08	0.104
EWE subjAttr mean	0.04	0.02 – 0.06	**0.001**	0.04	0.02 – 0.06	**0.001**	0.04	0.02 – 0.07	**0.001**	0.05	0.03 – 0.08	**<0.001**
polAff bin str [Dem] × nEvents	-3.08	-6.07 – -0.08	**0.044**	-0.17	-0.43 – 0.09	0.205	-0.06	-0.21 – 0.08	0.397	-0.01	-0.11 – 0.09	0.826
polAff bin str [Dem] × EWE subjAttr mean	0.07	0.02 – 0.12	**0.008**	0.07	0.02 – 0.12	**0.010**	0.06	0.01 – 0.11	**0.023**	0.06	-0.00 – 0.13	0.051
nEvents × EWE subjAttr mean	-0.57	-1.08 – -0.06	**0.028**	-0.00	-0.01 – 0.01	0.704	-0.01	-0.02 – 0.01	0.345	-0.01	-0.02 – 0.00	0.085
(polAff bin str [Dem] × nEvents) × EWE subjAttr mean	0.74	0.06 – 1.43	**0.034**	0.03	-0.02 – 0.09	0.241	0.01	-0.02 – 0.04	0.511	0.00	-0.02 – 0.03	0.789
**Random Effects**												
σ^2	0.29			0.29			0.29			0.29		
τ_{00}	0.13 subject			0.13 subject			0.13 subject			0.13 subject		
	0.00 trialNum_fixed			0.00 trialNum_fixed			0.00 trialNum_fixed			0.00 trialNum_fixed		
ICC	0.31			0.31			0.31			0.31		
N	1451 subject			1451 subject			1451 subject			1451 subject		
	25 trialNum_fixed			25 trialNum_fixed			25 trialNum_fixed			25 trialNum_fixed		
Observations	34294			34294			34294			34294		
Marginal R^2 / Conditional R^2	0.013 / 0.317			0.012 / 0.317			0.012 / 0.317			0.012 / 0.317		

	M3.3_Hail_210d			M3.3_Strong Wind_210d			M3.3_Tornado_210d			M3.3_Wildfire_210d		
Predictors	*Estimates*	*CI*	*p*	*Estimates*	*CI*	*p*	*Estimates*	*CI*	*p*	*Estimates*	*CI*	*p*
(Intercept)	-0.17	-0.29 – -0.05	**0.004**	-0.13	-0.23 – -0.03	**0.011**	-0.13	-0.24 – -0.03	**0.013**	-0.12	-0.22 – -0.03	**0.013**
polAff bin str [Dem]	-0.18	-0.46 – 0.10	0.199	-0.12	-0.35 – 0.11	0.303	-0.15	-0.39 – 0.10	0.244	-0.21	-0.44 – 0.01	0.064
nEvents	0.04	-0.01 – 0.09	0.106	0.07	-0.07 – 0.20	0.317	0.08	-0.08 – 0.23	0.320	0.05	-0.06 – 0.15	0.366
EWE subjAttr mean	0.05	0.02 – 0.08	**0.001**	0.04	0.02 – 0.07	**<0.001**	0.04	0.01 – 0.07	**0.003**	0.04	0.02 – 0.07	**0.001**
polAff bin str [Dem] × nEvents	-0.05	-0.18 – 0.08	0.474	-0.70	-1.09 – -0.32	**<0.001**	-0.45	-0.91 – 0.00	0.051	-0.31	-0.67 – 0.04	0.080
polAff bin str [Dem] × EWE subjAttr mean	0.06	-0.01 – 0.12	0.074	0.04	-0.01 – 0.09	0.139	0.05	-0.01 – 0.10	0.091	0.06	0.01 – 0.11	**0.021**
nEvents × EWE subjAttr mean	-0.01	-0.02 – 0.01	0.279	-0.03	-0.06 – 0.01	0.141	-0.01	-0.05 – 0.03	0.636	-0.01	-0.04 – 0.01	0.307
(polAff bin str [Dem] × nEvents) × EWE subjAttr mean	0.01	-0.02 – 0.04	0.567	0.16	0.07 – 0.24	**<0.001**	0.09	-0.01 – 0.19	0.069	0.07	-0.01 – 0.15	0.088
**Random Effects**												
σ^2	0.29			0.29			0.29			0.29		
τ_{00}	0.13 $_{subject}$			0.13 $_{subject}$			0.13 $_{subject}$			0.13 $_{subject}$		
	0.00 $_{trialNum\_fixed}$			0.00 $_{trialNum\_fixed}$			0.00 $_{trialNum\_fixed}$			0.00 $_{trialNum\_fixed}$		
ICC	0.31			0.31			0.31			0.31		
N	1451 $_{subject}$			1451 $_{subject}$			1451 $_{subject}$			1451 $_{subject}$		
	25 $_{trialNum\_fixed}$			25 $_{trialNum\_fixed}$			25 $_{trialNum\_fixed}$			25 $_{trialNum\_fixed}$		
Observations	34294			34294			34294			34294		
Marginal R^2 / Conditional R^2	0.013 / 0.317			0.015 / 0.317			0.013 / 0.317			0.012 / 0.317		

	M3.3_Coastal Flood_240d			M3.3_Drought_240d			M3.3_Excessive Heat_240d			M3.3_Flash Flood_240d		
Predictors	*Estimates*	*CI*	*p*	*Estimates*	*CI*	*p*	*Estimates*	*CI*	*p*	*Estimates*	*CI*	*p*
(Intercept)	-0.11	-0.21 – -0.02	**0.016**	-0.11	-0.21 – -0.01	**0.031**	-0.13	-0.23 – -0.03	**0.011**	-0.17	-0.29 – -0.05	**0.005**
polAff bin str [Dem]	-0.25	-0.47 – -0.03	**0.028**	-0.25	-0.47 – -0.02	**0.033**	-0.21	-0.45 – 0.03	0.081	-0.23	-0.51 – 0.05	0.104
nEvents	2.43	0.23 – 4.63	**0.030**	-0.00	-0.04 – 0.04	0.951	0.03	-0.02 – 0.08	0.256	0.04	-0.01 – 0.08	0.108
EWE subjAttr mean	0.04	0.02 – 0.06	**0.001**	0.04	0.01 – 0.06	**0.002**	0.04	0.02 – 0.07	**0.001**	0.05	0.02 – 0.08	**<0.001**
polAff bin str [Dem] × nEvents	-3.08	-6.07 – -0.08	**0.044**	-0.13	-0.38 – 0.11	0.292	-0.06	-0.21 – 0.08	0.392	-0.01	-0.11 – 0.09	0.839
polAff bin str [Dem] × EWE subjAttr mean	0.07	0.02 – 0.12	**0.008**	0.07	0.02 – 0.12	**0.008**	0.06	0.01 – 0.11	**0.024**	0.06	0.00 – 0.13	**0.049**
nEvents × EWE subjAttr mean	-0.57	-1.08 – -0.06	**0.028**	0.00	-0.01 – 0.01	0.919	-0.01	-0.02 – 0.01	0.333	-0.01	-0.02 – 0.00	0.094
(polAff bin str [Dem] × nEvents) × EWE subjAttr mean	0.74	0.06 – 1.43	**0.034**	0.03	-0.03 – 0.08	0.339	0.01	-0.02 – 0.04	0.505	0.00	-0.02 – 0.03	0.812
**Random Effects**												
σ^2	0.29			0.29			0.29			0.29		
τ_{00}	0.13 $_{subject}$			0.13 $_{subject}$			0.13 $_{subject}$			0.13 $_{subject}$		
	0.00 $_{trialNum\_fixed}$			0.00 $_{trialNum\_fixed}$			0.00 $_{trialNum\_fixed}$			0.00 $_{trialNum\_fixed}$		
ICC	0.31			0.31			0.31			0.31		
N	1451 $_{subject}$			1451 $_{subject}$			1451 $_{subject}$			1451 $_{subject}$		
	25 $_{trialNum\_fixed}$			25 $_{trialNum\_fixed}$			25 $_{trialNum\_fixed}$			25 $_{trialNum\_fixed}$		
Observations	34294			34294			34294			34294		
Marginal R^2 / Conditional R^2	0.013 / 0.317			0.012 / 0.317			0.012 / 0.317			0.012 / 0.317		

	M3.3_Hail_240d			M3.3_Strong Wind_240d			M3.3_Tornado_240d			M3.3_Wildfire_240d		
Predictors	*Estimates*	*CI*	*p*	*Estimates*	*CI*	*p*	*Estimates*	*CI*	*p*	*Estimates*	*CI*	*p*
(Intercept)	-0.17	-0.29 – -0.05	**0.004**	-0.13	-0.23 – -0.03	**0.011**	-0.12	-0.23 – -0.02	**0.023**	-0.12	-0.22 – -0.03	**0.013**
polAff bin str [Dem]	-0.20	-0.47 – 0.08	0.166	-0.14	-0.37 – 0.09	0.242	-0.17	-0.42 – 0.08	0.174	-0.21	-0.44 – 0.02	0.068
nEvents	0.04	-0.01 – 0.09	0.102	0.07	-0.06 – 0.21	0.282	0.05	-0.10 – 0.20	0.542	0.05	-0.06 – 0.15	0.381
EWE subjAttr mean	0.05	0.02 – 0.08	**0.001**	0.04	0.02 – 0.07	**<0.001**	0.04	0.01 – 0.06	**0.006**	0.04	0.02 – 0.07	**0.001**
polAff bin str [Dem] × nEvents	-0.04	-0.17 – 0.09	0.569	-0.56	-0.90 – -0.22	**0.001**	-0.32	-0.76 – 0.12	0.157	-0.33	-0.68 – 0.02	0.067
polAff bin str [Dem] × EWE subjAttr mean	0.06	-0.00 – 0.12	0.060	0.04	-0.01 – 0.09	0.107	0.05	-0.00 – 0.11	0.058	0.06	0.01 – 0.11	**0.024**
nEvents × EWE subjAttr mean	-0.01	-0.02 – 0.00	0.260	-0.02	-0.06 – 0.01	0.134	-0.00	-0.04 – 0.04	0.964	-0.01	-0.04 – 0.01	0.290
(polAff bin str [Dem] × nEvents) × EWE subjAttr mean	0.01	-0.02 – 0.03	0.663	0.13	0.05 – 0.20	**0.001**	0.06	-0.03 – 0.16	0.206	0.07	-0.01 – 0.15	0.069
**Random Effects**												
σ^2	0.29			0.29			0.29			0.29		
τ_{00}	0.13 $_{subject}$			0.13 $_{subject}$			0.13 $_{subject}$			0.13 $_{subject}$		
	0.00 $_{trialNum\_fixed}$			0.00 $_{trialNum\_fixed}$			0.00 $_{trialNum\_fixed}$			0.00 $_{trialNum\_fixed}$		
ICC	0.31			0.31			0.31			0.31		
N	1451 $_{subject}$			1451 $_{subject}$			1451 $_{subject}$			1451 $_{subject}$		
	25 $_{trialNum\_fixed}$			25 $_{trialNum\_fixed}$			25 $_{trialNum\_fixed}$			25 $_{trialNum\_fixed}$		
Observations	34294			34294			34294			34294		
Marginal R^2 / Conditional R^2	0.013 / 0.317			0.015 / 0.317			0.013 / 0.317			0.013 / 0.317		

	M3.3_Coastal Flood_270d			M3.3_Drought_270d			M3.3_Excessive Heat_270d			M3.3_Flash Flood_270d		
Predictors	*Estimates*	*CI*	*p*	*Estimates*	*CI*	*p*	*Estimates*	*CI*	*p*	*Estimates*	*CI*	*p*
(Intercept)	-0.11	-0.21 – -0.02	**0.016**	-0.11	-0.21 – -0.01	**0.034**	-0.13	-0.23 – -0.03	**0.011**	-0.16	-0.28 – -0.05	**0.007**
polAff bin str [Dem]	-0.25	-0.47 – -0.03	**0.028**	-0.24	-0.47 – -0.02	**0.035**	-0.21	-0.45 – 0.03	0.081	-0.25	-0.53 – 0.03	0.080
nEvents	2.43	0.23 – 4.63	**0.030**	-0.00	-0.04 – 0.04	0.877	0.03	-0.02 – 0.08	0.256	0.03	-0.01 – 0.07	0.153
EWE subjAttr mean	0.04	0.02 – 0.06	**0.001**	0.04	0.01 – 0.06	**0.003**	0.04	0.02 – 0.07	**0.001**	0.05	0.02 – 0.08	**0.001**
polAff bin str [Dem] × nEvents	-3.08	-6.07 – -0.08	**0.044**	-0.10	-0.32 – 0.12	0.351	-0.06	-0.21 – 0.08	0.392	-0.00	-0.10 – 0.10	0.989
polAff bin str [Dem] × EWE subjAttr mean	0.07	0.02 – 0.12	**0.008**	0.07	0.02 – 0.12	**0.009**	0.06	0.01 – 0.11	**0.024**	0.07	0.00 – 0.13	**0.036**
nEvents × EWE subjAttr mean	-0.57	-1.08 – -0.06	**0.028**	0.00	-0.01 – 0.01	0.861	-0.01	-0.02 – 0.01	0.333	-0.01	-0.02 – 0.00	0.135
(polAff bin str [Dem] × nEvents) × EWE subjAttr mean	0.74	0.06 – 1.43	**0.034**	0.02	-0.03 – 0.07	0.396	0.01	-0.02 – 0.04	0.505	0.00	-0.02 – 0.02	0.963
**Random Effects**												
σ^2	0.29			0.29			0.29			0.29		
τ_{00}	0.13 $_{subject}$			0.13 $_{subject}$			0.13 $_{subject}$			0.13 $_{subject}$		
	0.00 $_{trialNum\_fixed}$			0.00 $_{trialNum\_fixed}$			0.00 $_{trialNum\_fixed}$			0.00 $_{trialNum\_fixed}$		
ICC	0.31			0.31			0.31			0.31		
N	1451 $_{subject}$			1451 $_{subject}$			1451 $_{subject}$			1451 $_{subject}$		
	25 $_{trialNum\_fixed}$			25 $_{trialNum\_fixed}$			25 $_{trialNum\_fixed}$			25 $_{trialNum\_fixed}$		
Observations	34294			34294			34294			34294		
Marginal R^2 / Conditional R^2	0.013 / 0.317			0.012 / 0.317			0.012 / 0.317			0.012 / 0.317		

	M3.3_Hail_270d			M3.3_Strong Wind_270d			M3.3_Tornado_270d			M3.3_Wildfire_270d		
Predictors	*Estimates*	*CI*	*p*	*Estimates*	*CI*	*p*	*Estimates*	*CI*	*p*	*Estimates*	*CI*	*p*
(Intercept)	-0.17	-0.29 – -0.05	**0.005**	-0.12	-0.22 – -0.02	**0.017**	-0.11	-0.21 – 0.00	0.054	-0.12	-0.22 – -0.03	**0.013**
polAff bin str [Dem]	-0.19	-0.47 – 0.09	0.178	-0.15	-0.38 – 0.08	0.212	-0.18	-0.43 – 0.07	0.149	-0.21	-0.44 – 0.02	0.068
nEvents	0.04	-0.01 – 0.08	0.108	0.04	-0.08 – 0.16	0.530	-0.01	-0.15 – 0.14	0.941	0.05	-0.06 – 0.15	0.381
EWE subjAttr mean	0.05	0.02 – 0.08	**0.001**	0.04	0.02 – 0.07	**<0.001**	0.03	0.01 – 0.06	**0.015**	0.04	0.02 – 0.07	**0.001**
polAff bin str [Dem] × nEvents	-0.04	-0.17 – 0.09	0.524	-0.46	-0.77 – -0.16	**0.003**	-0.29	-0.73 – 0.15	0.201	-0.33	-0.68 – 0.02	0.067
polAff bin str [Dem] × EWE subjAttr mean	0.06	-0.00 – 0.12	0.066	0.04	-0.01 – 0.10	0.099	0.06	0.00 – 0.11	**0.049**	0.06	0.01 – 0.11	**0.024**
nEvents × EWE subjAttr mean	-0.01	-0.02 – 0.00	0.277	-0.02	-0.05 – 0.01	0.228	0.01	-0.03 – 0.05	0.565	-0.01	-0.04 – 0.01	0.290
(polAff bin str [Dem] × nEvents) × EWE subjAttr mean	0.01	-0.02 – 0.04	0.611	0.11	0.04 – 0.17	**0.002**	0.06	-0.04 – 0.15	0.257	0.07	-0.01 – 0.15	0.069
**Random Effects**												
σ^2	0.29			0.29			0.29			0.29		
τ_{00}	0.13 subject			0.13 subject			0.13 subject			0.13 subject		
	0.00 trialNum_fixed			0.00 trialNum_fixed			0.00 trialNum_fixed			0.00 trialNum_fixed		
ICC	0.31			0.31			0.31			0.31		
N	1451 subject			1451 subject			1451 subject			1451 subject		
	25 trialNum_fixed			25 trialNum_fixed			25 trialNum_fixed			25 trialNum_fixed		
Observations	34294			34294			34294			34294		
Marginal R^2 / Conditional R^2	0.013 / 0.317			0.015 / 0.317			0.013 / 0.317			0.013 / 0.317		

	M3.3_Coastal Flood_300d			M3.3_Drought_300d			M3.3_Excessive Heat_300d			M3.3_Flash Flood_300d		
Predictors	*Estimates*	*CI*	*p*	*Estimates*	*CI*	*p*	*Estimates*	*CI*	*p*	*Estimates*	*CI*	*p*
(Intercept)	-0.12	-0.22 – -0.03	**0.010**	-0.10	-0.20 – 0.01	0.063	-0.13	-0.23 – -0.03	**0.011**	-0.16	-0.28 – -0.04	**0.008**
polAff bin str [Dem]	-0.21	-0.44 – 0.01	0.061	-0.25	-0.49 – -0.02	**0.031**	-0.21	-0.45 – 0.03	0.081	-0.25	-0.53 – 0.03	0.077
nEvents	0.44	0.01 – 0.88	**0.046**	-0.01	-0.05 – 0.02	0.466	0.03	-0.02 – 0.08	0.256	0.03	-0.01 – 0.07	0.180
EWE subjAttr mean	0.04	0.02 – 0.06	**<0.001**	0.03	0.01 – 0.06	**0.006**	0.04	0.02 – 0.07	**0.001**	0.05	0.02 – 0.08	**0.001**
polAff bin str [Dem] × nEvents	-1.93	-3.28 – -0.58	**0.005**	-0.04	-0.23 – 0.15	0.667	-0.06	-0.21 – 0.08	0.392	0.00	-0.10 – 0.10	0.982
polAff bin str [Dem] × EWE subjAttr mean	0.06	0.01 – 0.11	**0.020**	0.07	0.02 – 0.12	**0.008**	0.06	0.01 – 0.11	**0.024**	0.07	0.01 – 0.13	**0.034**
nEvents × EWE subjAttr mean	-0.10	-0.23 – 0.02	0.104	0.00	-0.01 – 0.01	0.481	-0.01	-0.02 – 0.01	0.333	-0.01	-0.02 – 0.00	0.166
(polAff bin str [Dem] × nEvents) × EWE subjAttr mean	0.46	0.14 – 0.77	**0.004**	0.01	-0.03 – 0.05	0.721	0.01	-0.02 – 0.04	0.505	0.00	-0.02 – 0.02	0.994
**Random Effects**												
σ^2	0.29			0.29			0.29			0.29		
τ_{00}	0.13 subject			0.13 subject			0.13 subject			0.13 subject		
	0.00 trialNum_fixed			0.00 trialNum_fixed			0.00 trialNum_fixed			0.00 trialNum_fixed		
ICC	0.31			0.31			0.31			0.31		
N	1451 subject			1451 subject			1451 subject			1451 subject		
	25 trialNum_fixed			25 trialNum_fixed			25 trialNum_fixed			25 trialNum_fixed		
Observations	34294			34294			34294			34294		
Marginal R^2 / Conditional R^2	0.014 / 0.317			0.012 / 0.317			0.012 / 0.317			0.012 / 0.317		

	M3.3_Hail_300d			M3.3_Strong Wind_300d			M3.3_Tornado_300d			M3.3_Wildfire_300d		
Predictors	*Estimates*	*CI*	*p*	*Estimates*	*CI*	*p*	*Estimates*	*CI*	*p*	*Estimates*	*CI*	*p*
(Intercept)	-0.17	-0.29 – -0.05	**0.005**	-0.12	-0.22 – -0.02	**0.023**	-0.11	-0.22 – -0.00	**0.044**	-0.12	-0.22 – -0.03	**0.013**
polAff bin str [Dem]	-0.20	-0.48 – 0.08	0.158	-0.17	-0.40 – 0.07	0.162	-0.18	-0.43 – 0.07	0.155	-0.21	-0.43 – 0.02	0.072
nEvents	0.04	-0.01 – 0.08	0.114	0.02	-0.09 – 0.13	0.743	0.01	-0.13 – 0.15	0.899	0.04	-0.06 – 0.14	0.446
EWE subjAttr mean	0.05	0.02 – 0.08	**0.001**	0.04	0.02 – 0.07	**0.001**	0.03	0.01 – 0.06	**0.013**	0.04	0.02 – 0.07	**<0.001**
polAff bin str [Dem] × nEvents	-0.03	-0.16 – 0.09	0.598	-0.36	-0.64 – -0.09	**0.010**	-0.27	-0.70 – 0.16	0.211	-0.33	-0.66 – 0.01	0.056
polAff bin str [Dem] × EWE subjAttr mean	0.06	-0.00 – 0.12	0.057	0.05	-0.00 – 0.10	0.070	0.06	0.00 – 0.11	**0.049**	0.06	0.01 – 0.11	**0.026**
nEvents × EWE subjAttr mean	-0.01	-0.02 – 0.01	0.296	-0.01	-0.04 – 0.02	0.426	0.01	-0.03 – 0.04	0.638	-0.01	-0.04 – 0.01	0.308
(polAff bin str [Dem] × nEvents) × EWE subjAttr mean	0.01	-0.02 – 0.03	0.691	0.08	0.02 – 0.14	**0.007**	0.05	-0.04 – 0.14	0.277	0.07	-0.00 – 0.15	0.055
**Random Effects**												
σ^2	0.29			0.29			0.29			0.29		
τ_{00}	0.13 $_{subject}$			0.13 $_{subject}$			0.13 $_{subject}$			0.13 $_{subject}$		
	0.00 $_{trialNum\_fixed}$			0.00 $_{trialNum\_fixed}$			0.00 $_{trialNum\_fixed}$			0.00 $_{trialNum\_fixed}$		
ICC	0.31			0.31			0.31			0.31		
N	1451 $_{subject}$			1451 $_{subject}$			1451 $_{subject}$			1451 $_{subject}$		
	25 $_{trialNum\_fixed}$			25 $_{trialNum\_fixed}$			25 $_{trialNum\_fixed}$			25 $_{trialNum\_fixed}$		
Observations	34294			34294			34294			34294		
Marginal R^2 / Conditional R^2	0.013 / 0.317			0.014 / 0.317			0.013 / 0.317			0.013 / 0.317		

	M3.3_Coastal Flood_330d			M3.3_Drought_330d			M3.3_Excessive Heat_330d			M3.3_Flash Flood_330d		
Predictors	*Estimates*	*CI*	*p*	*Estimates*	*CI*	*p*	*Estimates*	*CI*	*p*	*Estimates*	*CI*	*p*
(Intercept)	-0.12	-0.22 – -0.03	**0.012**	-0.10	-0.20 – 0.01	0.066	-0.13	-0.23 – -0.03	**0.009**	-0.17	-0.29 – -0.05	**0.006**
polAff bin str [Dem]	-0.21	-0.44 – 0.01	0.062	-0.25	-0.48 – -0.02	**0.033**	-0.21	-0.44 – 0.03	0.081	-0.24	-0.53 – 0.04	0.090
nEvents	0.19	-0.10 – 0.48	0.202	-0.01	-0.05 – 0.02	0.466	0.03	-0.02 – 0.07	0.201	0.03	-0.01 – 0.07	0.136
EWE subjAttr mean	0.04	0.02 – 0.06	**0.001**	0.03	0.01 – 0.06	**0.006**	0.04	0.02 – 0.07	**0.001**	0.05	0.02 – 0.08	**0.001**
polAff bin str [Dem] × nEvents	-1.13	-2.09 – -0.17	**0.021**	-0.05	-0.23 – 0.14	0.609	-0.06	-0.19 – 0.07	0.352	-0.00	-0.10 – 0.10	0.958
polAff bin str [Dem] × EWE subjAttr mean	0.06	0.01 – 0.11	**0.017**	0.07	0.02 – 0.12	**0.009**	0.06	0.01 – 0.11	**0.024**	0.07	0.00 – 0.13	**0.041**
nEvents × EWE subjAttr mean	-0.03	-0.10 – 0.05	0.474	0.00	-0.01 – 0.01	0.485	-0.01	-0.02 – 0.00	0.262	-0.01	-0.02 – 0.00	0.132
(polAff bin str [Dem] × nEvents) × EWE subjAttr mean	0.24	0.02 – 0.45	**0.030**	0.01	-0.03 – 0.05	0.661	0.01	-0.02 – 0.04	0.452	0.00	-0.02 – 0.02	0.934
**Random Effects**												
σ^2	0.29			0.29			0.29			0.29		
τ_{00}	0.13 $_{subject}$			0.13 $_{subject}$			0.13 $_{subject}$			0.13 $_{subject}$		
	0.00 $_{trialNum\_fixed}$			0.00 $_{trialNum\_fixed}$			0.00 $_{trialNum\_fixed}$			0.00 $_{trialNum\_fixed}$		
ICC	0.31			0.31			0.31			0.31		
N	1451 $_{subject}$			1451 $_{subject}$			1451 $_{subject}$			1451 $_{subject}$		
	25 $_{trialNum\_fixed}$			25 $_{trialNum\_fixed}$			25 $_{trialNum\_fixed}$			25 $_{trialNum\_fixed}$		
Observations	34294			34294			34294			34294		
Marginal R^2 / Conditional R^2	0.014 / 0.317			0.012 / 0.317			0.012 / 0.317			0.012 / 0.317		

	M3.3_Hail_330d			M3.3_Strong Wind_330d			M3.3_Tornado_330d			M3.3_Wildfire_330d		
Predictors	*Estimates*	*CI*	*p*	*Estimates*	*CI*	*p*	*Estimates*	*CI*	*p*	*Estimates*	*CI*	*p*
(Intercept)	-0.16	-0.28 – -0.04	**0.007**	-0.12	-0.22 – -0.02	**0.022**	-0.09	-0.20 – 0.02	0.104	-0.12	-0.22 – -0.03	**0.013**
polAff bin str [Dem]	-0.21	-0.49 – 0.07	0.143	-0.17	-0.40 – 0.06	0.155	-0.18	-0.43 – 0.08	0.170	-0.21	-0.43 – 0.02	0.072
nEvents	0.03	-0.01 – 0.08	0.175	0.02	-0.08 – 0.12	0.731	-0.04	-0.17 – 0.10	0.596	0.04	-0.06 – 0.13	0.459
EWE subjAttr mean	0.05	0.02 – 0.07	**0.002**	0.04	0.02 – 0.07	**0.001**	0.03	-0.00 – 0.05	0.056	0.04	0.02 – 0.07	**<0.001**
polAff bin str [Dem] × nEvents	-0.03	-0.15 – 0.10	0.666	-0.35	-0.61 – -0.08	**0.012**	-0.31	-0.73 – 0.10	0.142	-0.31	-0.63 – 0.01	0.056
polAff bin str [Dem] × EWE subjAttr mean	0.06	-0.00 – 0.12	0.050	0.05	-0.00 – 0.10	0.066	0.06	0.00 – 0.11	**0.045**	0.06	0.01 – 0.11	**0.026**
nEvents × EWE subjAttr mean	-0.00	-0.02 – 0.01	0.407	-0.01	-0.04 – 0.01	0.412	0.02	-0.01 – 0.06	0.192	-0.01	-0.04 – 0.01	0.290
(polAff bin str [Dem] × nEvents) × EWE subjAttr mean	0.00	-0.02 – 0.03	0.766	0.08	0.02 – 0.14	**0.008**	0.06	-0.03 – 0.15	0.227	0.07	-0.00 – 0.14	0.053
**Random Effects**												
σ^2	0.29			0.29			0.29			0.29		
τ_{00}	0.13 $_{subject}$			0.13 $_{subject}$			0.13 $_{subject}$			0.13 $_{subject}$		
	0.00 $_{trialNum\_fixed}$			0.00 $_{trialNum\_fixed}$			0.00 $_{trialNum\_fixed}$			0.00 $_{trialNum\_fixed}$		
ICC	0.31			0.31			0.31			0.31		
N	1451 $_{subject}$			1451 $_{subject}$			1451 $_{subject}$			1451 $_{subject}$		
	25 $_{trialNum\_fixed}$			25 $_{trialNum\_fixed}$			25 $_{trialNum\_fixed}$			25 $_{trialNum\_fixed}$		
Observations	34294			34294			34294			34294		
Marginal R^2 / Conditional R^2	0.013 / 0.317			0.014 / 0.317			0.014 / 0.317			0.013 / 0.317		

	M3.3_Coastal Flood_360d			M3.3_Drought_360d			M3.3_Excessive Heat_360d			M3.3_Flash Flood_360d		
Predictors	*Estimates*	*CI*	*p*	*Estimates*	*CI*	*p*	*Estimates*	*CI*	*p*	*Estimates*	*CI*	*p*
(Intercept)	-0.12	-0.21 – -0.02	**0.016**	-0.09	-0.20 – 0.01	0.074	-0.13	-0.23 – -0.03	**0.009**	-0.16	-0.28 – -0.03	**0.012**
polAff bin str [Dem]	-0.23	-0.45 – -0.01	**0.044**	-0.26	-0.49 – -0.02	**0.032**	-0.21	-0.44 – 0.03	0.082	-0.26	-0.54 – 0.03	0.077
nEvents	0.12	-0.14 – 0.38	0.353	-0.02	-0.05 – 0.02	0.399	0.03	-0.01 – 0.07	0.181	0.02	-0.02 – 0.06	0.246
EWE subjAttr mean	0.04	0.02 – 0.06	**0.001**	0.03	0.01 – 0.06	**0.008**	0.04	0.02 – 0.07	**<0.001**	0.05	0.02 – 0.08	**0.001**
polAff bin str [Dem] × nEvents	-0.56	-1.31 – 0.19	0.146	-0.04	-0.21 – 0.14	0.668	-0.06	-0.18 – 0.06	0.337	0.00	-0.09 – 0.10	0.933
polAff bin str [Dem] × EWE subjAttr mean	0.06	0.01 – 0.11	**0.012**	0.07	0.02 – 0.12	**0.008**	0.06	0.01 – 0.11	**0.024**	0.07	0.01 – 0.13	**0.034**
nEvents × EWE subjAttr mean	-0.02	-0.08 – 0.04	0.526	0.00	-0.01 – 0.01	0.399	-0.01	-0.02 – 0.00	0.242	-0.01	-0.02 – 0.00	0.248
(polAff bin str [Dem] × nEvents) × EWE subjAttr mean	0.12	-0.05 – 0.28	0.173	0.01	-0.03 – 0.04	0.726	0.01	-0.02 – 0.04	0.439	-0.00	-0.02 – 0.02	0.954
**Random Effects**												
σ^2	0.29			0.29			0.29			0.29		
τ_{00}	0.13 $_{subject}$			0.13 $_{subject}$			0.13 $_{subject}$			0.13 $_{subject}$		
	0.00 $_{trialNum\_fixed}$			0.00 $_{trialNum\_fixed}$			0.00 $_{trialNum\_fixed}$			0.00 $_{trialNum\_fixed}$		
ICC	0.31			0.31			0.31			0.31		
N	1451 $_{subject}$			1451 $_{subject}$			1451 $_{subject}$			1451 $_{subject}$		
	25 $_{trialNum\_fixed}$			25 $_{trialNum\_fixed}$			25 $_{trialNum\_fixed}$			25 $_{trialNum\_fixed}$		
Observations	34294			34294			34294			34294		
Marginal R^2 / Conditional R^2	0.012 / 0.317			0.012 / 0.317			0.012 / 0.317			0.012 / 0.317		

	M3.3_Hail_360d			M3.3_Strong Wind_360d			M3.3_Tornado_360d			M3.3_Wildfire_360d		
Predictors	*Estimates*	*CI*	*p*	*Estimates*	*CI*	*p*	*Estimates*	*CI*	*p*	*Estimates*	*CI*	*p*
(Intercept)	-0.16	-0.28 – -0.04	**0.008**	-0.12	-0.22 – -0.02	**0.023**	-0.10	-0.21 – 0.01	0.072	-0.12	-0.22 – -0.02	**0.014**
polAff bin str [Dem]	-0.22	-0.50 – 0.06	0.120	-0.17	-0.40 – 0.07	0.160	-0.16	-0.41 – 0.09	0.219	-0.21	-0.43 – 0.02	0.072
nEvents	0.03	-0.01 – 0.07	0.182	0.01	-0.08 – 0.11	0.767	-0.01	-0.15 – 0.12	0.849	0.03	-0.06 – 0.12	0.529
EWE subjAttr mean	0.05	0.02 – 0.07	**0.002**	0.04	0.02 – 0.07	**0.001**	0.03	0.00 – 0.06	**0.041**	0.04	0.02 – 0.07	**<0.001**
polAff bin str [Dem] × nEvents	-0.02	-0.14 – 0.10	0.777	-0.34	-0.60 – -0.08	**0.011**	-0.33	-0.74 – 0.08	0.115	-0.28	-0.56 – -0.01	**0.046**
polAff bin str [Dem] × EWE subjAttr mean	0.06	0.00 – 0.13	**0.042**	0.05	-0.00 – 0.10	0.069	0.05	-0.00 – 0.11	0.064	0.06	0.01 – 0.11	**0.026**
nEvents × EWE subjAttr mean	-0.00	-0.01 – 0.01	0.388	-0.01	-0.03 – 0.01	0.454	0.02	-0.02 – 0.05	0.304	-0.01	-0.03 – 0.01	0.334
(polAff bin str [Dem] × nEvents) × EWE subjAttr mean	0.00	-0.02 – 0.03	0.868	0.08	0.02 – 0.13	**0.009**	0.06	-0.03 – 0.15	0.184	0.06	0.00 – 0.12	**0.042**
**Random Effects**												
σ^2	0.29			0.29			0.29			0.29		
τ_{00}	0.13 $_{subject}$			0.13 $_{subject}$			0.13 $_{subject}$			0.13 $_{subject}$		
	0.00 $_{trialNum\_fixed}$			0.00 $_{trialNum\_fixed}$			0.00 $_{trialNum\_fixed}$			0.00 $_{trialNum\_fixed}$		
ICC	0.31			0.31			0.31			0.31		
N	1451 $_{subject}$			1451 $_{subject}$			1451 $_{subject}$			1451 $_{subject}$		
	25 $_{trialNum\_fixed}$			25 $_{trialNum\_fixed}$			25 $_{trialNum\_fixed}$			25 $_{trialNum\_fixed}$		
Observations	34294			34294			34294			34294		
Marginal R^2 / Conditional R^2	0.013 / 0.317			0.014 / 0.317			0.014 / 0.317			0.013 / 0.317		

Table S16: ***Sensitivity analyses: Mixed-effects models predicting ΔDuration across different EWE exposure time windows and event types (M3).*** *ΔDuration (deltaDuration) represents relative dwell time spent on carbon information compared to bonus information. Random intercepts were included for participants (subject) and trials (trialNum_fixed) (N = 1,451 participants). The models included political affiliation (polAff_bin_str, subject-level binary variable with 0 = Republican and 1 = Democrat), the number of EWE a participant experienced (nEvents), subjective attribution and their interaction. Models are calculated for different time windows (60-360 days) and event types (coastal flood, drought, excessive heat, flash flood, hail, strong wind, tornado and wildfire). In some models, the three-way interaction could not be estimated due to insufficient variance in nEvents; in these cases, the interaction term was omitted. Estimates represent unstandardized beta coefficients. Confidence intervals are 95%. Statistically significant effects (p < .05) are shown in bold.*

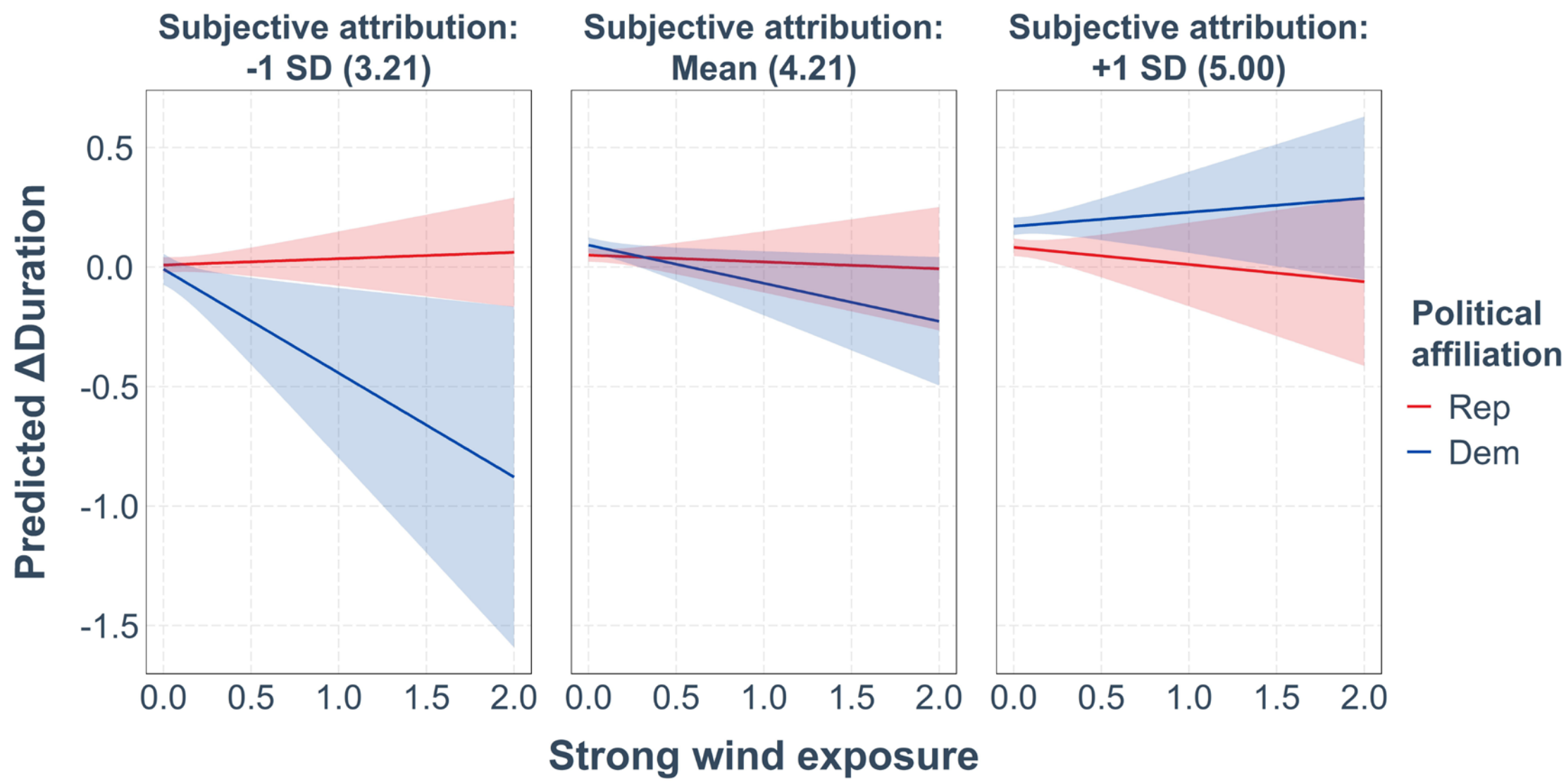

Figure S13: ***Three-way interaction between political affiliation, strong wind exposure in the last 120 days, and subjective attribution on ΔDuration.*** *Predicted values are based on the corresponding mixed-effects model (N = 1,451 participants). Panels display predicted ΔDuration as a function of strong wind exposure during the 120 days preceding study completion at low (−1 SD), mean, and high (+1 SD) levels of subjective attribution of EWE to climate change (bounded by the observed range), separately for Republicans and Democrats. Red and blue lines represent predicted values for Republicans and Democrats, respectively. Shaded areas represent 95% confidence intervals.*

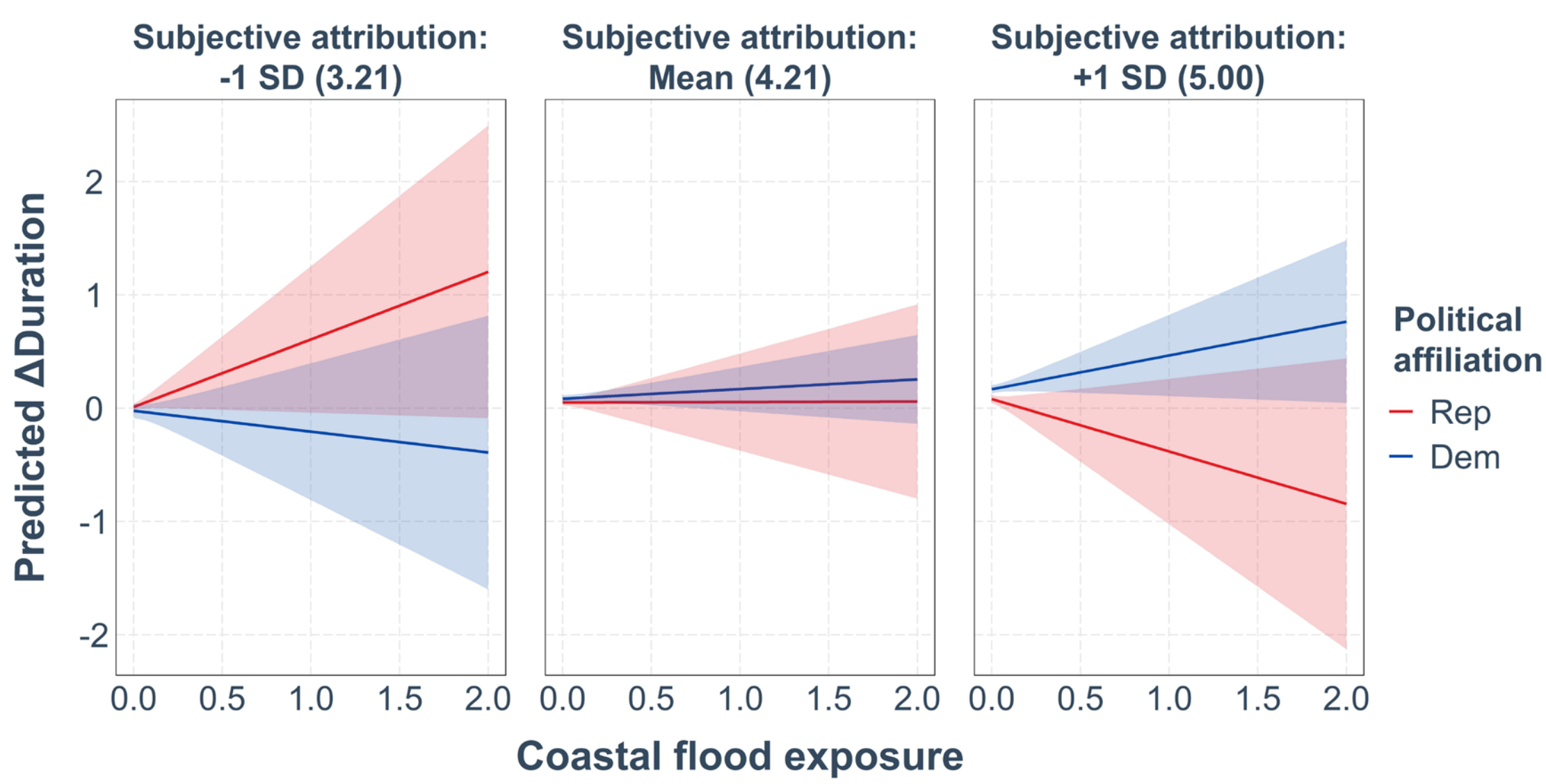

Figure S14: ***Three-way interaction between political affiliation, coastal flood in the last 120 days, and subjective attribution on ΔDuration.*** *Predicted values are based on the corresponding mixed-effects model (N = 1,451 participants). Panels display predicted ΔDuration as a function of coastal flood exposure during the 120 days preceding study completion at low (−1 SD), mean, and high (+1 SD) levels of subjective attribution of EWE to climate change (bounded by the observed range), separately for Republicans and Democrats. Red and blue lines represent predicted values for Republicans and Democrats, respectively. Shaded areas represent 95% confidence intervals.*

1.3.2. Subjective Attribution to Climate Change and Choice (H_{S3})

To test the hypothesis that political affiliation, EWE exposure, and subjective attribution of EWE to climate change interact in predicting pro climate choices, we estimated mixed-effects logistic regression models with choice as the dependent variable and random intercepts for participants and trials (see Table S17).

In Model 1, political affiliation and subjective attribution of EWE to climate change were positively linked to pro-environmental choices (*OR* = 1.68, p = .002; *OR* = 1.41, p < .001), whereas EWE exposure had a negative effect (*OR* = 0.72, p = .036). This association between exposure and choice was not observed in the models reported for Hypothesis 2 (i.e., without controlling for subjective attribution).

Model 2 included political affiliation, subjective attribution of EWE to climate change and their interaction. This interaction was positive and statistically significant (*OR* = 1.63, p = .016), indicating that the association between subjective attribution and pro-climate choices was stronger among Democrats than among Republicans.

Model 3 included EWE exposure and subjective attribution of EWE to climate change and their interaction. This interaction was negative and statistically significant (*OR* = 0.65, p = .010), indicating that the association between EWE exposure and pro-climate choices weakened as subjective attribution increased.

Model 4 included political affiliation, EWE exposure, and subjective attribution of EWE to climate change, as well as all three two-way interactions. The interaction between political affiliation and EWE exposure was not statistically significant (*OR* = 1.21, p = .564).

Model 5 additionally included the full three-way interaction between political affiliation, EWE exposure, and subjective attribution to climate change, which was also not statistically significant (*OR* = 0.70, p = .406). This pattern remained unchanged when additionally controlling for task and participant-level characteristics (Model 6, *OR* = 0.75, p = .485), county-level indicators of EWE severity and impacts (Model 7, *OR* = 0.69, p = .384), and when replacing the binary exposure indicator with a duration-weighted measure of EWE exposure (Model 8, *OR* = 0.98, p = .599).

	M3.1			M3.2		
Predictors	*Odds Ratios*	*CI*	*p*	*Odds Ratios*	*CI*	*p*
(Intercept)	0.28	0.14 – 0.58	**0.001**	0.38	0.17 – 0.83	**0.016**
polAff bin str [Dem]	1.68	1.21 – 2.33	**0.002**	0.20	0.03 – 1.21	0.079
nEpisodesIsGreaterThanZeroTRUE	0.72	0.52 – 0.98	**0.036**			
EWE subjAttr mean	1.41	1.19 – 1.66	**<0.001**	1.26	1.04 – 1.51	**0.016**
polAff bin str [Dem] × EWE subjAttr mean				1.64	1.09 – 2.46	**0.017**
**Random Effects**						
σ^2	3.29			3.29		
τ_{00}	8.38 $_{subject}$			8.38 $_{subject}$		
	0.24 $_{trialNum\_fixed}$			0.24 $_{trialNum\_fixed}$		
ICC	0.72			0.72		
N	1451 $_{subject}$			1451 $_{subject}$		
	25 $_{trialNum\_fixed}$			25 $_{trialNum\_fixed}$		
Observations	34428			34428		
Marginal R^2 / Conditional R^2	0.022 / 0.730			0.022 / 0.730		

	M3.3		
Predictors	*Odds Ratios*	*CI*	*p*
(Intercept)	0.13	0.05 – 0.32	**<0.001**
nEpisodesIsGreaterThanZeroTRUE	4.14	1.02 – 16.79	**0.046**
EWE subjAttr mean	1.82	1.47 – 2.24	**<0.001**
nEpisodesIsGreaterThanZeroTRUE × EWE subjAttr mean	0.65	0.47 – 0.90	**0.010**
**Random Effects**			
σ^2	3.29		
$\tau_{00\ subject}$	8.40		
$\tau_{00\ trialNum\_fixed}$	0.24		
ICC	0.72		
N $_{subject}$	1451		
N $_{trialNum\_fixed}$	25		
Observations	34428		
Marginal R^2 / Conditional R^2	0.020 / 0.730		

		M3.4	
Predictors	*Odds Ratios*	*CI*	*p*
(Intercept)	0.20	0.08 – 0.54	**0.001**
polAff bin str [Dem]	0.19	0.03 – 1.20	0.077
nEpisodesIsGreaterThanZeroTRUE	4.07	1.01 – 16.43	**0.048**
EWE subjAttr mean	1.54	1.21 – 1.95	**<0.001**
polAff bin str [Dem] × nEpisodesIsGreaterThanZeroTRUE	1.21	0.63 – 2.33	0.564
polAff bin str [Dem] × EWE subjAttr mean	1.60	1.07 – 2.40	**0.023**
nEpisodesIsGreaterThanZeroTRUE × EWE subjAttr mean	0.65	0.46 – 0.91	**0.011**
**Random Effects**			
σ^2		3.29	
$\tau_{00\ subject}$		8.31	
$\tau_{00\ trialNum\_fixed}$		0.24	
ICC		0.72	
$N_{subject}$		1451	
$N_{trialNum\_fixed}$		25	
Observations		34428	
Marginal R^2 / Conditional R^2		0.028 / 0.730	

	M3.5		
Predictors	*Odds Ratios*	*CI*	*p*
(Intercept)	0.23	0.08 – 0.64	**0.005**
polAff bin str [Dem]	0.10	0.01 – 1.07	0.057
nEpisodesIsGreaterThanZeroTRUE	3.06	0.65 – 14.39	0.158
EWE subjAttr mean	1.49	1.16 – 1.91	**0.002**
polAff bin str [Dem] × nEpisodesIsGreaterThanZeroTRUE	5.67	0.14 – 229.60	0.358
polAff bin str [Dem] × EWE subjAttr mean	1.85	1.09 – 3.12	**0.023**
nEpisodesIsGreaterThanZeroTRUE × EWE subjAttr mean	0.70	0.48 – 1.02	0.061
(polAff bin str [Dem] × nEpisodesIsGreaterThanZeroTRUE) × EWE subjAttr mean	0.70	0.31 – 1.61	0.406
**Random Effects**			
σ^2		3.29	
$\tau_{00\ subject}$		8.30	
$\tau_{00\ trialNum\_fixed}$		0.24	
ICC		0.72	
$N_{subject}$		1451	
$N_{trialNum\_fixed}$		25	
Observations		34428	
Marginal R^2 / Conditional R^2		0.029 / 0.730	

	M3.6		
Predictors	*Odds Ratios*	*CI*	*p*
(Intercept)	0.02	0.01 – 0.08	**<0.001**
polAff bin str [Dem]	0.42	0.04 – 4.33	0.470
nEpisodesIsGreaterThanZeroTRUE	3.22	0.70 – 14.80	0.133
EWE subjAttr mean	1.83	1.42 – 2.36	**<0.001**
carbon prcnt	1.01	1.01 – 1.01	**<0.001**
bonus prcnt	0.99	0.99 – 0.99	**<0.001**
age	1.03	1.02 – 1.04	**<0.001**
racethn [European]	1.20	0.87 – 1.65	0.278
racethn [Hispanic]	1.11	0.75 – 1.63	0.611
racethn [African]	0.27	0.13 – 0.55	**<0.001**
racethn [Asian]	1.86	0.89 – 3.89	0.098
racethn [moreThanOne]	1.05	0.48 – 2.28	0.901
polAff bin str [Dem] × nEpisodesIsGreaterThanZeroTRUE	3.93	0.10 – 152.18	0.463
polAff bin str [Dem] × EWE subjAttr mean	1.34	0.79 – 2.26	0.280
nEpisodesIsGreaterThanZeroTRUE × EWE subjAttr mean	0.71	0.49 – 1.02	0.067
(polAff bin str [Dem] × nEpisodesIsGreaterThanZeroTRUE) × EWE subjAttr mean	0.75	0.33 – 1.69	0.485
**Random Effects**			
σ^2		3.29	
$\tau_{00\ subject}$		7.97	
$\tau_{00\ trialNum\_fixed}$		0.02	
ICC		0.71	
$N_{subject}$		1451	
$N_{trialNum\_fixed}$		25	
Observations		34428	
Marginal R^2 / Conditional R^2		0.074 / 0.730	

	M3.7		
Predictors	*Odds Ratios*	*CI*	*p*
(Intercept)	0.18	0.06 – 0.50	**0.001**
polAff bin str [Dem]	0.13	0.01 – 1.36	0.089
nEpisodesIsGreaterThanZeroTRUE	2.75	0.56 – 13.55	0.213
EWE subjAttr mean	1.56	1.21 – 2.01	**0.001**
abnormality final	0.94	0.80 – 1.11	0.481
sum damage	1.01	0.86 – 1.19	0.891
sum injuries	1.09	0.93 – 1.28	0.267
sum deaths	1.00	0.85 – 1.17	0.951
recency index	1.51	0.19 – 12.08	0.695
population density peopPerSqKilometer	1.03	0.86 – 1.23	0.743
countyPvi	0.75	0.62 – 0.91	**0.003**
countyIncome	1.12	0.94 – 1.32	0.208
polAff bin str [Dem] × nEpisodesIsGreaterThanZeroTRUE	5.55	0.14 – 225.25	0.365
polAff bin str [Dem] × EWE subjAttr mean	1.76	1.04 – 2.99	**0.035**
nEpisodesIsGreaterThanZeroTRUE × EWE subjAttr mean	0.73	0.50 – 1.07	0.107
(polAff bin str [Dem] × nEpisodesIsGreaterThanZeroTRUE) × EWE subjAttr mean	0.69	0.30 – 1.58	0.384
**Random Effects**			
σ^2	3.29		
$\tau_{00\ subject}$	8.23		
$\tau_{00\ trialNum\_fixed}$	0.24		
ICC	0.72		
$N_{subject}$	1451		
$N_{trialNum\_fixed}$	25		
Observations	34428		
Marginal R^2 / Conditional R^2	0.035 / 0.730		

	M3.8		
Predictors	*Odds Ratios*	*CI*	*p*
(Intercept)	0.33	0.14 – 0.74	**0.007**
polAff bin str [Dem]	0.18	0.03 – 1.17	0.073
nEpisodes durationWeighted	1.04	0.99 – 1.09	0.151
EWE subjAttr mean	1.35	1.12 – 1.64	**0.002**
polAff bin str [Dem] × nEpisodes durationWeighted	1.13	0.80 – 1.60	0.492
polAff bin str [Dem] × EWE subjAttr mean	1.61	1.06 – 2.45	**0.025**
nEpisodes durationWeighted × EWE subjAttr mean	0.98	0.97 – 1.00	**0.010**
(polAff bin str [Dem] × nEpisodes durationWeighted) × EWE subjAttr mean	0.98	0.91 – 1.06	0.599
**Random Effects**			
σ^2		3.29	
$\tau_{00\ subject}$		8.25	
$\tau_{00\ trialNum\_fixed}$		0.24	
ICC		0.72	
$N_{subject}$		1451	
$N_{trialNum\_fixed}$		25	
Observations		34428	
Marginal R^2 / Conditional R^2		0.033 / 0.730	

Table S17: ***Mixed-effects logistic regression models predicting choice (M3.1–M3.8).*** *Choice represents the actual decision in the Carbon Emission Task (trial-level variable with 0 = pro-self and 1 = pro-climate). Random intercepts were included for participants (subject) and trials (trialNum_fixed) (N = 1,451 participants). In Model M3.1, political affiliation (polAff_bin_str), EWE exposure (nEpisodesIsGreaterThanZero), and subjective attribution of EWE to climate change (EWE_subAttr_mean) were included as predictors of choice. Model M3.2 included political affiliation, subjective attribution and their interaction. Model M3.3 included EWE exposure and subjective attribution and their interaction. Model M3.4 included political affiliation, EWE exposure and subjective attribution, as well as all three two-way interactions. Model M3.5 additionally included the three-way interaction between political affiliation, EWE exposure, and subjective attribution. Model M3.6 further controlled for attribute differences (carbon_prcnt and bonus_prcnt) age, and race/ethnicity (racethn). racethn was effect-coded using sum-to-zero contrasts, such that coefficients represent deviations from the overall mean. The category Other is not displayed and can be derived as the negative sum of the reported coefficients. Model M3.7 instead controlled for county-level indicators related to EWE severity and impacts, specifically the abnormality score for the number of EWE episodes (abnormality_final), the amount of financial damage (sum_damage), and the number of injuries (sum_injuries) and deaths (sum_deaths) associated with each episode. The model further controlled for the temporal proximity of the most recent EWE episode (recency_index), as well as broader county characteristics: population density (population_density_peopPerSqKilometer), median household income (countyIncome), and the county-level Partisan Voting Index (countyPvi). All county-level variables included as controls in Model M3.7 were z-standardized, except recency_index. Model M3.8 included political affiliation, subjective attribution and a continuous, duration-weighted measure of EWE exposure (nEpisodes_durationWeighted), as well as all three two-way interactions and the*

three-way interaction. Confidence intervals are 95%. Statistically significant effects (p < .05) are shown in bold.

1.3.3. Multicollinearity tests

Previous research suggests that *polAff* and *subjAttr* covary (Ogunbode et al. 2019; Zanocco et al. 2024). Thus, including *polAff* and *subjAttr* in the same statistical model might raise concerns regarding multicollinearity. While multicollinearity does not affect coefficient estimates, it increases standard errors, thereby reducing statistical efficiency. Variance inflation factor (VIF) values are commonly reported to detect multicollinearity in statistical models, with higher values indicating higher multicollinearity. Importantly, however, if interaction terms are included in a model, high VIF values are necessary. This type of multicollinearity between the components of an interaction, known as inessential ill-conditioning, leads to inflated VIF values commonly observed in models with interaction terms (Francoeur 2013). Thus, VIF values are not informative of multicollinearity in models including interaction terms, which is why we checked VIF values only in models without interaction terms (see model M3.1).

Variance inflation factors were uniformly low (all VIFs = 1.01–1.10), suggesting that multicollinearity was negligible and unlikely to affect coefficient estimation.

```
$deltaDuration
# Check for Multicollinearity

Low Correlation

                       Term  VIF   VIF 95% CI adj. VIF Tolerance
             polAff_bin_str 1.10 [1.09, 1.11]     1.05      0.91
 nEpisodesIsGreaterThanZero 1.01 [1.00, 1.03]     1.00      0.99
          EWE_subjAttr_mean 1.10 [1.09, 1.11]     1.05      0.91
 Tolerance 95% CI
     [0.90, 0.92]
     [0.97, 1.00]
     [0.90, 0.92]

$choice
# Check for Multicollinearity

Low Correlation

                       Term  VIF   VIF 95% CI adj. VIF Tolerance
             polAff_bin_str 1.09 [1.08, 1.10]     1.04      0.92
 nEpisodesIsGreaterThanZero 1.01 [1.00, 1.03]     1.00      0.99
          EWE_subjAttr_mean 1.09 [1.08, 1.10]     1.04      0.92
 Tolerance 95% CI
     [0.91, 0.93]
     [0.97, 1.00]
     [0.91, 0.93]
```

1.4. Subjective Experiences of Extreme Weather Events

Next, we explored whether subjective experiences of EWE, rather than objective county-level exposure, were associated with ΔDuration and choice. The subjective exposure variable was highly right-skewed (M = 36.99, SD = 152.37, skew = 29.5). Therefore, one extreme outlier (5,258 events) was excluded, and remaining values were log-transformed.

First, we tested whether subjective experiences of EWE differed by political affiliation. Using log-transformed values, Democrats reported higher levels of EWE exposure than Republicans (M_{Dem} = 3.06, SD_{Dem} = 0.97, M_{Rep} = 2.87, SD_{Rep} = 1.01, $p < .001$), despite lower levels of objective exposure. Next, we re-estimated models for H2 and H3 using participants' self-reported number of experienced episodes. Subjective experience was positively associated with greater relative attention to carbon information (b = 0.02, p = .020) and pro-climate choices (OR = 1.46, $p < .001$).

However, subjective experience did not interact with political affiliation or subjective attribution when predicting ΔDuration. A marginal interaction between subjective exposure and political affiliation predicting pro-climate choices was observed (OR = 0.70, p = .045), indicating that partisan differences in pro-climate choices were attenuated at higher levels of subjective exposure. The three-way interaction between political affiliation, subjective EWE experience, and subjective attribution was not significant, indicating no joint moderation of ΔDuration or choice (see Table S18, Table S19, Table S20, Table S21 for all exploratory results).

	M4.1			M4.2		
Predictors	*Estimates*	*CI*	*p*	*Estimates*	*CI*	*p*
(Intercept)	-0.03	-0.09 – 0.03	0.381	-0.02	-0.10 – 0.06	0.602
polAff bin str [Dem]	0.08	0.04 – 0.12	**<0.001**	0.06	-0.06 – 0.19	0.338
EWE evTyps N sum log	0.02	0.00 – 0.04	**0.020**	0.02	-0.01 – 0.05	0.119
polAff bin str [Dem] × EWE evTyps N sum log				0.01	-0.03 – 0.04	0.801
**Random Effects**						
σ^2	0.29			0.29		
τ_{00}	0.13 $_{subject}$			0.13 $_{subject}$		
	0.00 $_{trialNum\_fixed}$			0.00 $_{trialNum\_fixed}$		
ICC	0.31			0.31		
N	1450 $_{subject}$			1450 $_{subject}$		
	25 $_{trialNum\_fixed}$			25 $_{trialNum\_fixed}$		
Observations	34269			34269		
Marginal R^2 / Conditional R^2	0.005 / 0.316			0.005 / 0.316		

	M4.3		
Predictors	*Estimates*	*CI*	*p*
(Intercept)	-0.05	-0.16 – 0.05	0.331
polAff bin str [Dem]	0.04	-0.08 – 0.17	0.484
EWE evTyps N sum log	0.01	-0.02 – 0.04	0.424
trialNum	0.00	0.00 – 0.00	**<0.001**
CB carbonUp	-0.04	-0.08 – -0.00	**0.035**
gender [Male]	-0.05	-0.09 – -0.01	**0.018**
age	0.00	0.00 – 0.00	**<0.001**
racethn [European]	-0.01	-0.05 – 0.03	0.512
racethn [Hispanic]	-0.05	-0.10 – -0.00	**0.042**
racethn [African]	-0.02	-0.11 – 0.07	0.685
racethn [Asian]	0.12	0.03 – 0.21	**0.012**
racethn [moreThanOne]	-0.02	-0.11 – 0.08	0.721
polAff bin str [Dem] × EWE evTyps N sum log	0.01	-0.03 – 0.05	0.760
**Random Effects**			
σ^2		0.29	
$\tau_{00\ subject}$		0.13	
$\tau_{00\ trialNum\_fixed}$		0.00	
ICC		0.31	
$N_{subject}$		1450	
$N_{trialNum\_fixed}$		25	
Observations		34269	
Marginal R^2 / Conditional R^2		0.016 / 0.318	

Table S18: ***Mixed-effects models predicting ΔDuration (exploratory analysis: M4.1-M4.3).*** *ΔDuration (deltaDuration) represents relative dwell time spent on carbon information compared to bonus information. Random intercepts were included for participants (subject) and trials (trialNum_fixed) (N = 1,451 participants). Model M4.1 included political affiliation (polAff_bin_str, subject-level binary variable with 0 = Republican and 1 = Democrat) and subjective EWE exposure (EWE_evTyps_N_sum_log). Model M4.2 additionally included their interaction. Model M4.3 further controlled for trial position (trialNum), the position of the carbon attribute (CB_carbonUp), gender, age and race/ethnicity (racethn). racethn was effect-coded using sum-to-zero contrasts, such that coefficients represent deviations from the overall mean. The category Other is not displayed and can be derived as the negative sum of the reported coefficients. Estimates represent unstandardized beta coefficients. Confidence intervals are 95%. Statistically significant effects (p < .05) are shown in bold.*

	M4.1			M4.2		
Predictors	*Odds Ratios*	*CI*	*p*	*Odds Ratios*	*CI*	*p*
(Intercept)	0.31	0.18 – 0.54	**<0.001**	0.23	0.12 – 0.46	**<0.001**
polAff bin str [Dem]	1.95	1.43 – 2.67	**<0.001**	3.85	1.42 – 10.45	**0.008**
EWE evTyps N sum log	1.46	1.25 – 1.71	**<0.001**	1.62	1.31 – 2.00	**<0.001**
polAff bin str [Dem] × EWE evTyps N sum log				0.80	0.58 – 1.10	0.161
**Random Effects**						
σ^2	3.29			3.29		
τ_{00}	8.38 $_{subject}$			8.37 $_{subject}$		
	0.24 $_{trialNum\_fixed}$			0.24 $_{trialNum\_fixed}$		
ICC	0.72			0.72		
N	1450 $_{subject}$			1450 $_{subject}$		
	25 $_{trialNum\_fixed}$			25 $_{trialNum\_fixed}$		
Observations	34403			34403		
Marginal R^2 / Conditional R^2	0.023 / 0.730			0.023 / 0.730		

	M4.3		
Predictors	*Odds Ratios*	*CI*	*p*
(Intercept)	0.09	0.04 – 0.21	**<0.001**
polAff bin str [Dem]	4.03	1.49 – 10.87	**0.006**
EWE evTyps N sum log	1.50	1.21 – 1.86	**<0.001**
carbon prcnt	1.01	1.01 – 1.01	**<0.001**
bonus prcnt	0.99	0.99 – 0.99	**<0.001**
age	1.02	1.01 – 1.03	**<0.001**
racethn [European]	1.09	0.79 – 1.50	0.614
racethn [Hispanic]	1.17	0.79 – 1.72	0.434
racethn [African]	0.30	0.15 – 0.61	**0.001**
racethn [Asian]	2.06	0.98 – 4.33	0.056
racethn [moreThanOne]	1.12	0.51 – 2.44	0.784
polAff bin str [Dem] × EWE evTyps N sum log	0.79	0.57 – 1.08	0.144
**Random Effects**			
σ^2		3.29	
$\tau_{00\ subject}$		8.13	
$\tau_{00\ trialNum\_fixed}$		0.02	
ICC		0.71	
$N_{subject}$		1450	
$N_{trialNum\_fixed}$		25	
Observations		34403	
Marginal R^2 / Conditional R^2		0.059 / 0.730	

Table S19: ***Mixed-effects logistic regression models predicting choice (exploratory analysis: M4.1-M4.3).*** *choice represents the actual decision in the Carbon Emission Task (trial-level variable with 0 = pro-self and 1 = pro-climate). Random intercepts were included for participants (subject) and trials (trialNum_fixed) (N = 1,451 participants). Model M4.1 included political affiliation (polAff_bin_str, subject-level binary variable with 0 = Republican and 1 = Democrat) and subjective EWE exposure (EWE_evTyps_N_sum_log). Model M4.2 additionally included their interaction. Model M4.3 further controlled for attribute differences (carbon_prcnt and bonus_prcnt), age and race/ethnicity (racethn). racethn was effect-coded using sum-to-zero contrasts, such that coefficients represent deviations from the overall mean. The category Other is not displayed and can be derived as the negative sum of the reported coefficients. Confidence intervals are 95%. Statistically significant effects (p < .05) are shown in bold.*

	M5.1			M5.2		
Predictors	*Estimates*	*CI*	*p*	*Estimates*	*CI*	*p*
(Intercept)	-0.22	-0.32 – -0.12	**<0.001**	-0.11	-0.21 – -0.02	**0.020**
polAff bin str [Dem]	0.05	0.01 – 0.09	**0.025**	-0.25	-0.47 – -0.03	**0.024**
EWE evTyps N sum log	0.02	-0.00 – 0.04	0.054			
EWE subjAttr mean	0.05	0.03 – 0.07	**<0.001**	0.04	0.02 – 0.06	**0.001**
polAff bin str [Dem] × EWE subjAttr mean				0.07	0.02 – 0.12	**0.006**
**Random Effects**						
σ^2	0.29			0.29		
τ_{00}	0.13 $_{subject}$			0.13 $_{subject}$		
	0.00 $_{trialNum\_fixed}$			0.00 $_{trialNum\_fixed}$		
ICC	0.31			0.31		
N	1450 $_{subject}$			1450 $_{subject}$		
	25 $_{trialNum\_fixed}$			25 $_{trialNum\_fixed}$		
Observations	34269			34269		
Marginal R^2 / Conditional R^2	0.011 / 0.316			0.012 / 0.316		

	M5.3			M5.4		
Predictors	*Estimates*	*CI*	*p*	*Estimates*	*CI*	*p*
(Intercept)	-0.18	-0.41 – 0.05	0.133	-0.12	-0.36 – 0.11	0.302
EWE evTyps N sum log	0.00	-0.08 – 0.08	0.959	0.00	-0.07 – 0.08	0.903
EWE subjAttr mean	0.05	-0.01 – 0.10	0.106	0.03	-0.03 – 0.09	0.374
EWE evTyps N sum log × EWE subjAttr mean	0.00	-0.01 – 0.02	0.624	0.00	-0.02 – 0.02	0.694
polAff bin str [Dem]				-0.22	-0.46 – 0.02	0.077
polAff bin str [Dem] × EWE evTyps N sum log				-0.01	-0.05 – 0.03	0.674
polAff bin str [Dem] × EWE subjAttr mean				0.07	0.02 – 0.12	**0.010**
**Random Effects**						
σ^2		0.29			0.29	
τ_{00}		0.13 subject			0.13 subject	
		0.00 trialNum_fixed			0.00 trialNum_fixed	
ICC		0.31			0.31	
N		1450 subject			1450 subject	
		25 trialNum_fixed			25 trialNum_fixed	
Observations		34269			34269	
Marginal R^2 / Conditional R^2		0.010 / 0.316			0.012 / 0.317	

	M5.5		
Predictors	*Estimates*	*CI*	*p*
(Intercept)	-0.17	-0.43 – 0.10	0.220
polAff bin str [Dem]	-0.04	-0.61 – 0.54	0.901
EWE evTyps N sum log	0.02	-0.07 – 0.11	0.664
EWE subjAttr mean	0.04	-0.03 – 0.10	0.270
polAff bin str [Dem] × EWE evTyps N sum log	-0.08	-0.27 – 0.12	0.453
polAff bin str [Dem] × EWE subjAttr mean	0.02	-0.11 – 0.16	0.718
EWE evTyps N sum log × EWE subjAttr mean	-0.00	-0.02 – 0.02	0.996
(polAff bin str [Dem] × EWE evTyps N sum log) × EWE subjAttr mean	0.02	-0.03 – 0.06	0.499
**Random Effects**			
σ^2	0.29		
$\tau_{00\ subject}$	0.13		
$\tau_{00\ trialNum\_fixed}$	0.00		
ICC	0.31		
$N_{subject}$	1450		
$N_{trialNum\_fixed}$	25		
Observations	34269		
Marginal R^2 / Conditional R^2	0.012 / 0.317		

	M5.6		
Predictors	*Estimates*	*CI*	*p*
(Intercept)	-0.39	-0.67 – -0.11	**0.007**
polAff bin str [Dem]	0.08	-0.49 – 0.65	0.781
EWE evTyps N sum log	0.03	-0.06 – 0.11	0.562
EWE subjAttr mean	0.08	0.01 – 0.15	**0.020**
trialNum	0.00	0.00 – 0.00	**<0.001**
CB carbonUp	-0.04	-0.08 – -0.00	**0.043**
gender [Male]	-0.04	-0.08 – -0.00	**0.048**
age	0.00	0.00 – 0.00	**<0.001**
racethn [European]	-0.00	-0.04 – 0.04	0.980
racethn [Hispanic]	-0.06	-0.11 – -0.01	**0.014**
racethn [African]	-0.03	-0.11 – 0.06	0.560
racethn [Asian]	0.11	0.02 – 0.20	**0.017**
racethn [moreThanOne]	-0.03	-0.12 – 0.07	0.608
polAff bin str [Dem] × EWE evTyps N sum log	-0.07	-0.26 – 0.13	0.487
polAff bin str [Dem] × EWE subjAttr mean	-0.01	-0.14 – 0.12	0.852
EWE evTyps N sum log × EWE subjAttr mean	-0.01	-0.03 – 0.02	0.631
(polAff bin str [Dem] × EWE evTyps N sum log) × EWE subjAttr mean	0.02	-0.03 – 0.06	0.491
**Random Effects**			
σ^2		0.29	
$\tau_{00\ subject}$		0.12	
$\tau_{00\ trialNum\_fixed}$		0.00	
ICC		0.30	
$N_{subject}$		1450	
$N_{trialNum\_fixed}$		25	
Observations		34269	
Marginal R^2 / Conditional R^2		0.027 / 0.318	

Table S20: ***Mixed-effects models predicting ΔDuration (exploratory analysis: M5.1-M5.6).*** *ΔDuration (deltaDuration) represents relative dwell time spent on carbon information compared to bonus information. Random intercepts were included for participants (subject) and trials (trialNum_fixed) (N = 1,451 participants). In Model M5.1, political affiliation (polAff_bin_str), subjective EWE exposure*

(EWE_evTyps_N_sum_log), and subjective attribution of EWE to climate change (EWE_subAttr_mean) were included as predictors of ΔDuration. Model M5.2 included political affiliation, subjective attribution and their interaction. Model M5.3 included subjective EWE exposure and subjective attribution and their interaction. Model M5.4 included political affiliation, subjective EWE exposure, and subjective attribution, as well as all three two-way interactions. Model M5.5 additionally included the three-way interaction between political affiliation, subjective EWE exposure, and subjective attribution. Model M5.6 further controlled for trial position (trialNum), the position of the carbon attribute (CB_carbonUp), gender, age and race/ethnicity (racethn). racethn was effect-coded using sum-to-zero contrasts, such that coefficients represent deviations from the overall mean. The category Other is not displayed and can be derived as the negative sum of the reported coefficients. Estimates represent unstandardized beta coefficients. Confidence intervals are 95%. Statistically significant effects ($p < .05$) are shown in bold.

	M5.1			M5.2		
Predictors	*Odds Ratios*	*CI*	*p*	*Odds Ratios*	*CI*	*p*
(Intercept)	0.10	0.04 – 0.23	**<0.001**	0.38	0.17 – 0.83	**0.015**
polAff bin str [Dem]	1.66	1.20 – 2.29	**0.002**	0.20	0.03 – 1.22	0.081
EWE evTyps N sum log	1.43	1.22 – 1.68	**<0.001**			
EWE subjAttr mean	1.36	1.15 – 1.60	**<0.001**	1.26	1.04 – 1.52	**0.016**
polAff bin str [Dem] × EWE subjAttr mean				1.63	1.09 – 2.46	**0.018**
**Random Effects**						
σ^2	3.29			3.29		
τ_{00}	8.31 $_{subject}$			8.39 $_{subject}$		
	0.24 $_{trialNum\_fixed}$			0.24 $_{trialNum\_fixed}$		
ICC	0.72			0.72		
N	1450 $_{subject}$			1450 $_{subject}$		
	25 $_{trialNum\_fixed}$			25 $_{trialNum\_fixed}$		
Observations	34403			34403		
Marginal R^2 / Conditional R^2	0.030 / 0.730			0.023 / 0.730		

	M5.3			M5.4		
Predictors	*Odds Ratios*	*CI*	*p*	*Odds Ratios*	*CI*	*p*
(Intercept)	0.12	0.02 – 0.82	**0.030**	0.20	0.03 – 1.35	0.098
EWE evTyps N sum log	1.29	0.69 – 2.43	0.421	1.26	0.67 – 2.37	0.465
EWE subjAttr mean	1.35	0.86 – 2.12	0.189	1.04	0.65 – 1.68	0.862
EWE evTyps N sum log × EWE subjAttr mean	1.03	0.89 – 1.19	0.718	1.06	0.91 – 1.25	0.432
polAff bin str [Dem]				0.61	0.09 – 4.39	0.627
polAff bin str [Dem] × EWE evTyps N sum log				0.70	0.50 – 0.99	**0.045**
polAff bin str [Dem] × EWE subjAttr mean				1.58	1.05 – 2.38	**0.028**
**Random Effects**						
σ^2	3.29			3.29		
τ_{00}	8.36 $_{subject}$			8.26 $_{subject}$		
	0.24 $_{trialNum\_fixed}$			0.24 $_{trialNum\_fixed}$		
ICC	0.72			0.72		
N	1450 $_{subject}$			1450 $_{subject}$		
	25 $_{trialNum\_fixed}$			25 $_{trialNum\_fixed}$		
Observations	34403			34403		
Marginal R^2 / Conditional R^2	0.025 / 0.730			0.034 / 0.730		

	M5.5		
Predictors	*Odds Ratios*	*CI*	*p*
(Intercept)	0.25	0.03 – 2.20	0.213
polAff bin str [Dem]	0.21	0.00 – 22.41	0.509
EWE evTyps N sum log	1.16	0.56 – 2.38	0.695
EWE subjAttr mean	0.98	0.57 – 1.68	0.929
polAff bin str [Dem] × EWE evTyps N sum log	1.05	0.21 – 5.16	0.953
polAff bin str [Dem] × EWE subjAttr mean	2.04	0.70 – 5.93	0.192
EWE evTyps N sum log × EWE subjAttr mean	1.09	0.91 – 1.31	0.351
(polAff bin str [Dem] × EWE evTyps N sum log) × EWE subjAttr mean	0.91	0.64 – 1.31	0.616
**Random Effects**			
σ^2		3.29	
$\tau_{00\ subject}$		8.25	
$\tau_{00\ trialNum\_fixed}$		0.24	
ICC		0.72	
$N_{subject}$		1450	
$N_{trialNum\_fixed}$		25	
Observations		34403	
Marginal R^2 / Conditional R^2		0.034 / 0.730	

	M5.6		
Predictors	*Odds Ratios*	*CI*	*p*
(Intercept)	0.09	0.04 – 0.21	**<0.001**
polAff bin str [Dem]	4.03	1.49 – 10.87	**0.006**
EWE evTyps N sum log	1.50	1.21 – 1.86	**<0.001**
carbon prcnt	1.01	1.01 – 1.01	**<0.001**
bonus prcnt	0.99	0.99 – 0.99	**<0.001**
age	1.02	1.01 – 1.03	**<0.001**
racethn [European]	1.09	0.79 – 1.50	0.614
racethn [Hispanic]	1.17	0.79 – 1.72	0.434
racethn [African]	0.30	0.15 – 0.61	**0.001**
racethn [Asian]	2.06	0.98 – 4.33	0.056
racethn [moreThanOne]	1.12	0.51 – 2.44	0.784
polAff bin str [Dem] × EWE evTyps N sum log	0.79	0.57 – 1.08	0.144
**Random Effects**			
σ^2		3.29	
$\tau_{00\ subject}$		8.13	
$\tau_{00\ trialNum\_fixed}$		0.02	
ICC		0.71	
$N_{subject}$		1450	
$N_{trialNum\_fixed}$		25	
Observations		34403	
Marginal R^2 / Conditional R^2		0.059 / 0.730	

Table S21: ***Mixed-effects logistic regression models predicting choice (exploratory analysis: M5.1-M5.6).*** *Choice represents the actual decision in the Carbon Emission Task (trial-level variable with 0 = pro-self and 1 = pro-climate). Random intercepts were included for participants (subject) and trials (trialNum_fixed) (N = 1,451 participants). In Model M5.1, political affiliation (polAff_bin_str), subjective EWE exposure (EWE_evTyps_N_sum_log), and subjective attribution of EWE to climate change (EWE_subAttr_mean) were included as predictors of choice. Model M5.2 included political affiliation, subjective attribution and their interaction. Model M5.3 included subjective EWE exposure and subjective attribution and their interaction. Model M5.4 included political affiliation, subjective EWE exposure, and subjective attribution, as well as all three two-way interactions. Model M5.5 additionally included the three-way interaction between political affiliation, subjective EWE exposure, and subjective attribution. Model M5.6 further controlled for attribute differences (carbon_prcnt and bonus_prcnt), age and race/ethnicity (racethn). racethn was effect-coded using sum-to-zero contrasts, such that coefficients represent deviations from the overall mean. The category Other is not displayed and can be derived as the negative sum of the reported coefficients. Confidence intervals are 95%. Statistically significant effects (p < .05) are shown in bold.*

2. Methods

2.1. Procedure

Participants were first directed from the panel provider's page (MSI-ACI's FlexPanel) to a survey hosted in Qualtrics. After passing quality and attention checks, they were directed to a decision-making experiment programmed in MouselabWEB (Willemsen and Johnson 2019), which tracked their information search processes. Following the experiment, participants were informed which trial was randomly selected to be implemented and received details about their corresponding bonus payment and the amount of carbon emitted as a result of their choices. They then returned to the Qualtrics survey to answer questions about the experiment, their experience with extreme weather events, their climate change concern, political affiliation, and demographic information. Participants received a flat participation fee of 95 cents USD from the panel provider, with the opportunity to earn an additional bonus of 30 to 96 cents USD based on their decisions during the task (see Carbon Emission Task: Trials below).

2.2. Survey Structure and Questions

Table S22 shows the general structure of the survey as well as specific questions.

**Block Name**	**Exact Questions**	**Answer Options**
detectMobile	It seems like you are using a [device]. Please switch to a desktop/laptop.	- No action<br>- Switch to desktop/laptop<br>- Adjust screen resolution
Captcha	Please complete the captcha below.	- Captcha input field
InformationConsent	Welcome to this study on decision-making! Do you consent to participate?	- I have read and understood and would like to participate<br>- I do not wish to participate
Welcome	Dear Participant, To conduct the experiment calmly, please close other programs.	- No response needed; instructions only
AttentionChecks	What is your year of birth? /<br>What does the current study investigate? /<br>What is your age? /<br>Choose 'Somewhat disagree' /	- Free-text entry for year of birth<br>- Multiple-choice question on study topic<br>- Free-text entry for age<br>- Multiple-choice on instructed response
handleIDs	Thank you for answering the questions.	- No response needed; system logs timing data
Experiment	You can go to the experiment now by clicking the button below.	- Button to start experiment<br>- Password input field<br>- Button to restart experiment
QuestionsExperiment	What kind of device did you use to display the experiment? / How did you complete the experiment? / What type of mouse did you use? / Did you experience any technical problems during the experiment? / Please describe the technical problems you experienced: / Please indicate your level of agreement with the following statement: Buying and retiring carbon emission certificates is an effective means to reduce carbon emissions.	- for device: “Laptop's internal screen“, “External monitor“, ”Tablet“, “Mobile phone“, “Other (specify)“<br>- for how completing the experiment: “With a mouse“, “With a trackpad“, “With a touchscreen“<br>- for type of mouse: “Mouse with a cable“, “Wireless mouse (e.g., using Bluetooth or USB plug)“, “Other (specify)“<br>- for technical problems: yes-no question and free-text entry<br>- for agreement to carbon emission certificates: 5-point Likert scale ranging from “strongly disagree” to “strongly agree”

**Block Name**	**Exact Questions**	**Answer Options**
extremeWeatherExperiences	If any, which of the following extreme weather events did you experience in the past days? To access definitions of the different event types, click Info. / How often? / How much did they affect you? / Please indicate how many times you experienced each event type in the past days.	- Checklist of extreme weather events<br>- Numeric input for frequency<br>- Likert scale for impact
personalEWE	My current or previous property was affected by an extreme weather event. / I experienced travel disruption or disruption to ability to work as a result of an extreme weather event. / I experienced disruption of essential services such as gas, electricity, water supply, drains, telephone or internet as a result of an extreme weather event. / Other people in my local area experienced damage to their property from an extreme weather event.	- Yes-no questions
indirectEWE	Did you read or hear (through newspapers, online sources, social media, TV, radio, or other sources) about one or more extreme weather events in the past 120 days (i.e. last four months) in your county of residence or your state of residence or other states of the U.S.? /<br>Were any of your significant others (e.g., family, friends, or colleagues) affected by one or more extreme weather events in the past 120 days (i.e. last four months)?	- for hear/read: 3 yes-no responses for my county of residence, my state of residence, other states of the U.S.<br>- for significant others: 4 yes-no responses for family, friends, colleagues, others [please specify]
subjectiveAttribution	Extreme weather events are caused in part by climate change. / Extreme weather events are a sign that impacts of climate change are happening now. / Extreme weather events show us what we can expect from climate change in the future.	- 5-point Likert scales ranging from “strongly disagree” to “strongly agree”
climateChangeConcern	In general, how concerned are you about climate change?	- slider scale ranging from 0 to 100 with labels “not at all concerned (0)”, “not very concerned (25)”, “somewhat concerned (50)”, “very concerned (75)”, and “extremely concerned (100)”
politicalOrientation	Generally speaking, do you think of yourself as a Democrat, Republican or Independent? /	- “Strong Democrat”, “Not very strong Democrat”, “Independent, close to Democrat”, “Independent”, “Independent, close to Republican”, “Not very strong

Block Name	Exact Questions	Answer Options
	In case "Independent" was chosen, participants were additionally asked: If you had to identify with one party of the two parties, which one would you choose?	Republican", "Strong Republican"<br>- "Democratic Party" and "Republican Party" if Independent was chosen
Demographics	For statistical purposes, what is the gender you identify with? /<br>What is the highest level of education that you have completed and received credit for? /<br>Last year, that is in 2024, what was your total family income from all sources, before taxes? /<br>What is your cultural background? Choose all that apply. /<br>How would you describe the area where you live?	- for sex: "Male", "Female", "Other (specify)"<br>- for education: "Did not complete high school / GED", "High school diploma / GED", "Some college", "Associate's degree", "Bachelor's degree", "Master's degree or higher"<br>- for income: "Under $30,000", "$30,000 - $49,999", "$50,000 - $74,999", "$75,000 - $99,999", "$100,000 - $149,999", "More than $150,000"<br>- for cultural background: "European", "African", "East Asian", "South Asian", "South East Asian", "Hispanic or Latinx", "Middle Eastern", "First Nations or Indigeneous (specify)", "Other (specify)"<br>- for area of residency: "Urban, a big city", "Suburb, outskirt of a big city", "Rural town or small city"
FIPS	Please provide your State, County, and 5-digit FIPS code.	- Free-text entry for FIPS code, State, and County
Thanks	Do you have any suggestions or comments on the study?	- Free-text entry for feedback

Table S22: ***Structure of survey and specific survey questions***

2.3. Carbon Emission Task

2.3.1. Carbon Emission Task: Everyday Examples

When shopping for groceries, consumers face trade-offs between the lower price of conventional products (e.g., cow milk) and the higher cost of environmentally sustainable alternatives (e.g., oat milk). Similarly, transportation decisions also require balancing personal costs with environmental impact. Ride-hailing services like Uber now display estimated carbon emissions for different travel options, allowing users to compare a cheaper, high-emission car ride with a more sustainable but potentially less convenient public transit alternative.

2.3.2. Carbon Emission Task: Trials

Table S23 shows all trials in the new variant of the Carbon Emission Task (CET). Each row corresponds to one trial. *Carbon Diff.* and *Bonus Diff.* index the unique combinations of relative differences in carbon and bonus consequences in a trial. *Carbon Level Rand.* and *Bonus Level Rand.* correspond to the random carbon and bonus level used in the construction of a trial. The average carbon level over all trials is 19.85 lbs CO2, to which we add a random deviation with mean = 0 and SD = 0.9925 lbs CO_2. The average bonus level over all trials is 60 cents, to which we add a random deviation with mean = 0 and SD = 3 cents USD. *Carbon Option Pro-Self* and *Bonus Option Pro-Self* represent the actual information available to participants related to the option that will lead to the greater bonus payment for participants. *Carbon Option Pro-Climate* and *Bonus Option Pro-Self* represent the same information but for the option leading to the greater benefit for the climate (lower carbon emissions).

Carbon Diff (%)	Bonus Diff (%)	Carbon Level Rand (lbs.CO2)	Bonus Level Rand (cent USD)	Carbon Option Pro-Self (lbs.CO2)	Carbon Option Pro-Climate (lbs.CO2)	Bonus Option Pro-Self (cent USD)	Bonus Option Pro-Climate (cent USD)
10	10	20.54	60.33	21.56	19.51	63	57
10	15	19.80	63.84	20.79	18.81	69	59
10	20	19.89	57.64	20.89	18.90	63	52
10	50	20.78	58.24	21.82	19.74	73	44
10	100	20.44	62.12	21.46	19.42	93	31
15	10	20.14	59.66	21.66	18.63	63	57
15	15	19.37	56.33	20.83	17.92	61	52
15	20	18.69	63.63	20.09	17.29	70	57
15	50	19.47	63.24	20.93	18.01	79	47
15	100	18.37	64.15	19.75	16.99	96	32
20	10	19.27	60.23	21.20	17.34	63	57
20	15	18.55	63.58	20.40	16.69	68	59
20	20	19.31	63.65	21.24	17.38	70	57
20	50	19.56	60.05	21.52	17.60	75	45
20	100	20.38	63.13	22.42	18.34	95	32
50	10	19.30	57.58	24.13	14.48	60	55
50	15	20.82	55.45	26.02	15.61	60	51
50	20	20.46	59.72	25.58	15.35	66	54
50	50	20.89	57.35	26.12	15.67	72	43
50	100	21.21	60.94	26.52	15.91	91	30
100	10	19.89	60.80	29.83	9.94	64	58
100	15	20.54	61.23	30.81	10.27	66	57
100	20	19.54	65.30	29.30	9.77	72	59
100	50	20.50	60.10	30.75	10.25	75	45
100	100	19.04	63.07	28.57	9.52	95	32

*Table S23: **Trials in the new variant of the CET***

In response to a reviewer's feedback, we implemented five additional trials with an inverted incentive structure to reduce predictability and potential learning effects (Table S24). In these trials, the initial pro-self option is now associated with lower emissions and higher bonus amounts – a deliberate modification of the initial structure. These trials are designed to encourage attentive processing, reduce reliance on heuristics (e.g., "more money = more emissions"), and maintain cognitive engagement throughout the task.

To ensure structured variation across the inverted trials, we began by stratifying the original trial set into five blocks based on the size of the carbon difference between options (10%, 15%, 20%, 50%, and 100%). From each block, we randomly selected one trial. In these selected trials (see Table S24), we swapped the CO_2 values of the pro-self and pro-climate options while keeping the bonus amounts unchanged. This means that in the inverted versions, the pro-self option offers both a higher bonus and lower emissions, thus intentionally contrasting with the structure of the original task. Crucially, this inversion preserves the absolute and relative differences in emissions and incentives, maintaining

the comparability of trade-off strength across conditions. At the same time, it introduces controlled irregularities that disrupt predictable patterns (e.g., “more money = more emissions”) and reduces the strategy-driven (or heuristic) responding over time.

Carbon Diff (%)	Bonus Diff (%)	Carbon Level Rand (lbs.CO2)	Bonus Level Rand (cent USD)	Carbon Option Pro-Self Inverted (lbs.CO2)	Carbon Option Pro-Climate Inverted (lbs.CO2)	Bonus Option Pro-Self (cent USD)	Bonus Option Pro-Climate (cent USD)
10	15	19.80	63.84	18.81	20.79	69	59
15	20	18.69	63.63	17.29	20.09	70	57
20	10	19.27	60.23	17.34	21.20	63	57
50	15	20.82	55.45	15.61	26.02	60	51
100	20	19.54	65.30	9.77	29.30	72	59

Table S24: ***Additional inverted trials in the new variant of the CET***

2.4. Process-tracing with MouselabWEB

While we focus on ΔDuration and Choice in the present analyses, the process-tracing paradigm also yielded additional measures that may be explored for exploratory reasons to provide further insights into information search and attention processes during climate-relevant decision-making. These additional measures are described here.

The frequency of (re-)visiting each piece of information provides an alternative way of assessing the amount of attention and, by extension, the weight this information receives in participants’ decision-making processes, with more frequent acquisitions indicating more attention and decision-weight (Willemsen and Johnson 2019; Rahal and Fiedler 2019). For each trial, we assessed ΔFrequency as follows (with f representing the number of times a specific piece of information was acquired):

$$\Delta Frequency = \frac{(f_{Carbon_A} + f_{Carbon_B}) - (f_{Bonus_A} + f_{Bonus_B})}{f_{Carbon_A} + f_{Carbon_B} + f_{Bonus_A} + f_{Bonus_B}}$$

Other established process-tracing measures focus on transitions between different pieces of information. Most notably, the Payne Index combines the number and kind of transitions into a single measure reflecting comparison or information integration processes during decision-making (Willemsen and Johnson 2019; Reeck, Wall, and Johnson 2017). For each trial, we assessed the Payne Index as follows, with trans reflecting the number of transitions within options (e.g., between bonus and carbon attribute of Option A) or between options (e.g., between the bonus attributes of Option A and Option B):

$$\text{Payne Index} = \frac{trans_{withinOptions} - trans_{betweenOptions}}{trans_{withinOptions} + trans_{betweenOptions}}$$

As for ΔDuration and ΔFrequency, the Payne Index varies between -1 and +1, with negative values indicating more between options search patterns (i.e., more comparative information processing) and positive values indicating more within options search patterns (i.e., more integrative information processing).

Moreover, process-tracing data also contain information on the order in which information is searched for, i.e., when in the decision-making process a specific piece of information is attended to. First and last acquisitions, i.e., information that is acquired at the beginning of the decision-making process and the last piece of information acquired right before choice, have been interpreted as indexing preference-driven information weighting capable of predicting subsequent choice (Rahal and Fiedler 2019; Ghaffari and Fiedler 2018; Gwinn, Leber, and Krajbich 2019; Pärnamets et al. 2015). For each trial, we assessed the first and last acquisition as follows:

$$\text{First Acquisition} = \begin{cases} 0, & \text{if Bonus} \\ 1, & \text{if Carbon} \end{cases}$$

$$\text{Last Acquisition} = \begin{cases} 0, & \text{if Bonus} \\ 1, & \text{if Carbon} \end{cases}$$

2.5. Extreme Weather Data

2.5.1. Details About NOAA's Storm Events Database

We measured extreme weather exposure using data from NOAA's Storm Events Database, maintained by the NWS. This database archives (1) the occurrence of storms and other significant weather phenomena that are sufficiently intense to cause loss of life, injuries, significant property damage and/or disruption to commerce, (2) rare or unusual weather phenomena that generate media attention, and (3) other significant meteorological events, such as record maximum or minimum temperatures or precipitation (NWS 2016). The NWS gathers information from various sources, including state and local officials, emergency responders, media reports, insurance data, the general public, and NWS-trained "skywarn spotters". Each event in the database is validated and compiled and includes detailed records of start and end dates, geographic location with a resolution up to county-level, and any associated damages, injuries, or deaths.

In the database, each event is associated with an episode, and each episode typically includes multiple events. For instance, one extreme weather episode might encompass several contiguous excessive heat events in combination with wildfires that took place in a single county over several days. We conjectured that individuals are more likely to perceive such contiguous or cooccurring events as one coherent extreme weather episode and that exposure to such episodes might affect information search processes. For this reason, and to avoid over-counting of extreme weather events (Konisky, Hughes, and Kaylor 2016), we aggregated extreme weather events up to the episode level for our analyses. Note, however, that event-level data are still available for additional analyses investigating how different event types might differently affect information search and attention processes.

2.5.2. Extreme Weather Episode Variables for use in Sensitivity Analyses

Previous research found that the abnormality of extreme weather events relative to local historical activity is positively associated with attention to climate change on social media (Sisco, Bosetti, and Weber 2017). To account for this potential influence in sensitivity analyses, we calculated a score representing abnormal frequency of extreme weather episodes. To this end, we divided the number of episodes (E) in a given county (c), time interval (i), and year (Y) by the historical average for that time interval and county in the last ten years (Konisky, Hughes, and Kaylor 2016). The formula is:

$$abnormality_{raw}(E_{ciY}) = \frac{E_{ciY}}{E_{ci(Y-10,Y-1)}}$$

For example, if 4 episodes occurred in New Jersey in July 2023 and the historical average is 2, the abnormality ratio is 4/2=2. If two episodes occurred, the ratio is 1/1 = 1. If only 1 episode occurred, the ratio is 1/2=0.5. If the denominator was zero, we replaced it with 0.1 to avoid undefined scores. We then subtracted 1 from the raw ratio so that a score of zero indicates no abnormality. For example, a ratio of 1/1 → 0 shows no deviation, while 1/2 → -0.5 indicates fewer events than average., i.e., this subtraction also resulted in negative scores for below-average frequencies. Next, we took the absolute value of this adjusted score, making both deviations (above and below average) positive. This produced a skewed distribution, so we log-transformed the scores for normal distribution. We added 1 before the log transformation to avoid taking the logarithm of zero:

$$abnormality_{final} = log(|abnormality_{raw} - 1| + 1)$$

Another study found that when the severity of extreme weather events is considered, the time frame in which these events predict climate change concern can extend from 30 days up to 120 days before a survey (Konisky, Hughes, and Kaylor 2016). Following the same procedure as outlined in this study, we calculated a severity-weighted count of extreme weather episodes for the use in additional analyses. The duration of an extreme weather episode can be argued to be a reasonable indicator of the episode's severity, as long-lived episodes like a month-long drought might be perceived as more severe than short-lived episodes like a 10 minutes long hail event (Konisky, Hughes, and Kaylor 2016). Thus, for each extreme weather episode, we calculated the duration of extreme weather in days by using the earliest start and latest end dates of associated events. As in the original study (Konisky, Hughes, and Kaylor 2016), episodes lasting for less than a day were assigned a duration of 0.5 days. We then summed these durations to create a weighted count of episodes for the 30 days prior to each participant's study completion. As an alternative measure of extreme weather episodes' severity, we also calculated the total amount of financial damage (sum of *damage_property* and *damage_crops*), the total number of injuries (sum of *injuries_direct* and *injuries_indirect*), and the total number of deaths (sum of *deaths_direct* and *deaths_indirect*) associated with each episode based on records in the Storm Events Database. Additionally, more recent exposure to extreme weather episodes might impact information search processes differently compared to more distant exposure. To account for this, we calculated the number of days since the most recent

extreme weather episode in each participant's county of residence prior to study completion. Finally, while county-level occurrence of extreme weather episodes may indicate individual exposure to such episodes (Konisky, Hughes, and Kaylor 2016), this link might be stronger in densely populated counties than in sparsely populated ones. Therefore, we also considered each county's population density, measured in people per square kilometer (US Census Bureau 2023).

2.5.3. Risk Management

Predicting the specific type and timing of future extreme weather events remains difficult, which makes planning and preregistering studies on extreme weather exposure challenging. A possible concern is the potential lack of variability in extreme weather exposure between participants, due to the uncertainty of whether any significant extreme weather events will occur in temporal proximity to data collection. To mitigate this risk, we conducted the following analyses of extreme weather events prior to data collection. The following section is reproduced from the Supplementary Information submitted at Stage 1 and outlines this preparatory risk-management procedure.

2.5.3.1. Purpose & Rationale

As outlined in the Registered Report, we will assess the number of extreme weather episodes recorded in each participant's county of residence within the 30 days prior to study completion. Regarding the time window during which we plan to conduct the study, we aim for maximizing the likelihood of capturing suitable variability in the exposure to extreme weather episodes with notable geographic variability. To this end, we analyzed records of extreme weather episodes over the last ten years.

2.5.3.2. Filter Data

We filter the storm events data for the specific years, months, and extreme weather event types we are interested in. We filter for all years from 2014 to 2023 (as data are not complete for the year 2024 yet), we highlight the month of July, and we focus on those types of extreme weather events that are predicted to increase in frequency and severity due to climate change : Excessive Heat, Drought, Wildfire, Flash Flood, Coastal Flood, Strong Wind, Hail, and Tornado (IPCC 2023).

2.5.3.3. Analysis

Our analysis spanning the last ten years evidenced high numbers of occurrences of extreme weather episodes in every single year (Figure S15) as well as considerable geographical variability (Figure S16). Analyzing the seasonal distribution of extreme weather episodes, Figure S15 shows that July consistently shows a high number of extreme weather episodes over the last ten years, with an average occurrence of 1,777 episodes in each year (SD = 450). Note that numbers represent counts of distinct episode-county combinations to represent how many counties were affected. Table S25 shows the average number of extreme weather events by event type in July over the last ten years, demonstrating that July typically witnesses a multitude of different extreme weather event

types. Additionally, Figure S16 indicates that within the month of July, these extreme weather episodes also display a high geographical variability.

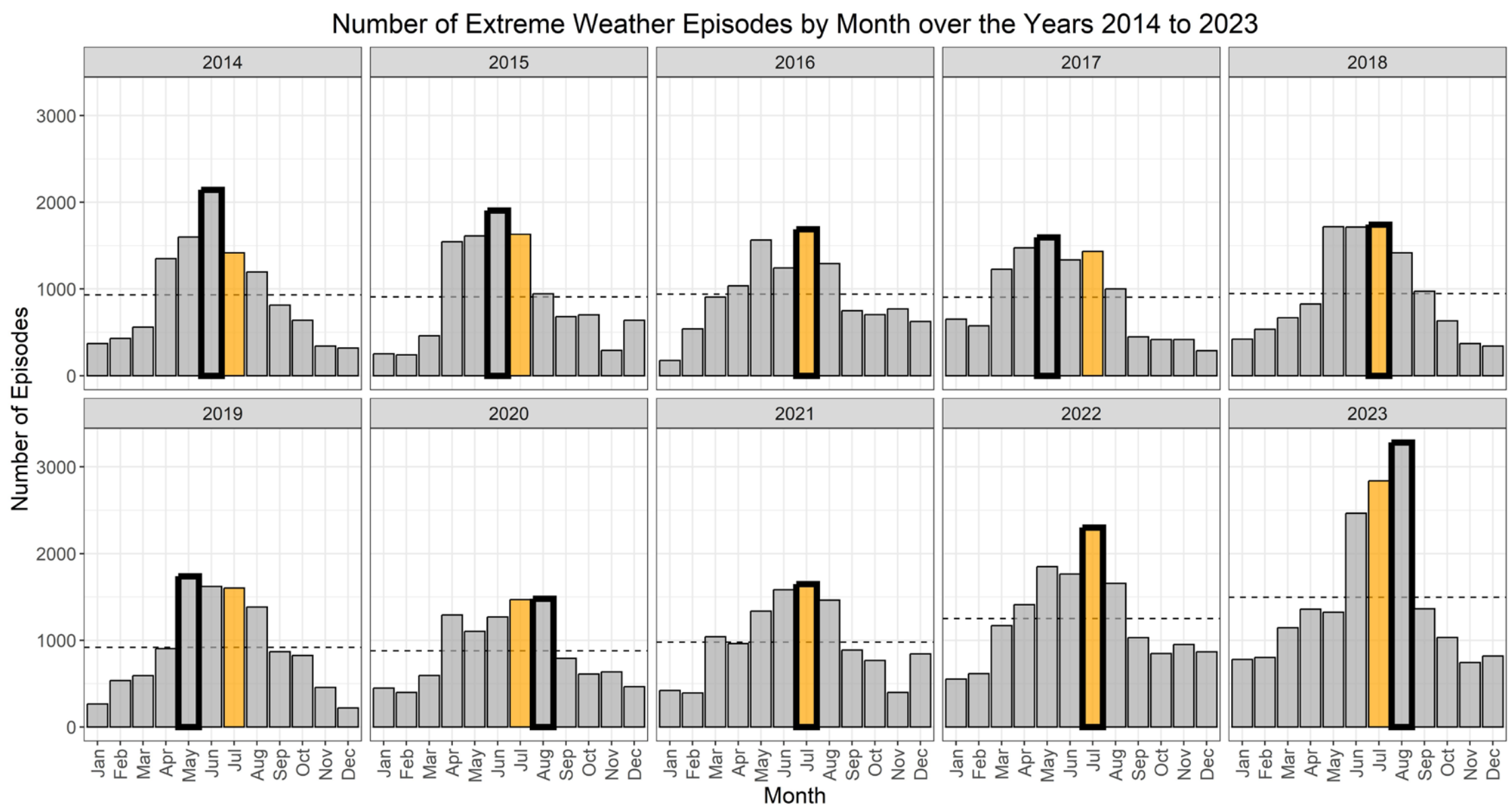

Figure S15: ***Number of extreme weather episodes by month over the years 2014 to 2023.*** *Histograms showing the number of extreme weather episodes by month from 2014 to 2023. The dashed horizontal line indicates the mean number of extreme weather episodes in each year. The thick-bordered bar marks the month with the most extreme weather events each year. The orange bar represents July. July had the most extreme weather events in 4 out of 10 years, and in another 4 years, it was right before or after the peak month. Only episodes that included at least one of the following event types were considered: excessive heat, drought, wildfire, flash flood, coastal flood, strong wind, hail, tornado.*

**Event Type**	**Mean Number of Events**	***SD***
Coastal Flood	5.43	3.31
Drought	272.70	254.42
Excessive Heat	429.50	346.85
Flash Flood	778.80	240.83
Hail	1249.70	383.11
Strong Wind	8.60	5.60
Tornado	107.60	20.08
Wildfire	106.90	33.62

Table S25: ***Mean number of extreme weather events in July over the years 2014 to 2023 by event type.***

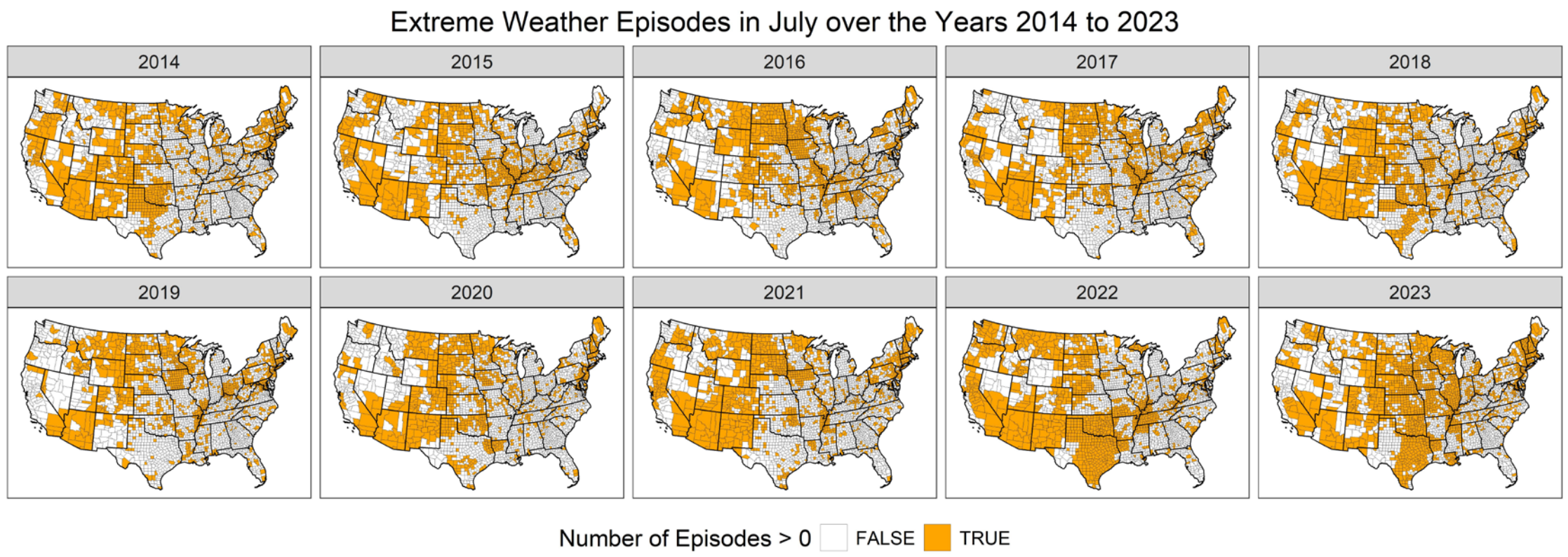

Figure S16: ***Extreme weather episodes in July over the years 2014 to 2023.*** *Maps displaying the geographical distribution of the occurrence of at least one extreme weather episode in July over the years 2014 to 2023. Only episodes that included at least one of the following event types were considered: excessive heat, drought, wildfire, flash flood, coastal flood, strong wind, hail, tornado. Map boundaries are based on the U.S. Census Bureau Cartographic Boundary Files and were visualized using the R package usmap.*

While Figure S16 visualizes the occurrence of at least one extreme weather episode in July for each county and year (binary variable), Figure S17 displays the actual number of such episodes (continuous). The vast majority of counties were exposed to few episodes, indicating that most of the variability is due to whether an extreme weather episode occurred at all or not. This is further supported by Figure S18 showing histograms for the number of extreme weather episodes in July over the past ten years. Most counties reported either zero or one extreme weather episode in July, and the ratio of counties experiencing no episodes to counties experiencing at least one episode seems to gradually approach 1:1. In July 2023, for instance, this ratio reached 1.02, with 50.43% of counties being exposed to zero and 49.57% of counties being exposed to at least one extreme weather episode.

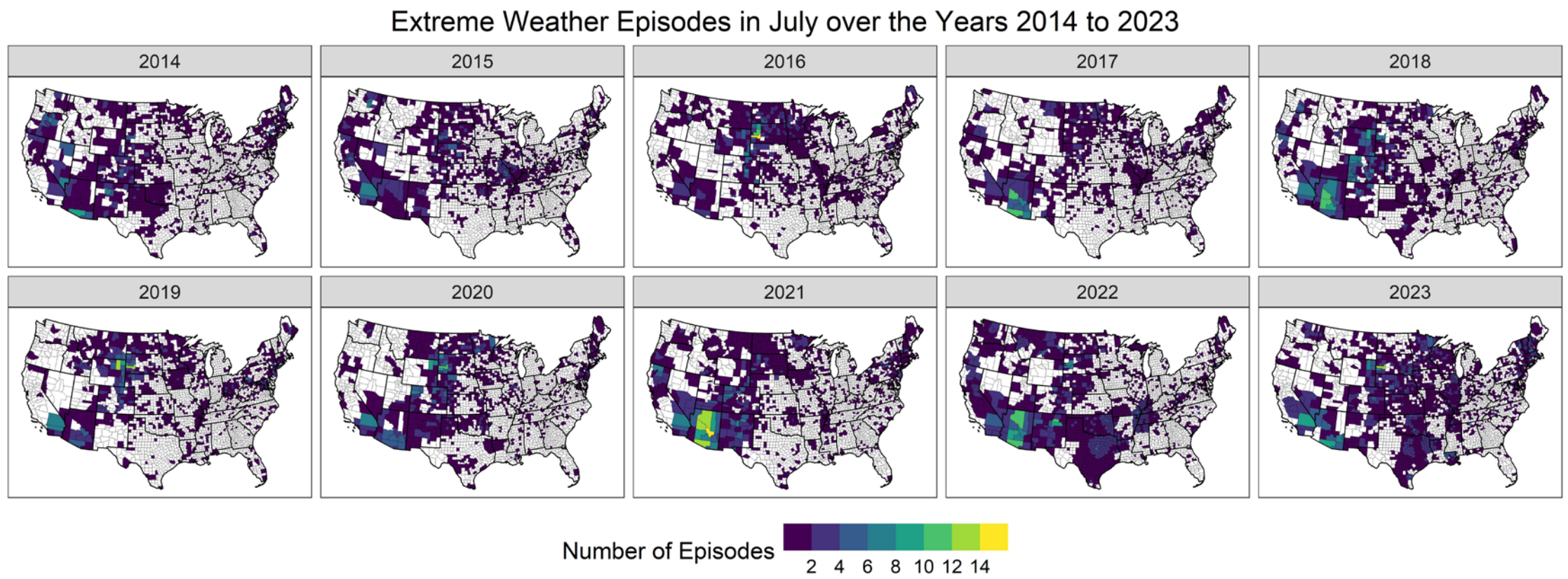

Figure S17: ***Extreme weather episodes in July over the years 2014 to 2023.*** *Maps displaying the geographical distribution of the raw number of extreme weather episodes in July over the years 2014 to 2023. The color palette indicates numbers greater than zero, and white represent a count of zero episodes. Map boundaries are based on the U.S. Census Bureau Cartographic Boundary Files and were visualized using the R package usmap.*

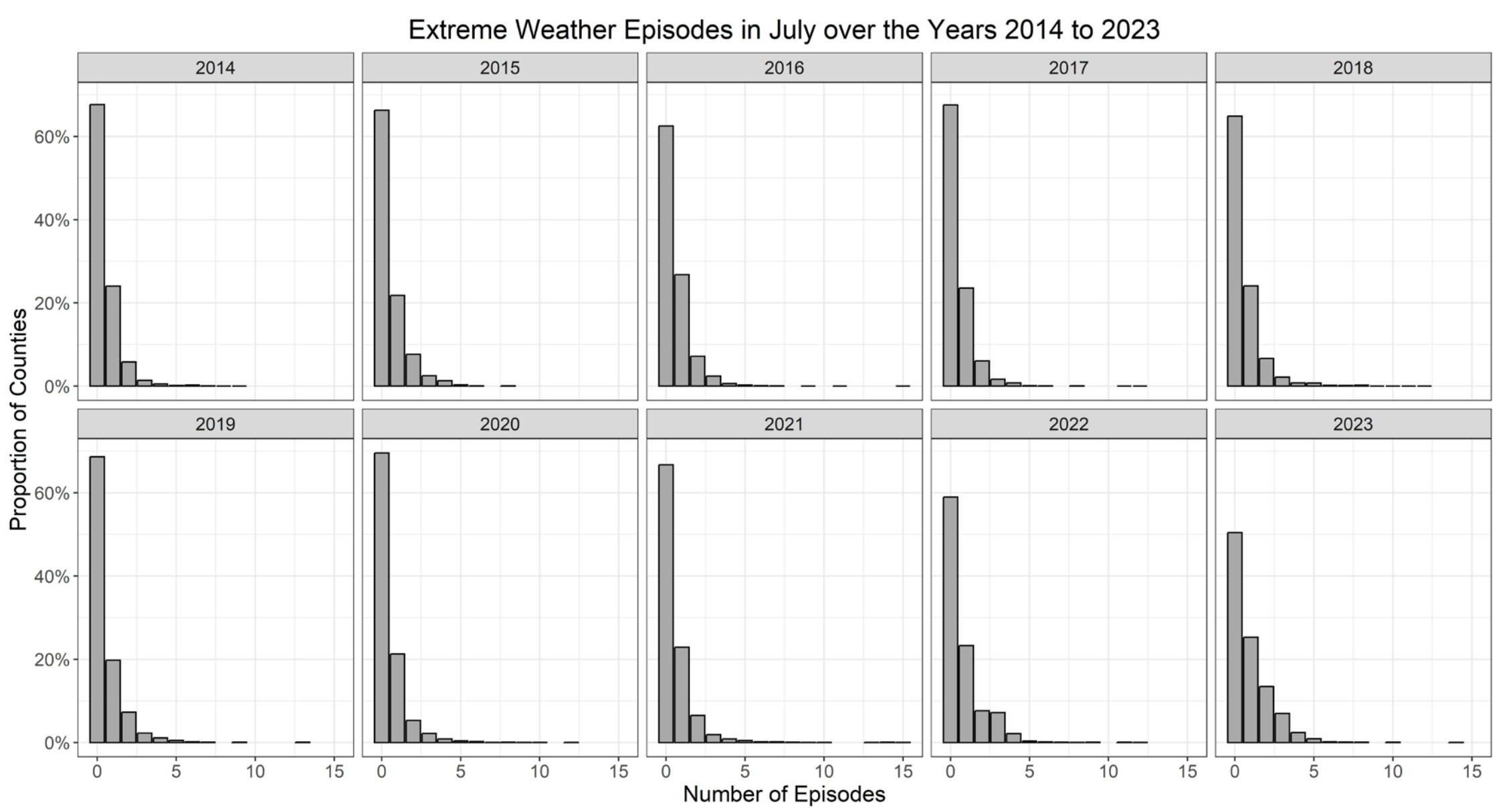

Figure S18: ***Extreme weather episodes in July over the years 2014 to 2023.*** *Histograms displaying the distribution of the raw number of extreme weather episodes in July over the years 2014 to 2023. For each number of episodes on the x-axis, the y-axis shows the proportion of counties that recorded this number of episodes.*

Finally, as reported in the analysis plan and the design table, we plan to run a set of additional analyses regarding hypotheses H_2 and H_3, in which we will test the sensitivity of results to the time period prior to study completion used to assess extreme weather exposure. Regarding H_2, we will estimate the two-way interaction effect of political affiliation and extreme weather exposure on ΔDuration for different time periods from 30 days to 360 days in increments of 30 days. Similarly for H_3, we will estimate the three-way

interaction effect of political affiliation, extreme weather exposure, and attribution of extreme weather events to climate change on ΔDuration for the same time periods. We will visualize results of these additional analyses by plotting the two-way (or three-way) interaction regression coefficients as points surrounded by their 95%-CI on the y-axis and the 12 time periods on the x-axis, as displayed in Figure S19 with simulated data. Based on previous research (Konisky, Hughes, and Kaylor 2016), we expect that the estimated effects will decay as the number of days prior to study completion used to assess the occurrence of extreme weather episodes increases.

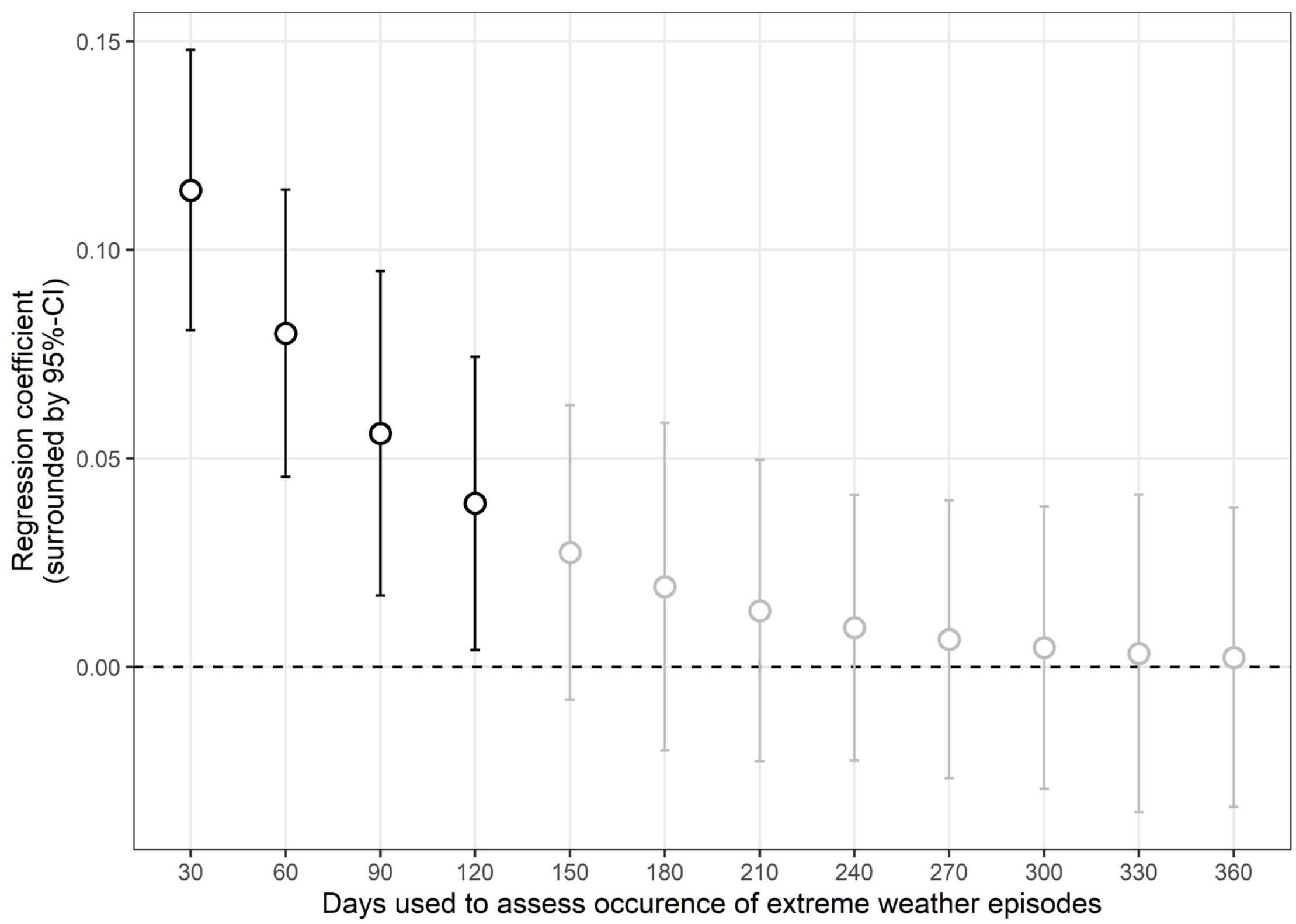

*Figure S19: **Simulation of regression coefficients across different time windows.** Simulated regression coefficients of interaction effects for different number of days prior to study completion used to assess the occurrence of extreme weather episodes. Point estimates are surrounded by their 95% confidence intervals. The dashed line represents the absence of an interaction effect (regression coefficient of zero). Significance of regression coefficients is color-coded, with black indicating regression coefficients significantly different from zero, and grey indicating no significant difference from zero.*

2.5.3.4. Conclusion

Our analyses indicate that July consistently shows a high number of extreme whether episodes with notable geographic variability (Figure S15 and Figure S16). Therefore, to maximize the likelihood of capturing suitable variability in exposure to extreme weather episodes, we plan to conduct our study at the beginning of August, ensuring that the 30-day period prior to study completion falls within July. Moreover, the main source of variability in exposure to extreme weather episodes in July is due to whether at least one episode occurred or not (Figure S17 and Figure S18). Thus, our main analyses will focus on whether a participant was exposed to at least one extreme weather episode in the 30 days prior to

study completion, treated as a binary variable. In additional analyses, we will test the sensitivity of our results to different time periods used to assess extreme weather exposure prior to study completion. We will also conduct these analyses separately for different types of extreme weather events to examine whether different types of extreme weather events may have distinct effects.

2.6. Design Table

Table S26 provides an overview of the research questions, hypotheses, sampling plan, analysis plan, and interpretation as preregistered.

Question	Hypothesis	Sampling Plan	Analysis Plan	Interpretation Given To Different Outcomes
RQ_1: Does political affiliation impact how individuals search for and attend to information during climate-relevant decision-making?<br><br>RQ_{S1}: Does political affiliation impact the choices individuals make in a climate-relevant decision-making context?	H_1: Compared to Republicans, Democrats prioritize searching for and attending to carbon over bonus information during decision-making in the Carbon Emission Task (CET). That is, Democrats display higher ΔDuration values compared to Republicans.<br><br>H_{S1}: Compared to Republicans, Democrats are more likely to make choices that prioritize climate protection over personal financial gain in the CET. That is, Democrats have	Our target sample size is based on a sample-size determination power analysis. We determined the sample size required to detect a smallest effect size of interest (SESOI) of the effect of political affiliation on ΔDuration. We derived this SESOI based on the common practice of filtering out information acquisitions lasting less than 200 msec as such spurious acquisiitons are very unlikely to be consciously processed. Translating this threshold into the metric of ΔDuration results in a SESOI of 0.1143 (see Power Analysis). Sample-size determination	To test H_1, we will run mixed-effects models with the dependent variable ΔDuration (*deltaDuration*) and random intercepts for participants (*subject*, an identifier for each of the N participants) and trials (*trialNum_fixed*, an identifier for each of the 30 CET trials).<br><br>In a first model (M1.1), we will include political affiliation (*polAff*, subject-level binary variable with 0 = Republican and 1 = Democrat) as a fixed effect. To test the sensitivity of this model to our exclusion criteria, we will also run this model with data for all participants.<br><br>In a second model (M1.2), we will test the sensitivity of results of M1.1 to the addition of control variables related to the CET. To this end, we will add to M1.1 fixed effects for: (1) the level of percentage difference in carbon emissions between Options A and B (*carbon_prcnt_level*, trial-level integer variable ranging from 1 [=10%] to 5 [=100%]), (2) the level of percentage difference in bonus payments between Option A and B (*bonus_prcnt_level*, trial-level integer variable ranging from 1 [=10%] to 5 [=100%]), (3) the sequential position a trial was presented at within the sequence of all 30 trials (*trialNum*, trial-level integer variable ranging from 1 to 30), (4) whether the carbon or bonus attributes of Options A and B were presented in the top row of the information matrix (*CB_carbonUp*, subject-level binary variable with 0 = bonus displayed on top and 1 = carbon displayed on top), and (5) whether the option leading to a higher bonus payment and higher carbon emission was presented in the left or in the right column of the information matrix (*CB_selfishLeft*, trial-level binary variable with 0 = higher bonus/carbon option presented in the right column and 1 = higher bonus/carbon	If the fixed effect of *polAff* in M1.1 should not be significantly different from zero at $\alpha = 0.05$ (two-tailed), we will test whether the effect of political affiliation is smaller than the SESOI (unstandardized $\beta = 0.1143$, see Sampling Plan) using equivalence testing. If the narrow confidence interval $(1-2*\alpha)$ surrounding the effect of political affiliation is completely inside the interval [-SESOI; +SESOI], we accept practical equivalence. That is, we will interpret results as suggestive evidence that political affiliation does not impact how individuals search for and attend to information during climate-relevant decision-making.<br><br>If M1.2 or M1.3, compared to M1.1, imply different conclusions regarding the significance of political affiliation, we will interpret results as suggestive evidence that the effect of political affiliation is sensitive to features of the CET trials (M1.2) or demographic variables (M1.3).<br><br>In case of significant effects of control variables in M1.2 or M1.3, we will additionally assess whether these control variables interact with political affiliation. For instance, if *trialNum* turns out to be a

Question	Hypothesis	Sampling Plan	Analysis Plan	Interpretation Given To Different Outcomes
	a higher probability of making pro-climate choices compared to Republicans.	analysis with simulated data suggested that N = 600 would provide > 95% statistical power to detect this SESOI. To ensure broad geographical sampling coverage and to account for smaller statistical power of models investigating interaction effects (H_2 and H_3), we will aim for a target sample size of N = 2,000.	option presented in the left column).<br><br>In a third model (M1.3), we will test the sensitivity of results of M1.1 to the addition of demographic control variables. To this end, we will add to M1.1 fixed effects for: (1) gender (*gender*, subject-level binary variable with 0 = Male and 1 = Female), (2) age (*age*, subject-level integer variable indicating participant's age in years at the time of study completion), (3) education (*education*, subject-level factor with 6 levels: [1] "Did not complete high school / GED", [2] "High school diploma / GED", [3] "Some college", [4] "Associates degree", [5] "Bachelor's degree", [6] "Master's degree or higher"), (4) income (*income*, subject-level factor with 6 levels: [1] "Under $30,000", [2] "$30,000 - $49,999", [3] "$50,000 - $74,999", [4] "$75,000 - $99,999", [5] "$100,000 - $149,999", and [6] "More than $150,000"), (5) race / ethnicity (*racethn_simple*, subject-level factor with 6 levels: [1] "European", [2] "Hispanic", [3] "African", [4] "Asian", [5] "More Than One", and [6] "Other"), and (6) area of residency (*ruralUrb*, subject-level factor with 3 levels: [1] "Urban, a big city", [2] "Suburb, outskirt of a big city", and [3] "Rural town or small city").<br><br>To test H_{S1}, we will run mixed-effects logistic regressions with the actual decision (*choice*, trial-level variable with 0 = pro-self and 1 = pro-climate) as dependent variable and random intercepts for *subject* and *trialNum_fixed*. We will rerun models M1.1 to M1.3, exchanging *deltaDuration* with *choice* as dependent variable.	significant predictor, we will assess an additional mixed-effects model with the dependent variable *deltaDuration*, random intercepts for *subject* and *trialNum_fixed*, and fixed effects for *polAff*, *trialNum*, and *polAff* × *trialNum*. If the interaction term is significant, we will interpret results as suggestive evidence that the effect of political affiliation on information search processes depends on the respective moderating variable. We will then probe the interaction to assess whether and at which values/levels of the moderating variable the effect of political affiliation is significantly different from zero.
RQ_2: Does exposure to extreme weather events interact with political affiliation to shape these information search processes?	H_2: Exposure to extreme weather events amplifies the polarization in information search processes during the CET, increasing the difference in	We conducted effect-size sensitivity power analyses to estimate the smallest two-way interaction effect size for which our target sample size of N = 2,000 will	To test H_2, we will run mixed-effects models with deltaDuration as dependent variable and random intercepts for subject and trialNum_fixed. We will add fixed effects in a stepwise manner.<br><br>In a first model (M2.1), which builds on M1.1 of RQ_1, we will include fixed effects for (1) *polAff* and (2) whether a participant was exposed to at least one extreme weather episode in the 30 days prior to CET completion (*ewe*, subject-level binary variable with 0 = participant was exposed to zero extreme weather	If the fixed two-way interaction effect of *polAff* × *ewe* in M2.2 should not be significantly different from zero at α = 0.05 (two-tailed), we will test whether the interaction effect is smaller than the SESOI (unstandardized β = 0.1143, see Sampling Plan) using equivalence testing. If the narrow confidence interval (1-2*α) surrounding the interaction effect is

Question	Hypothesis	Sampling Plan	Analysis Plan	Interpretation Given To Different Outcomes
RQ_{S2}: Does exposure to extreme weather events interact with political affiliation to shape these climate-relevant choices?	time spent on carbon relative to bonus information between Republicans and Democrats among individuals with (vs. without) such exposure. That is, we predict a positive two-way interaction effect of extreme weather exposure and political affiliation on ΔDuration.<br><br>H_{S2}: Exposure to extreme weather events amplifies the polarization in climate-relevant choices, increasing the difference in climate-protecting choices made by Republicans and Democrats. That is, we predict a positive two-way	provide ≥ 95% statistical power. Analyses based on simulated data suggested that two-way interactions ≥ 0.1619 will be detected with high statistical power, even after accounting for a participant exclusion-rate of 5% (N = 950). We conclude that a target sample size of N = 2000 provides high statistical power to detect a theoretically informative two-way interaction effect between political affiliation and extreme weather exposure.	episodes and 1 = participant was exposed to at least one extreme weather episode). This model provides insights into a potential main effect of extreme weather exposure on *deltaDuration* independent of political affiliation.<br><br>In a second model (M2.2), we will add to M2.1 a fixed effect interaction term *polAff* × *ewe*. In case of a significant two-way interaction, we will probe the interaction by testing the effect of political affiliation among the two levels of exposure to extreme weather events. That is, we will test whether the difference in deltaDuration between Democrats and Republicans is significantly different among participants that were exposed to extreme weather episodes and among participants that were not. To test the sensitivity of this model to our exclusion criteria, we will also run this model with data for all participants.<br><br>In a third model (M2.3), we will test the sensitivity of results of M2.2 to the addition of control variables found to be significant predictors in sensitivity analyses testing H_1. For instance, if *trialNum* and *gender* were significant predictors in the previous sensitivity analyses, these variables will be included as fixed effects in M2.3.<br><br>In a fourth model (M2.4), we will test the sensitivity of results of M2.2 to the addition of control variables related to the exposure to extreme weather episodes. To this end, we will add to M2.2 fixed effects for: (1) the abnormality of the number of extreme weather episodes in participants' county of residence relative to the same 30 days period over the past ten years 64 (*abnormality_final*, subject-level continuous variable), (2) total damage in USD associated with the extreme weather episodes participants were exposed to in the 30 days prior to CET completion (*sum_damage*, subject-level continuous variable), (3) total number of injuries associated with these extreme weather episodes (*sum_injuries*, subject-level integer variable), (4) total number of deaths associated with these extreme weather	completely inside the interval [-SESOI; +SESOI], we accept practical equivalence. That is, we will interpret results as suggestive evidence that there is no interaction effect of political affiliation and whether individuals were exposed to at least one extreme weather episode in the 30 days prior to CET completion or not on information search processes.<br><br>If M2.3 or M2.4, compared to M2.2, imply different conclusions regarding the significance of the two-way interaction, we will interpret results as suggestive evidence that the interaction effect of political affiliation and extreme weather exposure is sensitive to features of the CET trials or demographic variables (M2.3) or variables related to the exposure to extreme weather episodes (M2.4).<br><br>If M2.5 reveals a significant two-way interaction *polAff* × *nEpisodes_durationWeighted*, while M2.2 results in no significant interaction, we will interpret results as suggestive evidence that there is indeed an interaction effect of political affiliation and extreme weather exposure on information search processes, but only if the episodes severity is considered, operationalized by their durations.

Question	Hypothesis	Sampling Plan	Analysis Plan	Interpretation Given To Different Outcomes
	interaction between extreme weather exposure and political affiliation on choice.		episodes (*sum_deaths*, subject-level integer variable), (5) the number of days since the most recent extreme weather episode in each participant's county of residence prior to study completion (*daysSinceMostRecentEpisode*, subject-level continuous variable), (6) the population density in participants' county of residence (*population_density_peopPerSqKilometer*, subject-level continuous variable), (7) county-level median household income estimates (*countyIncome*, continuous variable), and (8) county's Partisan Voting Index (PVI) (*countyPvi*, continuous variable).<br><br>To explore potential contextual heterogeneity, we will conduct additional moderation analyses. We will extend model M2.2 by testing three-way interactions of the form *polAff* × *ewe* × *moderator*, where the moderator is one of the following county-level variables: *population_density_peopPerSqKilometer*, *countyIncome*, *countyPvi*. These analyses are intended to provide context on potential effect heterogeneity across structural conditions. To maintain parsimony in the main models and avoid straining statistical power, these moderation analyses are exploratory.<br><br>In a fifth model (M2.5), we will test the sensitivity of results of M2.2 to an alternative operationalization of exposure to extreme weather episodes. To this end, we will rerun M2, exchanging ewe with a duration-weighted count of extreme weather episodes in the 30 days prior to CET completion (*nEpisodes_durationWeighted*, subject-level continuous variable), which takes the severity of these episodes into account.<br><br>Furthermore, in a set of additional analyses (A2.1), we will test the sensitivity of results of M2.2 to the time period prior to study completion used to assess extreme weather exposure. We will estimate the two-way interaction effect of political affiliation and extreme weather exposure for different time periods from 1 standard month (30 days) to 12 standard months (360 days) in	

Question	Hypothesis	Sampling Plan	Analysis Plan	Interpretation Given To Different Outcomes
			increments of 1 standard month. In these analyses, we will operationalize extreme weather exposure as the raw number of extreme weather episodes participants were exposed to (*ewe_n*, subject-level integer variable). That is, for each of the 12 time periods, we will report the two-way interaction effect of *polAff* × *ewe_n*.<br><br>Finally, in a second set of additional analyses (A2.2) we will refine analyses A2.1 by taking into account the different extreme weather event types under consideration (excessive heat, droughts, wildfires, flash floods, coastal floods, hail, strong wind, and tornados). Specifically, we will rerun analyses of A2.1 separately for the different extreme weather event types. This will provide a nuanced picture of the two-way interaction between political affiliation and extreme weather exposure, accounting for the exposure's recency and involved event types.<br><br>To test H_{S2}, we will run mixed-effects logistic regressions with choice as dependent variable and random intercepts for *subject* and *trialNum_fixed*. We will rerun models M2.1 to M2.5, exchanging *deltaDuration* with *choice* as dependent variable.	
RQ_3: Does the interaction of the exposure to extreme weather events and political affiliation on information search processes depend on individuals' subjective attribution of these events to climate change?	H_3: The amplifying effect of exposure to extreme weather events on the polarization in attention allocation during information search processes between Republicans and Democrats is more	We conducted effect-size sensitivity power analyses to estimate the smallest three-way interaction effect size for which our target sample size of N = 2,000 will provide ≥ 95% statistical power. Analyses based on simulated data suggested that	To test H_3, we will run mixed-effects models with *deltaDuration* as dependent variable and random intercepts for *subject* and *trialNum_fixed*. We will add fixed effects in a stepwise manner.<br><br>All models testing H_3 will include both, participants' political affiliation and subjective attribution of extreme weather events to climate change (*subjAttr*, subject-level continuous variable, ranging from 1 to 5 indicating strong disagreement [1] to [5] strong agreement that extreme weather events can be attributed to climate change, mean centered to ease interpretation of simple effects in models including interaction terms). Previous research suggests that *polAff* and *subjAttr* covary 50,108. Thus, including *polAff* and *subjAttr* in the same statistical model might	If VIF values for *polAff* or *subjAttr* in M3.1 are greater than 10, we will report these VIF values as well as the factor by which the standard error is estimated to be increased. We will proceed with running the remaining models but emphasize that readers should prioritize interpretation of the unbiased regression coefficients and pay less attention to statistical significance, which might be affected by increased standard errors.<br><br>If the fixed three-way interaction effect of *polAff* × *ewe* × *subjAttr* in M3.5 should

Question	Hypothesis	Sampling Plan	Analysis Plan	Interpretation Given To Different Outcomes
RQ_{S3}: Does the interaction of the exposure to extreme weather events and political affiliation on choices depend on individuals' subjective attribution of these events to climate change?	pronounced for individuals who attribute these events more strongly to climate change. That is, we predict a positive three-way interaction effect of political affiliation, exposure to extreme weather events, and their attribution to climate change on ΔDuration.<br><br>H_{S3}: The amplifying effect of exposure to extreme weather events on the polarization in climate-relevant choices between Republicans and Democrats is more pronounced for individuals who attribute these events more strongly to climate change.	three-way interactions ≥ 0.1890 will be detected with high statistical power, even after accounting for a conservative participant exclusion-rate of 5% (N = 950). We conclude that a target sample size of N = 2000 provides high statistical power to detect a theoretically informative three-way interaction effect between political affiliation, extreme weather exposure, and subjective attribution of extreme weather events to climate change.	raise concerns regarding multicollinearity. While multicollinearity does not affect coefficient estimates, it increases standard errors, thereby reducing statistical efficiency. Variance inflation factor (VIF) values are commonly reported to detect multicollinearity in statistical models, with higher values indicating higher multicollinearity. Importantly, however, if interaction terms are included in a model, high VIF values are necessarily expected. This type of multicollinearity between the components of an interaction, known as inessential ill-conditioning, leads to inflated VIF values commonly observed in models with interaction terms. Thus, VIF values are not informative of multicollinearity in models including interaction terms, which is why we will check VIF values only in models without interaction terms (see model M3.1).In a first model (M3.1), which builds on M2.1 of RQ_2, we will include fixed effects for: (1) *polAff*, (2) *ewe*, and (3) *subjAttr*.<br><br>In a second model (M3.2), we will include the two-way interaction terms *polAff* × *subjAttr*.<br><br>In a third model (M3.3), we will include the two-way interaction terms *ewe* × *subjAttr*.<br><br>In a fourth model (M3.4), will add to M3.1 all fixed effect two-way interaction terms: (1) *polAff* × *ewe*, (2) *polAff* × *subjAttr*, and (3) *ewe* × *subjAttr*.<br><br>In a fifth model (M3.5), we will add to M3.2 the fixed effect three-way interaction term *polAff* × *ewe* × *subjAttr*. To test the sensitivity of this model to our exclusion criteria, we will also run this model with data for all participants. In case of a significant three-way interaction, we will probe the interaction by testing the two-way interaction effects of political affiliation and extreme weather exposure at different levels of subjective attribution of extreme weather events to climate change. Specifically, we will assess these two-way interactions for individuals scoring low (mean - 1 SD), average (mean), and high (mean + 1 SD) on	not be significantly different from zero at α = 0.05 (two-tailed), we will test whether the interaction effect is smaller than the SESOI (unstandardized β = 0.1345, see Sampling Plan) using equivalence testing. If the narrow confidence interval (1-2*α) surrounding the interaction effect is completely inside the interval [-SESOI; +SESOI], we accept practical equivalence. That is, we will interpret results as suggestive evidence that the interaction effect of political affiliation and extreme weathere exposure 30 days prior to CET completion on information search processes does not depend on the subjective attribution of extreme weather events to climate change.<br><br>If M3.6 or M3.7, compared to M3.5, imply different conclusions regarding the significance of the three-way interaction, we will interpret results as suggestive evidence that the three-way interaction effect of political affiliation, exposure to extreme weather events, and subjective attribution of such events to climate change is sensitive to features of the CET or demographic variables (M3.6) or variables related to the exposure to extreme weather episodes (M3.7).<br><br>If M3.8 reveals a significant three-way interaction *polAff* × *nEpisodes_durationWeighted* × *subjAttr*, while M3.3 results in no significant three-way interaction, we will interpret results as suggestive evidence

Question	Hypothesis	Sampling Plan	Analysis Plan	Interpretation Given To Different Outcomes
	That is, we predict a positive three-way interaction effect of political affiliation, exposure to extreme weather events, and their attribution to climate change on choice.		subjective attribution of extreme weather events to climate change.<br><br>In a sixth model (M3.6), we will test the sensitivity of results of M3.5 to the addition of control variables found to be significant predictors in sensitivity analyses testing H_1.<br><br>In a seventh model (M3.7), we will test the sensitivity of results of M3.5 to the addition of control variables related to the exposure to extreme weather episodes. To this end, we will add to M3.5 fixed effects for: (1) *abnormality_final*, (2) *sum_damage*, (3) *sum_injuries*, (4) *sum_deaths*, (5) *daysSinceMostRecentEpisode*, (6) *population_density_peopPerSqKilometer*, (7) *countyIncome*, (8) *countyPvi*.<br><br>To explore potential contextual heterogeneity, we will conduct additional exploratory moderation analyses. We will extend model M3.7 by testing four-way interactions of the form *polAff* × *ewe* × *subjAttr* × moderator, where the moderator is one of the following county-level variables: *population_density_peopPerSqKilometer*, *countyIncome*, *countyPvi*.<br><br>In an eighth model (M3.8), we will test the sensitivity of results of M3.5 to an alternative operationalization of exposure to extreme weather episodes. To this end, we will rerun M3.4, exchanging *ewe* with *nEpisodes_durationWeighted*.<br><br>Furthermore, in a set of additional analyses (A3.1), we will test the sensitivity of results of M3.3 to the time period prior to study completion used to assess extreme weather exposure. We will estimate the three-way interaction effect *polAff* × *ewe_n* × *subjAttr* for different time periods from 1 to 12 standard months in increments of 1 standard month.<br><br>Finally, in a second set of additional analyses (A3.2) we will refine	that the moderating role of exposure to extreme weather events on the relationship between political affiliation and information search processes does indeed depend on the subjective attribution of extreme weather events to climate change, but only if the episodes severity is considered, operationalized by their durations.

Question	Hypothesis	Sampling Plan	Analysis Plan	Interpretation Given To Different Outcomes
			analyses A3.1 by taking into account the different extreme weather event types under consideration (excessive heat, droughts, wildfires, flash floods, coastal floods, hail, strong wind, and tornados). Specifically, we will rerun analyses A3.1 separately for the different extreme weather event types. This will provide a nuanced picture of the interaction between political affiliation, extreme weather exposure, and subjective attribution of extreme weather events to climate change, accounting for the recency and event type of exposure.<br><br>To test H_{S3}, we will run mixed-effects logistic regressions with *choice* as dependent variable and random intercepts for *subject* and *trialNum_fixed*. We will rerun models M3.1 to M3.8, exchanging *deltaDuration* with *choice* as dependent variable.	

Table S26: ***Design table*** *Exclusion criteria: We employ several exclusion criteria on participant- and trial-level to ensure high data quality in survey and experimental data. We will screen-out respondents who (1) fail the CAPTCHA or (2) do not give informed consent or (3) fail any of the attention checks described in the Methods Section or (4) do not finish the study. Such screen-outs are not included in the targeted sample size. Additionally, we apply exclusion criteria based on process-tracing metrics in the CET on trial- and participant-level. We exclude trials and participants with exceptionally short or long box opening times as identified by an aggregate function of five different outlier detection algorithms (see Methods Section for details). Finally, we will exclude participants who report having conducted the CET on a mobile phone.*

2.7. Power Analyses

2.7.1. Purpose & Rationale

The primary goal of the present analyses is to determine the number of participants required to achieve 95% statistical power to detect a Smallest Effect Size Of Interest (SESOI) for a main effect of political affiliation (Democrat vs. Republican) on attentional information search behavior as assessed by ΔDuration. That is, we primarily aim for conducting a power analysis for *sample-size determination,* also called *a priori* power analysis (Giner-Sorolla et al. 2024). To this end, we use power simulations with parameters informed by previous studies to assess the statistical power for different sample sizes. This will allow us to decide on a sample size that will provide high statistical power to detect a true and theoretically relevant main effect of political affiliation.

Given this sample size, our secondary goal is to then assess the statistical power to detect different effect sizes for (1) the two-way interaction between political affiliation and extreme weather exposure and (2) the three-way interaction between political affiliation, extreme weather exposure, and the subjective attribution of extreme weather events to climate change. That is, we conduct power analyses for assessing *effect-size sensitivity* for these two- and three-way interactions (Giner-Sorolla et al. 2024).

The present report is organized as follows:

1. We describe all important variables of the planned study and how we will assess them.
2. We derive a SESOI for the main effect of political affiliation on ΔDuration.
3. We conduct power simulations for *sample-size determination analysis* based on this political affiliation main effect SESOI.
4. We conduct power simulations for *effect-size sensitivity analyses* for a two-way interaction effect between political affiliation and extreme weather exposure.
5. We conduct power simulations for *effect-size sensitivity analyses* for a three-way interaction effect between political affiliation, extreme weather exposure, and subjective attribution of extreme weather events to climate change.

2.7.2. Important Variables

2.7.2.1. ΔDuration

Participants will complete 25 trials in a new variant of the Carbon Emission Task (CET, Berger and Wyss 2021) optimized for online process-tracing using MouselabWEB (Willemsen and Johnson 2019). An example trial of the task is displayed and explained in Figure S20.

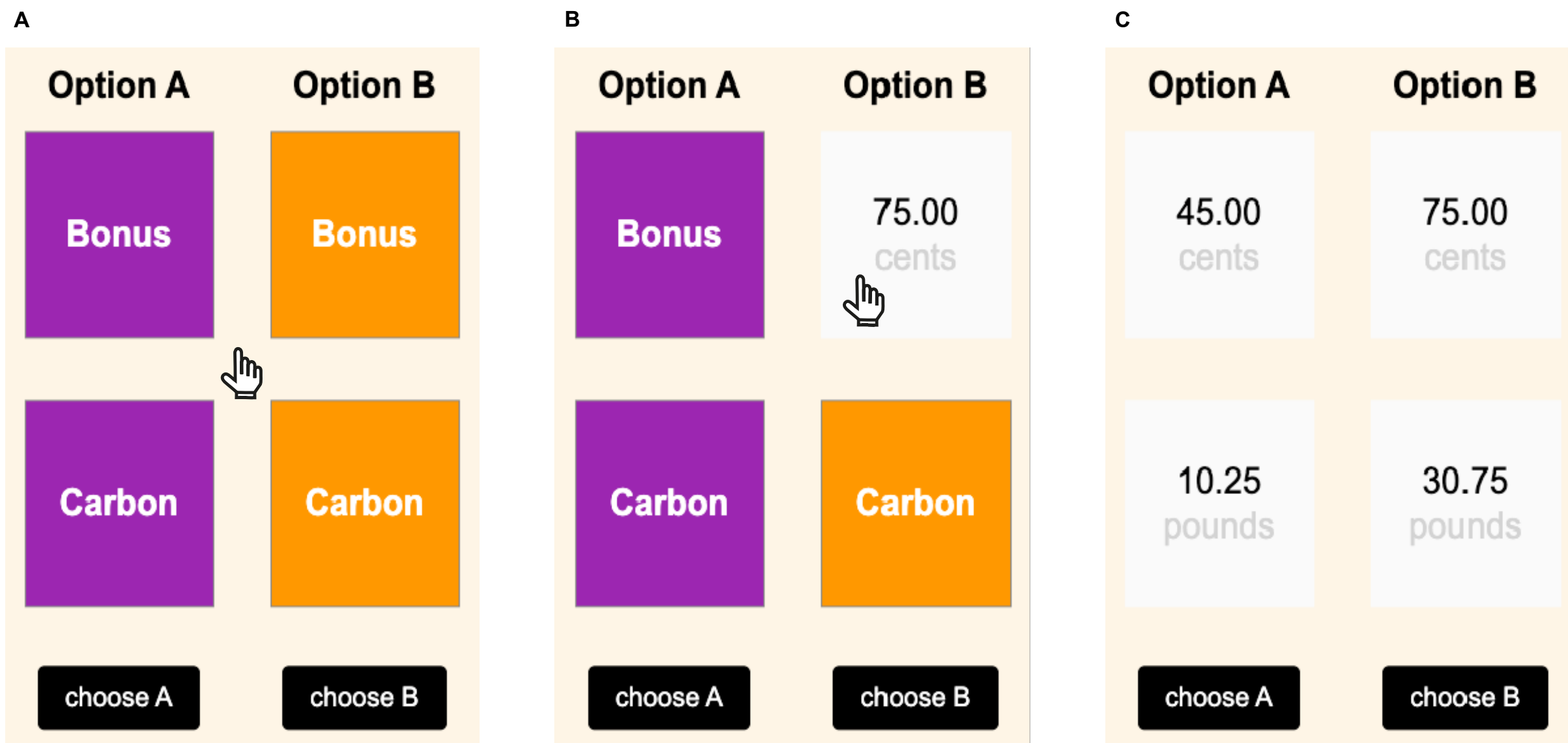

Figure S20: ***An example trial of the new variant of the CET. (A)*** *Participants are presented with two options which are associated with different bonus payment and carbon emission consequences.* ***(B)*** *Participants inspect the exact consequences regarding each attribute of each option by hovering their mouse over the respective boxes. Moving the mouse outside of a box occludes the information again. Note that whether the bonus or carbon attributes are displayed in the top row is randomized across participants but held constant within participants.* ***(C)*** *By visiting all boxes, participants can acquire all available information in a trial (all boxes opened simultaneously for demonstration purposes only). Note that whether the option maximizing the bonus payment for participants is presented in the left or right row is randomized within participants.*

In all analyses, the criterion (dependent variable) will be ΔDuration (`deltaDuration`), which is calculated in each trial as:

$$\Delta Duration = \frac{(t_{Carbon_A} + t_{Carbon_B}) - (t_{Bonus_A} + t_{Bonus_B})}{t_{Carbon_A} + t_{Carbon_B} + t_{Bonus_A} + t_{Bonus_B}}$$

with t representing the summed up dwell time on $Carbon/Bonus$ boxes in Option A/B. ΔDuration varies between -1 and 1 with:

- a value of -1 indicating that the entire dwell time was spent on gathering Bonus information
- a value of 0 indicating that the dwell time was equally split between gathering Bonus and Carbon information
- a value of 1 indication that the entire dwell time was spent on gathering Carbon information

2.7.2.2. Political Affiliation

We will assess political affiliation using the following question:

Generally speaking, do you think of yourself as a Democrat, Republican or Independent?

Participants will answer on the following 7-point Likert scale:

[1] Strong Republican, [2] Not strong Republican, [3] Independent, close to Republican, [4] Independent, [5] Independent, close to Democrat, [6] Not strong Democrat, [7] Strong Democrat

Additionally, if participants self-identify as [4] Independent, they will be asked:

You said that you think of yourself as an Independent politically. If you had to identify with one party of the two parties, which one would you choose?

Participants will answer on the following scale:

[1] Republican Party, [2] Democratic Party

Based on these questions, we classify participants as either Republican or Democrat as represented by the variable `polAff` that can take on the following values:

[rep] Republican Party, [dem] Democratic Party

2.7.2.3. Extreme Weather Exposure

For each participant, we will assess the number of extreme weather episodes that occurred in the participants' county of residence in the 30 days prior to study completion. We then create a variable `ewe` which equals to *TRUE* if at least one extreme weather episode occurred in the specified time interval, and *FALSE* otherwise.

2.7.2.4. Subjective Attribution

We will assess the degree to which participants attribute extreme weather events to climate change (see Ogunbode et al. 2019). Participants will rate their agreement to the following three questions:

1. *Extreme weather events are caused in part by climate change.*
2. *Extreme weather events are a sign that the impacts of climate change are happening now.*
3. *Extreme weather events show us what we can expect from climate change in the future.*

Participants will answer on the following 5-point Likert scale:

[1] Strongly disagree, [2] Somewhat disagree, [3] Neither agree nor disagree, [4] Somewhat agree, [5] Strongly agree

Subjective attribution of EWE to climate change will be operationalised as the mean agreement to these three statements. Note that Ogunbode et al. (2019), who used the same questions, response options, and aggregation, report a mean of 3.67 and a SD of 0.85 for this variable.

2.7.3. SESOI

As argued in the Registered Report, we hypothesize that (H_1) compared to Republicans, Democrats prioritize searching for and attending to carbon over bonus information during decision-making in the CET. That is, Democrats display higher ΔDuration values compared to Republicans.

While H_1 describes the direction of the expected effect, it does not specify the expected magnitude of the effect. This later point is clarified by asking the question what would be the smallest effect size that researchers would still consider theoretically relevant, i.e., what is the smallest effect size of interest (SESOI)? We argue for such a SESOI on theoretical grounds and based on previous MouselabWEB research.

In mouselabWEB studies, it is standard practice to filter out any information acquisition lasting less than 200 msec because such very short (spurious) acquisitions are very unlikely to be consciously processed (Willemsen and Johnson 2019). Therefore, we derive the SESOI based on the consideration that for an effect to be meaningful, the increase in ΔDuration from a Republican to a Democrat should be due to an increase of the time spent gathering carbon (relative to bonus) information of at least 200 msec. We need to translate these considerations into the metric of ΔDuration.

Suppose that Republicans on average spend t_{Carbon} msec on gathering carbon information and t_{Bonus} msec on gathering bonus information in each trial, with a total time of gathering any information $t_{Total} = t_{Carbon} + t_{Bonus}$. Thus, for Republicans, this results in:

$$\Delta Duration_{Rep} = \frac{t_{Carbon} - t_{Bonus}}{t_{Total}}$$

Suppose that Democrats have the same t_{Total} as Republicans, but they spend 200 msec more on gathering carbon information and, correspondingly, 200 msec less on gathering bonus information. Thus, for Democrats, this results in:

$$\Delta Duration_{Dem} = \frac{(t_{Carbon} + 200) - (t_{Bonus} - 200)}{t_{Total}} = \frac{400 + t_{Carbon} - t_{Bonus}}{t_{Total}}$$

Therefore, the difference in $\Delta Duration$ for Democrats and Republicans is:

$$\begin{aligned} \Delta Duration_{Dem} - \Delta Duration_{Rep} &= \frac{400 + t_{Carbon} - t_{Bonus}}{t_{Total}} - \frac{t_{Carbon} - t_{Bonus}}{t_{Total}} \\ &= \frac{(400 + t_{Carbon} - t_{Bonus}) - (t_{Carbon} - t_{Bonus})}{t_{Total}} \\ &= \frac{400}{t_{Total}} \end{aligned}$$

Thus, we define our SESOI as:

$$SESOI_{polAff} = \frac{400\ msec}{t_{Total}}$$

As this definition shows, the SESOI depends on our expectation regarding t_{Total}. We form these expectations based on previous research. In our lab, we recently conducted another MouselabWEB study whose setup was very similar to the one described in the Registered Report (Studler et al., in preparation). In short, student participants completed a behavioral task in which they searched for and attended to information presented in a 2-by-2 decision matrix adapted from Reeck, Wall, and Johnson (2017). The average time participants spent on acquiring information in each trial (t_{Total}) was 3.3 seconds. In the planned study we will assess a representative US sample. That is, the average age of our sample will be higher than in a typical student sample. As processing speed is known to decline with age (Salthouse 2000), we assume a slightly higher total information acquisition time in our sample of $t_{Total} = 3500\ msec$. Therefore, we define our SESOI as:

$$SESOI_{polAff} = \frac{400\ msec}{3500\ msec} = 0.1143$$

To account for uncertainty in this estimation, we also provide sample-size determination analysis results based on $t_{Total} = 4000\ msec$ and $t_{Total} = 3000\ msec$. This results in a high and low estimate of SESOI:

$$SESOI_{polAff_{high}} = \frac{400\ msec}{3000\ msec} = 0.1333$$
$$SESOI_{polAff_{low}} = \frac{400\ msec}{4000\ msec} = 0.1000$$

2.7.4. Main Effect

As argued in the Registered Report, we hypothesize:

(H_1) *Compared to Republicans, Democrats prioritize searching for and attending to carbon over bonus information during decision-making in the Carbon Emission Task. That is, Democrats display higher ΔDuration values compared to Republicans.*

```
# Create a data frame with predicted true effects

# Smallest Effect Size Of Interest (SESOI)
SESOI <- 0.4/3.5

# Betas
beta_p <- SESOI

# Predicted "true" effects
polAff_trueEffects <- expand_grid(
  polAff = factor(c("rep", "dem"), levels = c("rep", "dem"))
) %>%
  add_contrast("polAff", contrast = "anova", colnames = "X_p") %>%
  mutate(
    trueDeltaDuration =
      0 +          # intercept
      X_p * beta_p # main effect polAff
  )
```

2.7.4.1. Data Simulation Function

We first define a function that simulates data for the main effect of political affiliation on ΔDuration: `FUN_sim`. The function will simulate data according to the following model, expressed in lme4-lingo:

```
deltaDuration ~ polAff + (1|subj) + (1|trial)
```

The function `FUN_sim` takes, among others, the following important arguments:

- `n_subj`: **Number of subjects**. Chaning this parameter allows us to assess statistical power for different sample sizes.

- `beta_0`: **Fixed intercept (grand mean)**. We assume that the average participant (irrespective of political affiliation or any other predictor variable) spends about the same time on searching for and attending to carbon as to bonus information in the CET. Therefore, we set `beta_0` to zero. The effects of predictor variables will be modeled as deviations from this grand mean.

- `beta_p`: **Fixed effect of political affiliation**. This value represents the average difference in ΔDuration between Democrats and Republicans (Democrats - Republicans). As discussed above, we set this value to 0.1143 by default, and we assess the effect of changing this variable on statistical power.

- `subj_0`: **By-subject random intercept SD**. We simulate that a participants' deviations from the grand mean for ΔDuration follows a normal distribution with a mean of 0 and a standard deviation of `subj_0` = 0.29. We base our default value on a study by Reeck, Wall, and Johnson (2017). They investigated whether variability in information search behavior is driven predominantly by differences in the features of a choice (i.e., in our case: the relative differences between carbon and bonus outcomes in options A and B) or by individual differences. To this end, they predicted information acquisition using a intercept-only model that included random intercepts for subjects and items. They estimated the random intercept of subjects to be 0.29.

- `trial_0`: **By-trial random intercept SD**. We simulate that a items' deviations from the grand mean for ΔDuration follows a normal distribution with a mean of 0 and a standard deviation of `trial_0` = 0.04. Again, we base our default value on the study by Reeck, Wall, and Johnson (2017), who estimated a random intercept for trials of 0.04.

- `sigma`: **Trial-level noise (error) SD**. We model the error SD to be of the same size as the sum of the random intercept SDs = 0.29 + 0.04 = 0.33. We also report simulation results for an error SD that is twice the size of the random intercept SDs, i.e., 0.66.

The function `FUN_sim` is defined below (code visible in html output only):

We call the function once and extract the results of this single simulation (code visible in html output only):

Figure S21 visualizes the results of this single simulation and Table S27 summarises the statistical results of fitting the actual model used in data generation to the simulated data.

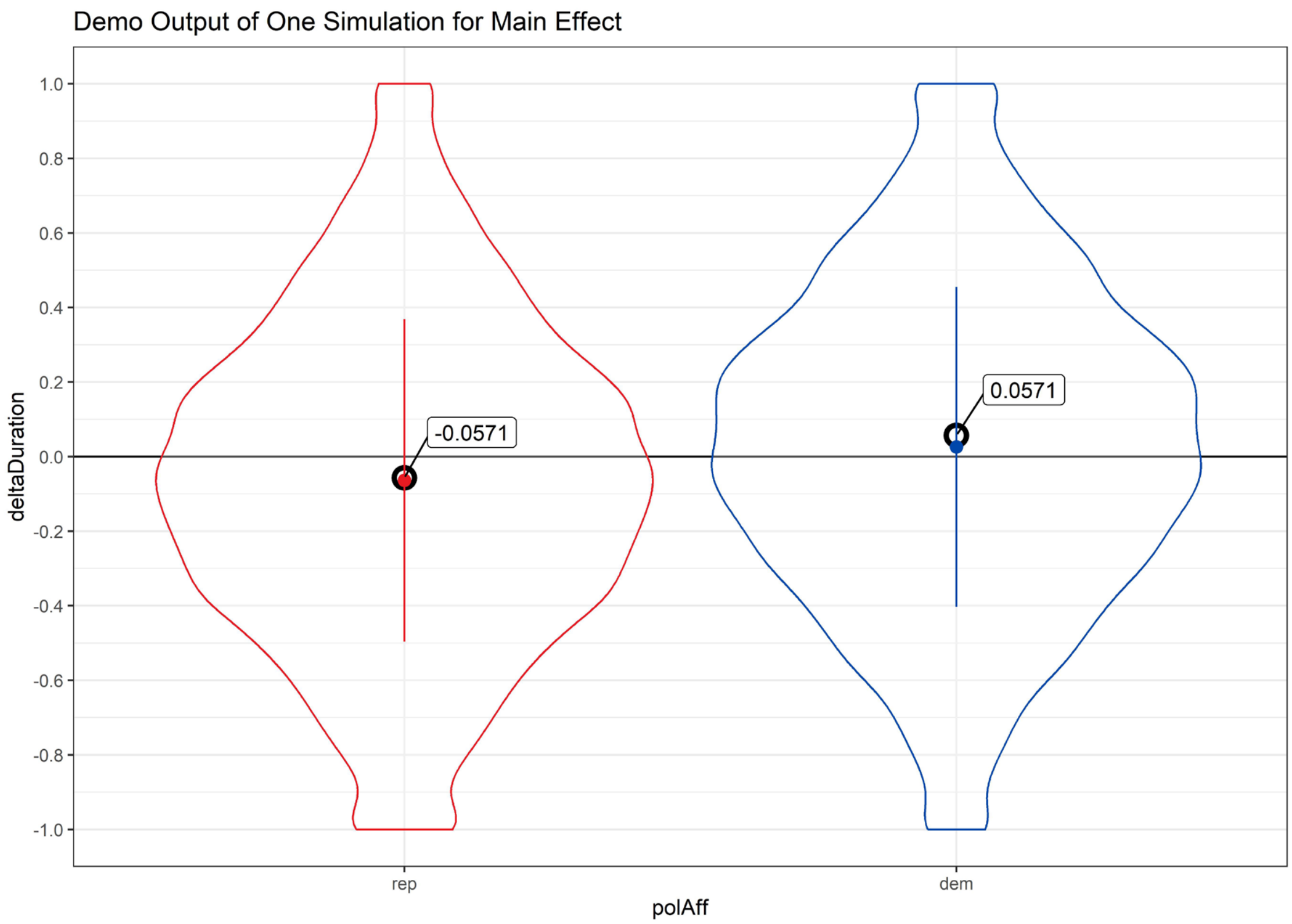

Figure S21: ***Demo output of one simulation for main effect.*** *Visual representation of results of one simulation created using FUN_sim. Violin plots display the full distribution of the data (red = Republicans; blue = Democrats). Points and surrounding lines indicate the mean ± 1 SD. The black horizontal line displays the true sample mean and the black open circles indicate the true means for each cell.*

effect	group	term	estimate	p.value
fixed	NA	(Intercept)	-0.0188	0.1105
fixed	NA	polAffX_p	0.0896	0.0000
ran_pars	subj	sd__(Intercept)	0.2841	NA
ran_pars	trial	sd__(Intercept)	0.0363	NA
ran_pars	Residual	sd__Observation	0.3223	NA

Table S27: ***Statistical results of one simulation.*** *Data were simulated using FUN_sim and fitted using deltaDuration ~ polAff + (1 | subj) + (1 | trial).*

2.7.4.2. Power Simulation

We now simulate multiple random samples drawn from the same synthetic population with a known true effect of political affiliation. For each random sample, we fit our statistical model to the data. The statistical power to detect a true effect of political affiliation is calculated as the proportion of significant effects out of the total number of simulations. We aim for a statistical power of 95%.

In the following code, the simulations are calculated. We do not recommend executing this code junk as it takes several hours to run (code only shown in the html version).

We retrieve pre-run results:

```
# Load power simulation data
resList_mainEffect <- readRDS(file.path("../powerSimulationsOutput", "res_mainEffect_
20240813_2141.RDS"))
resList_mainEffect.summary <- resList_mainEffect$res_mainEffect.summary

# Extract power values for some specific effect sizes at N = 1000
chosenN <- 600
chosenSigma <- c("0.3300", "0.6600")
chosenSESOI <- c("0.1000", "0.1143")
powerValues <- resList_mainEffect.summary %>%
  filter(sigma_fact %in% chosenSigma) %>%
  filter(sesoi_fact %in% chosenSESOI) %>%
  filter(nSubjects %in% chosenN) %>%
  mutate(power_str = paste0(round(ci.lower*100, 2), "%")) %>%
  pull(power_str)

# Extract number of simulations
label_nSimulations <- resList_mainEffect$res_mainEffect$sim %>% n_distinct()
```

Figure S22 displays the distribution of estimated fixed effects across all simulations. The figure shows that the estimated fixed effects are close to the true ones provided as input in the data simulation function, validating that simulations worked as expected.

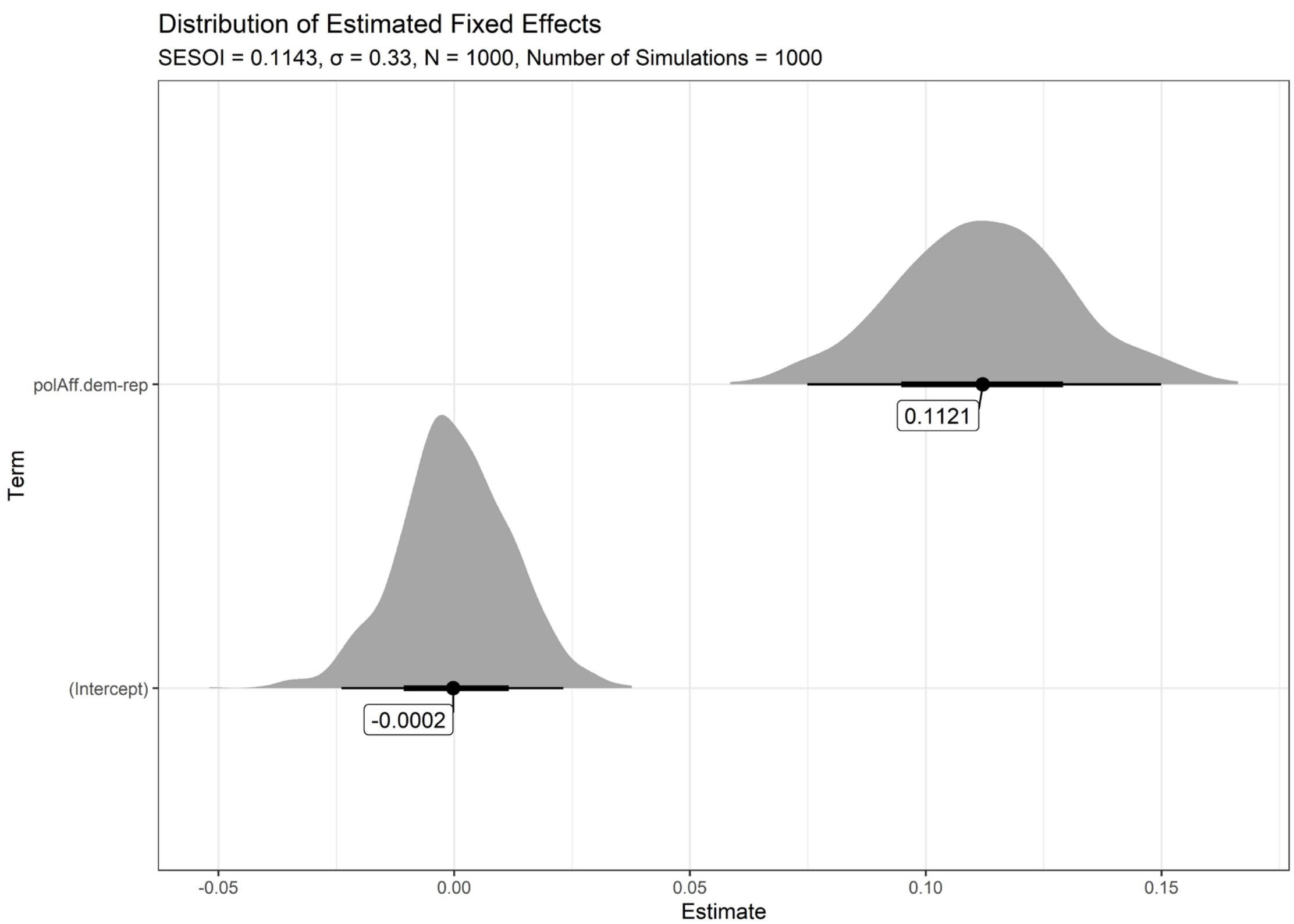

Figure S22: ***Distribution of estimated fixed effects.*** *The figure shows the estimated fixed effects obtained from 1000 simulations for the model deltaDuration ~ polAff + (1 | subj) + (1 | trial). Shaded area represent densities, annotated points indicate medians, and thick and thin lines represent 66% and 95% quantiles, respectively.*

Figure S23 shows results of our sample-size determination analyses. We find that a sample size of 600 provides a statistical power of 95.4% (lower bound of 95%-CI) even under the most conservative assumptions (SESOI = 0.1000, Error SD = 0.6600).

Moreover, we inspect the lower bounds of the 95%-CI power estimates in Figure S23. Specifically, we focus on the power simulation results for a conservative SESOI of 0.1000 and an error SD of 0.6600. Then, we use interpolation between sample sizes of 800 and 1000 to estimate the number of participants needed to achieve 99% statistical power (lower bounds of 95%-CI power estimates). Results reveal that with a sample size of N = 895, we would achieve 99% statistical power (lower bound of 95%-CI power estimate) to detect a true effect of at least 0.1000.

We optimize the study design to detect a true SESOI for political affiliation. However, we are also interested in two-way and three-way interaction effects, which are known to require greater sample sizes to achieve the same statistical power as for main effects. Moreover, greater sample sizes are more likely to accurately represent target populations with respect to variables like exposure to extreme weather events and subjective attribution of extreme weather events to climate change. Therefore, we opt for a sample size of N = 1000 for further effect-size sensitivity analyses regarding interaction effects.

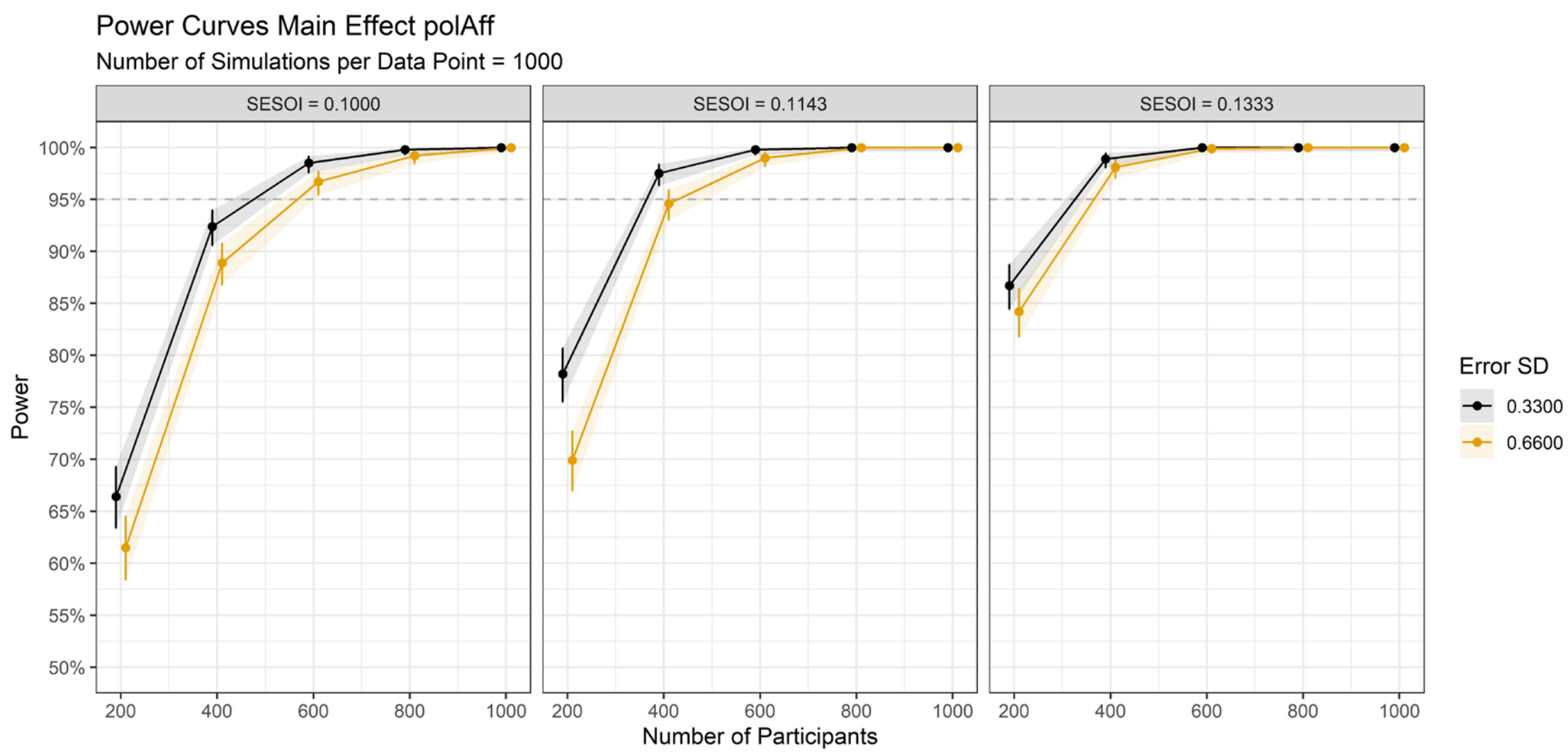

Figure S23: ***Power curves for the main effect of political affiliation.*** *Points represent statistical power surrounded by a 95%-CI based on 1000 simulations with α = 0.05.*

2.7.5. Two-Way Interaction Effect

As argued in the Registered Report, we hypothesize:

(H_2): *Exposure to extreme weather events amplifies the polarization in attentional information search processes, increasing the difference in ΔDuration between Republicans and Democrats among individuals with (vs. without) such exposure. In other words, there is a positive two-way interaction effect of extreme weather exposure and political affiliation on ΔDuration.*

```
# Create a data frame with predicted true effects

# Smallest Effect Size Of Interest (SESOI)
SESOI <- 0.4/3.5

# Betas
beta_p <- SESOI
beta_e <- 0
beta_p_e_inx <- SESOI

# Predicted "true" effects
polAff_ewe_trueEffects <- expand_grid(
  polAff = factor(c("rep", "dem"), levels = c("rep", "dem")),
  ewe = factor(c(FALSE, TRUE), levels = c(FALSE, TRUE))
) %>%
  add_contrast("polAff", contrast = "anova", colnames = "X_p") %>%
  add_contrast("ewe", contrast = "anova", colnames = "X_e") %>%
  mutate(
    trueDeltaDuration =
      0 +                         # intercept
      X_p * beta_p +              # main effect polAff
      X_e * beta_e +              # main effect ewe
      X_p * X_e * beta_p_e_inx    # interaction effect polAff*ewe
  )
```

2.7.5.1. SESOI for Two-Way Interaction

For deriving a SESOI for the two-way interaction of interest, similar considerations apply as in the case of the SESOI for the main effect of interest. In this section, we make these considerations explicit. We start by noticing that the complete fixed two-way interaction polAff × ewe is modeled as:

$$\Delta Duration = \beta_0 + \beta_1 \cdot polAff + \beta_2 \cdot ewe + \beta_3 \cdot (polAff \times ewe)$$

By rearranging terms, one can show that the effect of polAff is given by:

$$Effect_{polAff} = \beta_1 + \beta_3 \cdot ewe$$

Now, let's calculate this effect for two individuals who differ in their levels of ewe. As ewe is a binary variable, we have two types of individuals: individuals with (ewe = 1) and without (ewe = 0) extreme weather exposure. For an individual without extreme weather exposure, the effect of polAff will be:

$$Effect_{noEWE} = \beta_1 + \beta_3 \cdot 0 = \beta_1$$

For an individual with extreme weather exposure, the effect of polAff will be:

$$Effect_{EWE} = \beta_1 + \beta_3 \cdot 1 = \beta_1 + \beta_3$$

The difference in the effect of polAff between these two individuals is given by:

$$Effect_{EWE} - Effect_{noEWE} = (\beta_1 + \beta_3) - \beta_1 = \beta_3$$

We consider this difference as theoretically relevant if it is at least of the same size as the SESOI for the effect of polAff:

$$SESOI_{polAff \times ewe} = Effect_{EWE} - Effect_{noEWE} = SESOI_{polAff} = 0.1143$$

2.7.5.2. Data Simulation Function

We next define a function that simulates data for the two-way interaction effect of political affiliation with extreme weather exposure on ΔDuration: `FUN_sim_2wayInt`. The function will simulate data according to the following model, expressed in lme4-lingo:

```
deltaDuration ~ polAff * ewe + (1|subj) + (1|trial)
```

The function `FUN_sim_2wayInt` takes, among others, the following important arguments (in addition to the arguments discussed for `FUN_sim`):

- `beta_p`: **Fixed main effect of political affiliation**. Compatible with H_1, we keep this value at the SESOI of 0.1143. That is, we model that the average effect of political affiliation across all participants, irrespective of their extreme weather exposure, is 0.1143.

- `beta_e`: **Fixed main effect of extreme weather exposure**. As we are interested in the moderating role of extreme weather exposure, we set this main effect to zero.

That is, we assume that the effect of extreme weather exposure is highly dependent on participants' political affiliation.

- `beta_p_e_inx`: **Fixed two-way interaction effect of political affiliation and extreme weather exposure**. We set this initial value to the SESOI derived above, but we investigate how changing this effect size impacts statistical power, as we are conducting effect-size sensitivity analyses for interaction effects.

The function `FUN_sim_2wayInt` is defined below:

We call the function once and extract the results of this single simulation:

Figure S24 visualizes the results of this single simulation and Table S28 summarizes the statistical results of fitting the actual model used in data generation to the simulated data.

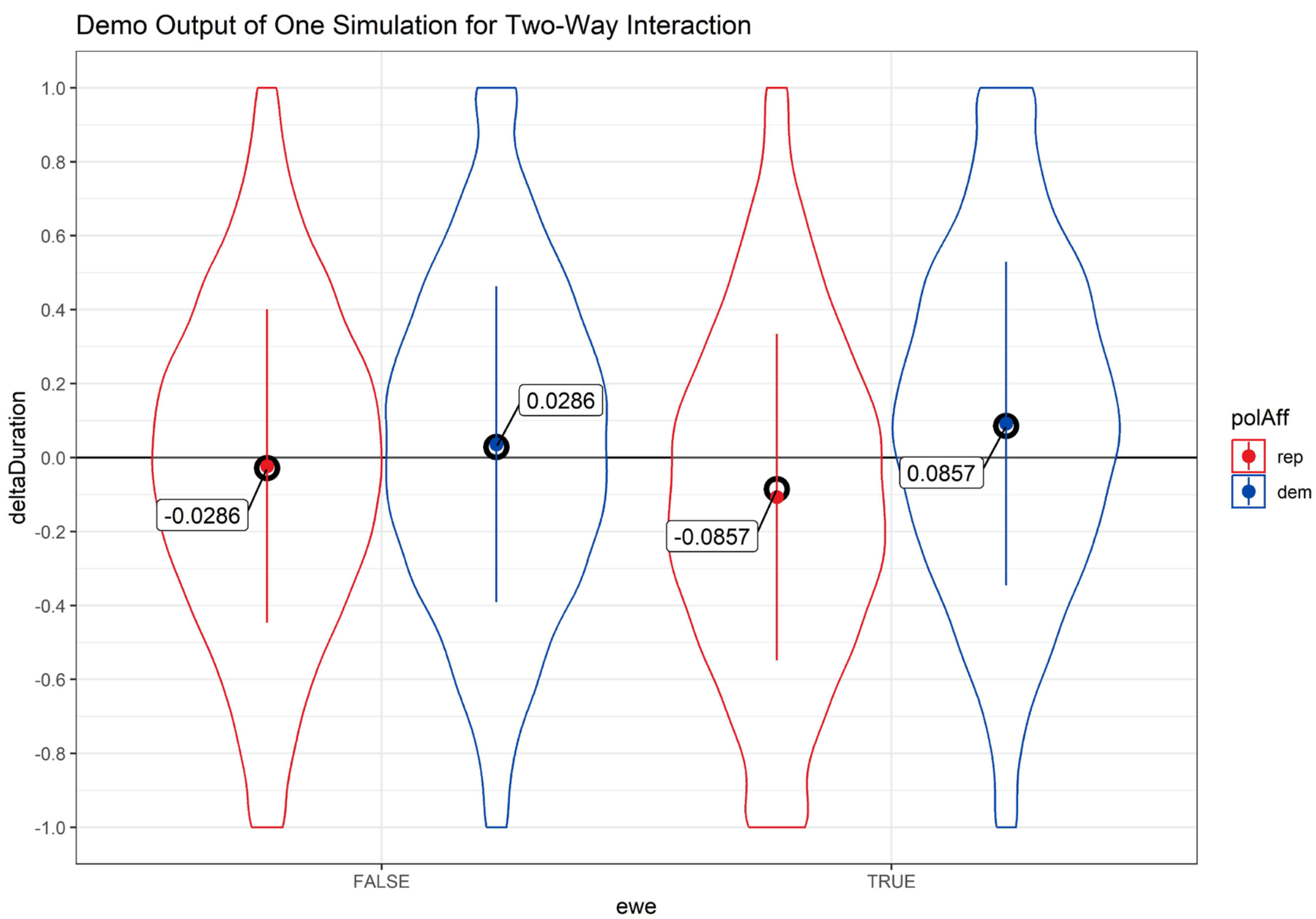

Figure S24: ***Demo output of one simulation for two-way interaction.*** *Visual representation of results of one simulation created using* `FUN_sim_2wayInt`*. Violin plots display the full distribution of the data (red = Republicans; blue = Democrats). Points and surrounding lines indicate the mean ± 1 SD. The black horizontal line displays the true sample mean and the black open circles indicate the true means for each cell.*

effect	group	term	estimate	p.value
fixed	NA	(Intercept)	-0.0006	0.9625
fixed	NA	polAffX_p	0.1290	0.0000
fixed	NA	eweX_e	-0.0139	0.4516
fixed	NA	polAffX_p:eweX_e	0.1398	0.0002
ran_pars	subj	sd__(Intercept)	0.2855	NA
ran_pars	trial	sd__(Intercept)	0.0382	NA
ran_pars	Residual	sd__Observation	0.3232	NA

*Table S28: **Statistical results of one simulation.** Data were simulated using FUN_sim_2wayInt and fitted using deltaDuration ~ polAff * ewe + (1 | subj) + (1 | trial).*

2.7.5.3. Power Simulation

In the following code, the simulations are calculated. We do not recommend executing this code junk as it takes several hours to run.

We retrieve pre-run results:

```
# Load power simulation data
resList_2wayInt <- readRDS(file.path("../powerSimulationsOutput", "res_2wayInt_20240814_1052.RDS"))
resList_2wayInt.summary <- resList_2wayInt$res_2wayInt.summary

# Extract power values for some specific assumptions
chosenN <- 950
chosenSigma <- c("0.3300", "0.6600")
chosenSESOI <- c("0.1429", "0.1714")
powerValues <- resList_2wayInt.summary %>%
  filter(sigma_fact %in% chosenSigma) %>%
  filter(sesoi_fact %in% chosenSESOI) %>%
  filter(nSubjects %in% chosenN) %>%
  mutate(power_str = paste0(round(power*100, 2), "%")) %>%
  pull(power_str)

# Extract number of simulations
label_nSimulations <- resList_2wayInt$res_2wayInt$sim %>% n_distinct()

# Repeat breaks_sesoi
breaks_sesoi <- (0.4/3.5)*seq(1, 2, .25)
```

Figure S25 displays the distribution of estimated fixed effects across all simulations. The figure shows that the estimated fixed effects are close to the true ones provided as input in the data simulation function, validating that simulations worked as expected.

```r
# Define some filters
filter_nSubjects <- 1000
filter_sesoi <- unique(resList_2wayInt$res_2wayInt$sesoi)[1]
filter_sigma <- unique(resList_2wayInt$res_2wayInt$sigma)[1]

# Prepare data for plot
fixedEstimates <- resList_2wayInt$res_2wayInt %>%
  mutate(
    group = ifelse(is.na(group), "", group),
    group_term = str_remove(str_c(group, term, sep = "_"), "^_")
  ) %>%
  filter(effect == "fixed") %>%
  filter(nSubjects == filter_nSubjects) %>%
  filter(sesoi == filter_sesoi) %>%
  filter(sigma == filter_sigma)
fixedEstimates_medians <- fixedEstimates %>%
  group_by(group_term) %>%
  summarise(
    median = median(estimate, na.rm = TRUE),
    median_rounded = format(round(median, 4), nsmall = 4, scientific = FALSE),
    .groups = 'drop'
  )

# Create plot
p.checkSims.2wayInt <-fixedEstimates %>%
  ggplot(aes(x = estimate, y = group_term)) +
  ggdist::stat_halfeye() +
  ggrepel::geom_label_repel(
    data = fixedEstimates_medians,
    mapping = aes(x = median, y = group_term, label = median_rounded),
    box.padding = .5
  ) +
  labs(
    title = "Distribution of Estimated Fixed Effects",
    subtitle = paste0(
      "SESOI = ", round(filter_sesoi, 4), ", ",
      "σ = ", round(filter_sigma, 4), ", ",
      "N = ", filter_nSubjects, ", ",
      "Number of Simulations = ", label_nSimulations
    ),
    x = "Estimate",
    y = "Term"
  ) +
  theme_bw()

jpeg(
  file = "../images/pa_checkSims2wayInt.jpeg",
  width = 9.1, height = 6.5, units = "in", res = 600
)
print(p.checkSims.2wayInt)
invisible(dev.off())
```

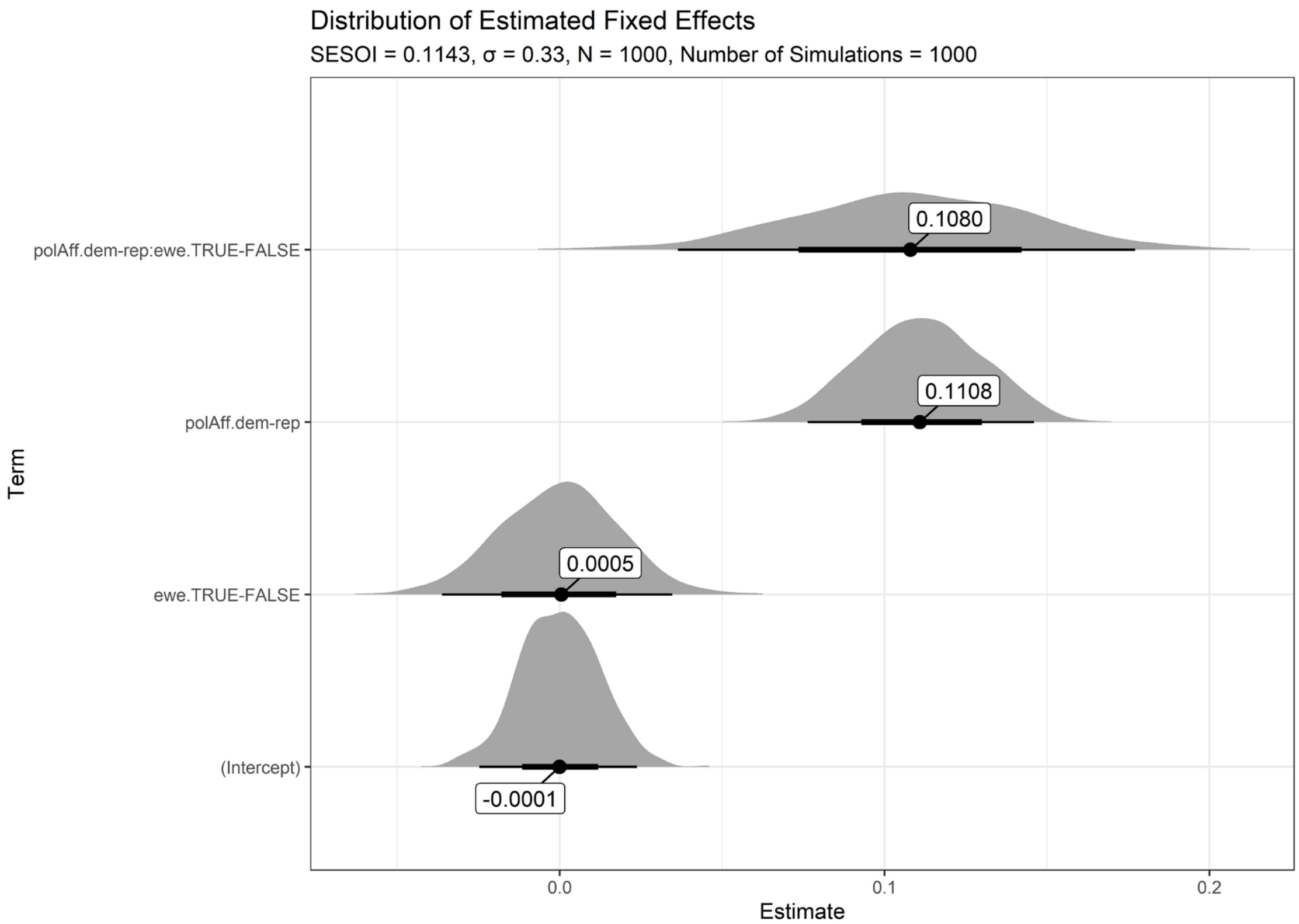

Figure S25: ***Distribution of estimated fixed effects.*** *The figure shows the estimated fixed effects obtained from 1000 simulations for the model deltaDuration ~ polAff * ewe + (1 | subj) + (1 | trial). Shaded area represent densities, annotated points indicate medians, and thick and thin lines represent 66% and 95% quantiles.*

Figure S26 shows results of our effect-size sensitivity analyses. We plot statistical power (y-axis) for different effect sizes (x-axis), taking into account different assumptions for the error SD (color) and sample size (panel). Regarding the latter, we report results not only for the full sample size we aim for (N = 1000), but also for sample sizes taking into account different participant exclusion-rates due to exclusion criteria defined in the Registered Report.

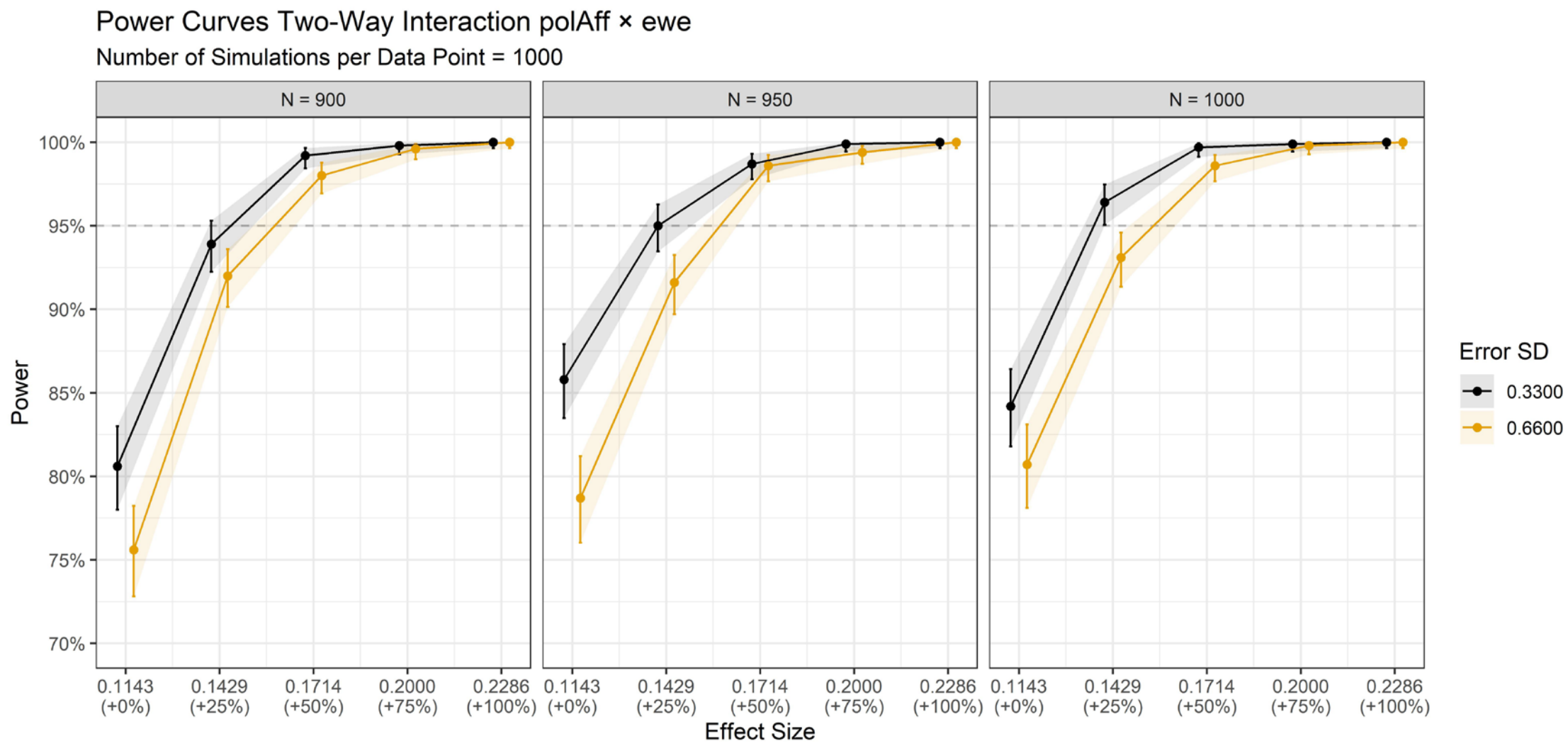

Figure S26: ***Power curves for the two-way interaction polAff × ewe.*** *Points represent simulated power surrounded by a 95%-CI based on 1000 simulations with α = 0.05. Note that, in contrast to Figure S23, the x-axsis represents different effect sizes, starting from the defined SESOI, while the panels represent different sample sizes, taking into account participant exclusion-rates of 10% (N = 900), 5% (N = 950), and 0% (N = 1000). Note that estimates are displayed with a slight shift along the x-axis to reduce overlap. Since we only consider effect sizes equal to or greater than the SESOI to be of relevance, power curves were only examined for positive effect sizes, which is why only positive values are shown.*

To assess the smallest effect size that can be detected with 95% statistical power, we inspect the lower bounds of the 95%-CI power estimates in Figure S26. Specifically, we focus on the power simulation results for N = 950, which takes into account a participant exclusion-rate of 5%. There, we interpolate between the two point estimates that lie just below and above the 95% power line, i.e., between the power estimates for effect sizes 0.1429 and 0.1714. Assuming an error SD of 0.3300, we achieve 95% statistical power to detect a two-way interaction effect of at least 0.1530. For a more conservative error SD of 0.6600, this smallest detectable effect size is only marginally higher (0.1619).

Increased Sample Size

We additionally conduct an effect-size sensitivity analysis for increased sample sizes.

```r
# Load power simulation data
resList_2wayInt <- readRDS(file.path("../powerSimulationsOutput", "res_2wayInt_increa
sedN_20250308_0703.RDS"))
resList_2wayInt.summary <- resList_2wayInt$res_2wayInt.summary

# Extract power values for some specific assumptions
chosenN <- 2000
chosenSigma <- c("0.3300", "0.6600")
chosenSESOI <- c("0.1429", "0.1714")
powerValues <- resList_2wayInt.summary %>%
  filter(sigma_fact %in% chosenSigma) %>%
  filter(sesoi_fact %in% chosenSESOI) %>%
  filter(nSubjects %in% chosenN) %>%
  mutate(power_str = paste0(round(power*100, 2), "%")) %>%
  pull(power_str)

# Extract number of simulations
label_nSimulations <- resList_2wayInt$res_2wayInt$sim %>% n_distinct()

# Repeat breaks_sesoi
breaks_sesoi <- (0.4/3.5)*seq(1, 2, .25)
```

Figure S26 shows results of our effect-size sensitivity analyses. We plot statistical power (y-axis) for different effect sizes (x-axis), taking into account different assumptions for the error SD (color) and sample size (panel). Regarding the latter, we report results not only for the full sample size we aim for (N = 1000), but also for sample sizes taking into account different participant exclusion-rates due to exclusion criteria defined in the Registered Report.

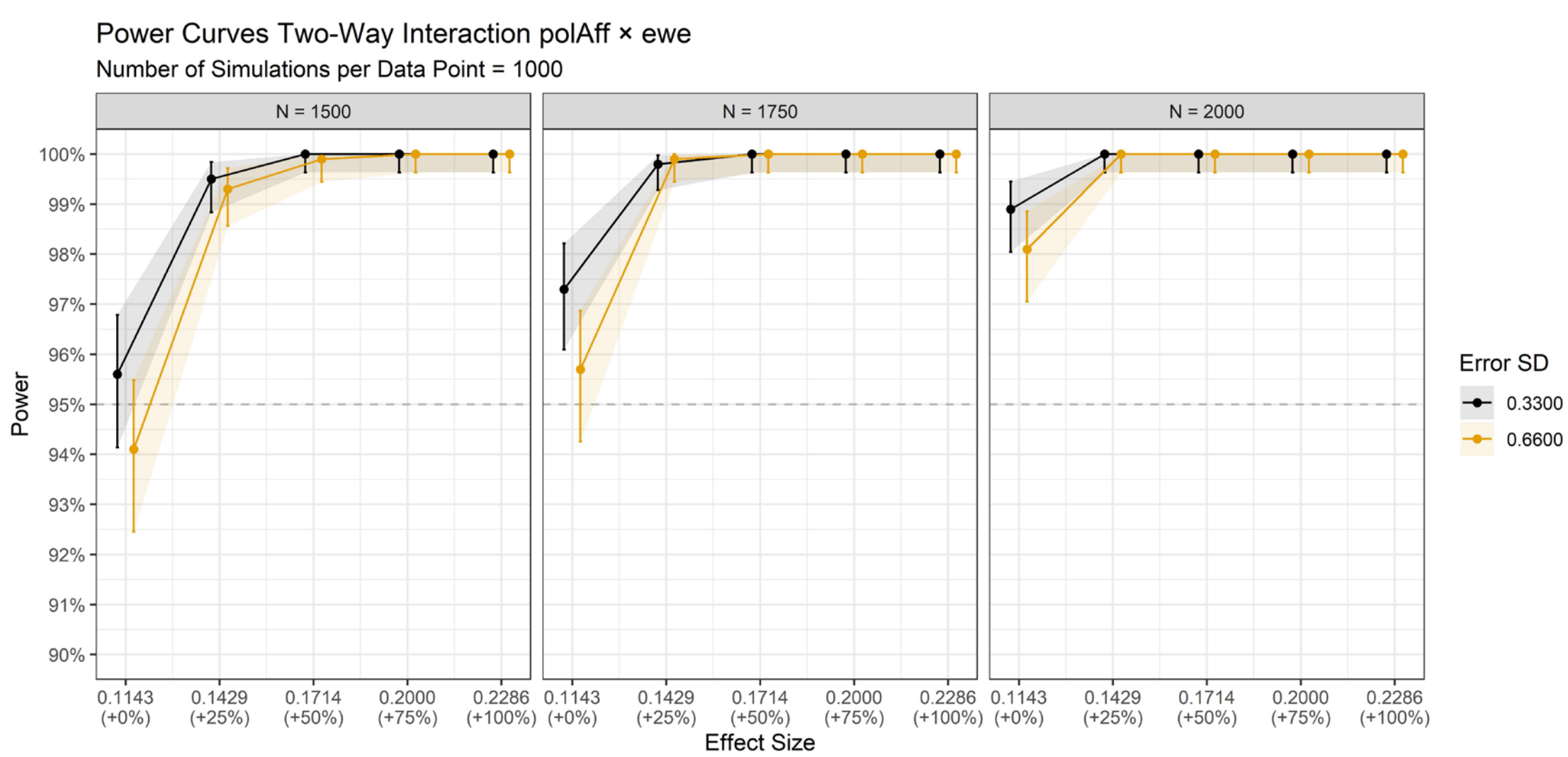

Figure S27: ***Power curves for the two-way interaction polAff × ewe.*** *Points represent simulated power surrounded by a 95%-CI based on 1000 simulations with α = 0.05. Note that, in contrast to Figure S23, the x-axsis represents different effect sizes, starting from the defined SESOI, while the panels represent different sample sizes. Note that estimates are displayed with a slight shift along the x-axis to reduce overlap. Since we only consider effect sizes equal to or greater than the SESOI to be of relevance, power curves were only examined for positive effect sizes, which is why only positive values are shown.*

2.7.6. Three-Way Interaction Effect

As argued in the Registered Report, we hypothesize:

(H_3): *The two-way interaction effect of extreme weather exposure and political affiliation is greater for individuals who more strongly attribute extreme weather events to climate change. In other words, there is a positive three-way interaction effect of political affiliation, exposure to extreme weather events, and their attribution to climate change on ΔDuration.*

```
# Smallest Effect Size Of Interest (SESOI)
SESOI <- 0.4/3.5

# Betas
beta_p <- SESOI
beta_e <- 0
beta_s <- 0
beta_p_e_inx <- SESOI
beta_p_s_inx <- 0
beta_e_s_inx <- 0
beta_p_e_s_inx <- SESOI

# Predicted "true" effects
polAff_ewe_subjAttr_trueEffects <- expand_grid(
  polAff = factor(c("rep", "dem"), levels = c("rep", "dem")),
  ewe = factor(c(FALSE, TRUE), levels = c(FALSE, TRUE)),
  subjAttr = factor(c("-SD", "M", "+SD"), levels = c("-SD", "M", "+SD"))
) %>%
  add_contrast("polAff", contrast = "anova", colnames = "X_p") %>%
  add_contrast("ewe", contrast = "anova", colnames = "X_e") %>%
  add_contrast("subjAttr", contrast = "anova", colnames = c("X_s_1", "X_s_2")) %>%
  mutate(
    trueDeltaDuration =
      0 +                                       # intercept
      X_p * beta_p +                            # main effect polAff
      X_e * beta_e +                            # main effect ewe
      X_s_1 * beta_s +                          # main effect subjAttr, dummy variable
for M vs. -SD
      X_s_2 * (2 * beta_s) +                    # main effect subjAttr, dummy variable
for +SD vs. -SD
      X_p * X_e * beta_p_e_inx +                # 2-way interaction polAff * ewe
      X_p * X_s_1 * beta_p_s_inx +              # 2-way interaction polAff * subjAttr,
dummy variable for M vs. -SD
      X_p * X_s_2 * (2 * beta_p_s_inx) +        # 2-way interaction polAff * subjAttr,
dummy variable for +SD vs. -SD
      X_e * X_s_1 * beta_e_s_inx +              # 2-way interaction ewe * subjAttr, du
mmy variable for M vs. -SD
      X_e * X_s_2 * (2 * beta_e_s_inx) +        # 2-way interaction ewe * subjAttr, du
mmy variable for +SD vs. -SD
      X_p * X_e * X_s_1 * beta_p_e_s_inx +      # 3-way interaction polAff*ewe*subjAtt
r, dummy variable for M vs -SD
      X_p * X_e * X_s_2 * (2 * beta_p_e_s_inx) # 3-way interaction polAff*ewe*subjAtt
r, dummy variable for +SD vs. -SD
  )
```

2.7.6.1. SESOI for Three-way Interaction

In the sections above, we derived the SESOI used for the main effect sample-size determination analysis and for the two-way interaction effect-size sensitivity analysis for the binary variables `polAff` (dem vs. rep) and `ewe` (TRUE vs. FALSE). `subjAttr`, however, is a continuous variable that ranges from 1 to 5. Fortunately, in finding a theoretically sound SESOI for the three-way interaction, the same considerations apply as for the main effect and two-way interaction before. We just need to translate these considerations into the continuous metric of subjAttr.

We start by noticing that the complete fixed three-way interaction polAff × ewe × subjAttr is modeled as:

$$\begin{aligned} \Delta Duration = \beta_0 + \\ \beta_1 \cdot polAff + \beta_2 \cdot ewe + \beta_3 \cdot subjAttr + \\ \beta_4 \cdot (polAff \times ewe) + \beta_5 \cdot (polAff \times subjAttr) + \beta_6 \cdot (ewe \times subjAttr) + \\ \beta_7 \cdot (polAff \times ewe \times subjAttr) \end{aligned}$$

By rearranging terms, one can show that the two-way interaction polAff × ewe is given by:

$$Two - way\ Interaction_{polAff \times ewe} = \beta_4 + \beta_7 \cdot subjAttr$$

Now, let's calculate this two-way interaction for two individuals who differ in their level of subjective attribution of extreme weather events to climate change. First, an individual who has an average score on subjAttr will show the following two-way interaction effect, with $\mu_{subjAttr}$ being the sample average of the variable subjAttr:

$$Effect_{Avg} = \beta_4 + \beta_7 \cdot \mu_{subjAttr}$$

Second, we define an individual with a low score on subjAttr as one that shows a subjective attribution of one SD bellow the average. This individual will show the following two-way interaction effect, with $\sigma_{subjAttr}$ being the SD of subjAttr:

$$Effect_{Low} = \beta_4 + \beta_7 \cdot (\mu_{subjAttr} - \sigma_{subjAttr})$$

The difference in the two-way interaction effect polAff × ewe between these two individuals is given by:

$$\begin{aligned} Effect_{Avg} - Effect_{Low} = \\ \beta_4 + \beta_7 \cdot \mu_{subjAttr} - \left(\beta_4 + \beta_7 \cdot (\mu_{subjAttr} - \sigma_{subjAttr})\right) = \\ \beta_7 \cdot \left[\mu_{subjAttr} - (\mu_{subjAttr} - \sigma_{subjAttr})\right] = \\ \beta_7 \cdot \sigma_{subjAttr} \end{aligned}$$

As outlined above in Section Section 2.7.5.1, we assume that the SESOI for the two-way interaction polAff × ewe is 0.1143 for an average individual (with respect to subjAttr). If the same two-way interaction polAff × ewe shrinks to zero for an individual low in subjAttr, we would consider this interaction effect difference as theoretically relevant (see Figure S28). These assumptions translate to:

$$Effect_{Avg} - Effect_{Low} = 0.1143 = \beta_7 \cdot \sigma_{subjAttr}$$

Division by $\sigma_{subjAttr}$ gives us the SESOI for the three-way interaction in the suitable metric of subjAttr:

$$SESOI_{polAff \times ewe \times subjAttr} = \frac{0.1143}{\sigma_{subjAttr}}$$

We will assess subjective attribution of extreme weather events to climate change using the same questions, response options, and aggregation as Ogunbode et al. (2019). These authors reported $\mu_{subjAttr}$ = 3.67 and $\sigma_{subjAttr}$ = 0.85, resulting in:

$$SESOI_{polAff \times ewe \times subjAttr} = \frac{0.1143}{0.85} = 0.1345$$

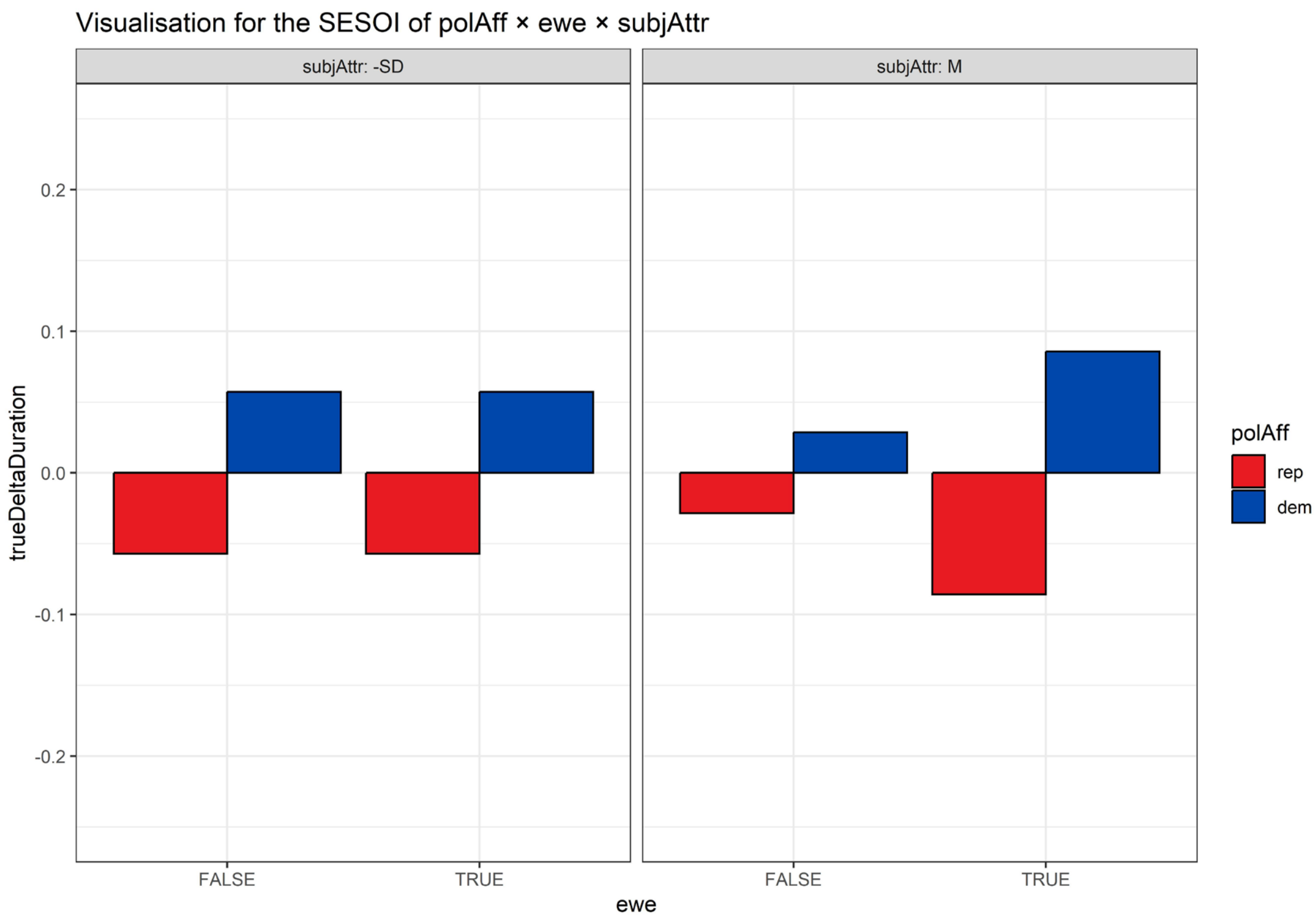

Figure S28: ***Visualisation for the SESOI of polAff x ewe x subjAttr*** *Assumed ΔDuration values for individuals scoring low (-SD) and average (M) on subjAttr (red = Republicans; blue = Democrats). While individuals scoring average on subjAttr show a two-way interaction effect of polAff × ewe of 0.1143, this effect shrinks to zero for individuals scoring low on subjAttr. These individuals only show the predicted main effect of polAff (which is 0.1143).*

2.7.6.2. Data Simulation Function

We finally define a function that simulates data for the three-way interaction effect of political affiliation with extreme weather exposure and attribution of extreme weather events to climate change on ΔDuration: `FUN_sim_3wayInt`. The function will simulate data according to the following model, expressed in lme4-lingo:

```
deltaDuration ~ polAff * ewe * subjAttr + (1|subj) + (1|trial)
```

The function `FUN_sim_3wayInt` takes, among others, the following important arguments (in addition to the arguments discussed for `FUN_sim_2wayInt`):

- `s_mean`: **Sample mean of subjective attribution**. Based on results reported by Ogunbode et al. (2019), we set this value to 3.67.
- `s_sd`: **Sample SD of subjective attribution**. Based on results reported by Ogunbode et al. (2019), we set this value to 0.85.
- `beta_s`: **Fixed main effect of subjective attribution**. As we are interested in the moderating role of subjective attribution of extreme weather events to climate change, we set this main effect to zero.
- `beta_p_s_inx` **Fixed two-way interaction effect of political affiliation and subjective attribution**. In order to accurately model a three-way interaction, one needs to include all two-way interactions in statistical models. Therefore, we include this two-way interaction, but we assume it to be zero.
- `beta_e_s_inx` **Fixed two-way interaction effect of extreme weather exposure and subjective attribution**. As before, we include this interaction for accurately modelling the three-way interaction of interest, but we assume this two-way interaction to be zero.
- `beta_p_e_s_inx` **Fixed three-way interaction effect of political affiliation, extreme weather exposure, and subjective attribution**. We set this initial value to the SESOI derived above, i.e. 0.1345. We investigate how changing this effect size impacts statistical power, as we are conducting effect-size sensitivity analyses for interaction effects.

The function is defined below:

We call the function once and extract the results of this single simulation:

Figure S29 visualizes predictions based on this single simulation and Table S29 summarizes the statistical results of fitting the actual model used in data generation to the simulated data.

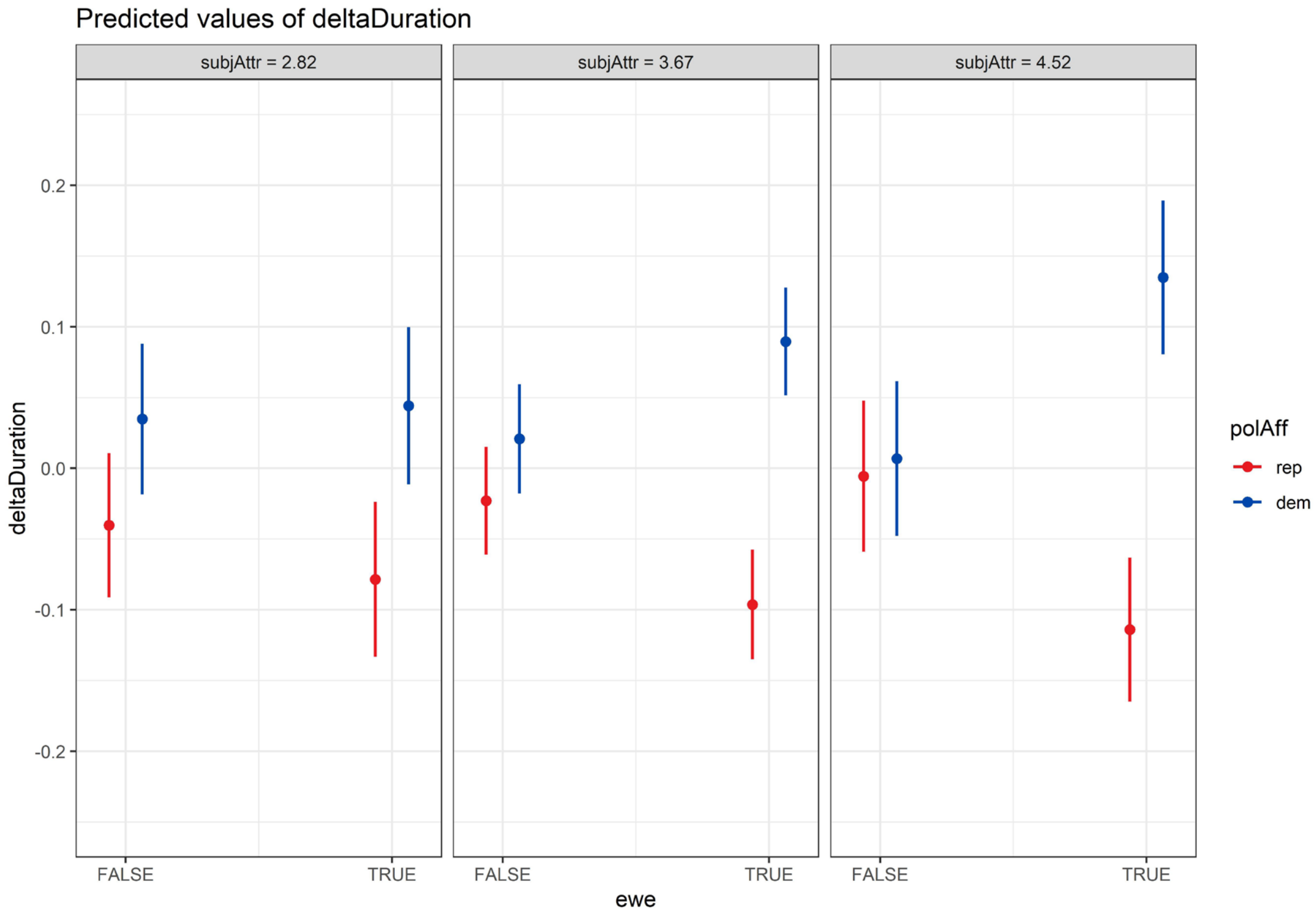

*Figure S29: **Predicted values of deltaDuration.** Visual representation of results of one simulation created using FUN_`sim_3wayInt`. Points indicate the predicted means surrounded by 95% Confidence Intervals (red = Republicans; blue = Democrats). Panels indicate predictions for different values of subjAttr (Mean - 1 SD, Mean, and Mean + 1 SD).*

effect	group	term	estimate	p.value
fixed	NA	(Intercept)	-0.0022	0.8478
fixed	NA	polAffX_p	0.1149	0.0000
fixed	NA	eweX_e	-0.0023	0.9031
fixed	NA	subjAttr_c	0.0091	0.4141
fixed	NA	polAffX_p:eweX_e	0.1426	0.0001
fixed	NA	polAffX_p:subjAttr_c	0.0187	0.4025
fixed	NA	eweX_e:subjAttr_c	0.0142	0.5239
fixed	NA	polAffX_p:eweX_e:subjAttr_c	0.1111	0.0130
ran_pars	subj	sd__(Intercept)	0.2849	NA
ran_pars	trial	sd__(Intercept)	0.0323	NA
ran_pars	Residual	sd__Observation	0.3240	NA

*Table S29: **Statistical results of one simulation.** Data were simulated using FUN_`sim_3wayInt` and fitted using deltaDuration ~ polAff * ewe * subjAttr_c + (1 | subj) + (1 | trial). Note that subjAttr is mean centered to ease interpretation of lower-level interactions and main effects.*

2.7.6.3. Power Simulation

In the following code, the simulations are calculated. We do not recommend executing this code junk as it takes several hours to run.

We retrieve pre-run results:

```
# Load power simulation data
resList_3wayInt <- readRDS(file.path("../powerSimulationsOutput", "res_3wayInt_202408
14_1612.RDS"))
resList_3wayInt.summary <- resList_3wayInt$res_3wayInt.summary

# Extract power values for some specific effect sizes at N = 1000
powerValues <- resList_3wayInt.summary %>%
  filter(sigma_fact == "0.3300") %>%
  filter(sesoi_fact == "0.1345") %>%
  filter(nSubjects == 950) %>%
  mutate(power_str = paste0(round(power*100, 2), "%")) %>%
  pull(power_str)

# Extract number of simulations
label_nSimulations <- resList_3wayInt$res_3wayInt$sim %>% n_distinct()

# Repeat breaks_sesoi
breaks_sesoi <- (0.4/3.5)/(0.85) * seq(1, 2, .25)
```

Figure S30 displays the distribution of estimated fixed effects across all simulations. The figure shows that the estimated fixed effects are close to the true ones provided as input in the data simulation function, validating that simulations worked as expected.

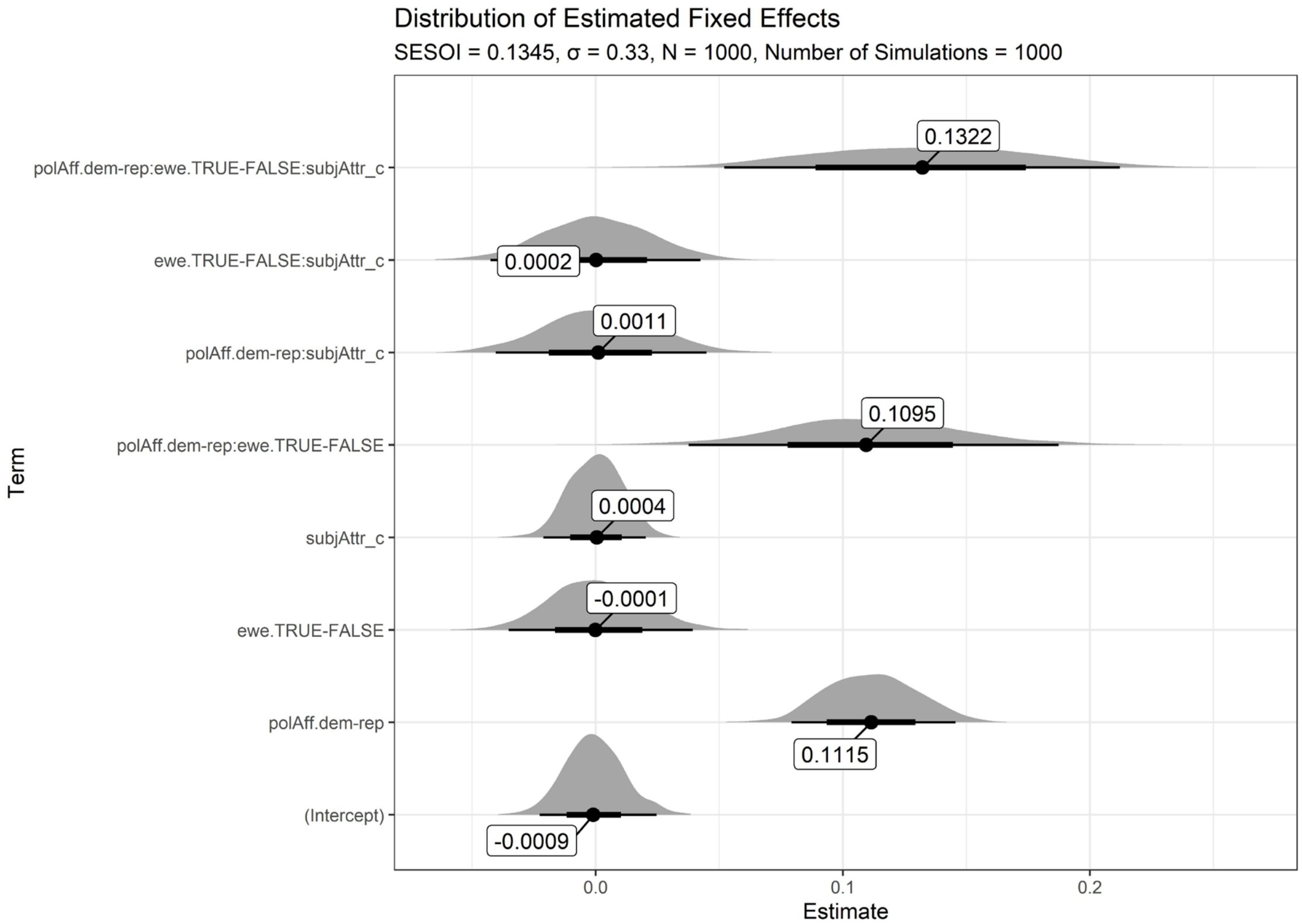

Figure S30: ***Distribution of estimated fixed effects.*** *The figure shows the estimated fixed effects obtained from 1000 simulations for the model deltaDuration ~ polAff * ewe * subjAttr_c + (1 | subj) + (1 | trial). Shaded area represent densities, annotated points indicate medians, and thick and thin lines represent 66% and 95% quantiles, respectively.*

Figure S31 shows results of our effect-size sensitivity analyses. We plot statistical power (y-axis) for different effect sizes (x-axis), taking into account different assumptions for the error SD (color) and sample size (panel). Regarding the latter, we report results not only for the full sample size we aim for (N = 1000), but also for sample sizes taking into account different participant exclusion-rates due to exclusion criteria defined in the Registered Report.

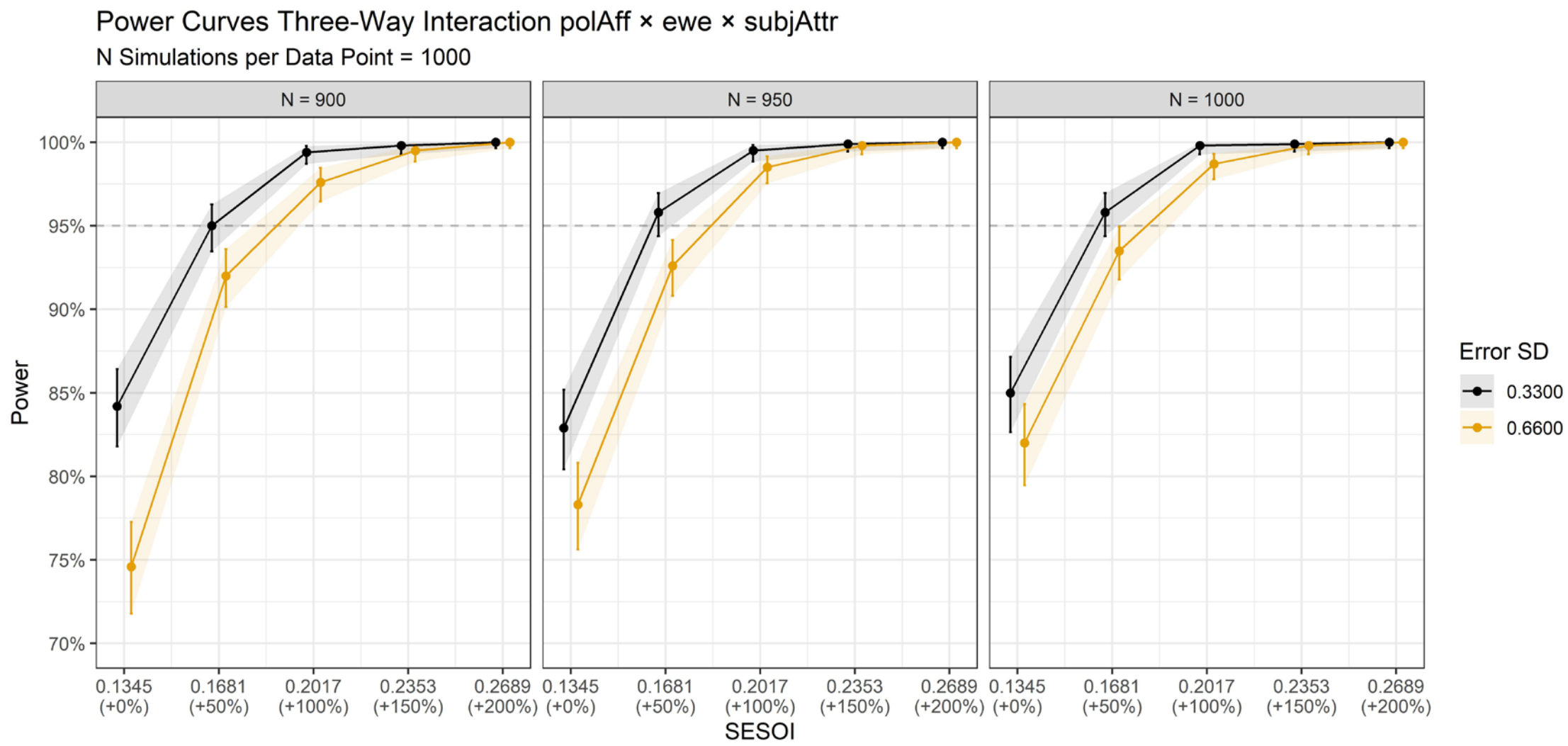

Figure S31: ***Power curves for the three-way interaction polAff × ewe × subjAttr.*** *Points represent simulated power surrounded by a 95%-CI based on 1000 simulations with α = 0.05. Note that, in contrast to Figure S23, the x-axsis represents different effect sizes, starting from the defined SESOI, while the panels represent different sample sizes, taking into account participant exclusion-rates of 10% (N = 900), 5% (N = 950), and 0% (N = 1000). Note that estimates are displayed with a slight shift along the x-axis to reduce overlap. Since we only consider effect sizes equal to or greater than the SESOI to be of relevance, power curves were only examined for positive effect sizes, which is why only positive values are shown.*

To assess the smallest effect size that can be detected with 95% statistical power, we inspect the lower bounds of the 95%-CI power estimates in Figure S31. Specifically, we focus on the power simulation results for N = 950, which takes into account a participant exclusion rate of 5%. There, we interpolate between the two point estimates that lie just below and above the 95% power line, i.e., between the power estimates for effect sizes 0.1681 and 0.2017. Assuming an error SD of 0.3300, we achieve 95% statistical power to detect a three-way interaction effect of at least 0.1728. For a more conservative error SD of 0.6600, this smallest detectable effect size is only marginally higher (0.1890).

Increased Sample Size

We additionally conduct an effect-size sensitivity analysis for increased sample sizes.

```
# Load power simulation data
resList_3wayInt <- readRDS(file.path("../powerSimulationsOutput", "res_3wayInt_increasedN_20250308_1649.RDS"))
resList_3wayInt.summary <- resList_3wayInt$res_3wayInt.summary

# Extract power values for some specific effect sizes at N = 1000
powerValues <- resList_3wayInt.summary %>%
  filter(sigma_fact == "0.3300") %>%
  filter(sesoi_fact == "0.1345") %>%
  filter(nSubjects == 950) %>%
  mutate(power_str = paste0(round(power*100, 2), "%")) %>%
  pull(power_str)

# Extract number of simulations
label_nSimulations <- resList_3wayInt$res_3wayInt$sim %>% n_distinct()

# Repeat breaks_sesoi
breaks_sesoi <- (0.4/3.5)/(0.85) * seq(1, 2, .25)
```

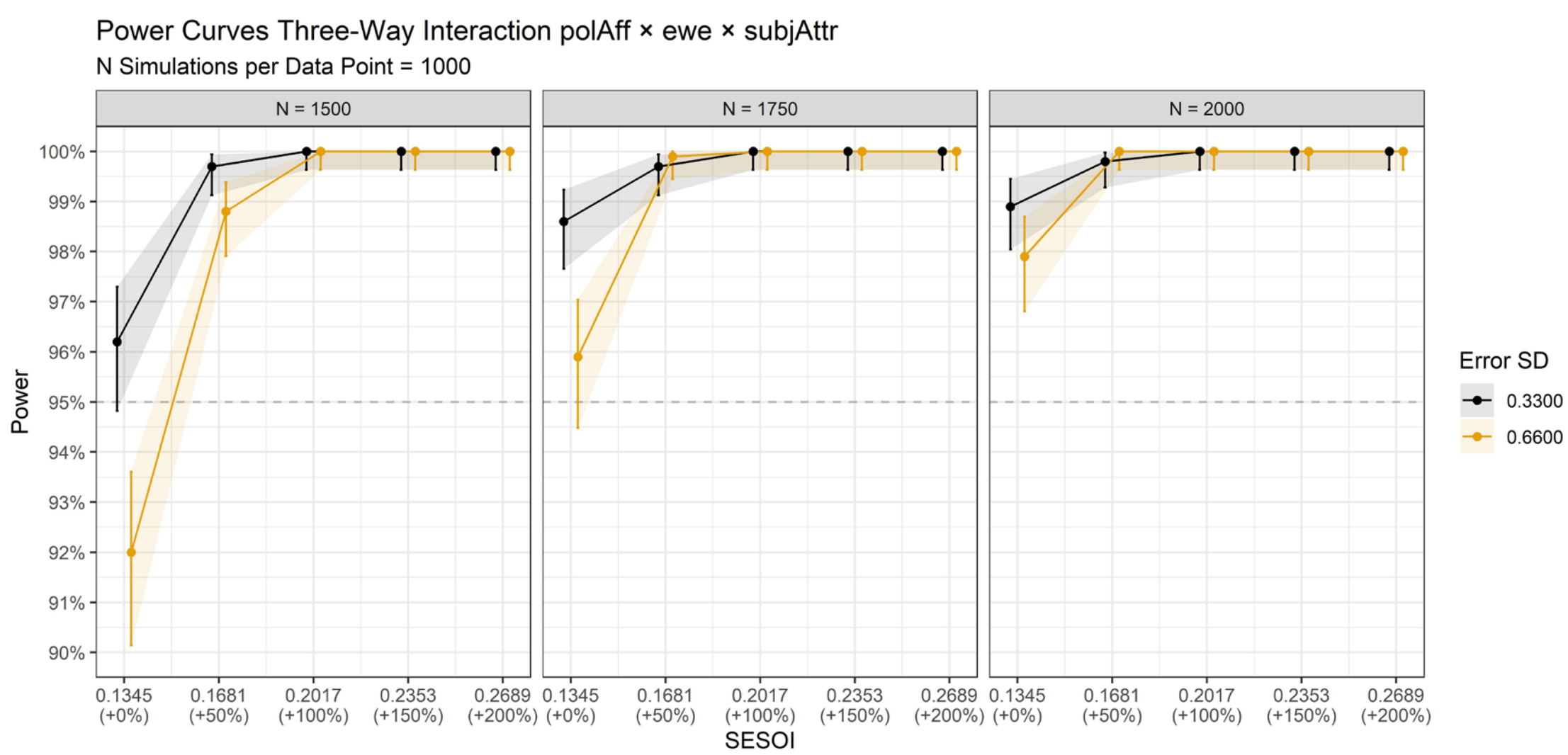

*Figure S32: **Power curves for the three-way interaction polAff × ewe × subjAttr.** Points represent simulated power surrounded by a 95%-CI based on 1000 simulations with α = 0.05. Note that, in contrast to Figure S23, the x-axsis represents different effect sizes, starting from the defined SESOI, while the panels represent different sample sizes. Note that estimates are displayed with a slight shift along the x-axis to reduce overlap. Since we only consider effect sizes equal to or greater than the SESOI to be of relevance, power curves were only examined for positive effect sizes, which is why only positive values are shown.*

2.7.7. Conclusion

Using power simulations with mixed-effects models for sample-size determination and effect-size sensitivity analyses, we show that with a final sample size of N = 950:

1. We will achieve ≥ 95% statistical power to detect a main effect of political affiliation.

2. We will be able to detect a two-way interaction effect of political affiliation with extreme weather exposure of at least 0.1619 with ≥ 95% statistical power.
3. We will be able to detect a three-way interaction effect of political affiliation with extreme weather exposure and subjective attribution of extreme weather events to climate change of at least 0.1890 with ≥ 95% statistical power.

By increasing the planned sample size to N = 2'000, we will be able to detect all of the defined smallest effect sizes of interest of the hypothesized main effect and the two- and three-way interaction effects with a statistical power of at least 95%.

2.8. Sample Characteristics

Data were collected between August and September 2025. The sample (N = 2,000) was quota-matched to the U.S. population on political affiliation, age, gender, and region, with a deviation tolerance of <5%. Table S30 reports the final distribution of respondents (absolute and relative frequencies) across all quota criteria. After applying all exclusion criteria, the final analytic sample consisted of n = 1,451 respondents. Table S31 summarizes the sociodemographic characteristics of this final sample.

A

Age	Gender	*N*	Target (%)	Observed (%)	Difference (pp)
18-25	Female	138	5.9%	6.9%	0.96
	Male	164	6.1%	8.2%	2.12
26-35	Female	170	8.6%	8.5%	-0.03
	Male	206	8.5%	10.3%	1.82
36-45	Female	157	8.0%	7.9%	-0.18
	Male	162	8.0%	8.1%	0.17
46-55	Female	168	8.0%	8.4%	0.47
	Male	178	8.0%	8.9%	0.87
56-65	Female	162	7.4%	8.1%	0.72
	Male	136	7.6%	6.8%	-0.79
66-74	Female	121	6.0%	6.1%	0.01
	Male	101	5.9%	5.1%	-0.84
75-85	Female	75	6.0%	3.8%	-2.29
	Male	58	5.9%	2.9%	-2.99

B

Region	*N*	Target (%)	Observed (%)	Difference (pp)
Midwest	364	20.5%	18.2%	-2.30
Northeast	304	17.0%	15.2%	-1.80
South	782	39.0%	39.1%	0.10
West	550	23.5%	27.5%	4.00

C

Party	*N*	Target (%)	Observed (%)	Difference (pp)
Democrat	1'005	50.0%	50.2%	0.25
Republican	995	50.0%	49.8%	-0.25

Table S30: ***Overview of sample characteristics and target quotas.*** *Panel A reports sample characteristics by age and gender. Panel B reports regional distribution. Panel C reports political affiliation. Target and observed proportions are expressed in percent; differences are expressed in percentage points (N = 2,000).*

A

**Variable**	**Category**	***n***	**%**
Gender	Male	720	49.66
	Female	730	50.34
Education	Did not complete high school / GED	10	0.69
	High school diploma / GED	123	8.48
	Some college	203	14.00
	Associates degree	131	9.03
	Bachelor's degree	635	43.79
	Master's degree or higher	348	24.00
Income	Under $30,000	146	10.07
	$30,000 - $49,999	161	11.10
	$50,000 - $74,999	209	14.41
	$75,000 - $99,999	233	16.07
	$100,000 - $149,999	465	32.07
	More than $150,000	236	16.28
Race/Ethnicity	African	54	3.72
	Asian	48	3.31
	European	774	53.38
	Hispanic or Latinx	407	28.07
	More than one	44	3.03
	Other	123	8.48
Residential area	Rural town or small city	227	15.66
	Suburb, outskirt of a big city	461	31.79
	Urban, a big city	762	52.55

B

**Variable**	***M***	***SD***	**Min**	**Max**
Age	48.26	18.09	18.00	89.00

Table S31: ***Overview of participants' sociodemographic characteristics.*** *Panel A displays frequencies of categorical variables, whereas Panel B reports descriptive statistics for age (N = 1,451 participants).*

2.9. References

Berger, Sebastian, and Annika M Wyss. 2021. “Measuring Pro-Environmental Behavior Using the Carbon Emission Task.” *Journal of Environmental Psychology* 75: 101613. https://doi.org/https://doi.org/10.1016/j.jenvp.2021.101613.

Francoeur, Richard B. 2013. “Could Sequential Residual Centering Resolve Low Sensitivity in Moderated Regression? Simulations and Cancer Symptom Clusters.” *Open Journal of Statistics* 3 (6): 24–44. https://doi.org/10.4236/ojs.2013.36A004.

Ghaffari, Minou, and Susann Fiedler. 2018. “The Power of Attention: Using Eye Gaze to Predict Other-Regarding and Moral Choices.” *Psychological Science* 29 (11): 1878–89. https://www.jstor.org/stable/26957512.

Giner-Sorolla, Roger, Amanda K. Montoya, Alan Reifman, Tom Carpenter, Neil A. Lewis, Christopher L. Aberson, Dries H. Bostyn, et al. 2024. “Power to Detect What? Considerations for Planning and Evaluating Sample Size.” *Personality and Social Psychology Review*, February, 10888683241228328. https://doi.org/10.1177/10888683241228328.

Gwinn, Rachael, Andrew B. Leber, and Ian Krajbich. 2019. “The Spillover Effects of Attentional Learning on Value-Based Choice.” *Cognition* 182 (January): 294–306. https://doi.org/10.1016/j.cognition.2018.10.012.

IPCC, ed. 2023. “Weather and Climate Extreme Events in a Changing Climate.” In, 1513–1766. Cambridge: Cambridge University Press. https://doi.org/10.1017/9781009157896.013.

Konisky, David M., Llewelyn Hughes, and Charles H. Kaylor. 2016. “Extreme Weather Events and Climate Change Concern.” *Climatic Change* 134 (4): 533–47. https://doi.org/10.1007/s10584-015-1555-3.

NWS. 2016. “Storm Data Preparation. National Weather Service Instruction 10-1605; Department of Commerce, National Oceanic & Atmospheric Administration.” Silver Spring.

Ogunbode, Charles A., Christina Demski, Stuart B. Capstick, and Robert G. Sposato. 2019. “Attribution Matters: Revisiting the Link Between Extreme Weather Experience and Climate Change Mitigation Responses.” *Global Environmental Change* 54 (January): 31–39. https://doi.org/10.1016/j.gloenvcha.2018.11.005.

Pärnamets, Philip, Petter Johansson, Lars Hall, Christian Balkenius, Michael J. Spivey, and Daniel C. Richardson. 2015. “Biasing Moral Decisions by Exploiting the Dynamics of Eye Gaze.” *Proceedings of the National Academy of Sciences* 112 (13): 4170–75. https://doi.org/10.1073/pnas.1415250112.

Rahal, Rima-Maria, and Susann Fiedler. 2019. “Understanding Cognitive and Affective Mechanisms in Social Psychology Through Eye-Tracking.” *Journal of Experimental Social Psychology* 85 (November): 103842. https://doi.org/10.1016/j.jesp.2019.103842.

Reeck, Crystal, Daniel Wall, and Eric J. Johnson. 2017. “Search Predicts and Changes Patience in Intertemporal Choice.” *Proceedings of the National Academy of Sciences* 114 (45): 11890–95. https://doi.org/10.1073/pnas.1707040114.

Salthouse, Timothy A. 2000. “Aging and Measures of Processing Speed.” *Biological Psychology* 54 (1): 35–54. https://doi.org/10.1016/S0301-0511(00)00052-1.

Sisco, Matthew R., Valentina Bosetti, and Elke U. Weber. 2017. “When Do Extreme Weather Events Generate Attention to Climate Change?” *Climatic Change* 143 (1): 227–41. https://doi.org/10.1007/s10584-017-1984-2.

US Census Bureau. 2023. “Average Household Size and Population Density - County.”

Willemsen, Martijn C., and Eric J. Johnson. 2019. “(Re)Visiting the Decision Factory: Obswerving Cognition with MouselabWEB.” In, edited by Michael Schulte-Mecklenbeck, Anton Kuehberger, and Joseph G. Johnson, 2nd ed., 76–95. New York: Routledge. https://doi.org/10.4324/9781315160559.

Zanocco, Chad, Philip Mote, June Flora, and Hilary Boudet. 2024. “Comparing Public and Scientific Extreme Event Attribution to Climate Change.” *Climatic Change* 177 (5): 76. https://doi.org/10.1007/s10584-024-03735-0.
