# Supplementary material for: Political affiliation impacts climate attention and choice beyond extreme weather exposure
Source: Nat Clim Chang. 2026 Oct 16;16(11):1332–8. doi: 10.1038/s41558-026-02765-z (PMC13731144; doi:10.1038/s41558-026-02765-z)
Supplement: Supplementary file 2 [file 41558_2026_2765_MOESM2_ESM.pdf]

nature portfolio

Corresponding author(s): Prof. Daria Knoch, Prof. Ulf J.J. Hahnel

Last updated by author(s): 2026/08/18

Reporting Summary

Nature Portfolio wishes to improve the reproducibility of the wReork that we publish. This form provides structure for consistency and transparency in reporting. For further information on Nature Portfolio policies, see our Editorial Policies and the Editorial Policy Checklist.

Please do not complete any field with "not applicable" or n/a. Refer to the help text for what text to use if an item is not relevant to your study.
For final submission: please carefully check your responses for accuracy; you will not be able to make changes later.

Statistics

For all statistical analyses, confirm that the following items are present in the figure legend, table legend, main text, or Methods section.

n/a	Confirmed	
☐	☒	The exact sample size (*n*) for each experimental group/condition, given as a discrete number and unit of measurement
☐	☒	A statement on whether measurements were taken from distinct samples or whether the same sample was measured repeatedly
☐	☒	The statistical test(s) used AND whether they are one- or two-sided<br>*Only common tests should be described solely by name; describe more complex techniques in the Methods section.*
☐	☒	A description of all covariates tested
☐	☒	A description of any assumptions or corrections, such as tests of normality and adjustment for multiple comparisons
☐	☒	A full description of the statistical parameters including central tendency (e.g. means) or other basic estimates (e.g. regression coefficient) AND variation (e.g. standard deviation) or associated estimates of uncertainty (e.g. confidence intervals)
☐	☒	For null hypothesis testing, the test statistic (e.g. *F*, *t*, *r*) with confidence intervals, effect sizes, degrees of freedom and *P* value noted<br>*Give P values as exact values whenever suitable.*
☒	☐	For Bayesian analysis, information on the choice of priors and Markov chain Monte Carlo settings
☐	☒	For hierarchical and complex designs, identification of the appropriate level for tests and full reporting of outcomes
☒	☐	Estimates of effect sizes (e.g. Cohen's *d*, Pearson's *r*), indicating how they were calculated

Our web collection on statistics for biologists contains articles on many of the points above.

Software and code

Policy information about availability of computer code

Data collection	Data were collected using Qualtrics XM for survey administration and MouselabWEB to record participants' information acquisition process during the decision-making task. No custom code was used for data collection
Data analysis	All analyses were conducted in R (version 4.5.2). Packages: apa7 (0.1.0), bayestestR (0.17.0), binom (1.1-1.1), correlation (0.8.8), cowplot (1.2.0), datawizard (1.3.0), dplyr (1.2.0), easystats (0.7.5), effectsize (1.0.2), emmeans (2.0.2), faux (1.2.4), flextable (0.9.11), forcats (1.0.1), fs (1.6.7), ggdist (3.3.3), ggeffects (2.3.2), ggplot2 (4.0.2), ggpubr (0.6.3), ggrepel (0.9.7), ggthemes (5.2.0), here (1.0.2), insight (1.4.6), interactions (1.2.0), jtools (2.3.1), kableExtra (1.4.0), knitr (1.51), lme4 (2.0-1), lmerTest (3.2-1), lubridate (1.9.5), Matrix (1.7-4), mirai (2.6.1), modelbased (0.14.0), parameters (0.28.3), performance (0.16.0), purrr (1.2.1), readr (2.2.0), readxl (1.4.5), report (0.6.3), see (0.13.0), sessioninfo (1.2.3), sf (1.1-0), sjPlot (2.9.0), stringr (1.6.0), tibble (3.3.1), tictoc (1.2.1), tidyr (1.3.2), tidyverse (2.0.0), usmap (1.0.0), viridisLite (0.4.3). All data and materials are openly available on https://github.com/JulieBuchs/EcoTRACE

For manuscripts utilizing custom algorithms or software that are central to the research but not yet described in published literature, software must be made available to editors and reviewers. We strongly encourage code deposition in a community repository (e.g. GitHub). See the Nature Portfolio guidelines for submitting code & software for further information.

Data

Policy information about availability of data

All manuscripts must include a data availability statement. This statement should provide the following information, where applicable:

- Accession codes, unique identifiers, or web links for publicly available datasets
- A description of any restrictions on data availability
- For clinical datasets or third party data, please ensure that the statement adheres to our policy

All data and materials are openly available on https://github.com/JulieBuchs/EcoTRACE

Research involving human participants, their data, or biological material

Policy information about studies with human participants or human data. See also policy information about sex, gender (identity/presentation), and sexual orientation and race, ethnicity and racism.

Reporting on sex and gender	Sex was collected by self-report for quota matching and demographic characterization. Participants self-reported their gender as part of the demographic questionnaire. Sex and gender were not central variables of interest and were included only as demographic covariates in robustness analyses; no sex- or gender-stratified analyses were performed because the study was not designed or powered to detect such differences. Aggregate demographic characteristics are reported in the manuscript and Supplementary Information.
Reporting on race, ethnicity, or other socially relevant groupings	Participants self-reported their race/ethnicity as part of the demographic questionnaire. Race/ethnicity was collected to characterize the study sample and was not used as a primary predictor in the analyses. Political affiliation, rather than race/ethnicity, was the primary individual-level variable of interest. Race/ethnicity was considered only as a demographic covariate in robustness analyses where applicable.
Population characteristics	The final analytic sample comprised 1,451 U.S. adults from a quota-matched online sample (political affiliation, age, sex, and region; <5% deviation from targets). The sample was designed to approximate the U.S. adult population on these characteristics but was not probability-based. A U.S. sample was chosen to examine partisan differences between Democrats and Republicans. Demographic characteristics are reported in Table S31. Existing county-level data included extreme weather exposure (NOAA Storm Events Database), population density, household income (U.S. Census Bureau SAIPE), and political voting patterns (MIT Election Data and Science Lab).
Recruitment	Participants were recruited through MSI-ACI's FlexPanel, an opt-in online market research panel. Quotas for political affiliation, age, sex, and region were used to approximate the U.S. adult population on these characteristics. Participants received USD 0.95 plus a performance-contingent bonus. As with opt-in online panels, self-selection may limit generalizability beyond the sampled population, as panel participants may differ systematically from the broader U.S. population. Quota matching reduced imbalances on the targeted characteristics but does not eliminate this potential selection bias. Robustness analyses additionally adjusted for relevant demographic and contextual covariates to assess the sensitivity of the findings to observed sample characteristics.
Ethics oversight	The study protocol was approved by the Ethics Commission of the Faculty of Human Sciences, University of Bern (approval no. 2024-09-02). All participants provided informed consent prior to participation, and the study was conducted in accordance with the Declaration of Helsinki.

Note that full information on the approval of the study protocol must also be provided in the manuscript.

Field-specific reporting

Please select the one below that is the best fit for your research. If you are not sure, read the appropriate sections before making your selection.

☐ Life sciences ☒ Behavioural & social sciences ☐ Ecological, evolutionary & environmental sciences

For a reference copy of the document with all sections, see nature.com/documents/nr-reporting-summary-flat.pdf

Behavioural & social sciences study design

All studies must disclose on these points even when the disclosure is negative.

Study description	This study was a preregistered, quantitative, cross-sectional online behavioral experiment combined with survey measures and county-level observational data on objectively measured extreme weather exposure. Participants completed an incentivized decision-making task using MouselabWEB and questionnaires administered in Qualtrics.
Research sample	The study included a quota-matched sample of U.S. adults recruited through MSI-ACI's FlexPanel. Quotas were set for political affiliation, age, sex, and geographic region to approximate the U.S. adult population. After preregistered exclusions, the final analytic sample comprised 1,451 participants. Additional demographic information (e.g., education, household income, race/ethnicity, and urbanicity) was collected and is reported in the manuscript and Supplementary Information.
Sampling strategy	Participants were recruited through an online panel provider using quota sampling to approximate the U.S. adult population with respect to political affiliation, age, sex, and region. The target sample size of 2,000 participants was determined a priori based on preregistered power analyses (see Supplementary Information). Following initial quality screening, participants were randomly downsampled within quota cells to preserve the planned quota structure before preregistered exclusion criteria were applied.
Data collection	Data were collected online. Participants completed questionnaires in Qualtrics and an incentivized decision-making task implemented in MouselabWEB, which recorded mouse-based process-tracing measures of information acquisition. No researchers were present during data collection. Participants completed the study independently on desktop or laptop computers. Allocation to experimental conditions (e.g., randomization of task features) was automated, and researchers were not involved in data collection.
Timing	Data were collected between August and September 2025.
Data exclusions	Exclusion criteria were preregistered before data collection. Of the 2,000 participants included after quota matching, 549 were excluded from the final analyses because of missing process-tracing data, excessive missing trials, insufficient task engagement (e.g., repeated failure to inspect information), or predefined process-tracing outlier criteria, resulting in a final analytic sample of 1,451 participants. Detailed exclusion criteria are provided in the Methods and Supplementary Information.

Non-participation	Before the analytic sample was established, 672 individuals were screened out because they failed the CAPTCHA, did not provide informed consent, failed preregistered attention checks, or did not complete the study. An additional 73 participants were excluded because valid county-level geographic information was unavailable or they resided outside the study area (Alaska or Hawaii). These exclusions occurred before quota matching and formation of the target sample.
Randomization	Participants were not allocated to experimental groups. However, several aspects of the decision-making task were randomized to control for presentation effects. Specifically, (1) the position of carbon versus bonus information (top vs. bottom) was randomized between participants, (2) the location of the higher-bonus option (left vs. right) was randomized within participants across trials, and (3) the order of trials was randomized across participants

Reporting for specific materials, systems and methods

We require information from authors about some types of materials, experimental systems and methods used in many studies. Here, indicate whether each material, system or method listed is relevant to your study. If you are not sure if a list item applies to your research, read the appropriate section before selecting a response.

Materials & experimental systems

n/a	Involved in the study
☒	☐ Antibodies
☒	☐ Eukaryotic cell lines
☒	☐ Palaeontology and archaeology
☒	☐ Animals and other organisms
☒	☐ Clinical data
☒	☐ Dual use research of concern
☒	☐ Plants

Methods

n/a	Involved in the study
☒	☐ ChIP-seq
☒	☐ Flow cytometry
☒	☐ MRI-based neuroimaging

Plants

Seed stocks	*Report on the source of all seed stocks or other plant material used. If applicable, state the seed stock centre and catalogue number. If plant specimens were collected from the field, describe the collection location, date and sampling procedures.*
Novel plant genotypes	*Describe the methods by which all novel plant genotypes were produced. This includes those generated by transgenic approaches, gene editing, chemical/radiation-based mutagenesis and hybridization. For transgenic lines, describe the transformation method, the number of independent lines analyzed and the generation upon which experiments were performed. For gene-edited lines, describe the editor used, the endogenous sequence targeted for editing, the targeting guide RNA sequence (if applicable) and how the editor was applied.*
Authentication	*Describe any authentication procedures for each seed stock used or novel genotype generated. Describe any experiments used to assess the effect of a mutation and, where applicable, how potential secondary effects (e.g. second site T-DNA insertions, mosiacism, off-target gene editing) were examined.*

This checklist template is licensed under a Creative Commons Attribution 4.0 International License, which permits use, sharing, adaptation, distribution and reproduction in any medium or format, as long as you give appropriate credit to the original author(s) and the source, provide a link to the Creative Commons license, and indicate if changes were made. The images or other third party material in this article are included in the article's Creative Commons license, unless indicated otherwise in a credit line to the material. If material is not included in the article's Creative Commons license and your intended use is not permitted by statutory regulation or exceeds the permitted use, you will need to obtain permission directly from the copyright holder. To view a copy of this license, visit http://creativecommons.org/licenses/by/4.0/
